# Comparative Genomic Analysis of *Brucella melitensis* Vaccine Strain M5 Provides Insights into Virulence Attenuation

**DOI:** 10.1371/journal.pone.0070852

**Published:** 2013-08-14

**Authors:** Hai Jiang, Pengcheng Du, Wen Zhang, Heng Wang, Hongyan Zhao, Dongri Piao, Guozhong Tian, Chen Chen, Buyun Cui

**Affiliations:** 1 State Key Laboratory for Infectious Disease Prevention and Control, Collaborative Innovation Center for Diagnosis and Treatment of Infectious Diseases, National Institute for Communicable Disease Control and Prevention, Beijing, China; 2 Department of Endemic and Parasitic Diseases Control and Prevention, Hangzhou Center for Disease Control and Prevention, Hangzhou, China; Washington State University, United States of America

## Abstract

The *Brucella melitensis* vaccine strain M5 is widely used to prevent and control brucellosis in animals. In this study, we determined the whole-genome sequence of M5, and conducted a comprehensive comparative analysis against the whole-genome sequence of the virulent strain 16 M and other reference strains. This analysis revealed 11 regions of deletion (RDs) and 2 regions of insertion (RIs) within the M5 genome. Among these regions, the sequences encompassed in 5 RDs and 1 RI showed consistent variation, with a large deletion between the M5 and the 16 M genomes. RD4 and RD5 showed the large diversity among all *Brucella* genomes, both in RD length and RD copy number. Thus, RD4 and RD5 are potential sites for typing different *Brucella* strains. Other RD and RI regions exhibited multiple single nucleotide polymorphisms (SNPs). In addition, a genome fragment with a 56 kb rearrangement was determined to be consistent with previous studies. Comparative genomic analysis indicated that genomic island inversion in *Brucella* was widely present. With the genetic pattern common among all strains analyzed, these 2 RDs, 1 RI, and one inversion region are potential sites for detection of genomic differences. Several SNPs of important virulence-related genes (*motB*, *dhbC*, *sfuB*, *dsbAB, aidA, aroC*, and *lysR)* were also detected, and may be used to determine the mechanism of virulence attenuation. Collectively, this study reveals that comparative analysis between wild-type and vaccine strains can provide resources for the study of virulence and microevolution of *Brucella*.

## Introduction

Brucellosis is one of the most common bacterial zoonoses endemic in many countries, particularly those with developing livestock industries [1]. Among these, China is a large stock-raising country with approximately 300 million sheep [2]. Along with the increased number of animals, the incidence of human brucellosis has also increased rapidly over the last 10 years, which has negatively affected the public health and the livestock industry [3]. *B. melitensis* was the predominant strain associated with human brucellosis in China from 1950 to 2010 [2]–[4]. As a result, *Brucella* vaccination has become one of the most important measures for preventing and controlling brucellosis in animals, but different vaccine strains are used for different animals. For example, *B. abortus* B19 and *B. abortus* RB51 are used to vaccinate cattle [5]–[7] and *B. melitensis* Rev. 1 to vaccinate sheep and goats [8], [9].

In addition to these strains, the *B. melitensis* vaccine strain M5 was developed in 1962 by the Harbin Institute of Veterinary Medicine at the Academy of Agricultural Sciences. This vaccine was derived from the virulent strain of *B. melitensis* M28 and used mainly to vaccinate both pregnant and non-pregnant sheep and goats [2]. *B. melitensis* vaccine strain M5 exhibited superior protection against brucellosis in sheep and goats as demonstrated by the first large-scale use in 1970 [2]. However, the molecular and physiological mechanism causing the loss of virulence of this strain is not well understood. Species identification and subtyping of *Brucella* isolates are very important for epidemiologic surveillance, especially for differential diagnosis between infected and vaccinated animals. To address this problem, a series of methods have been reported. For example, Cloeckaert et al. discovered a unique mutation in the *rpsL* gene of Rev. 1 that eliminates an NciI restriction site; thus, this mutation can be detected by polymerase chain reaction–restriction fragment length polymorphism [10]. In addition, *B. abortus* strain-specific PCR assays were conducted to identify and distinguish specific *B. abortus* field strains from the vaccine strains S19 and RB51 [11]–[14]. Several other *B. melitensis* attenuated strains have also been sequenced and the data are available [15]–[17]. However, none of these studies addressed factors affecting virulence in the M5 vaccine strain. Thus, the primary aim of this study was to identify candidate ORFs associated with virulence in 16 M or lack of virulence in M5. Using bioinformatics, this study is the first to perform whole genome analysis directed toward identifying the sequence differences between the *B. melitensis* vaccine strain M5 and the virulent strain 16 M and reveals a mechanism of attenuation genes.

## Results and Discussion

### General features of the *B. melitensis* vaccine strain M5 genome

High-throughput bioinformatics revealed 26 genes present in the M5 genome. These genes were compared to those of the 16 M stain, and all were found to be homologues of the 16 M genome. These data suggest that the genomes of M5 and 16 M have a very similar genomic structure.

#### Genome assembly and annotation

The draft-quality genome of the vaccine strain M5 was sequenced and assembled into 95 contigs with N50 = 775,745 bp (Table S1). Scaffold 1, the largest scaffold, comprised most of chromosome II. Further analysis by PCR sequencing showed that this scaffold contained a complete 16S rRNA gene which was different from the copy in chromosome I. However, we did not continue to close the whole gap caused by duplication of the 16S-23S-5S rRNA operon. Compared to the sequenced genome of *B. melitensis* 16 M, the size, number of open reading frames (ORFs), guanine–cytosine (GC) content, gene length, and gene density were largely conserved in the genome of M5 (Table 1).

**Table 1:** Genomic features of the newly sequenced genome of the *B. melitensis* vaccine strain M5 compared with the known genomic sequences of the virulent strain 16 M.

Genome Feature	M5	16M
G+C content(%)	57.25	57.22
# of ORFs	3305	3198
% of ORFs in Genome	86.51	87.60
Average Length of ORFs	862	894
# of tRNA	49	54
# of rRNA operons	3	3

In the M5 genome, we identified 3305 ORFs that were larger than 300 bp (100aa), covering 86.5% of the genome, of which 80.9% matched the NCBI COG database with an e-value less than 1e-5 (Table S2). Many genes of these ORFs were located in common pathogenic pathways, such as *virB* and *bvrS/bvrR*. In addition, the previously reported genomic island containing a type IV secretion system [14] was also identified in the vaccine strain M5. Moreover, two additional GC islands with unknown functions were identified, and appeared to be possible transfer elements by a horizontal transfer with extremely low GC content. We also detected all 3 rRNA operons in the genome, which was consistent with the *B. melitensis* 16 M. However, 2 SNPs in the rRNA sequences within chromosome II of M5 were identified when compared to those of chromosome I.

### Genome region differences

#### Genomic arrangement

Although there was homology between the genes of M5 and 16 M, the genome layout of M5 suggests a ∼56 kb inversion or rearrangement in the head of scaffold 1, which mapped to chromosome II of 16 M. This region is flanked by two duplicate sequences of the insertion sequence (IS) IS1953. Two genes located upstream (BMEII0183/BMII0184) and downstream (BMEII0227/BMEII0228) of these inversion sequences were associated with transposes. In addition, inactivated derivatives were found in the IS elements. These findings suggest that the inversion was due to the duplication of insertion elements.

Aligning this 56 kb inversion region with other *Brucella* strains revealed that genomic inversions vary among available genomes including; *B. microti* CCM 4915, *B. canis* ATCC 23365, *B. suis* ATCC 23445, and *B. suis* 1330; similar to *B. ovis* ATCC 25840 and *B. melitensis* ATCC 23457; and missing in *B. abortus bv.1* str.9-941, *B. abortus* S19, and *B. Abortus* 2308 (Table 2). Functional analysis of this 56 kb inversion indicated that the region contains a large number of peptide ABC transporter genes (19 of 42 genes) such as spermidine–putrescine (*pot*), oligopeptide (*opp*), and dipeptide (*dpp*) systems. These ABC transporter genes play an important role the virulence of *Brucella* stains, and can influence trafficking of *Brucella* to compartments associated with endoplasmic reticulum where peptides and amino acids can be found in large numbers. However, absence of these transporters can be negative effects. For example, loss of the *opp* system in *B. ovis* may deregulate peptide uptake resulting in increased peptide levels and reduced intracellular survival [18].

**Table 2:** Inversion on the *B. melitensis* vaccine strain M5 chromosome II aligned to reference sequences.

Accession Number	Strain	Biovar	Inversion*
NC_003318	*Brucella melitensis* 16M	biovar 1	+
NC_013118	*Brucella microti* CCM 4915	−	+
NC_010104	Brucella canis ATCC 23365	−	+
NC_010167	*Brucella suis* ATCC 23445	biovar 2	+
NC_004311	Brucella suis 1330	biovar 1	+
NC_009504	*Brucella ovis* ATCC 25840	−	−
NC_012442	Brucella melitensis ATCC 23457	biovar 2	−
NC_006933	*Brucella abortus* 9–941	biovar 1	No hit
NC_010740	*Brucella abortus* S19	biovar1	No hit
NC_007624	*Brucella* a*bortus* 2308	biovar1	No hit

* “+”, inversion; “–”, the same direction; “No hit”, no alignment (but no deletion).

#### Genome deletions

This inversion region revealed a potential marker for distinguishing infected animals from animals vaccinated with M5. To explore this possibility, we designed primers and verified the results by testing various strains. Comparative genomic analysis identified 11 low read coverage regions and 2 high coverage regions of more than 300 bp each in the 16 M genome. Except for deletion, the other possible reason previous SOLEXA reads matching failed to identify these regions is that they were side effects of genomic rearrangement, where reads only match references with half-lengths. However, these characteristics may not produce a gap over 300 bp (100aa, 4 folds of reads length). Among these regions, we hypothesized that the low coverage regions in the 16 M genome comprised the majority of the genomic differences observed in the M5 genome. To test this, we examined with the assembled genome of M5 to verify whether these regions were missing. However, the large regions were not missing in the M5 draft genome, and only 11 large RDs were found: RD1 to RD11, 5 in chromosome I, and 6 in chromosome II (Table 3).

**Table 3:** Genes deleted from RDs of the *B. melitensis* vaccine strain M5.

Chromosome	Log2(CDM*)	RD #	RD Length	Gene name	% in RD	Annotation
I	>−2,<−1	RD1	337	BMEI0194	33.2	Putative cytoplasmic protein
		RD1	337	BMEI0195	2.07	ATPases with chaperone activity, ATP-binding subunit
		RD2	309	BMEI0934	14.55	Superfamily II DNA and RNA helicases
		RD3	906	BMEI1965	6.54	Translation initiation factor 2 (IF-2; GTPase)
	< = −2	RD1a**	461	BMEI0194	53.89	Putative cytoplasmic protein
		RD1a**	461	BMEI0195	0.89	ATPases with chaperone activity, ATP-binding subunit
		RD4	342	BMEI0407	39.24	Unknown function
		RD5	883	BMEI1661	74.97	Site-specific recombinases, DNA invertase Pin homologs
		RD5	883	BMEI1662	73.89	Hypothetical protein BMEI1662
II	>−2,<−1	RD6	306	BMEII0253	29.91	Murein endopeptidase
		RD7	330	BMEII0289	23.76	Asp-tRNAAsn/Glu-tRNAGln amidotransferase A subunit and related amidases
		RD8	719	BMEII0527	46.63	Exonuclease VII, large subunit
		RD9	335	BMEII0566	8.29	ABC-type Fe3+ transport system, permease component
		RD10	327	BMEII1039	19.26	16S rRNA uridine-516 pseudouridylate synthase and related pseudouridylate synthases
	< = **−**2	RD11	321	BMEII0124	23.46	NAD-dependent aldehyde dehyRDogenases
		RD11	321	BMEII0125	21.31	NAD-dependent aldehyde dehyRDogenases

* CDM:Coverage Divided by Mean coverage.

* RD1a: included in RD1.

**Figure 1:**
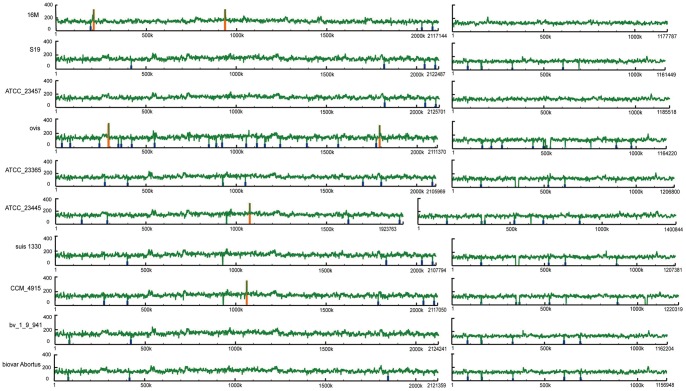
The differences between the whole genomes of M5, 16M, and other *Brucella* strains. Regions with higher and lower read coverage are identified by colored lines. Red: more than 4× means coverage, Orange: 2× to 4× means coverage, Blue: ¼ to ½× means coverage, and Aqua: less than ¼× means coverage. The window that is used to calculate the read coverage is 1000 bp with a 500 bp overlap (the figure will be indistinct with a smaller window); thus, only regions with lengths ≥ 1000 bp are displayed. This window is different from the method used to determine RDs.

The differences between the whole genomes of M5, 16 M, and other *Brucella* strains, are presented in Table 3 and Figure 1. Sixteen genes of 16 M genome were located in the RDs. The accurate genomic positions of these RDs were determined using the assembled M5 genome data. After the assembled M5 genome was compared with the 16 M genome, 5 of 11 RDs (RD1, RD2, RD4, RD5, and RD7) were confirmed to vary in the 16 M genome. The variation of these 5 RDs are described as follows:

RD1 included two ORFs, BMEI0194 and BMEI0195. BMEI0194 encodes a hypothetical cytosolic protein, whereas BMEI0195 encodes the ATP-dependent chaperone ClpB, which is essential for heat–shock response and belongs to the Class 1 family of Clp/Hsp100 AAA+ ATPases. It is involved in the recovery of the cell from heat-induced damage in cooperation with DnaK, DnaJ and GrpE.

RD2 contained 1 ORF, BMEI0934, encoding the ATP-dependent RNA helicase, which catalyzes the unwinding of double-stranded nucleic acids. Helicases are tightly integrated (or coupled) components of various macromolecular complexes involved in processes such as DNA replication, recombination, and nucleotide excision repair, as well as RNA transcription and splicing.

RD4 contained 1 ORF, BMEI0407, which encodes a hypothetical protein. A 59 bp repeat region was found in the flank of this region in the 16 M genome, and each of the two flanking repeats was duplicated on each side (Fig S1). This region is a candidate for multi-locus variable-number tandem-repeat analysis detection.

RD5 contained 2 ORFs, BMEI1661 and BMEI1662. BMEI1661 is part of an operon, whereas BMEI1661 belongs to the resolvase family (Fig S1). Notably, BMEI0902 in chromosome I of 16 M also belongs to the resolvase family. Located in a region with high read coverage, this gene is considered a duplicate in the M5 genome. Of the whole 231-bp sequence of BMEI0902, 180 bp was identical to BMEI1661. This observation suggests that the two genes are possible homologs, although BMEI1661 contained other sequences more than 500 bp that were not present in any other species. This kind of use bias in different site-specific recombinases may lead to various acquisition abilities among different DNA fragments.

RD7 contained 1 ORF, BMEII0259, which encodes the Asp-tRNAAsn/Glu-tRNAGln aminotransferase A subunit and related amidases. In this gene, a 21 bp fragment in M5 replaced a 6 bp fragment in 16 M. This mutation may minimally affect the protein function because a 3× nucleotide insertion in a gene does not cause a downstream frame shift. Thus, we considered this region a normal mutation causing the low read coverage.

The other 6 RDs may be caused by large numbers or continual SNPs in the core regions (Figure S1). These regions were compared to all available *Brucella* genomes. In addition to RD4 and RD5, all other RDs were specific in the vaccine strain M5 compared with the virulent strain 16 M. RD4 and RD5 showed large divergence among *Brucella*, suggesting that they are active mobile elements and can be targets for typing and detection of *Brucella*. RD4 is a tandem repeat with different lengths in each genome ranging from 58 bp to 342 bp. A similar method was investigated in all *Brucella* genomes. Figure 1 shows all the RDs compared with the M5 genome.

#### Unique genome regions

Chromosome I of M5 contained two regions with high read coverage, suggesting duplication. These regions were identified as RI1 and RI2. RI1 contained the ORF BMEI0200, which encodes a transposase insertion sequence in the transposon Tn4003. This transposon was also found in 16 M, where another copy was also found. RI2 contained two ORFs, BMEI0901 and BMEI0902. BMEI0901 encodes a resolvase similar to phage Mu DNA invertase Pin homologs, whereas BMEI0902 encodes a site-specific recombinase similar to an anti-repressor (*ant*) gene. This gene encodes a phage-related DNA binding protein and is homologous to BMEI1661 in RD5. Comparative genome analysis revealed that most available *Brucella* genomes contained only one copy of BMEI0901/BMEI0902. However, *B. canis* ATCC 23365, *B. suis* 1330, *B. melitensis* ATCC 23457, *B. abortus* bv.1 str.9–941, *B.abortus* S19, and *B. Abortus* 2308 as well as the M5 strain, have two copies of these ORFs in each genome. Unlike other genomes, we did not find BMEI0901/BMEI0902 homologues in the genome of *B. ovis* ATCC 25840. Thus, this site-specific recombinase widely exists in almost every *Brucella* genome, suggesting that it originates from a *Brucella* ancestor. Interestingly, the recombinase appears to the same family to RD5, suggesting that it complements the absent genes in that region. Similar to RI2, RD5 exhibits different patterns of these genes in different genomes (Table 4).

**Table 4:** Site-specific recombinase genes on *Brucella* strains.

Accession Number	Strain	Biovar	Site specific recombinases genes	
			BMEI0901/0902	BMEI1661/1662
AONT00000000.1	*Brucella melitensis* M5	biovar1	+	−
NC_003317	*Brucella melitensis* 16M	biovar1	+	+
NC_013119	*Brucella microti* CCM 4915	−	+	+,2*
NC_010103	*Brucella canis* ATCC 23365	−	+,2	−
NC_010169	*Brucella suis* ATCC 23445	biovar2	+	+
NC_004310	*Brucella suis* 1330	biovar1	+,2	−
NC_009505	*Brucella ovis* ATCC 25840	−	−	+
NC_012441	*Brucella melitensis* ATCC 23457	biovar2	+,2	−
NC_006932	*Brucella abortus* 9–941	biovar1	+,2	−
NC_010742	*Brucella abortus* S19	biovar1	+,2	−
NC_007618	*Brucella abortus* 2308	biovar1	+,2	−

#### Genome SNPs

Beyond the 11 RDs, M5 and 16 M exhibit considerable general genetic homology. Pairwise analysis demonstrated that there were 2819 SNPs between the genomes of 16 M and M5. Among these SNPs, 585 were in the intergenic regions and 2234 were in the genetic regions. Although the genome divergence time can be calculated by non-synonymous intra-species mutations, the splice time of these two genomes is difficult to estimate because the genome is under manual selection pressure. The 2234 SNPs in the genetic regions included 798 synonymous SNPs and 1436 non-synonymous SNPs (Table S3). The non-synonymous SNPs only resulted in amino acid changes, with no frame shift or nonsense mutations.

**Figure 2:**
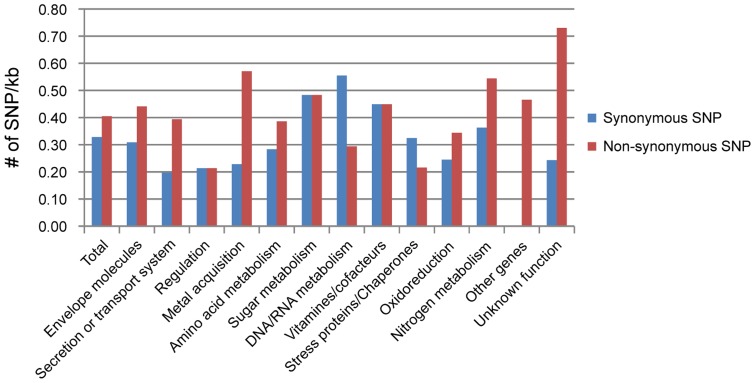
Synonymous and non-synonymous SNPs rates among the virulence-associated genes. The x-axis represents the functional groups of virulence-associated genes, and the y-axis represents the SNPs in 1 kbs. The blue and red bars represent synonymous and nonsynonymous SNPs, respectively.

Delrue et al. presented a list of 196 unique virulence-related genes divided into 12 functional classes [19]. Our comparative analysis of these two genomes suggests that SNPs widely exist in these genes. A total of 183 SNPs were present in 197 genes, among which 101 SNPs were non-synonymous mutations. Compared with SNPs occurring at other sites of the genome, these genes presented higher positive selection pressure, with Ka/Ks of 1.23. Detailed function analysis of these virulence-related genes revealed that most functional categories were involved in fast evolution (Figure 2 and Table 5). Within all known functional groups, both total SNP rates and non-synonymous SNP rates were higher in the genes of “metal acquisition” and “nitrogen metabolism,” and both Ka/Ks values were greater than 1. The Ka/Ks values were also large in classical virulence factors, including genes of envelope molecules and secretion or transport system. These data suggest that mutations in these genes can play important roles in virulence attenuation of the vaccine strain. However, the SNP rates and Ka/Ks were also high in the groups of unknown function and other genes, suggesting the need for further investigation into the functions of these genes.

**Table 5:** Distribution of SNPs in virulence-related genes.

Functional group	Gene		Non-synonymous SNP				Synonymous SNP				All SNP				Ka/Ks
	#	%	#	%	#/gene	#/kb	#	%	#/gene	#/kb	#	%	#/gene	#/kb	
Envelope molecules	19	9.64	10	9.90	0.53	0.44	7	8.54	0.37	0.31	17	9.29	0.89	0.75	1.43
Secretion or transport system	26	13.20	10	9.90	0.38	0.39	5	6.10	0.19	0.20	15	8.20	0.58	0.59	2.00
Regulation	18	9.14	5	4.95	0.28	0.21	5	6.10	0.28	0.21	10	5.46	0.56	0.43	1.00
Metal acquisition	6	3.05	5	4.95	0.83	0.57	2	2.44	0.33	0.23	7	3.83	1.17	0.80	2.50
Amino acid metabolism	25	12.69	15	14.85	0.60	0.39	11	13.41	0.44	0.28	26	14.21	1.04	0.67	1.36
Sugar metabolism	24	12.18	18	17.82	0.75	0.48	18	21.95	0.75	0.48	36	19.67	1.50	0.97	1.00
DNA/RNA metabolism	26	13.20	9	8.91	0.35	0.29	17	20.73	0.65	0.55	26	14.21	1.00	0.85	0.53
Vitamines/cofacteurs	5	2.54	3	2.97	0.60	0.45	3	3.66	0.60	0.45	6	3.28	1.20	0.90	1.00
Stress proteins/Chaperones	6	3.05	2	1.98	0.33	0.22	3	3.66	0.50	0.32	5	2.73	0.83	0.54	0.67
Oxidoreduction	16	8.12	7	6.93	0.44	0.34	5	6.10	0.31	0.25	12	6.56	0.75	0.59	1.40
Nitrogen metabolism	3	1.52	3	2.97	1.00	0.54	2	2.44	0.67	0.36	5	2.73	1.67	0.91	1.50
Other genes	3	1.52	2	1.98	0.67	0.47	0	0.00	0.00	0.00	2	1.09	0.67	0.47	-
Unknown function	20	10.15	12	11.88	0.60	0.73	4	4.88	0.20	0.24	16	8.74	0.80	0.97	3.00
Total	197	100.00	101	100.00	0.51	0.40	82	100.00	0.42	0.33	183	100.00	0.93	0.73	1.23

#### Virulence-associated metabolism and virulence factors

Comprehensive analysis of the 16 M and M5 genomes revealed variations in genome deletion, insertion, and SNPs. Given these genomic differences, we next sought to identify candidate ORFs associated with virulence in 16 M or lack of virulence in M5. To improve the accuracy of our analysis, we first performed an independent literature search to identify published differences between virulence factors and important metabolic pathways. Following this, large-scale screens and testing in the model systems were performed in *Brucella* to confirm and/or identify virulence factors associated with metabolism and virulence. The mutations of each virulence-associated gene between M5 and 16 M are listed in Table S4.

#### Envelope molecule

The LPS O-polysaccharide (O-PS) is the first molecule clearly involved in *Brucella* intracellular entry [20], [21]. Genetic studies demonstrated that the O-PS is synthesized by the so-called ABC transporter-dependent pathway, which characterizes homopolymetric polysaccharides. In *Brucella*, the genes involved in the synthesis of the O-PS and its translocation to the periplasm are clustered in the Wbk region of chromosome I (ORF BMEI1393 to BMEI1427) [22]. In our analysis of M5, no non-synonymous mutations were found in the core genes of this region (*wbpZ*, *wbkA*, *rfbD*, and others); however, several mutations were present in the ORFs BMEI1399 [G238C(A80P)] and BMEI1400 [C61T (P21S)], which are transposases in the cluster. In addition, other mutations involved in the synthesis of LPS were identified and included *pmm* [T397C(F133L)], *pgm* [A752G(H251R)], *wbdA* [T347C(L116S), G1103C(R368P)], *lpsA* [C278T(S93L)], *mgtA* [C142T(P48S), G380A(R127K)], and *amiC* [A545T(N182I)]. These data suggest that the reduced virulence of M5 is due, in part, to defects in O-PS synthesis.

#### Secretion or transport system

In addition to envelope molecules, the type IV secretion system (T4SS) of *Brucella* encoded by the VirB operon is a major virulence factor [23]. This operon is composed of 12 ORFs clustered in chromosome II (BMEII0025 to BMEII0036). We identified three ORFs associated with the M5 T4SS that contained single or two sites of non-synonymous mutations. These included: A463C (K155Q) in inner membrane ATPases (*virB4*, BMEII0028), A622G (T208A) and T1007A (F336Y) in the channel protein (*virB6*, BMEII0030), and C700G (R234G) in the channel protein (*virB9*, BMEII0033). The *virB* mutants cannot reach the replicative niche and reside in a membrane-bound vacuole [24]. In addition to mutations in the T4SS mutations C613G(P205A) in the ABC transporter (BMEII0923), C188T(A63V) and C659T(T220I) in the flagellar gene BMEII0154, and A427G(I143V) in the flagellar gene BMEII0159 were also identified. Recent studies showed that some proteins involved in the export of flagellar components are similar to the components of the type III secretion machinery, and flagellar apparatus itself can secrete virulence factors [25]. These data suggest that defects in virulence factor secretion may affect the replicative ability of M5.

#### Regulation

*Brucella* must survive under varying conditions ranging from the open environment to the intracellular medium. Thus, the bacterium must coordinate an intricate network of factors to generate a suitable adaptive response to various signals. The adaptive response is mediated by two-component regulatory systems (TCSs) and transcriptional regulators (TRs) [26]. To date, BvrS/R is the best characterized *Brucella* TCS [27]. Moreover, BvrS/R regulates several critical cellular functions, including cell division, chemotaxis, metabolism, and expression of toxins and other proteins important for pathogenesis. TRs important for *Brucella* survival are the GntR and LysR families. In addition, LysR-type transcriptional regulators (LTTRs) are widely distributed in the diverse genera of prokaryotes [28]. Many virulence factors of *Brucella* are regulated by LTTRs [29], [30]. Although no mutations were observed in the common TCSs or TRs, we did identify a non-synonymous mutation of T176C(L59P) and a 3 bp deletion from 391 to 393 at the M5 locus encoding LTTR (BMEI0116 in 16 M). These data suggest that M5 may have reduced ability to regulate survival in certain environmental conditions.

#### Metal acquisition

Iron acquisition and transport systems are essential for intracellular survival ability. For instance, *Brucella* with a mutation in the Fe^3+^ uptake gene *dhbC*, which affects DBHA siderophore synthesize, exhibits a high degree of attenuation [31]. In *B. abortus*, DHBA is important for bacterial growth on erythritol, explaining its potential role in cattle abortion [32], [33]. Detailed comparison between the *in vitro* and *in vivo* phenotypes of *B. melitensis dhb* mutants and strains with defined mutations in the erythritol catabolic genes is necessary to more accurately define the relationship among DHBA production, erythritol catabolism, and virulence in the natural ruminant host. The role of the TonB transport system is well documented in *Salmonella entrica* [34] and S*higella dysenteriae* [35]. The *tonB* mutants in these bacteria exhibit reduced growth in host cells, and the TonB complex is required for optimal uptake of DHBA siderophore and/or citrate in *B. melitensis* [36]. However, few studies have evaluated the expression of iron-related genes in *Brucella* spp. [31], [32]. The comparison between the transcript levels of *B. melitensis* 16 M and *B. melitensis* M5 during macrophage infection led us to presume that some common and differentially expressed transcripts of iron acquisition genes could be identified. In addition, previous proteomics studies of the vaccine strain M5 showed altered expression of proteins associated with iron utilization (data unpublished). To verify this hypothesis, we next focused on analyzing differences metabolic activity between M5 and 16 M.

#### Amino acid metabolism

Among the 19 synthesis subclass genes of these two strains, 10 genes of M5 contained point mutations. These data suggest that M5 may have some amino acid metabolism defects; yet failure of M5 to resist a sudden lack of nutrients requires further investigation.

#### Sugar metabolism

Maltose, ribose, arabinose, galactose, glucose, glycerol, erythriol, and inositol degradation pathways are essential for the intracellular survival of *Brucella* [37]. For example, mutants of genes encoding sugar transport systems, such as pyruvate carboxylase (BMEI0266), ribokinase (BMEII0089), 6-phosphogluconate dehydrogenase (BMEII1124), inosamine methylase (BMEI1301), glycosyl transferase (BMEII1101), ribose transport (BMEII0300), and glucose/galactose transport (BMEII1053) limit carbon or energy supply and affect gene regulation. In addition, the production of cyclic β-1,2 glucan (BMEI1837) is necessary for the intracellular replication of *Brucella* [38]. Upon examination of these pathways, SNP mutations in the ORF BMEI1837 of M5 caused five residue mutations at the positions 3587 to 3594, 3631 to 3638, and 3774 to 3782, and another non-synonymous mutation at position 4996. These data suggest that M5 may have defects in cyclic β-1,2 production.

#### DNA/RNA

Purine/pyrimidine synthesis, repair, and regulatory genes are essential for the intracellular replication of *Brucella*. A study by Crawford RM indicated that deletion of the *purE* gene from *B. melitensis* dramatically reduced bacterial virulence and suggested that this gene should be further evaluated as a potential vaccine [39]. Other genes under the same classification, such as *xseA*, *purL*, *purD*, *mgpS*, *tldD*, and *cobB* mutants, appear to be targets for attenuation vaccine strains.

#### Stress proteins/Chaperones

Genes encoding stress proteins have been an obvious choice for directed mutagenesis for virulence studies and vaccine generation. Within the M5 genome, genes encoding chaperones (BMEI2002 and BMEI1513) showed one non-synonymous mutation, namely, A37C(M13L) and G398A(G133D), respectively.

#### Oxidoreduction and nitrogen metabolism

DsbA has been shown to be involved in toxin production, adhesion, motility, extracellular enzyme production, and type III secretion system [40]. Few studies have investigated the role of DsbB in pathogenicity [41]. A G120A(M40I) mutation was detected in the *DsbB* (BMEI0384) gene of M5. In addition, published genome analysis indicated that *B. melitensis* can adapt to either aerobic or anaerobic environments. Among the genes involved in the process, we detected non-synonymous mutations in *FdhA*, *glnA*, *nifS*, and *glnD* of M5.

#### Unknown function

Seven ORFs encoding hypothetical protein were slightly different between the attenuated M5 strain and the virulent strain 16 M. Among these ORFs, BMEII0128 was identified as a virulence-associated ORF in another attenuated vaccine strain S19. Mutations of A31C(M11L), C589T(H197S), and A590C(H197S) were also identified in the vaccine strain M5.

## Conclusions

The vaccine strain M5 has been widely used in Asia for decades to vaccinate sheep against brucellosis [2]. While extensive studies have been performed on the phenotype and physiology of this strain, the molecular mechanisms responsible for loss of virulence are unclear. In this study, we determined the whole-genome sequence of M5 and conducted a comprehensive comparative analysis against the whole-genome sequence of the virulent strain 16 M. This comparative analysis revealed that the genome sequences were highly conserved with more than 99% identity, which agreed with previous studies. However, we identified 11 RDs and 2 RIs by the reads-to-genome comparisons. The gene and genomic structure analysis of RDs and RIs revealed that only 5 RDs and 1 RI showed a consistent variation with a large deletion in the M5 genome compared to 16 M. The other RDs and RIs displayed multiple SNPs, which caused the low read coverage. In addition, a genome fragment with a 56 kb rearrangement was determined to be consistent with the previous study. Based on these results, we suggest the use of genome sequence differences to create a precise map for effective vaccine detection and diagnosis. RD4 and RD5 indicated a large diversity among all *Brucella* genomes (Table S5) not only in RD length but in copy number as well. Thus, they are strong candidate sites for typing different *Brucella* strains.

RD4 is a typical random repeat region consisting of the sequence “GGGGATCAAGAGATCGCCCCCGTTTCCGTCGCCGTTCCCGTGCTGCCGAAGGCACACAAAGAGAAGAAACATGGCGGCGACGGCAATTGTTGAAAGCGCCATCATTTTTCGTGGGTGACGATGAC,” which repeats 1 to 2 times in each genome and can be considered the candidate VNTR loci for MLVA genotyping. RD5 is 883 bp and overlaps with two resolvase family genes in 16 M. The ORFs BMEI1661 and BMEI0902 belong to this family, which are 231 and 747 bp respectively. The proteins encoded by these ORFs have the same core functions but different protein and structure combinations. Comparative genome analysis in *Brucella* revealed two typical sequence types present in this region. The first sequence was approximately 139 bp and contained a gene similar to BMEI0902. The second was 883 bp and contained a gene similar to BMEI1661. In our study, RI2 belongs to the first sequence. Compared with the 16 M genome, the M5 strain had an additional RI2 region but the RD5 was missing. Further analysis in all *Brucella* strains presented different patterns of these two regions in the various genomes. This bias of the selected site-specific recombinase has been shown to be involved in the acquisition of drug resistance genes and alteration of gene expression [42], suggesting that this unique gene affects virulence.

Considering the genetic patterns widely present in all strains, we consider the 2 RDs, 1 RI, and the inversion region as candidates for detection of genome differences. Our analysis was also aimed at identifying 1) the genes or genome fragments that only existed in our genome,2) the mutations specific toward the virulent strain and the attenuated strain, and 3) the molecular mechanisms of M5 strain attenuation . Among the 11RDs identified, RD1, RD2, and RD7 were in M5, whereas they are consistent with other strains in 16 M. Furthermore, RD3, RD6, RD8, RD9, RD10, and RD11 were highly differentiated regions with a large number of mutations. All of these regions are possible candidates for distinguishing M5 from 16 M. Notably, RD9 includes an ORF encoding an ABC-type Fe^3+^ transport system (*sfuB*, BMEII0566).

In *Brucella*, SfuA binds free Fe ^3+^ in the periplasm, and SfuB and C transport this cation across the cytoplasmic membrane. Several studies suggested that the ferric dicitrate complex itself represents important substrates for the SfuABC type transporters [32], [33]. On the basis of the potential relevance of ferric dicitrate as an iron source for *Brucella* in vitro, the *sfuB* mutation seemingly is responsible for the loss of virulence in vaccine strain M5. Surveys of the genome sequences of *B. melitensis* 16 M and *B. suis* 1330 suggest that transport systems that allow the *Brucella* to utilize heme and ferric dicitrate as iron sources are important for sustaining the intracellular lifestyle of the *Brucella* in host macrophages [43].

The whole-genome sequences analyzed in this study clearly revealed large genome differences between 16 M and M5. Many of these differences were in genes associated with *Brucella* metabolism and survival, and thus serve as potential detection sites for distinguishing between the virulent 16 M and attenuated M5 strains. In addition, our analysis revealed two novel sites for typing *Brucella*. Furthermore, 3 RDs and SNPs of important virulence-related genes such as *motB*, *dhbC*, *sfuB*, *dsbAB*, *aidA*, *aroC*, and *lysR*, were considered to be sites causing virulence loss in the M5 strain. Collectively, this study reveals that comparative analysis between the wild-type and vaccine strains can provide resources for the study of *Brucella* virulence and *Brucella* microevolution. Further detailed analysis of the gene function of deletion genes can elucidate their roles in virulence attenuation of *B. melitensis.*

## Materials and Methods

### Isolation extraction and identification

*B. melitensis* M5 was obtained from the National Institute for Communicable Disease Control and Prevention Center. This strain was submitted for classical identification procedures as follows: CO_2_ requirement, H_2_S production, and inhibition of growth by basic fuchsin and thionin, agglutination with monospecific antisera, and phage typing. Total genomic DNA was extracted using the DNeasy Blood and Tissue Kit (QIAGEN, China Ltd., China). An aliquot of the DNA was subjected for analysis using the Bioanalyzer (Agilent Technologies) and was confirmed for absence of DNA degradation.

### Genome sequencing, assembly, and annotation

A genome shotgun method was used to collect the genome sequence. We constructed two DNA libraries: 500 bp and 3 kb∼5 kb DNA fragments for high-throughput genome sequencing with SOLEXA. Pair-end 75-bp reads were collected. We obtained 518 Mb and 7,000,000 reads covering ∼157 folds for the 16 M genome. The draft genome sequence of *B. melitensis* vaccine strain M5 is available in GenBank under accession number AONT00000000.1.

The SOLEXA data were assembled using the SOAP *de-novo* software. Genome sequences were analyzed using an automatic genome analysis pipeline. Gene prediction was conducted following the method used in the study by Glimmer [44] with default parameters. Gene annotation was performed with BLAST compared with nt, nr, and SwissProt databases with an e-value of 1e-5. Gene function prediction was conducted using COG and InterproScan. Gene pathways were annotated based on Kyoto Encyclopedia of Genes and Genomes.

### Comparative genomic analyses

High-throughput SOLEXA reads mapping to 16 M was used in the whole-genome comparison because that the draft genome had limits in gene prediction and alignment. Genome differences were valued by each site of SOLEXA coverage, which is standardized by calculating the CDM (read coverage of each site/mean coverage of the whole genome). The log2 value of the CDM (Log2CDM) was used to improve the description.

We compared the sequences of two strains by MUMmer [45] and removed unclear fragments by BLAST. The IS elements were identified and compared based on the IS database (http://www-is.biotoul.fr/). Genes lost or gained were selected by PERL scripts, and their functions were determined. A synteny map between the two strains was constructed by the OrthoMCL pipeline [46]. Whole-genome raw SNPs were detected following the method published by Mummer et al. with default parameters. The detailed variations in virulence-associated genes between M5 and 16 M, including SNPs, deletion, and insertion, were further identified by alignment using CLUSTALW and then calculated by PERL scripts. A Ka/Ks calculator was used [47].

## Supporting Information

Figure S1 **Alignment of RDs in the** ***B. melitensis*** **vaccine strain M5 to the virulent strain 16** **M.** RD1, RD2, RD4, RD5, and RD7 exhibit obvious genome insertion and deletion in these regions. The insertion and deletion caused low read coverage regions and are denoted as RDs. . Sequences of other RDs were identical with the virulent strain 16 M. Low coverage in these regions may be caused by uncertainty in sequencing.
(PDF)

Table S1 **Genome assembly of the** ***B. melitensis*** **vaccine strain M5.**
(PDF)

Table S2 **COG-based function of ORFs identified in the** ***B. melitensis*** **vaccine strain M5.**
(PDF)

Table S3 **SNPs detected in the** ***B. melitensis*** **vaccine strain M5 compared with the virulent strain 16** **M.**
(PDF)

Table S4 **Distribution of SNPs on virulence-related genes.**
(PDF)

Table S5 **RDs aligned to reference sequences.**
(PDF)
