# Supplementary figures and images for: Comparative Genomic Analysis of Brucella melitensis Vaccine Strain M5 Provides Insights into Virulence Attenuation
Source: PLoS One. 2013 Aug 14;8(8):e70852. doi: 10.1371/journal.pone.0070852 (PMC3743847; doi:10.1371/journal.pone.0070852)

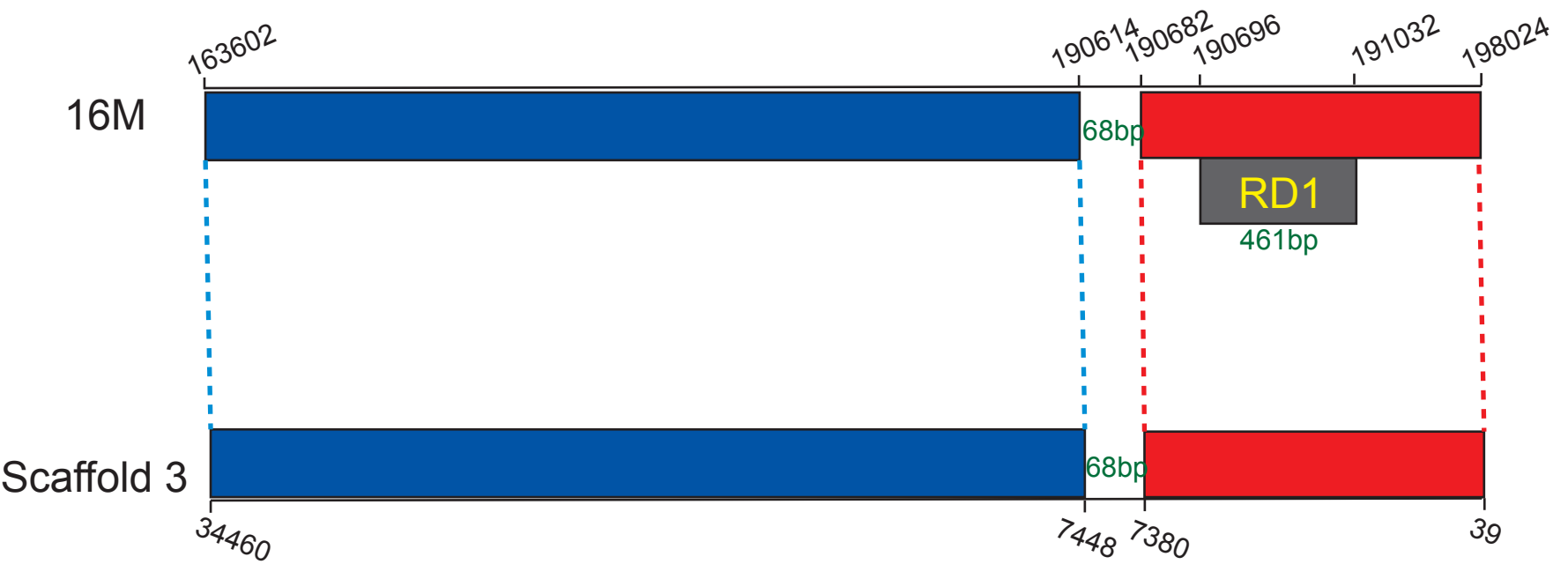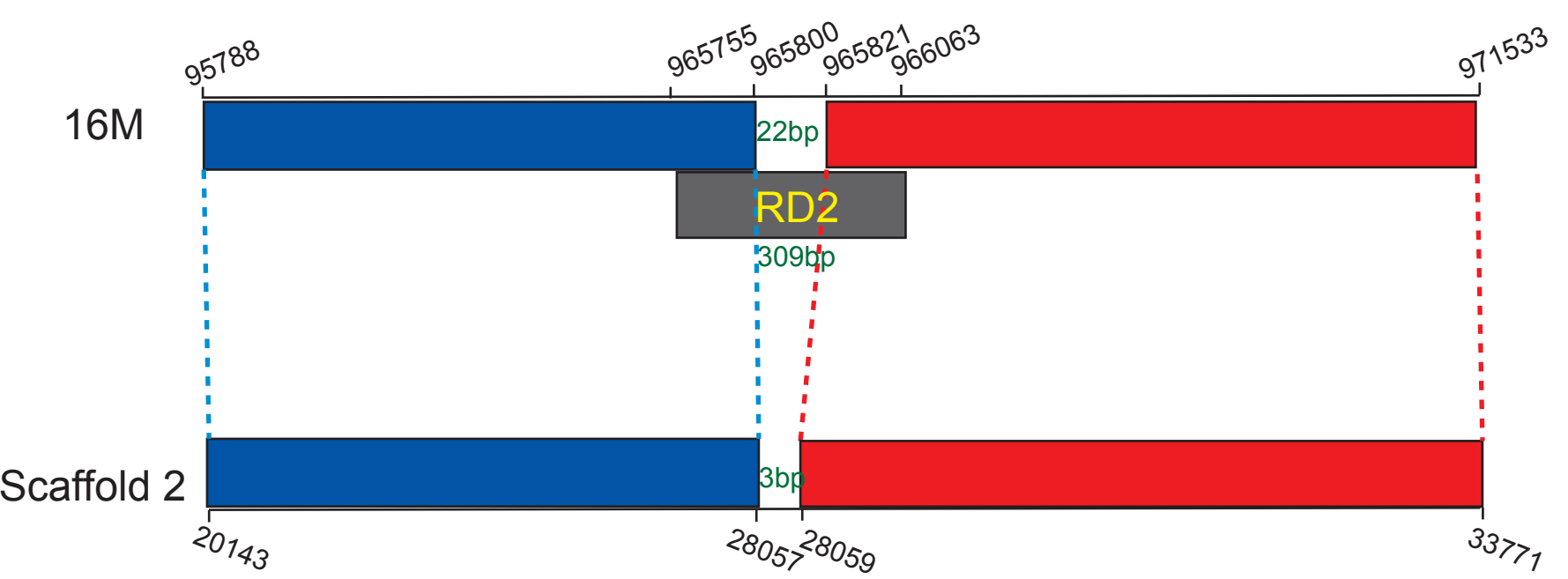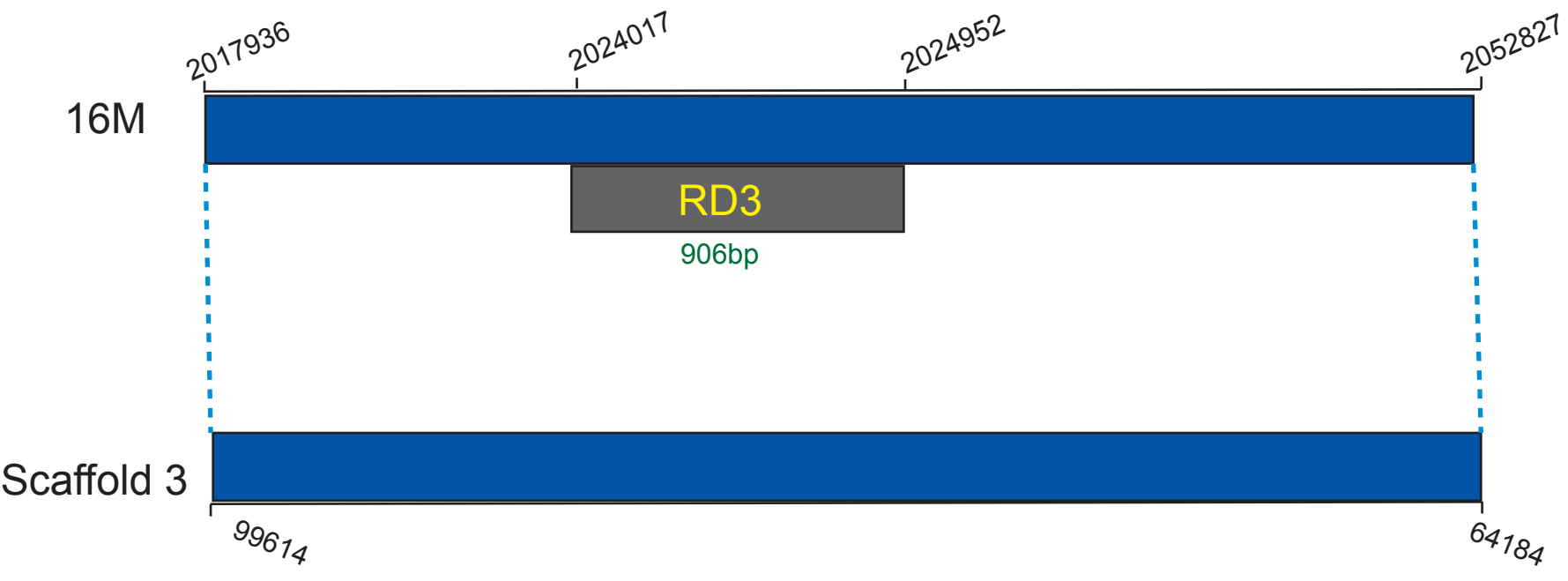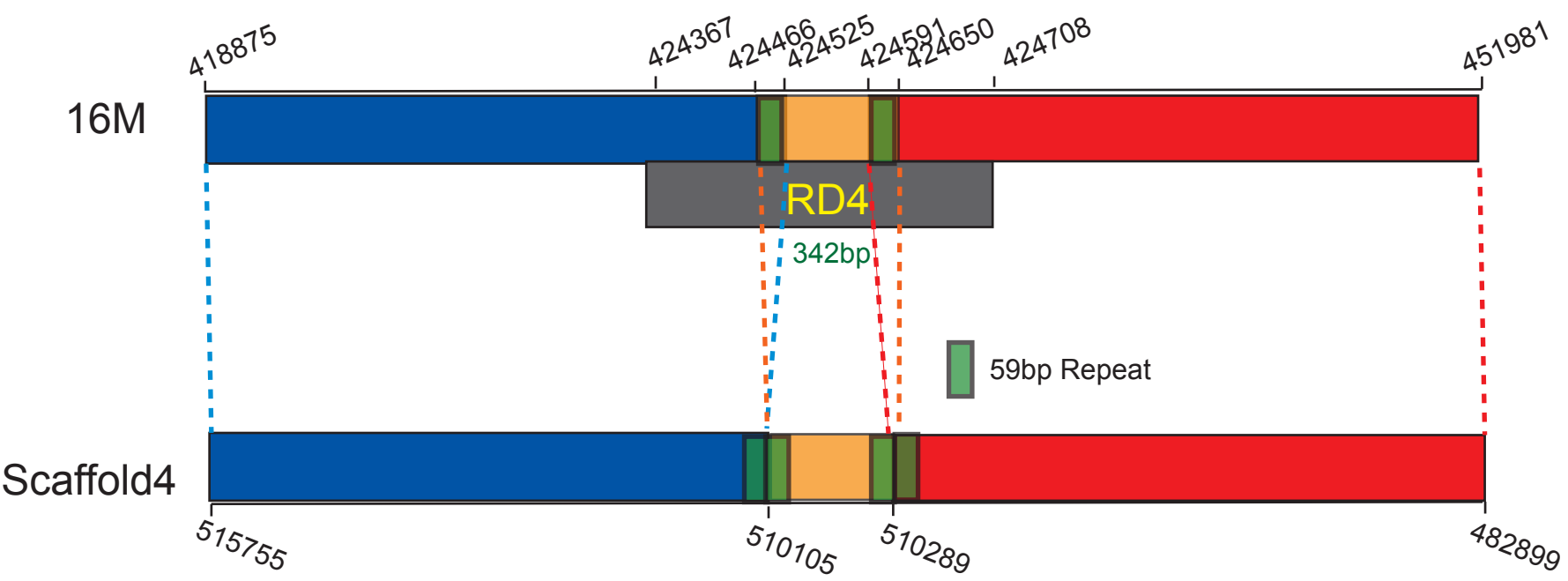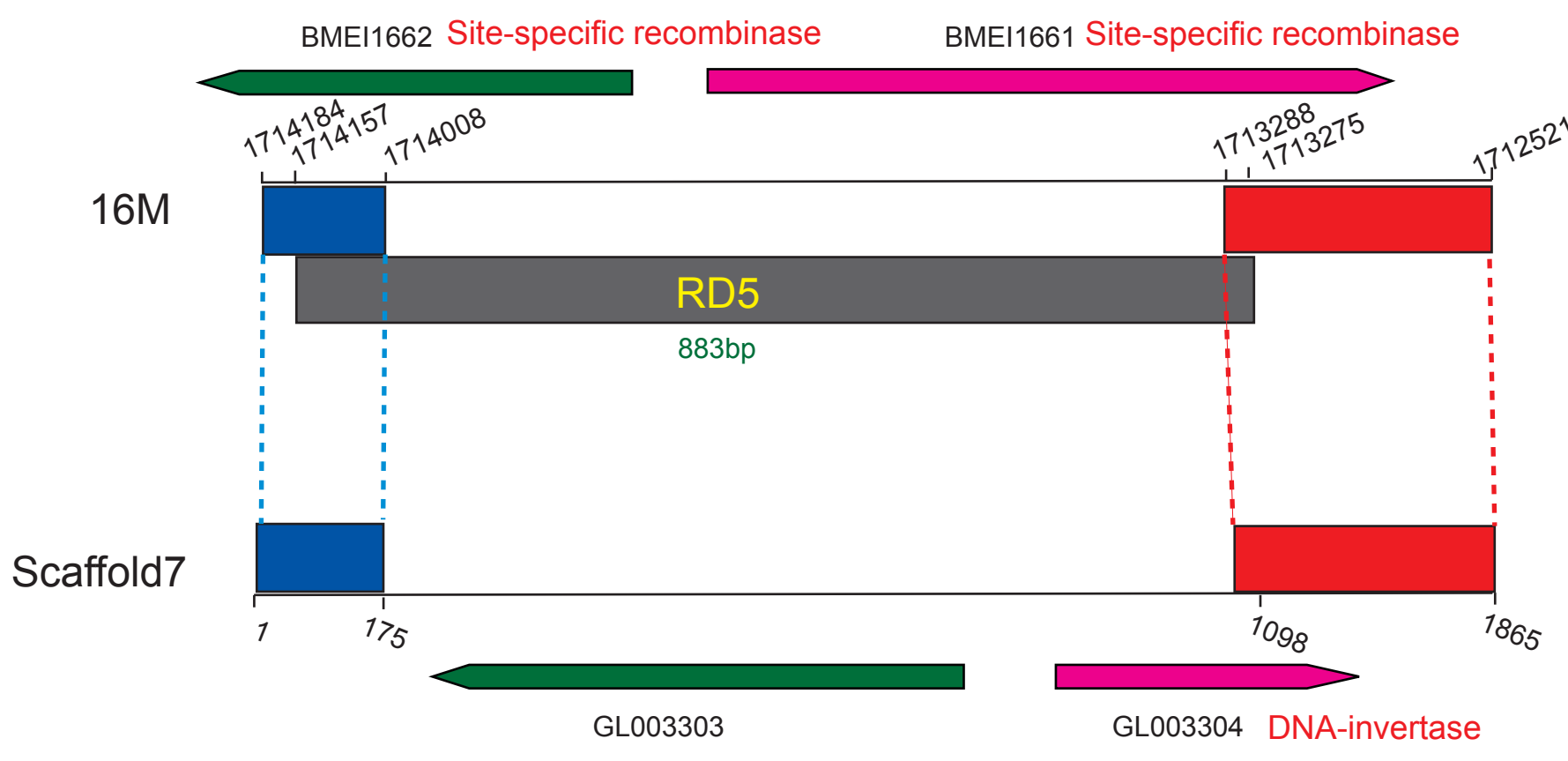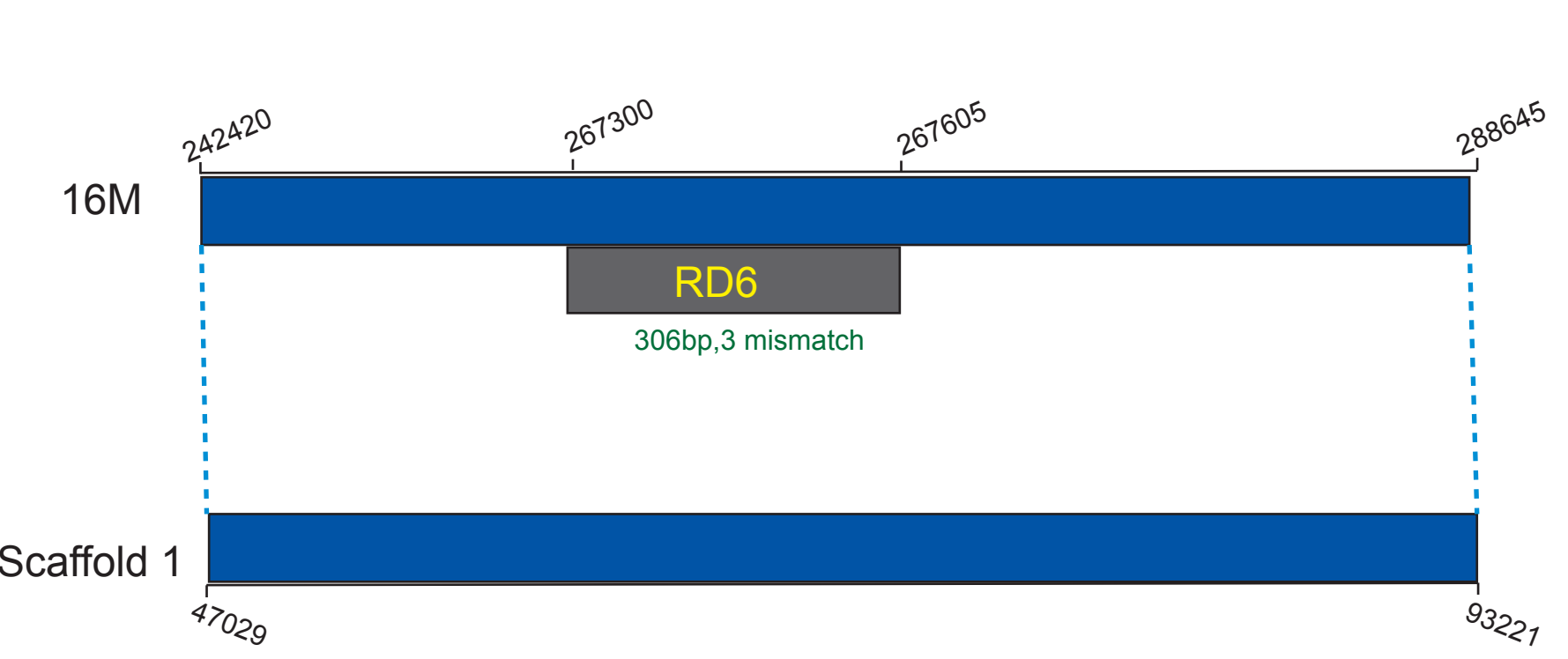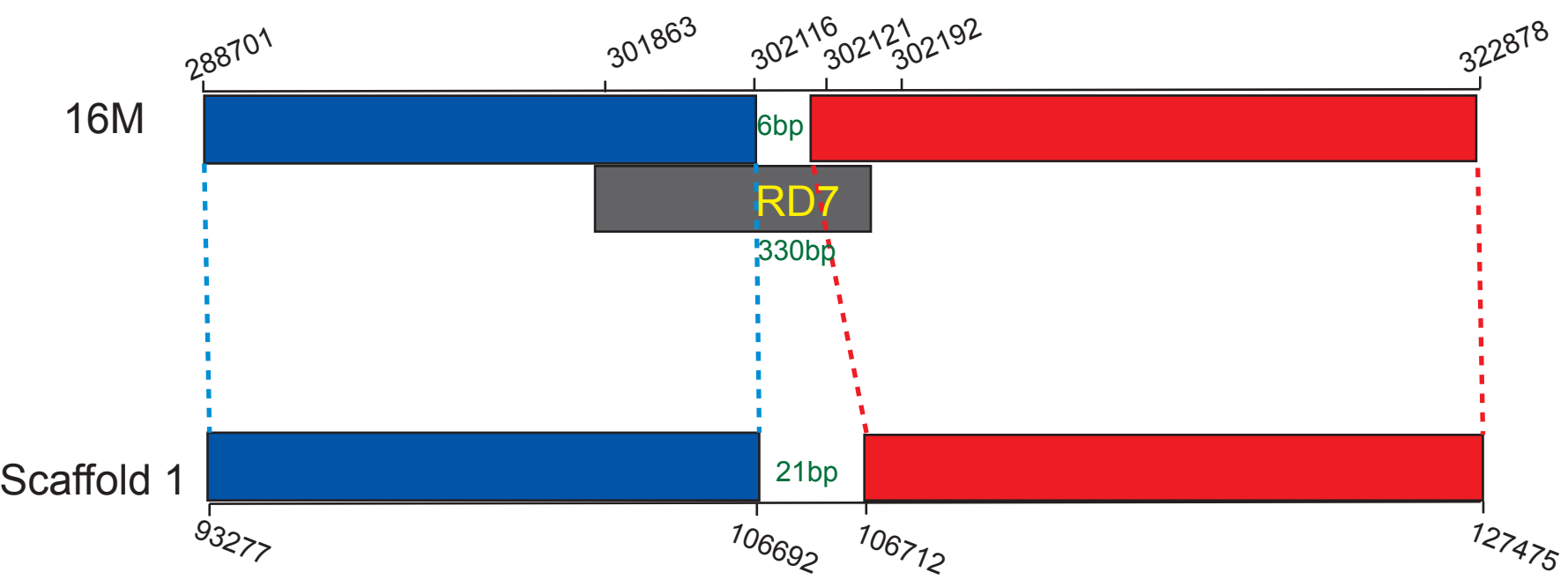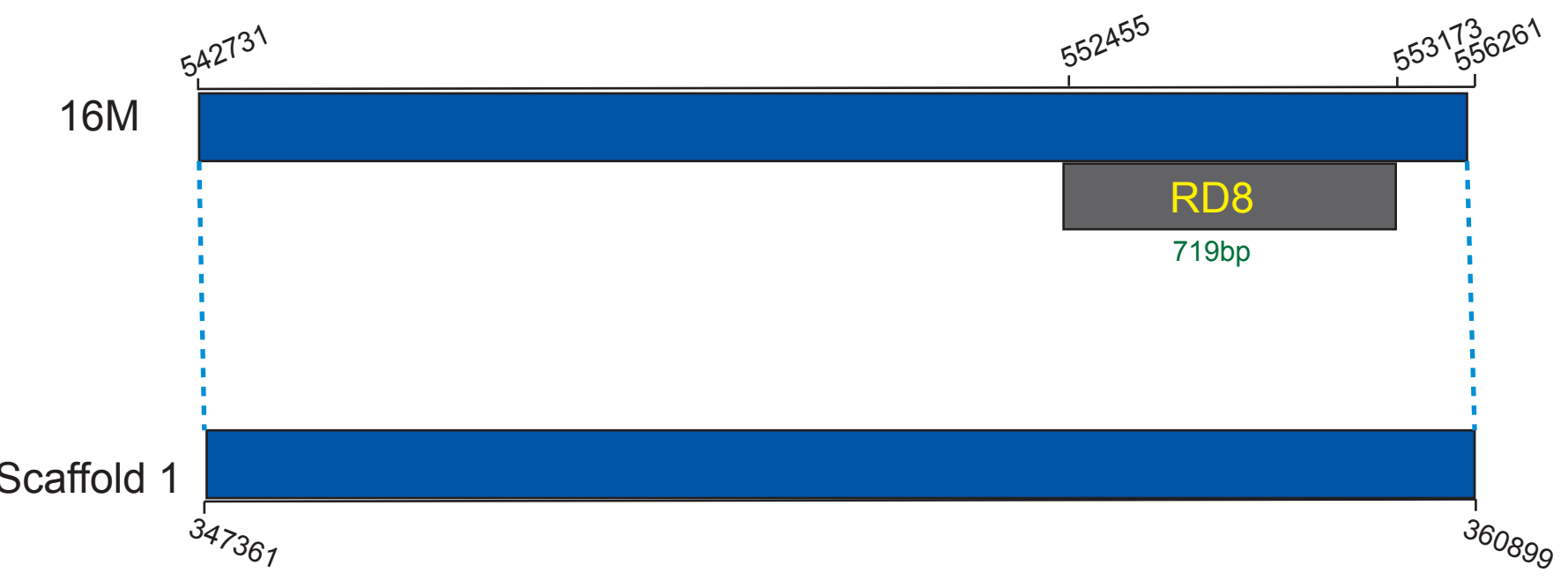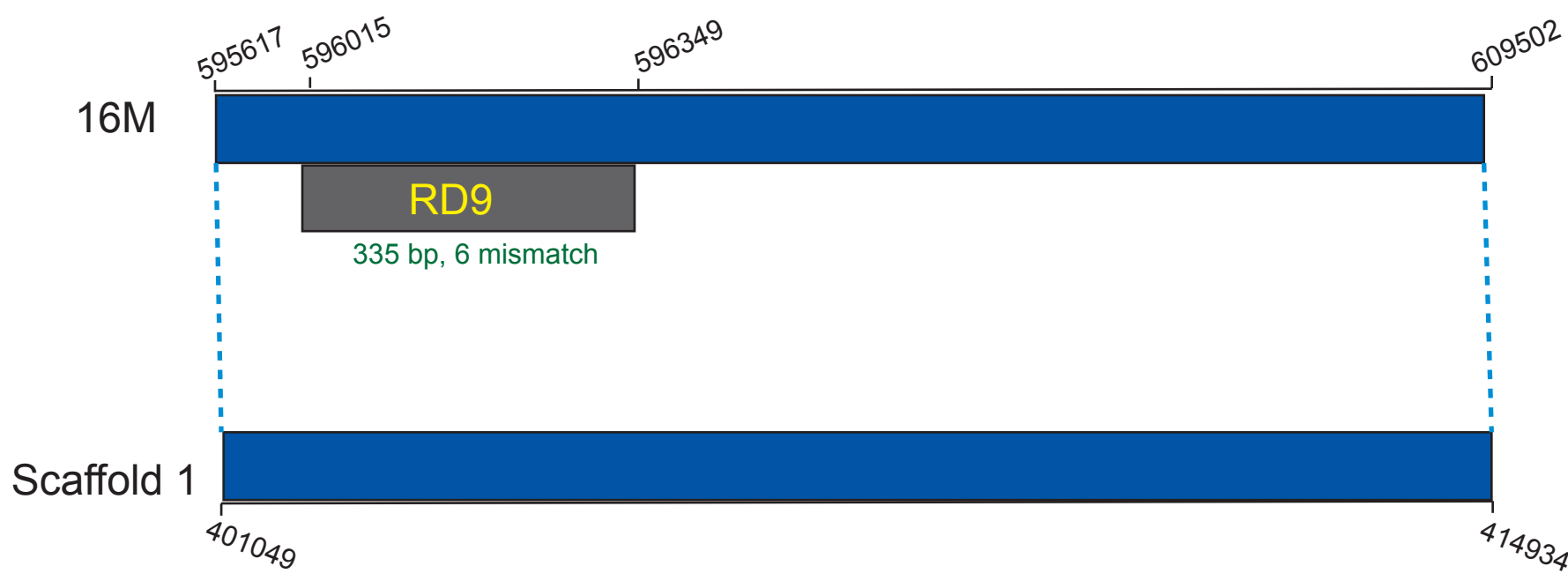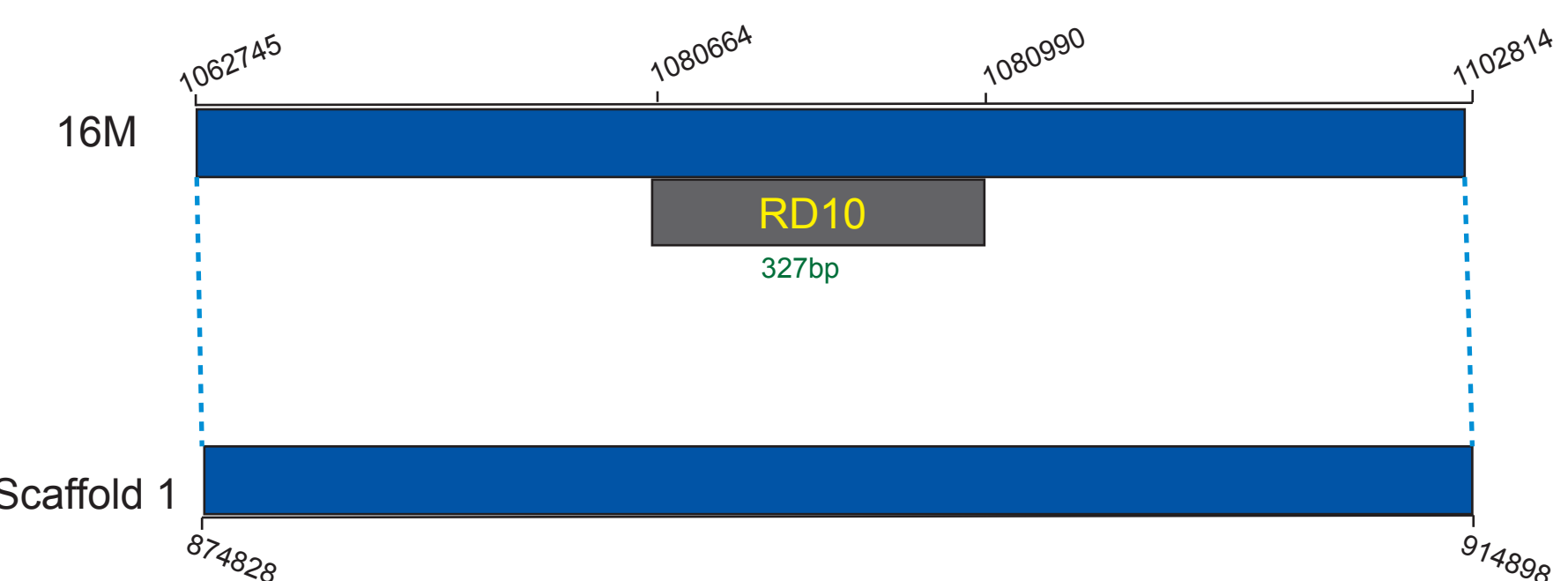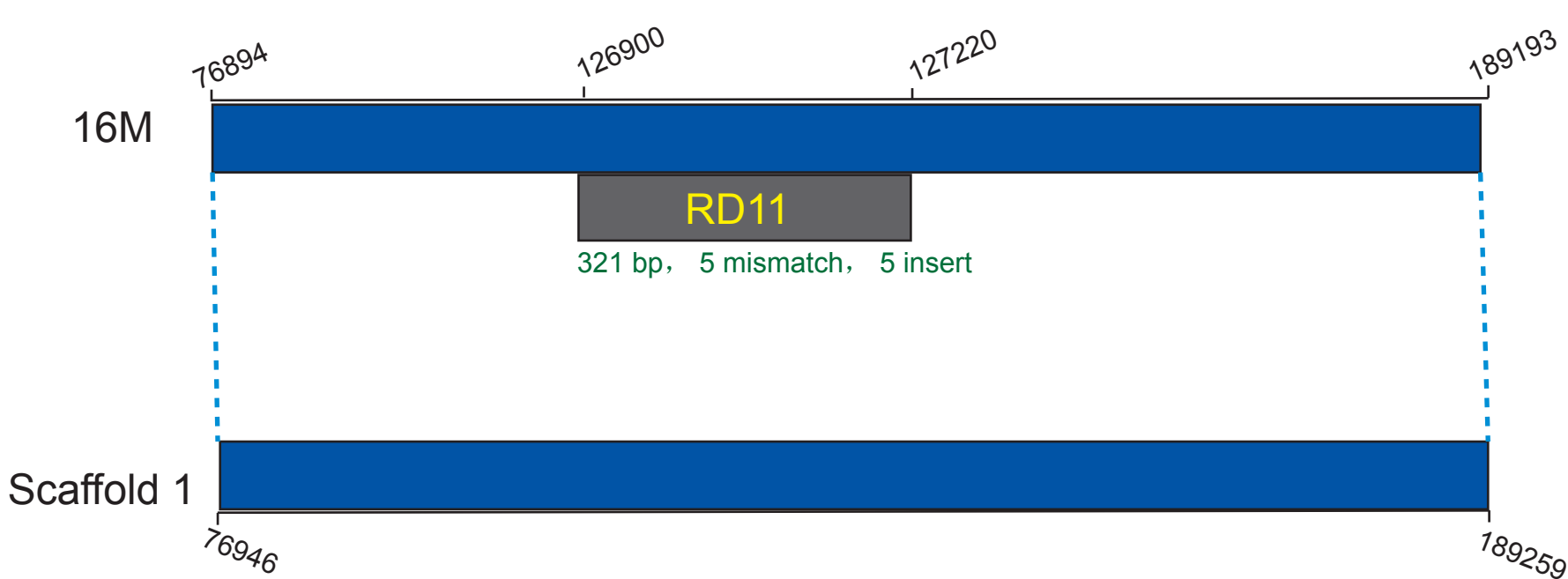

Supplement: Figure S1 — Alignment of RDs in the B. melitensis vaccine strain M5 to the virulent strain 16 M. RD1, RD2, RD4, RD5, and RD7 exhibit obvious genome insertion and deletion in these regions. The insertion and deletion caused low read coverage regions and are denoted as RDs. . Sequences of other RDs were identical with the virulent strain 16 M. Low coverage in these regions may be caused by uncertainty in sequencing. (PDF) [file pone.0070852.s001.pdf]
