# Supplementary material for: Comparative Genomic Analysis of Brucella melitensis Vaccine Strain M5 Provides Insights into Virulence Attenuation
Source: PLoS One. 2013 Aug 14;8(8):e70852. doi: 10.1371/journal.pone.0070852 (PMC3743847; doi:10.1371/journal.pone.0070852)
Supplement: Table S1 — Genome assembly of the B. melitensis vaccine strain M5. (PDF) [file pone.0070852.s002.pdf]

Table S1: Genome assembly of *B. melitensis* vaccine strain M5

Assemebling information	Scaffold	Contig
Total Number (#)	9	95
Total length (bp)	3,294,086	3,290,066
N50 Length (bp)	775,745	79,000
N90 Length (bp)	530,687	16,871
Maximum Length (bp)	1,179,763	162,942
