# Supplementary material for: Comparative Genomic Analysis of Brucella melitensis Vaccine Strain M5 Provides Insights into Virulence Attenuation
Source: PLoS One. 2013 Aug 14;8(8):e70852. doi: 10.1371/journal.pone.0070852 (PMC3743847; doi:10.1371/journal.pone.0070852)
Supplement: Table S2 — COG-based function of ORFs identified in the B. melitensis vaccine strain M5. (PDF) [file pone.0070852.s003.pdf]

Table S2: COG function for genes of *B. melitensis* vaccine strain M5

Gene name	Portein name in COG	Organism	COG id	COG class defination	Function class defination	Function code
BrucellaGL000001	RSc0578	Rso	COG2801	Transposase and inactivated derivatives	Replication, recombination and repair	L
BrucellaGL000002	BMEII0226	Bme	COG1802	Transcriptional regulators	Transcription	K
BrucellaGL000004	PA0265	Pae	COG1012	NAD-dependent aldehyde dehydrogenases	Energy production and conversion	C
BrucellaGL000005	BMEII0224	Bme	COG1804	Predicted acyl-CoA transferases/carnitine dehydratase	Energy production and conversion	C
BrucellaGL000006	BMEII0223	Bme	COG4608	ABC-type oligopeptide transport system, ATPase component	Amino acid transport and metabolism	E
BrucellaGL000007	BMEII0222	Bme	COG0444	ABC-type dipeptide/oligopeptide/nickel transport system, ATPase component	Amino acid transport and metabolism	E
BrucellaGL000008	BMEII0221	Bme	COG1173	ABC-type dipeptide/oligopeptide/nickel transport systems, permease components	Amino acid transport and metabolism	E
BrucellaGL000009	BMEII0220	Bme	COG0601	ABC-type dipeptide/oligopeptide/nickel transport systems, permease components	Amino acid transport and metabolism	E
BrucellaGL000010	BMEII0219	Bme	COG1414	Transcriptional regulator	Transcription	K
BrucellaGL000011	BMEII0218_2	Bme	COG0596	Predicted hydrolases or acyltransferases (alpha/beta hydrolase superfamily)	General function prediction only	R
BrucellaGL000012	BMEII0217	Bme	COG0747	ABC-type dipeptide transport system, periplasmic component	Amino acid transport and metabolism	E
BrucellaGL000013	BMEII0216_1	Bme	COG1071	Pyruvate/2-oxoglutarate dehydrogenase complex, dehydrogenase (E1) component, eukaryotic type, alpha subunit	Energy production and conversion	C
BrucellaGL000014	BMEII0215	Bme	COG1250	3-hydroxyacyl-CoA dehydrogenase	Lipid transport and metabolism	I
BrucellaGL000015	PA1629	Pae	COG1024	Enoyl-CoA hydratase/carnithine racemase	Lipid transport and metabolism	I
BrucellaGL000016	BMEII0213	Bme	COG1960	Acyl-CoA dehydrogenases	Lipid transport and metabolism	I
BrucellaGL000017	BMEII0212	Bme	COG2366	Protein related to penicillin acylase	General function prediction only	R
BrucellaGL000018	BMEII0211	Bme	COG2366	Protein related to penicillin acylase	General function prediction only	R
BrucellaGL000019	BMEII0210	Bme	COG0747	ABC-type dipeptide transport system, periplasmic component	Amino acid transport and metabolism	E

BrucellaGL000020	BMEII0209	Bme	COG0601	ABC-type dipeptide/oligopeptide/nickel transport systems, permease components	Amino acid transport and metabolism	E
BrucellaGL000021	BMEII0207	Bme	COG1173	ABC-type dipeptide/oligopeptide/nickel transport systems, permease components	Amino acid transport and metabolism	E
BrucellaGL000022	BMEII0206	Bme	COG0444	ABC-type dipeptide/oligopeptide/nickel transport system, ATPase component	Amino acid transport and metabolism	E
BrucellaGL000023	BMEII0205	Bme	COG4608	ABC-type oligopeptide transport system, ATPase component	Amino acid transport and metabolism	E
BrucellaGL000024	BMEII0204	Bme	COG1802	Transcriptional regulators	Transcription	K
BrucellaGL000025	BMEII0203	Bme	COG0747	ABC-type dipeptide transport system, periplasmic component	Amino acid transport and metabolism	E
BrucellaGL000026	BMEII0202	Bme	COG0601	ABC-type dipeptide/oligopeptide/nickel transport systems, permease components	Amino acid transport and metabolism	E
BrucellaGL000027	BMEII0201	Bme	COG1173	ABC-type dipeptide/oligopeptide/nickel transport systems, permease components	Amino acid transport and metabolism	E
BrucellaGL000028	BMEII0201	Bme	COG1173	ABC-type dipeptide/oligopeptide/nickel transport systems, permease components	Amino acid transport and metabolism	E
BrucellaGL000029	BMEII0200	Bme	COG0444	ABC-type dipeptide/oligopeptide/nickel transport system, ATPase component	Amino acid transport and metabolism	E
BrucellaGL000030	BMEII0199	Bme	COG4608	ABC-type oligopeptide transport system, ATPase component	Amino acid transport and metabolism	E
BrucellaGL000031	BMEII0198	Bme	COG3386	Gluconolactonase	Carbohydrate transport and metabolism	G
BrucellaGL000032	BMEII0197	Bme	COG0154	Asp-tRNAAsn/Glu-tRNAGln amidotransferase A subunit and related amidases	Translation, ribosomal structure and biogenesis	J
BrucellaGL000033	BMEII0196	Bme	COG0687	Spermidine/putrescine-binding periplasmic protein	Amino acid transport and metabolism	E
BrucellaGL000034	BMEII0195	Bme	COG1177	ABC-type spermidine/putrescine transport system, permease component II	Amino acid transport and metabolism	E
BrucellaGL000035	BMEII0194	Bme	COG1176	ABC-type spermidine/putrescine transport system, permease component I	Amino acid transport and metabolism	E
BrucellaGL000036	BMEII0193	Bme	COG3842	ABC-type spermidine/putrescine transport systems, ATPase components	Amino acid transport and metabolism	E
BrucellaGL000037	BMEII0192	Bme	COG0001	Glutamate-1-semialdehyde aminotransferase	Coenzyme transport and metabolism	H

BrucellaGL000041	BMEII0189	Bme	COG0235	Ribulose-5-phosphate 4-epimerase and related epimerases and aldolases	Carbohydrate transport and metabolism	G
BrucellaGL000042	BMEII0188	Bme	COG3395	Uncharacterized protein conserved in bacteria	Function unknown	S
BrucellaGL000043	BMEII0185	Bme	COG0277	FAD/FMN-containing dehydrogenases	Energy production and conversion	C
BrucellaGL000044	RSc0578	Rso	COG2801	Transposase and inactivated derivatives	Replication, recombination and repair	L
BrucellaGL000047	BMEII0230	Bme	COG0225	Peptide methionine sulfoxide reductase	Posttranslational modification, protein turnover, chaperones	O
BrucellaGL000048	BMEII0231	Bme	COG2900	Uncharacterized protein conserved in bacteria	Function unknown	S
BrucellaGL000049	BMEII0232	Bme	COG0035	Uracil phosphoribosyltransferase	Nucleotide transport and metabolism	F
BrucellaGL000050	BMEII0233	Bme	COG2226	Methylase involved in ubiquinone/menaquinone biosynthesis	Coenzyme transport and metabolism	H
BrucellaGL000051	BMEII0234	Bme	COG0661	Predicted unusual protein kinase	General function prediction only	R
BrucellaGL000052	BMEII0235	Bme	COG0452	Phosphopantothenoylcysteine synthetase/decarboxylase	Coenzyme transport and metabolism	H
BrucellaGL000053	BMEII0236	Bme	COG4108	Peptide chain release factor RF-3	Translation, ribosomal structure and biogenesis	J
BrucellaGL000055	BMEII0238	Bme	COG3247	Uncharacterized conserved protein	Function unknown	S
BrucellaGL000056	BMEII0239	Bme	COG1502	Phosphatidylserine/phosphatidylglycerophosphate/cardiolipin synthases and related enzymes	Lipid transport and metabolism	I
BrucellaGL000057	BMEII0240	Bme	COG3568	Metal-dependent hydrolase	General function prediction only	R
BrucellaGL000058	PA0366	Pae	COG1012	NAD-dependent aldehyde dehydrogenases	Energy production and conversion	C
BrucellaGL000059	PA0366	Pae	COG1012	NAD-dependent aldehyde dehydrogenases	Energy production and conversion	C
BrucellaGL000061	BMEII0243	Bme	COG3714	Predicted membrane protein	Function unknown	S
BrucellaGL000063	BMEII0246	Bme	COG0778	Nitroreductase	Energy production and conversion	C
BrucellaGL000064	BMEII0247	Bme	COG1574	Predicted metal-dependent hydrolase with the TIM-barrel fold	General function prediction only	R
BrucellaGL000065	BMEII0248	Bme	COG0588	Phosphoglycerate mutase 1	Carbohydrate transport and metabolism	G
BrucellaGL000066	BMEII0249	Bme	COG0289	Dihydrodipicolinate reductase	Amino acid transport and metabolism	E
BrucellaGL000067	BMEII0250	Bme	COG1132	ABC-type multidrug transport system, ATPase and permease components	Defense mechanisms	V

BrucellaGL000068	BMEII0251	Bme	COG0837	Glucokinase	Carbohydrate transport and metabolism	G
BrucellaGL000069	BMEII0252	Bme	COG1803	Methylglyoxal synthase	Carbohydrate transport and metabolism	G
BrucellaGL000070	BMEII0253	Bme	COG3770	Murein endopeptidase	Cell wall/membrane/envelope biogenesis	M
BrucellaGL000071	BMEII0254	Bme	COG0451	Nucleoside-diphosphate-sugar epimerases	Cell wall/membrane/envelope biogenesis	M
BrucellaGL000072	BMEII0255	Bme	COG1600	Uncharacterized Fe-S protein	Energy production and conversion	C
BrucellaGL000073	BMEII0256	Bme	COG0625	Glutathione S-transferase	Posttranslational modification, protein turnover, chaperones	O
BrucellaGL000074	BMEII0258	Bme	COG1968	Uncharacterized bacitracin resistance protein	Defense mechanisms	V
BrucellaGL000075	BMEII0259	Bme	COG0702	Predicted nucleoside-diphosphate-sugar epimerases	Cell wall/membrane/envelope biogenesis	M
BrucellaGL000076	BMEII0260	Bme	COG0481	Membrane GTPase LepA	Cell wall/membrane/envelope biogenesis	M
BrucellaGL000078	BMEII0261	Bme	COG2827	Predicted endonuclease containing a URI domain	Replication, recombination and repair	L
BrucellaGL000079	BMEII0262	Bme	COG3526	Uncharacterized protein conserved in bacteria	Posttranslational modification, protein turnover, chaperones	O
BrucellaGL000080	BMEII0263	Bme	COG1322	Uncharacterized protein conserved in bacteria	Function unknown	S
BrucellaGL000081	BMEII0264	Bme	COG0242	N-formylmethionyl-tRNA deformylase	Translation, ribosomal structure and biogenesis	J
BrucellaGL000082	BMEII0265	Bme	COG0223	Methionyl-tRNA formyltransferase	Translation, ribosomal structure and biogenesis	J
BrucellaGL000083	BMEII0266	Bme	COG0101	Pseudouridylate synthase	Translation, ribosomal structure and biogenesis	J
BrucellaGL000085	BMEII0268	Bme	COG0624	Acetylornithine deacetylase/Succinyl-diaminopimelate desuccinylase and related deacylases	Amino acid transport and metabolism	E
BrucellaGL000086	BMEII0269	Bme	COG3672	Predicted periplasmic protein	Function unknown	S
BrucellaGL000087	BMEII0270	Bme	COG2171	Tetrahydrodipicolinate N-succinyltransferase	Amino acid transport and metabolism	E
BrucellaGL000088	BMEII0271	Bme	COG1611	Predicted Rossmann fold nucleotide-binding protein	General function prediction only	R

BrucellaGL000089	BMEII0272	Bme	COG1011	Predicted hydrolase (HAD superfamily)	General function prediction only	R
BrucellaGL000090	BMEII0273	Bme	COG0548	Acetylglutamate kinase	Amino acid transport and metabolism	E
BrucellaGL000091	BMEII0274	Bme	COG0218	Predicted GTPase	General function prediction only	R
BrucellaGL000092	BMEII0275	Bme	COG0706	Preprotein translocase subunit YidC	Intracellular trafficking, secretion, and vesicular transport	U
BrucellaGL000093	BMEII0276	Bme	COG0594	RNase P protein component	Translation, ribosomal structure and biogenesis	J
BrucellaGL000094	BMEI0276	Bme	COG0230	Ribosomal protein L34	Translation, ribosomal structure and biogenesis	J
BrucellaGL000095	BMEII0278	Bme	COG0251	Putative translation initiation inhibitor, yjgF family	Translation, ribosomal structure and biogenesis	J
BrucellaGL000097	Z2875	EcZ	COG0477	Permeases of the major facilitator superfamily	Carbohydrate transport and metabolism	G
BrucellaGL000099	BMEII0281	Bme	COG1802	Transcriptional regulators	Transcription	K
BrucellaGL000100	RSc2350	Rso	COG1012	NAD-dependent aldehyde dehydrogenases	Energy production and conversion	C
BrucellaGL000101	XF1726	Xfa	COG1028	Dehydrogenases with different specificities (related to short-chain alcohol dehydrogenases)	Lipid transport and metabolism	I
BrucellaGL000102	BMEII0284	Bme	COG0747	ABC-type dipeptide transport system, periplasmic component	Amino acid transport and metabolism	E
BrucellaGL000103	BMEII0285	Bme	COG0601	ABC-type dipeptide/oligopeptide/nickel transport systems, permease components	Amino acid transport and metabolism	E
BrucellaGL000104	BMEII0286	Bme	COG1173	ABC-type dipeptide/oligopeptide/nickel transport systems, permease components	Amino acid transport and metabolism	E
BrucellaGL000105	BMEII0287	Bme	COG0444	ABC-type dipeptide/oligopeptide/nickel transport system, ATPase component	Amino acid transport and metabolism	E
BrucellaGL000106	BMEII0288	Bme	COG4608	ABC-type oligopeptide transport system, ATPase component	Amino acid transport and metabolism	E
BrucellaGL000107	BMEII0289	Bme	COG0154	Asp-tRNAAsn/Glu-tRNAGln amidotransferase A subunit and related amidases	Translation, ribosomal structure and biogenesis	J
BrucellaGL000108	BMEII0289	Bme	COG0154	Asp-tRNAAsn/Glu-tRNAGln amidotransferase A subunit and related amidases	Translation, ribosomal structure and biogenesis	J
BrucellaGL000109	BMEII0290	Bme	COG0847	DNA polymerase III, epsilon subunit and related 3'-5' exonucleases	Replication, recombination and repair	L

BrucellaGL000110	BMEII0291	Bme	COG0579	Predicted dehydrogenase	General function prediction only	R
BrucellaGL000111	BMEII0292	Bme	COG2199	FOG: GGDEF domain	Signal transduction mechanisms	T
BrucellaGL000114	BMEII0294	Bme	COG0625	Glutathione S-transferase	Posttranslational modification, protein turnover, chaperones	O
BrucellaGL000115	PA0880	Pae	COG0346	Lactoylglutathione lyase and related lyases	Amino acid transport and metabolism	E
BrucellaGL000117	BMEII0297	Bme	COG1629	Outer membrane receptor proteins, mostly Fe transport	Inorganic ion transport and metabolism	P
BrucellaGL000119	BMEII0299	Bme	COG1414	Transcriptional regulator	Transcription	K
BrucellaGL000120	mll1682	Mlo	COG1879	ABC-type sugar transport system, periplasmic component	Carbohydrate transport and metabolism	G
BrucellaGL000121	BMEII0300	Bme	COG1129	ABC-type sugar transport system, ATPase component	Carbohydrate transport and metabolism	G
BrucellaGL000122	BMEII0300	Bme	COG1129	ABC-type sugar transport system, ATPase component	Carbohydrate transport and metabolism	G
BrucellaGL000123	BMEII0301	Bme	COG1172	Ribose/xylose/arabinose/galactoside ABC-type transport systems, permease components	Carbohydrate transport and metabolism	G
BrucellaGL000124	BMEII0302	Bme	COG1172	Ribose/xylose/arabinose/galactoside ABC-type transport systems, permease components	Carbohydrate transport and metabolism	G
BrucellaGL000125	BMEII0303	Bme	COG0251	Putative translation initiation inhibitor, yjgF family	Translation, ribosomal structure and biogenesis	J
BrucellaGL000126	BMEII0304	Bme	COG2717	Predicted membrane protein	Function unknown	S
BrucellaGL000127	BMEII0305	Bme	COG2041	Sulfite oxidase and related enzymes	General function prediction only	R
BrucellaGL000128	BMEII0306	Bme	COG0400	Predicted esterase	General function prediction only	R
BrucellaGL000129	BMEII0307	Bme	COG2319	FOG: WD40 repeat	General function prediction only	R
BrucellaGL000130	BMEII0308	Bme	COG0523	Putative GTPases (G3E family)	General function prediction only	R
BrucellaGL000131	BMEII0309	Bme	COG1402	Uncharacterized protein, putative amidase	General function prediction only	R
BrucellaGL000132	BMEII0310	Bme	COG1846	Transcriptional regulators	Transcription	K
BrucellaGL000134	BMEII0312	Bme	COG1609	Transcriptional regulators	Transcription	K
BrucellaGL000136	BMEII0313	Bme	COG1052	Lactate dehydrogenase and related dehydrogenases	Energy production and conversion	C
BrucellaGL000137	BMEII0314	Bme	COG2071	Predicted glutamine amidotransferases	General function prediction only	R

BrucellaGL000139	MA0107	Mac	COG1028	Dehydrogenases with different specificities (related to short-chain alcohol dehydrogenases)	Lipid transport and metabolism	I
BrucellaGL000140	BMEII0317_1	Bme	COG2202	FOG: PAS/PAC domain	Signal transduction mechanisms	T
BrucellaGL000141	BMEII0318	Bme	COG1680	Beta-lactamase class C and other penicillin binding proteins	Defense mechanisms	V
BrucellaGL000145	BMEII0323	Bme	COG3288	NAD/NADP transhydrogenase alpha subunit	Energy production and conversion	C
BrucellaGL000146	BMEII0324	Bme	COG3288	NAD/NADP transhydrogenase alpha subunit	Energy production and conversion	C
BrucellaGL000147	BMEII0325	Bme	COG1282	NAD/NADP transhydrogenase beta subunit	Energy production and conversion	C
BrucellaGL000148	BMEII0326	Bme	COG4091	Predicted homoserine dehydrogenase	Amino acid transport and metabolism	E
BrucellaGL000149	BMEII0327	Bme	COG0637	Predicted phosphatase/phosphohexomutase	General function prediction only	R
BrucellaGL000150	BMEII0328	Bme	COG2030	Acyl dehydratase	Lipid transport and metabolism	I
BrucellaGL000153	BMEII0332	Bme	COG0828	Ribosomal protein S21	Translation, ribosomal structure and biogenesis	J
BrucellaGL000154	BMEII0334	Bme	COG0457	FOG: TPR repeat	General function prediction only	R
BrucellaGL000155	SMa1358	Sme	COG0346	Lactoylglutathione lyase and related lyases	Amino acid transport and metabolism	E
BrucellaGL000156	BMEII0336	Bme	COG2011	ABC-type metal ion transport system, permease component	Inorganic ion transport and metabolism	P
BrucellaGL000157	BMEII0337	Bme	COG1135	ABC-type metal ion transport system, ATPase component	Inorganic ion transport and metabolism	P
BrucellaGL000158	BMEII0338	Bme	COG1464	ABC-type metal ion transport system, periplasmic component/surface antigen	Inorganic ion transport and metabolism	P
BrucellaGL000160	SMc03116	Sme	COG2303	Choline dehydrogenase and related flavoproteins	Amino acid transport and metabolism	E
BrucellaGL000161	BMEII0340	Bme	COG4177	ABC-type branched-chain amino acid transport system, permease component	Amino acid transport and metabolism	E
BrucellaGL000162	BMEII0341	Bme	COG0559	Branched-chain amino acid ABC-type transport system, permease components	Amino acid transport and metabolism	E
BrucellaGL000163	BMEII0342	Bme	COG0410	ABC-type branched-chain amino acid transport systems, ATPase component	Amino acid transport and metabolism	E
BrucellaGL000164	BMEII0343	Bme	COG0411	ABC-type branched-chain amino acid transport systems, ATPase component	Amino acid transport and metabolism	E
BrucellaGL000165	AGl1462	Atu	COG0683	ABC-type branched-chain amino acid transport systems, periplasmic component	Amino acid transport and metabolism	E
BrucellaGL000167	BMEI0218	Bme	COG0583	Transcriptional regulator	Transcription	K

BrucellaGL000169	BMEII0346	Bme	COG1522	Transcriptional regulators	Transcription	K
BrucellaGL000170	BMEII0347_1	Bme	COG2334	Putative homoserine kinase type II (protein kinase fold)	General function prediction only	R
BrucellaGL000171	BMEII0348	Bme	COG0160	4-aminobutyrate aminotransferase and related aminotransferases	Amino acid transport and metabolism	E
BrucellaGL000172	BMEII0349	Bme	COG0834	ABC-type amino acid transport/signal transduction systems, periplasmic component/domain	Amino acid transport and metabolism	E
BrucellaGL000173	BMEII0350	Bme	COG1680	Beta-lactamase class C and other penicillin binding proteins	Defense mechanisms	V
BrucellaGL000175	BMEII0351	Bme	COG0028	Thiamine pyrophosphate-requiring enzymes [acetolactate synthase, pyruvate dehydrogenase (cytochrome), glyoxylate carboligase, phosphonopyruvate decarboxylase]	Amino acid transport and metabolism	E
BrucellaGL000177	BMEII0352	Bme	COG2186	Transcriptional regulators	Transcription	K
BrucellaGL000178	BMEII0354	Bme	COG3802	Uncharacterized protein conserved in bacteria	Function unknown	S
BrucellaGL000179	BMEII0355	Bme	COG0673	Predicted dehydrogenases and related proteins	General function prediction only	R
BrucellaGL000180	BMEII0356	Bme	COG0129	Dihydroxyacid dehydratase/phosphogluconate dehydratase	Amino acid transport and metabolism	E
BrucellaGL000181	BMEII0357	Bme	COG3734	2-keto-3-deoxy-galactonokinase	Carbohydrate transport and metabolism	G
BrucellaGL000182	BMEII0358	Bme	COG0800	2-keto-3-deoxy-6-phosphogluconate aldolase	Carbohydrate transport and metabolism	G
BrucellaGL000183	BMEII0359	Bme	COG4213	ABC-type xylose transport system, periplasmic component	Carbohydrate transport and metabolism	G
BrucellaGL000184	BMEII0360	Bme	COG4213	ABC-type xylose transport system, periplasmic component	Carbohydrate transport and metabolism	G
BrucellaGL000186	BMEII0361	Bme	COG1129	ABC-type sugar transport system, ATPase component	Carbohydrate transport and metabolism	G
BrucellaGL000187	BMEII0362	Bme	COG4214	ABC-type xylose transport system, permease component	Carbohydrate transport and metabolism	G
BrucellaGL000188	BMEII0363	Bme	COG0115	Branched-chain amino acid aminotransferase/4-amino-4-deoxychorismate lyase	Amino acid transport and metabolism	E

BrucellaGL000189	BMEII0364	Bme	COG3758	Uncharacterized protein conserved in bacteria	Function unknown	S
BrucellaGL000190	BMEII0365	Bme	COG2987	Urocanate hydratase	Amino acid transport and metabolism	E
BrucellaGL000191	BMEII0366	Bme	COG3741	N-formylglutamate amidohydrolase	Amino acid transport and metabolism	E
BrucellaGL000192	BMEII0367	Bme	COG2986	Histidine ammonia-lyase	Amino acid transport and metabolism	E
BrucellaGL000193	BMEII0367	Bme	COG2986	Histidine ammonia-lyase	Amino acid transport and metabolism	E
BrucellaGL000194	BMEII0368_1	Bme	COG1228	Imidazolonepropionase and related amidohydrolases	Secondary metabolites biosynthesis, transport and catabolism	Q
BrucellaGL000196	BMEII0369	Bme	COG0402	Cytosine deaminase and related metal-dependent hydrolases	Nucleotide transport and metabolism	F
BrucellaGL000197	BMEII0370	Bme	COG2188	Transcriptional regulators	Transcription	K
BrucellaGL000198	BMEII0371	Bme	COG0161	Adenosylmethionine-8-amino-7-oxononanoate aminotransferase	Coenzyme transport and metabolism	H
BrucellaGL000200	ycjC	Eco	COG1396	Predicted transcriptional regulators	Transcription	K
BrucellaGL000201	BMEII0373	Bme	COG0665	Glycine/D-amino acid oxidases (deaminating)	Amino acid transport and metabolism	E
BrucellaGL000202	BMEII0374	Bme	COG0787	Alanine racemase	Cell wall/membrane/envelope biogenesis	M
BrucellaGL000203	BMEII0375	Bme	COG1522	Transcriptional regulators	Transcription	K
BrucellaGL000204	AGl2258	Atu	COG3637	Opacity protein and related surface antigens	Cell wall/membrane/envelope biogenesis	M
BrucellaGL000205	BMEII0377	Bme	COG1304	L-lactate dehydrogenase (FMN-dependent) and related alpha-hydroxy acid dehydrogenases	Energy production and conversion	C
BrucellaGL000206	BMEII0378	Bme	COG0243	Anaerobic dehydrogenases, typically selenocysteine-containing	Energy production and conversion	C
BrucellaGL000209	BMEII0380	Bme	COG0845	Membrane-fusion protein	Cell wall/membrane/envelope biogenesis	M
BrucellaGL000210	BMEII0381	Bme	COG0845	Membrane-fusion protein	Cell wall/membrane/envelope biogenesis	M
BrucellaGL000211	BMEII0382	Bme	COG0841	Cation/multidrug efflux pump	Defense mechanisms	V
BrucellaGL000212	BMEII0383	Bme	COG2188	Transcriptional regulators	Transcription	K
BrucellaGL000213	BMEII0384	Bme	COG2222	Predicted phosphosugar isomerases	Cell wall/membrane/envelope biogenesis	M
BrucellaGL000214	BMEII0385	Bme	COG1820	N-acetylglucosamine-6-phosphate deacetylase	Carbohydrate transport and metabolism	G

BrucellaGL000215	BMEII0386	Bme	COG4821	Uncharacterized protein containing SIS (Sugar ISomerase) phosphosugar binding domain	General function prediction only	R
BrucellaGL000216	BMEII0387	Bme	COG0788	Formyltetrahydrofolate hydrolase	Nucleotide transport and metabolism	F
BrucellaGL000217	mll2867	Mlo	COG1012	NAD-dependent aldehyde dehydrogenases	Energy production and conversion	C
BrucellaGL000218	BMEII0389	Bme	COG5383	Uncharacterized protein conserved in bacteria	Function unknown	S
BrucellaGL000219	BMEI0896	Bme	COG0583	Transcriptional regulator	Transcription	K
BrucellaGL000220	BMEII0391	Bme	COG0665	Glycine/D-amino acid oxidases (deaminating)	Amino acid transport and metabolism	E
BrucellaGL000221	BMEII0392	Bme	COG2183	Transcriptional accessory protein	Transcription	K
BrucellaGL000222	BMEII0393	Bme	COG0640	Predicted transcriptional regulators	Transcription	K
BrucellaGL000223	BMEII0394	Bme	COG1902	NADH:flavin oxidoreductases, Old Yellow Enzyme family	Energy production and conversion	C
BrucellaGL000224	SMb21493	Sme	COG1522	Transcriptional regulators	Transcription	K
BrucellaGL000225	BMEII0396	Bme	COG0010	Arginase/agmatinase/formimionoglutamate hydrolase, arginase family	Amino acid transport and metabolism	E
BrucellaGL000226	BMEII0397	Bme	COG2423	Predicted ornithine cyclodeaminase, mu-crystallin homolog	Amino acid transport and metabolism	E
BrucellaGL000227	BMEII0398	Bme	COG2346	Truncated hemoglobins	General function prediction only	R
BrucellaGL000230	BMEII0400	Bme	COG3760	Uncharacterized conserved protein	Function unknown	S
BrucellaGL000231	BMEII0401	Bme	COG3118	Thioredoxin domain-containing protein	Posttranslational modification, protein turnover, chaperones	O
BrucellaGL000232	BMEII0402	Bme	COG2802	Uncharacterized protein, similar to the N-terminal domain of Lon protease	General function prediction only	R
BrucellaGL000233	BMEII0403	Bme	COG2835	Uncharacterized conserved protein	Function unknown	S
BrucellaGL000235	BMEII0404	Bme	COG0473	Isocitrate/isopropylmalate dehydrogenase	Energy production and conversion	C
BrucellaGL000236	BMEII0405_1	Bme	COG2169	Adenosine deaminase	Nucleotide transport and metabolism	F
BrucellaGL000237	RSc0547	Rso	COG0477	Permeases of the major facilitator superfamily	Carbohydrate transport and metabolism	G
BrucellaGL000239	BMEII0407	Bme	COG0136	Aspartate-semialdehyde dehydrogenase	Amino acid transport and metabolism	E
BrucellaGL000240	CC0915	Ccr	COG1846	Transcriptional regulators	Transcription	K
BrucellaGL000241	BMEII0409	Bme	COG1764	Predicted redox protein, regulator of disulfide bond formation	Posttranslational modification, protein turnover, chaperones	O
BrucellaGL000242	BMEII0410	Bme	COG3824	Uncharacterized protein conserved in bacteria	Function unknown	S

BrucellaGL000243	BMEII0411	Bme	COG0066	3-isopropylmalate dehydratase small subunit	Amino acid transport and metabolism	E
BrucellaGL000244	BMEII0413	Bme	COG2301	Citrate lyase beta subunit	Carbohydrate transport and metabolism	G
BrucellaGL000245	BMEII0414	Bme	COG5515	Uncharacterized conserved small protein	Function unknown	S
BrucellaGL000246	all8503	Nos	COG0346	Lactoylglutathione lyase and related lyases	Amino acid transport and metabolism	E
BrucellaGL000247	BMEII0416	Bme	COG0339	Zn-dependent oligopeptidases	Amino acid transport and metabolism	E
BrucellaGL000248	BMEII0418	Bme	COG2239	Mg/Co/Ni transporter MgtE (contains CBS domain)	Inorganic ion transport and metabolism	P
BrucellaGL000249	BMEII0419	Bme	COG1217	Predicted membrane GTPase involved in stress response	Signal transduction mechanisms	T
BrucellaGL000250	BMEII0420	Bme	COG1739	Uncharacterized conserved protein	Function unknown	S
BrucellaGL000252	BMEII0422	Bme	COG0158	Fructose-1,6-bisphosphatase	Carbohydrate transport and metabolism	G
BrucellaGL000253	BMEII0423	Bme	COG0191	Fructose/tagatose bisphosphate aldolase	Carbohydrate transport and metabolism	G
BrucellaGL000254	BMEII0424	Bme	COG0698	Ribose 5-phosphate isomerase RpiB	Carbohydrate transport and metabolism	G
BrucellaGL000255	BMEII0425	Bme	COG0149	Triosephosphate isomerase	Carbohydrate transport and metabolism	G
BrucellaGL000256	BMEII0426	Bme	COG1349	Transcriptional regulators of sugar metabolism	Transcription	K
BrucellaGL000258	BMEII0427	Bme	COG2390	Transcriptional regulator, contains sigma factor-related N-terminal domain	Transcription	K
BrucellaGL000260	BMEII0429	Bme	COG0578	Glycerol-3-phosphate dehydrogenase	Energy production and conversion	C
BrucellaGL000261	BMEII0430	Bme	COG1070	Sugar (pentulose and hexulose) kinases	Carbohydrate transport and metabolism	G
BrucellaGL000263	BMEII0431	Bme	COG5618	Predicted periplasmic lipoprotein	General function prediction only	R
BrucellaGL000265	BMEII0432	Bme	COG1129	ABC-type sugar transport system, ATPase component	Carbohydrate transport and metabolism	G
BrucellaGL000266	BMEII0433	Bme	COG1172	Ribose/xylose/arabinose/galactoside ABC-type transport systems, permease components	Carbohydrate transport and metabolism	G
BrucellaGL000267	BMEII0435	Bme	COG1879	ABC-type sugar transport system, periplasmic component	Carbohydrate transport and metabolism	G
BrucellaGL000269	BMEII0436	Bme	COG1349	Transcriptional regulators of sugar metabolism	Transcription	K

BrucellaGL000270	BMEII0437	Bme	COG0382	4-hydroxybenzoate polyprenyltransferase and related prenyltransferases	Coenzyme transport and metabolism	H
BrucellaGL000271	BMEII0438	Bme	COG0277	FAD/FMN-containing dehydrogenases	Energy production and conversion	C
BrucellaGL000272	BMEII0439	Bme	COG0697	Permeases of the drug/metabolite transporter (DMT) superfamily	Carbohydrate transport and metabolism	G
BrucellaGL000273	BMEII0440	Bme	COG0451	Nucleoside-diphosphate-sugar epimerases	Cell wall/membrane/envelope biogenesis	M
BrucellaGL000274	BMEII0441	Bme	COG4992	Ornithine/acetylornithine aminotransferase	Amino acid transport and metabolism	E
BrucellaGL000277	BMEI1001	Bme	COG3293	Transposase and inactivated derivatives	Replication, recombination and repair	L
BrucellaGL000278	BMEI1053	Bme	COG3293	Transposase and inactivated derivatives	Replication, recombination and repair	L
BrucellaGL000280	BMEII0447_2	Bme	COG3586	Uncharacterized conserved protein	Function unknown	S
BrucellaGL000281	BMEII0448	Bme	COG1451	Predicted metal-dependent hydrolase	General function prediction only	R
BrucellaGL000282	BMEII0449	Bme	COG0610	Type I site-specific restriction-modification system, R (restriction) subunit and related helicases	Defense mechanisms	V
BrucellaGL000283	BMEII0450	Bme	COG0732	Restriction endonuclease S subunits	Defense mechanisms	V
BrucellaGL000284	BMEII0451	Bme	COG0286	Type I restriction-modification system methyltransferase subunit	Defense mechanisms	V
BrucellaGL000285	BMEII0452	Bme	COG0732	Restriction endonuclease S subunits	Defense mechanisms	V
BrucellaGL000290	BMEII0460	Bme	COG2070	Dioxygenases related to 2-nitropropane dioxygenase	General function prediction only	R
BrucellaGL000294	BMEII0462	Bme	COG1201	Lhr-like helicases	General function prediction only	R
BrucellaGL000295	BMEII0463	Bme	COG1407	Predicted ICC-like phosphoesterases	General function prediction only	R
BrucellaGL000297	BMEII0465	Bme	COG0730	Predicted permeases	General function prediction only	R
BrucellaGL000298	BMEII0466_1	Bme	COG0790	FOG: TPR repeat, SEL1 subfamily	General function prediction only	R
BrucellaGL000299	BMEII0467	Bme	COG0789	Predicted transcriptional regulators	Transcription	K
BrucellaGL000300	BMEII0468	Bme	COG1993	Uncharacterized conserved protein	Function unknown	S
BrucellaGL000301	BMEII0469	Bme	COG0239	Integral membrane protein possibly involved in chromosome condensation	Cell cycle control, cell division, chromosome partitioning	D
BrucellaGL000302	BMEII0470	Bme	COG0239	Integral membrane protein possibly involved in chromosome condensation	Cell cycle control, cell division, chromosome partitioning	D

BrucellaGL000303	mll1427	Mlo	COG0845	Membrane-fusion protein	Cell wall/membrane/envelope biogenesis	M
BrucellaGL000304	BMEII0473	Bme	COG0841	Cation/multidrug efflux pump	Defense mechanisms	V
BrucellaGL000305	BMEII0474	Bme	COG1312	D-mannonate dehydratase	Carbohydrate transport and metabolism	G
BrucellaGL000306	BMEII0475	Bme	COG1802	Transcriptional regulators	Transcription	K
BrucellaGL000307	BMEII0476	Bme	COG1904	Glucuronate isomerase	Carbohydrate transport and metabolism	G
BrucellaGL000308	BMEII0477	Bme	COG1904	Glucuronate isomerase	Carbohydrate transport and metabolism	G
BrucellaGL000309	BMEII0478	Bme	COG0246	Mannitol-1-phosphate/altronate dehydrogenases	Carbohydrate transport and metabolism	G
BrucellaGL000310	BMEII0479	Bme	COG4134	ABC-type uncharacterized transport system, periplasmic component	General function prediction only	R
BrucellaGL000311	BMEII0481	Bme	COG3839	ABC-type sugar transport systems, ATPase components	Carbohydrate transport and metabolism	G
BrucellaGL000312	BMEII0483	Bme	COG1175	ABC-type sugar transport systems, permease components	Carbohydrate transport and metabolism	G
BrucellaGL000313	BMEII0484	Bme	COG1177	ABC-type spermidine/putrescine transport system, permease component II	Amino acid transport and metabolism	E
BrucellaGL000314	BMEII0485	Bme	COG2721	Altronate dehydratase	Carbohydrate transport and metabolism	G
BrucellaGL000315	BMEII0486	Bme	COG0864	Predicted transcriptional regulators containing the CopG/Arc/MetJ DNA-binding domain and a metal-binding domain	Transcription	K
BrucellaGL000316	BMEII0487	Bme	COG0747	ABC-type dipeptide transport system, periplasmic component	Amino acid transport and metabolism	E
BrucellaGL000317	ZnikB	EcZ	COG0601	ABC-type dipeptide/oligopeptide/nickel transport systems, permease components	Amino acid transport and metabolism	E
BrucellaGL000318	BMEII0489	Bme	COG1173	ABC-type dipeptide/oligopeptide/nickel transport systems, permease components	Amino acid transport and metabolism	E
BrucellaGL000319	BMEII0490	Bme	COG0444	ABC-type dipeptide/oligopeptide/nickel transport system, ATPase component	Amino acid transport and metabolism	E

BrucellaGL000320	BMEII0491	Bme	COG1124	ABC-type dipeptide/oligopeptide/nickel transport system, ATPase component	Amino acid transport and metabolism	E
BrucellaGL000321	BMEII0492	Bme	COG1960	Acyl-CoA dehydrogenases	Lipid transport and metabolism	I
BrucellaGL000322	BMEII0492	Bme	COG1960	Acyl-CoA dehydrogenases	Lipid transport and metabolism	I
BrucellaGL000324	cynR	Eco	COG0583	Transcriptional regulator	Transcription	K
BrucellaGL000326	BMEII0495	Bme	COG1960	Acyl-CoA dehydrogenases	Lipid transport and metabolism	I
BrucellaGL000327	BMEII0496	Bme	COG0183	Acetyl-CoA acetyltransferase	Lipid transport and metabolism	I
BrucellaGL000328	BMEII0497_2	Bme	COG1250	3-hydroxyacyl-CoA dehydrogenase	Lipid transport and metabolism	I
BrucellaGL000329	BMEII0498	Bme	COG2932	Predicted transcriptional regulator	Transcription	K
BrucellaGL000330	BMEII0499	Bme	COG1525	Micrococcal nuclease (thermonuclease) homologs	Replication, recombination and repair	L
BrucellaGL000331	BMEII0500	Bme	COG1384	Lysyl-tRNA synthetase (class I)	Translation, ribosomal structure and biogenesis	J
BrucellaGL000333	BMEII0502	Bme	COG0288	Carbonic anhydrase	Inorganic ion transport and metabolism	P
BrucellaGL000335	AGl2768	Atu	COG0747	ABC-type dipeptide transport system, periplasmic component	Amino acid transport and metabolism	E
BrucellaGL000336	BMEII0505	Bme	COG0601	ABC-type dipeptide/oligopeptide/nickel transport systems, permease components	Amino acid transport and metabolism	E
BrucellaGL000337	BH0349	Bha	COG1173	ABC-type dipeptide/oligopeptide/nickel transport systems, permease components	Amino acid transport and metabolism	E
BrucellaGL000338	BMEII0507	Bme	COG0444	ABC-type dipeptide/oligopeptide/nickel transport system, ATPase component	Amino acid transport and metabolism	E
BrucellaGL000339	BMEII0507	Bme	COG0444	ABC-type dipeptide/oligopeptide/nickel transport system, ATPase component	Amino acid transport and metabolism	E
BrucellaGL000340	BMEII0507	Bme	COG0444	ABC-type dipeptide/oligopeptide/nickel transport system, ATPase component	Amino acid transport and metabolism	E
BrucellaGL000341	BMEII0508	Bme	COG4608	ABC-type oligopeptide transport system, ATPase component	Amino acid transport and metabolism	E
BrucellaGL000342	BMEII0509	Bme	COG0345	Pyrroline-5-carboxylate reductase	Amino acid transport and metabolism	E
BrucellaGL000343	BMEII0510	Bme	COG0190	5,10-methylene-tetrahydrofolate dehydrogenase/Methenyl tetrahydrofolate cyclohydrolase	Coenzyme transport and metabolism	H

BrucellaGL000344	BMEII0511	Bme	COG0129	Dihydroxyacid dehydratase/phosphogluconate dehydratase	Amino acid transport and metabolism	E
BrucellaGL000345	BMEII0512	Bme	COG0363	6-phosphogluconolactonase/Glucosamine-6-phosphate isomerase/deaminase	Carbohydrate transport and metabolism	G
BrucellaGL000346	BMEII0513	Bme	COG0364	Glucose-6-phosphate 1-dehydrogenase	Carbohydrate transport and metabolism	G
BrucellaGL000347	TM1724	Tma	COG1028	Dehydrogenases with different specificities (related to short-chain alcohol dehydrogenases)	Lipid transport and metabolism	I
BrucellaGL000348	STM0382	Sty	COG0477	Permeases of the major facilitator superfamily	Carbohydrate transport and metabolism	G
BrucellaGL000349	BMEII0516	Bme	COG4541	Predicted membrane protein	Function unknown	S
BrucellaGL000350	BMEII0517	Bme	COG1296	Predicted branched-chain amino acid permease (azaleucine resistance)	Amino acid transport and metabolism	E
BrucellaGL000351	DR1894	Dra	COG1522	Transcriptional regulators	Transcription	K
BrucellaGL000354	BMEII0521	Bme	COG0665	Glycine/D-amino acid oxidases (deaminating)	Amino acid transport and metabolism	E
BrucellaGL000356	BMEII0523	Bme	COG0174	Glutamine synthetase	Amino acid transport and metabolism	E
BrucellaGL000359	AGc1163	Atu	COG0583	Transcriptional regulator	Transcription	K
BrucellaGL000360	AGc1163	Atu	COG0583	Transcriptional regulator	Transcription	K
BrucellaGL000361	BMEII0527	Bme	COG1570	Exonuclease VII, large subunit	Replication, recombination and repair	L
BrucellaGL000362	BMEII0528	Bme	COG3572	Gamma-glutamylcysteine synthetase	Coenzyme transport and metabolism	H
BrucellaGL000363	BMEII0529	Bme	COG1385	Uncharacterized protein conserved in bacteria	Function unknown	S
BrucellaGL000364	SMc00562	Sme	COG1846	Transcriptional regulators	Transcription	K
BrucellaGL000366	BMEII0531	Bme	COG1289	Predicted membrane protein	Function unknown	S
BrucellaGL000368	BMEII0533	Bme	COG1566	Multidrug resistance efflux pump	Defense mechanisms	V
BrucellaGL000370	BMEII0535	Bme	COG0614	ABC-type Fe3+-hydroxamate transport system, periplasmic component	Inorganic ion transport and metabolism	P
BrucellaGL000371	BMEII0536	Bme	COG0609	ABC-type Fe3+-siderophore transport system, permease component	Inorganic ion transport and metabolism	P
BrucellaGL000372	BMEII0537	Bme	COG1120	ABC-type cobalamin/Fe3+-siderophores transport systems, ATPase components	Inorganic ion transport and metabolism	P
BrucellaGL000373	BMEII0539	Bme	COG5476	Uncharacterized conserved protein	Function unknown	S

BrucellaGL000374	BMEII0540	Bme	COG0251	Putative translation initiation inhibitor, yjgF family	Translation, ribosomal structure and biogenesis	J
BrucellaGL000375	BMEII0541	Bme	COG0395	ABC-type sugar transport system, permease component	Carbohydrate transport and metabolism	G
BrucellaGL000376	TM1121	Tma	COG1175	ABC-type sugar transport systems, permease components	Carbohydrate transport and metabolism	G
BrucellaGL000377	BMEII0542	Bme	COG1653	ABC-type sugar transport system, periplasmic component	Carbohydrate transport and metabolism	G
BrucellaGL000378	STM2445	Sty	COG1028	Dehydrogenases with different specificities (related to short-chain alcohol dehydrogenases)	Lipid transport and metabolism	I
BrucellaGL000379	BMEII0544	Bme	COG3839	ABC-type sugar transport systems, ATPase components	Carbohydrate transport and metabolism	G
BrucellaGL000380	BMEII0545	Bme	COG1737	Transcriptional regulators	Transcription	K
BrucellaGL000381	BMEII0546	Bme	COG3616	Predicted amino acid aldolase or racemase	Amino acid transport and metabolism	E
BrucellaGL000383	BMEII0548	Bme	COG4175	ABC-type proline/glycine betaine transport system, ATPase component	Amino acid transport and metabolism	E
BrucellaGL000384	BMEII0549	Bme	COG4176	ABC-type proline/glycine betaine transport system, permease component	Amino acid transport and metabolism	E
BrucellaGL000385	BMEII0550	Bme	COG2113	ABC-type proline/glycine betaine transport systems, periplasmic components	Amino acid transport and metabolism	E
BrucellaGL000386	PAE0876	Pya	COG0697	Permeases of the drug/metabolite transporter (DMT) superfamily	Carbohydrate transport and metabolism	G
BrucellaGL000388	BMEII0553	Bme	COG1454	Alcohol dehydrogenase, class IV	Energy production and conversion	C
BrucellaGL000390	BMEII0554	Bme	COG0174	Glutamine synthetase	Amino acid transport and metabolism	E
BrucellaGL000391	BMEII0555	Bme	COG3931	Predicted N-formylglutamate amidohydrolase	Amino acid transport and metabolism	E
BrucellaGL000392	BMEII0556	Bme	COG1737	Transcriptional regulators	Transcription	K
BrucellaGL000393	BMEII0557	Bme	COG0446	Uncharacterized NAD(FAD)-dependent dehydrogenases	General function prediction only	R
BrucellaGL000394	BMEI1072	Bme	COG5457	Uncharacterized conserved small protein	Function unknown	S
BrucellaGL000395	BMEII0559	Bme	COG0404	Glycine cleavage system T protein (aminomethyltransferase)	Amino acid transport and metabolism	E
BrucellaGL000396	BMEII0560	Bme	COG0509	Glycine cleavage system H protein (lipoate-binding)	Amino acid transport and metabolism	E

BrucellaGL000397	BMEII0561_2	Bme	COG1003	Glycine cleavage system protein P (pyridoxal-binding), C-terminal domain	Amino acid transport and metabolism	E
BrucellaGL000398	AGl1389	Atu	COG1522	Transcriptional regulators	Transcription	K
BrucellaGL000399	BMEII0564_2	Bme	COG4230	Delta 1-pyrroline-5-carboxylate dehydrogenase	Energy production and conversion	C
BrucellaGL000400	BMEII0565	Bme	COG1840	ABC-type Fe3+ transport system, periplasmic component	Inorganic ion transport and metabolism	P
BrucellaGL000401	AGl194	Atu	COG1178	ABC-type Fe3+ transport system, permease component	Inorganic ion transport and metabolism	P
BrucellaGL000402	BMEII0567	Bme	COG3842	ABC-type spermidine/putrescine transport systems, ATPase components	Amino acid transport and metabolism	E
BrucellaGL000403	BMEII0568	Bme	COG0483	Archaeal fructose-1,6-bisphosphatase and related enzymes of inositol monophosphatase family	Carbohydrate transport and metabolism	G
BrucellaGL000404	BMEII0569	Bme	COG3718	Uncharacterized enzyme involved in inositol metabolism	Carbohydrate transport and metabolism	G
BrucellaGL000405	BMEII0570	Bme	COG1082	Sugar phosphate isomerases/epimerases	Carbohydrate transport and metabolism	G
BrucellaGL000406	BMEII0571	Bme	COG3962	Acetolactate synthase	Amino acid transport and metabolism	E
BrucellaGL000407	BMEII0572_1	Bme	COG0524	Sugar kinases, ribokinase family	Carbohydrate transport and metabolism	G
BrucellaGL000408	BMEII0573	Bme	COG1737	Transcriptional regulators	Transcription	K
BrucellaGL000409	AGl1682	Atu	COG0673	Predicted dehydrogenases and related proteins	General function prediction only	R
BrucellaGL000411	VC0732	Vch	COG0583	Transcriptional regulator	Transcription	K
BrucellaGL000412	BMEII0577	Bme	COG0450	Peroxiredoxin	Posttranslational modification, protein turnover, chaperones	O
BrucellaGL000413	HI0281	Hin	COG0477	Permeases of the major facilitator superfamily	Carbohydrate transport and metabolism	G
BrucellaGL000416	BMEII0580	Bme	COG2132	Putative multicopper oxidases	Secondary metabolites biosynthesis, transport and catabolism	Q
BrucellaGL000417	BMEII0581	Bme	COG2032	Cu/Zn superoxide dismutase	Inorganic ion transport and metabolism	P
BrucellaGL000418	SMa1869	Sme	COG0665	Glycine/D-amino acid oxidases (deaminating)	Amino acid transport and metabolism	E
BrucellaGL000419	SMa1869	Sme	COG0665	Glycine/D-amino acid oxidases (deaminating)	Amino acid transport and metabolism	E

BrucellaGL000420	BMEII0583	Bme	COG3842	ABC-type spermidine/putrescine transport systems, ATPase components	Amino acid transport and metabolism	E
BrucellaGL000421	BMEII0584	Bme	COG1840	ABC-type Fe3+ transport system, periplasmic component	Inorganic ion transport and metabolism	P
BrucellaGL000422	BMEII0585	Bme	COG1178	ABC-type Fe3+ transport system, permease component	Inorganic ion transport and metabolism	P
BrucellaGL000425	BMEII0587	Bme	COG3453	Uncharacterized protein conserved in bacteria	Function unknown	S
BrucellaGL000427	BMEII0588	Bme	COG1526	Uncharacterized protein required for formate dehydrogenase activity	Energy production and conversion	C
BrucellaGL000428	BMEII0589	Bme	COG0054	Riboflavin synthase beta-chain	Coenzyme transport and metabolism	H
BrucellaGL000429	BMEII0590	Bme	COG1653	ABC-type sugar transport system, periplasmic component	Carbohydrate transport and metabolism	G
BrucellaGL000430	BMEII0591	Bme	COG1175	ABC-type sugar transport systems, permease components	Carbohydrate transport and metabolism	G
BrucellaGL000431	BMEII0592	Bme	COG0395	ABC-type sugar transport system, permease component	Carbohydrate transport and metabolism	G
BrucellaGL000432	BMEII0593	Bme	COG3839	ABC-type sugar transport systems, ATPase components	Carbohydrate transport and metabolism	G
BrucellaGL000433	AGc1103	Atu	COG0477	Permeases of the major facilitator superfamily	Carbohydrate transport and metabolism	G
BrucellaGL000434	BMEII0597	Bme	COG0293	23S rRNA methylase	Translation, ribosomal structure and biogenesis	J
BrucellaGL000435	BMEII0598	Bme	COG0248	Exopolyphosphatase	Nucleotide transport and metabolism	F
BrucellaGL000436	BMEII0599	Bme	COG1126	ABC-type polar amino acid transport system, ATPase component	Amino acid transport and metabolism	E
BrucellaGL000437	BMEII0600	Bme	COG0765	ABC-type amino acid transport system, permease component	Amino acid transport and metabolism	E
BrucellaGL000438	mll3861	Mlo	COG0834	ABC-type amino acid transport/signal transduction systems, periplasmic component/domain	Amino acid transport and metabolism	E
BrucellaGL000439	BMEII0602_1	Bme	COG0145	N-methylhydantoinase A/acetone carboxylase, beta subunit	Amino acid transport and metabolism	E
BrucellaGL000440	BMEII0603	Bme	COG5631	Predicted transcription regulator, contains HTH domain (MarR family)	Transcription	K

BrucellaGL000441	BMEII0604	Bme	COG4604	ABC-type enterochelin transport system, ATPase component	Inorganic ion transport and metabolism	P
BrucellaGL000442	BMEII0605	Bme	COG4605	ABC-type enterochelin transport system, permease component	Inorganic ion transport and metabolism	P
BrucellaGL000443	BMEII0606	Bme	COG4606	ABC-type enterochelin transport system, permease component	Inorganic ion transport and metabolism	P
BrucellaGL000444	BMEII0607	Bme	COG4607	ABC-type enterochelin transport system, periplasmic component	Inorganic ion transport and metabolism	P
BrucellaGL000445	CC0419	Ccr	COG1012	NAD-dependent aldehyde dehydrogenases	Energy production and conversion	C
BrucellaGL000447	BMEII0610	Bme	COG3573	Predicted oxidoreductase	General function prediction only	R
BrucellaGL000448	BMEII0610	Bme	COG3573	Predicted oxidoreductase	General function prediction only	R
BrucellaGL000449	BMEII0611	Bme	COG0730	Predicted permeases	General function prediction only	R
BrucellaGL000450	mlr7635	Mlo	COG0115	Branched-chain amino acid aminotransferase/4-amino-4-deoxychorismate lyase	Amino acid transport and metabolism	E
BrucellaGL000451	SMc00197	Sme	COG0697	Permeases of the drug/metabolite transporter (DMT) superfamily	Carbohydrate transport and metabolism	G
BrucellaGL000454	SMb21281	Sme	COG2252	Permeases	General function prediction only	R
BrucellaGL000455	BMEII0619	Bme	COG0590	Cytosine/adenosine deaminases	Nucleotide transport and metabolism	F
BrucellaGL000456	SMc04179	Sme	COG0471	Di- and tricarboxylate transporters	Inorganic ion transport and metabolism	P
BrucellaGL000457	BMEII0620	Bme	COG0471	Di- and tricarboxylate transporters	Inorganic ion transport and metabolism	P
BrucellaGL000458	BMEII0621	Bme	COG3839	ABC-type sugar transport systems, ATPase components	Carbohydrate transport and metabolism	G
BrucellaGL000459	SMb20418	Sme	COG0395	ABC-type sugar transport system, permease component	Carbohydrate transport and metabolism	G
BrucellaGL000460	SMb20417	Sme	COG1175	ABC-type sugar transport systems, permease components	Carbohydrate transport and metabolism	G
BrucellaGL000461	BMEII0625	Bme	COG1653	ABC-type sugar transport system, periplasmic component	Carbohydrate transport and metabolism	G
BrucellaGL000462	BMEII0626	Bme	COG2355	Zn-dependent dipeptidase, microsomal dipeptidase homolog	Amino acid transport and metabolism	E
BrucellaGL000463	BMEII0627	Bme	COG1001	Adenine deaminase	Nucleotide transport and metabolism	F

BrucellaGL000464	BMEII0628	Bme	COG0410	ABC-type branched-chain amino acid transport systems, ATPase component	Amino acid transport and metabolism	E
BrucellaGL000465	BMEII0629	Bme	COG0411	ABC-type branched-chain amino acid transport systems, ATPase component	Amino acid transport and metabolism	E
BrucellaGL000466	SMb20786	Sme	COG4177	ABC-type branched-chain amino acid transport system, permease component	Amino acid transport and metabolism	E
BrucellaGL000467	BMEII0632	Bme	COG0559	Branched-chain amino acid ABC-type transport system, permease components	Amino acid transport and metabolism	E
BrucellaGL000468	AGl702	Atu	COG0683	ABC-type branched-chain amino acid transport systems, periplasmic component	Amino acid transport and metabolism	E
BrucellaGL000469	BMEII0634	Bme	COG0015	Adenylosuccinate lyase	Nucleotide transport and metabolism	F
BrucellaGL000470	BMEII0635	Bme	COG3485	Protocatechuate 3,4-dioxygenase beta subunit	Secondary metabolites biosynthesis, transport and catabolism	Q
BrucellaGL000471	BMEII0636	Bme	COG3485	Protocatechuate 3,4-dioxygenase beta subunit	Secondary metabolites biosynthesis, transport and catabolism	Q
BrucellaGL000472	BMEII0637	Bme	COG0599	Uncharacterized homolog of gamma-carboxymuconolactone decarboxylase subunit	Function unknown	S
BrucellaGL000473	BMEII0638	Bme	COG0596	Predicted hydrolases or acyltransferases (alpha/beta hydrolase superfamily)	General function prediction only	R
BrucellaGL000474	Z2299	EcZ	COG0583	Transcriptional regulator	Transcription	K
BrucellaGL000475	BMEII0640	Bme	COG0654	2-polyprenyl-6-methoxyphenol hydroxylase and related FAD-dependent oxidoreductases	Coenzyme transport and metabolism	H
BrucellaGL000476	BMEII0641	Bme	COG2207	AraC-type DNA-binding domain-containing proteins	Transcription	K
BrucellaGL000477	BMEII0642	Bme	COG1414	Transcriptional regulator	Transcription	K
BrucellaGL000478	AGl1055GM	Atu	COG1788	Acyl CoA:acetate/3-ketoacid CoA transferase, alpha subunit	Lipid transport and metabolism	I
BrucellaGL000479	BMEII0645	Bme	COG2057	Acyl CoA:acetate/3-ketoacid CoA transferase, beta subunit	Lipid transport and metabolism	I
BrucellaGL000480	mll4180	Mlo	COG0183	Acetyl-CoA acetyltransferase	Lipid transport and metabolism	I
BrucellaGL000481	Rv0552	Mtu	COG1574	Predicted metal-dependent hydrolase with the TIM-barrel fold	General function prediction only	R
BrucellaGL000482	mll4468	Mlo	COG1522	Transcriptional regulators	Transcription	K

BrucellaGL000483	mlr4469	Mlo	COG1748	Saccharopine dehydrogenase and related proteins	Amino acid transport and metabolism	E
BrucellaGL000484	BMEI1022	Bme	COG0834	ABC-type amino acid transport/signal transduction systems, periplasmic component/domain	Amino acid transport and metabolism	E
BrucellaGL000485	BMEI1104	Bme	COG0834	ABC-type amino acid transport/signal transduction systems, periplasmic component/domain	Amino acid transport and metabolism	E
BrucellaGL000486	AGc323	Atu	COG0665	Glycine/D-amino acid oxidases (deaminating)	Amino acid transport and metabolism	E
BrucellaGL000487	BMEII0648	Bme	COG2207	AraC-type DNA-binding domain-containing proteins	Transcription	K
BrucellaGL000489	BMEII0650	Bme	COG0847	DNA polymerase III, epsilon subunit and related 3'-5' exonucleases	Replication, recombination and repair	L
BrucellaGL000494	BMEII0654	Bme	COG2199	FOG: GGDEF domain	Signal transduction mechanisms	T
BrucellaGL000496	BMEII0656	Bme	COG0389	Nucleotidyltransferase/DNA polymerase involved in DNA repair	Replication, recombination and repair	L
BrucellaGL000497	BMEII0657	Bme	COG0782	Transcription elongation factor	Transcription	K
BrucellaGL000500	BMEII0659	Bme	COG0784	FOG: CheY-like receiver	Signal transduction mechanisms	T
BrucellaGL000501	BMEII0660	Bme	COG3706	Response regulator containing a CheY-like receiver domain and a GGDEF domain	Signal transduction mechanisms	T
BrucellaGL000502	BMEII0661	Bme	COG0267	Ribosomal protein L33	Translation, ribosomal structure and biogenesis	J
BrucellaGL000503	mll1210	Mlo	COG0477	Permeases of the major facilitator superfamily	Carbohydrate transport and metabolism	G
BrucellaGL000504	mlr0843	Mlo	COG0494	NTP pyrophosphohydrolases including oxidative damage repair enzymes	Replication, recombination and repair	L
BrucellaGL000505	BMEII0664	Bme	COG5349	Uncharacterized protein conserved in bacteria	Function unknown	S
BrucellaGL000506	BMEII0665	Bme	COG0557	Exoribonuclease R	Transcription	K
BrucellaGL000507	BMEII0666_1	Bme	COG0550	Topoisomerase IA	Replication, recombination and repair	L
BrucellaGL000509	BMEII0667	Bme	COG0758	Predicted Rossmann fold nucleotide-binding protein involved in DNA uptake	Replication, recombination and repair	L
BrucellaGL000510	BMEII0668	Bme	COG0344	Predicted membrane protein	Function unknown	S

BrucellaGL000511	BMEII0669	Bme	COG0044	Dihydroorotase and related cyclic amidohydrolases	Nucleotide transport and metabolism	F
BrucellaGL000512	BMEII0670	Bme	COG0540	Aspartate carbamoyltransferase, catalytic chain	Nucleotide transport and metabolism	F
BrucellaGL000513	mll0685	Mlo	COG1960	Acyl-CoA dehydrogenases	Lipid transport and metabolism	I
BrucellaGL000514	BMEII0672	Bme	COG0816	Predicted endonuclease involved in recombination (possible Holliday junction resolvase in Mycoplasmas and B. subtilis)	Replication, recombination and repair	L
BrucellaGL000516	BMEII0673	Bme	COG2220	Predicted Zn-dependent hydrolases of the beta-lactamase fold	General function prediction only	R
BrucellaGL000517	BMEII0674	Bme	COG0721	Asp-tRNAAsn/Glu-tRNAGln amidotransferase C subunit	Translation, ribosomal structure and biogenesis	J
BrucellaGL000518	BMEII0675	Bme	COG0154	Asp-tRNAAsn/Glu-tRNAGln amidotransferase A subunit and related amidases	Translation, ribosomal structure and biogenesis	J
BrucellaGL000519	BMEII0675	Bme	COG0154	Asp-tRNAAsn/Glu-tRNAGln amidotransferase A subunit and related amidases	Translation, ribosomal structure and biogenesis	J
BrucellaGL000521	BMEII0676	Bme	COG0187	Type IIA topoisomerase (DNA gyrase/topo II, topoisomerase IV), B subunit	Replication, recombination and repair	L
BrucellaGL000522	AGc2870	Atu	COG0697	Permeases of the drug/metabolite transporter (DMT) superfamily	Carbohydrate transport and metabolism	G
BrucellaGL000523	BMEII0678	Bme	COG0321	Lipoate-protein ligase B	Coenzyme transport and metabolism	H
BrucellaGL000524	BMEII0679_2	Bme	COG3920	Signal transduction histidine kinase	Signal transduction mechanisms	T
BrucellaGL000526	BMEII0681	Bme	COG3946	Type IV secretory pathway, VirJ component	Intracellular trafficking, secretion, and vesicular transport	U
BrucellaGL000527	BMEII0682_2	Bme	COG2898	Uncharacterized conserved protein	Function unknown	S
BrucellaGL000528	BMEII0683	Bme	COG0785	Cytochrome c biogenesis protein	Posttranslational modification, protein turnover, chaperones	O
BrucellaGL000529	BMEII0684	Bme	COG1207	N-acetylglucosamine-1-phosphate uridyltransferase (contains nucleotidyltransferase and I-patch acetyltransferase domains)	Cell wall/membrane/envelope biogenesis	M
BrucellaGL000530	BMEII0685	Bme	COG0449	Glucosamine 6-phosphate synthetase, contains amidotransferase and phosphosugar isomerase domains	Cell wall/membrane/envelope biogenesis	M
BrucellaGL000531	BMEII0686	Bme	COG1200	RecG-like helicase	Replication, recombination and repair	L

BrucellaGL000532	BMEII0687	Bme	COG2938	Uncharacterized conserved protein	Function unknown	S
BrucellaGL000533	BMEII0688	Bme	COG1197	Transcription-repair coupling factor (superfamily II helicase)	Replication, recombination and repair	L
BrucellaGL000534	lin1810	Lin	COG0346	Lactoylglutathione lyase and related lyases	Amino acid transport and metabolism	E
BrucellaGL000535	BMEII0690	Bme	COG2761	Predicted dithiol-disulfide isomerase involved in polyketide biosynthesis	Secondary metabolites biosynthesis, transport and catabolism	Q
BrucellaGL000536	BMEII0691	Bme	COG4166	ABC-type oligopeptide transport system, periplasmic component	Amino acid transport and metabolism	E
BrucellaGL000538	BMEII0692	Bme	COG5342	Invasion protein B, involved in pathogenesis	General function prediction only	R
BrucellaGL000539	BMEII0693	Bme	COG3785	Uncharacterized conserved protein	Function unknown	S
BrucellaGL000540	BMEII0694	Bme	COG0654	2-polyprenyl-6-methoxyphenol hydroxylase and related FAD-dependent oxidoreductases	Coenzyme transport and metabolism	H
BrucellaGL000541	BMEII0695	Bme	COG1183	Phosphatidylserine synthase	Lipid transport and metabolism	I
BrucellaGL000542	BMEII0696	Bme	COG0604	NADPH:quinone reductase and related Zn-dependent oxidoreductases	Energy production and conversion	C
BrucellaGL000543	BMEII0697	Bme	COG0604	NADPH:quinone reductase and related Zn-dependent oxidoreductases	Energy production and conversion	C
BrucellaGL000544	BMEII0698	Bme	COG3845	ABC-type uncharacterized transport systems, ATPase components	General function prediction only	R
BrucellaGL000545	BMEII0699	Bme	COG4603	ABC-type uncharacterized transport system, permease component	General function prediction only	R
BrucellaGL000546	BMEII0700	Bme	COG4603	ABC-type uncharacterized transport system, permease component	General function prediction only	R
BrucellaGL000547	BMEII0701	Bme	COG1079	Uncharacterized ABC-type transport system, permease component	General function prediction only	R
BrucellaGL000548	BMEII0702	Bme	COG1744	Uncharacterized ABC-type transport system, periplasmic component/surface lipoprotein	General function prediction only	R
BrucellaGL000549	BMEII0703	Bme	COG0621	2-methylthioadenine synthetase	Translation, ribosomal structure and biogenesis	J
BrucellaGL000551	BMEII0704	Bme	COG2193	Bacterioferritin (cytochrome b1)	Inorganic ion transport and metabolism	P
BrucellaGL000552	BMEII0706	Bme	COG2329	Uncharacterized enzyme involved in biosynthesis of extracellular polysaccharides	General function prediction only	R

BrucellaGL000553	BMEII0707	Bme	COG1959	Predicted transcriptional regulator	Transcription	K
BrucellaGL000554	BMEII0708	Bme	COG2606	Uncharacterized conserved protein	Function unknown	S
BrucellaGL000556	mll5949	Mlo	COG3293	Transposase and inactivated derivatives	Replication, recombination and repair	L
BrucellaGL000557	BMEII0711	Bme	COG2963	Transposase and inactivated derivatives	Replication, recombination and repair	L
BrucellaGL000558	BMEII0713	Bme	COG3436	Transposase and inactivated derivatives	Replication, recombination and repair	L
BrucellaGL000560	BMEI1398	Bme	COG3293	Transposase and inactivated derivatives	Replication, recombination and repair	L
BrucellaGL000562	BMEI1001	Bme	COG3293	Transposase and inactivated derivatives	Replication, recombination and repair	L
BrucellaGL000563	BMEII0721	Bme	COG2207	AraC-type DNA-binding domain-containing proteins	Transcription	K
BrucellaGL000566	BMEII0724	Bme	COG4124	Beta-mannanase	Carbohydrate transport and metabolism	G
BrucellaGL000568	BMEII0727	Bme	COG1004	Predicted UDP-glucose 6-dehydrogenase	Cell wall/membrane/envelope biogenesis	M
BrucellaGL000569	BMEII0728	Bme	COG1215	Glycosyltransferases, probably involved in cell wall biogenesis	Cell wall/membrane/envelope biogenesis	M
BrucellaGL000570	SMb20460	Sme	COG1215	Glycosyltransferases, probably involved in cell wall biogenesis	Cell wall/membrane/envelope biogenesis	M
BrucellaGL000571	BMEII0730	Bme	COG1087	UDP-glucose 4-epimerase	Cell wall/membrane/envelope biogenesis	M
BrucellaGL000572	BMEII0731	Bme	COG0451	Nucleoside-diphosphate-sugar epimerases	Cell wall/membrane/envelope biogenesis	M
BrucellaGL000573	BMEII0733	Bme	COG5455	Predicted integral membrane protein	Function unknown	S
BrucellaGL000574	BMEII0734	Bme	COG4166	ABC-type oligopeptide transport system, periplasmic component	Amino acid transport and metabolism	E
BrucellaGL000575	BMEII0735	Bme	COG4166	ABC-type oligopeptide transport system, periplasmic component	Amino acid transport and metabolism	E
BrucellaGL000576	AGl1206	Atu	COG0601	ABC-type dipeptide/oligopeptide/nickel transport systems, permease components	Amino acid transport and metabolism	E
BrucellaGL000577	AGl1208	Atu	COG1173	ABC-type dipeptide/oligopeptide/nickel transport systems, permease components	Amino acid transport and metabolism	E
BrucellaGL000578	BMEII0738	Bme	COG4172	ABC-type uncharacterized transport system, duplicated ATPase component	General function prediction only	R
BrucellaGL000579	BMEII0739	Bme	COG3145	Alkylated DNA repair protein	Replication, recombination and repair	L

BrucellaGL000580	SMa1447	Sme	COG0477	Permeases of the major facilitator superfamily	Carbohydrate transport and metabolism	G
BrucellaGL000581	BMEII0741	Bme	COG3191	L-aminopeptidase/D-esterase	Amino acid transport and metabolism	E
BrucellaGL000582	BMEII0742	Bme	COG1674	DNA segregation ATPase FtsK/SpoIIIE and related proteins	Cell cycle control, cell division, chromosome partitioning	D
BrucellaGL000583	BMEII0743	Bme	COG3346	Uncharacterized conserved protein	Function unknown	S
BrucellaGL000584	BMEII0745	Bme	COG1249	Pyruvate/2-oxoglutarate dehydrogenase complex, dihydrolipoamide dehydrogenase (E3) component, and related enzymes	Energy production and conversion	C
BrucellaGL000585	BMEII0746	Bme	COG0508	Pyruvate/2-oxoglutarate dehydrogenase complex, dihydrolipoamide acyltransferase (E2) component, and related enzymes	Energy production and conversion	C
BrucellaGL000586	BMEII0747	Bme	COG0022	Pyruvate/2-oxoglutarate dehydrogenase complex, dehydrogenase (E1) component, eukaryotic type, beta subunit	Energy production and conversion	C
BrucellaGL000587	BMEII0748	Bme	COG1071	Pyruvate/2-oxoglutarate dehydrogenase complex, dehydrogenase (E1) component, eukaryotic type, alpha subunit	Energy production and conversion	C
BrucellaGL000588	BMEII0750	Bme	COG3839	ABC-type sugar transport systems, ATPase components	Carbohydrate transport and metabolism	G
BrucellaGL000589	PH1881	Pho	COG0673	Predicted dehydrogenases and related proteins	General function prediction only	R
BrucellaGL000590	BMEII0752	Bme	COG0395	ABC-type sugar transport system, permease component	Carbohydrate transport and metabolism	G
BrucellaGL000591	BMEII0753	Bme	COG1175	ABC-type sugar transport systems, permease components	Carbohydrate transport and metabolism	G
BrucellaGL000593	BMEII0754	Bme	COG1653	ABC-type sugar transport system, periplasmic component	Carbohydrate transport and metabolism	G
BrucellaGL000594	BMEII0755	Bme	COG1653	ABC-type sugar transport system, periplasmic component	Carbohydrate transport and metabolism	G
BrucellaGL000595	BMEII0756	Bme	COG2971	Predicted N-acetylglucosamine kinase	Carbohydrate transport and metabolism	G
BrucellaGL000596	BMEII0757	Bme	COG2188	Transcriptional regulators	Transcription	K
BrucellaGL000597	BMEII0758	Bme	COG4890	Predicted outer membrane lipoprotein	Function unknown	S

BrucellaGL000598	BMEII0759	Bme	COG1294	Cytochrome bd-type quinol oxidase, subunit 2	Energy production and conversion	C
BrucellaGL000599	BMEII0760	Bme	COG1271	Cytochrome bd-type quinol oxidase, subunit 1	Energy production and conversion	C
BrucellaGL000600	BMEII0761	Bme	COG4987	ABC-type transport system involved in cytochrome bd biosynthesis, fused ATPase and permease components	Energy production and conversion	C
BrucellaGL000601	BMEII0762	Bme	COG4988	ABC-type transport system involved in cytochrome bd biosynthesis, ATPase and permease components	Energy production and conversion	C
BrucellaGL000602	BMEII0763	Bme	COG1510	Predicted transcriptional regulators	Transcription	K
BrucellaGL000603	BMEII0766	Bme	COG2212	Multisubunit Na+/H+ antiporter, MnhF subunit	Inorganic ion transport and metabolism	P
BrucellaGL000604	BMEII0765	Bme	COG1320	Multisubunit Na+/H+ antiporter, MnhG subunit	Inorganic ion transport and metabolism	P
BrucellaGL000605	BMEII0767	Bme	COG1863	Multisubunit Na+/H+ antiporter, MnhE subunit	Inorganic ion transport and metabolism	P
BrucellaGL000606	BMEII0768	Bme	COG0651	Formate hydrogenlyase subunit 3/Multisubunit Na+/H+ antiporter, MnhD subunit	Energy production and conversion	C
BrucellaGL000607	BMEII0769	Bme	COG1006	Multisubunit Na+/H+ antiporter, MnhC subunit	Inorganic ion transport and metabolism	P
BrucellaGL000608	BMEII0770_1	Bme	COG1009	NADH:ubiquinone oxidoreductase subunit 5 (chain L)/Multisubunit Na+/H+ antiporter, MnhA subunit	Energy production and conversion	C
BrucellaGL000609	BMEII0771	Bme	COG4529	Uncharacterized protein conserved in bacteria	Function unknown	S
BrucellaGL000610	BMEII0771	Bme	COG4529	Uncharacterized protein conserved in bacteria	Function unknown	S
BrucellaGL000611	BMEII0772	Bme	COG3560	Predicted oxidoreductase related to nitroreductase	General function prediction only	R
BrucellaGL000612	BMEII0773	Bme	COG0521	Molybdopterin biosynthesis enzymes	Coenzyme transport and metabolism	H
BrucellaGL000614	BMEII0775	Bme	COG0502	Biotin synthase and related enzymes	Coenzyme transport and metabolism	H
BrucellaGL000615	BMEII0776	Bme	COG0156	7-keto-8-aminopelargonate synthetase and related enzymes	Coenzyme transport and metabolism	H
BrucellaGL000616	BMEII0777	Bme	COG0132	Dethiobiotin synthetase	Coenzyme transport and metabolism	H
BrucellaGL000617	BMEII0778	Bme	COG0161	Adenosylmethionine-8-amino-7-oxononanoate aminotransferase	Coenzyme transport and metabolism	H
BrucellaGL000618	BMEII0779	Bme	COG0332	3-oxoacyl-[acyl-carrier-protein] synthase III	Lipid transport and metabolism	I

BrucellaGL000620	BMEII0781	Bme	COG1897	Homoserine trans-succinylase	Amino acid transport and metabolism	E
BrucellaGL000621	BMEII0782	Bme	COG3757	Lyzozyme M1 (1,4-beta-N-acetylmuramidase)	Cell wall/membrane/envelope biogenesis	M
BrucellaGL000622	BMEII0782	Bme	COG3757	Lyzozyme M1 (1,4-beta-N-acetylmuramidase)	Cell wall/membrane/envelope biogenesis	M
BrucellaGL000623	BMEII0783	Bme	COG1115	Na+/alanine symporter	Amino acid transport and metabolism	E
BrucellaGL000625	BMEII0784	Bme	COG3324	Predicted enzyme related to lactoylglutathione lyase	General function prediction only	R
BrucellaGL000626	AGc1559	Atu	COG1846	Transcriptional regulators	Transcription	K
BrucellaGL000627	BMEII0786	Bme	COG1252	NADH dehydrogenase, FAD-containing subunit	Energy production and conversion	C
BrucellaGL000628	BMEII0787	Bme	COG3189	Uncharacterized conserved protein	Function unknown	S
BrucellaGL000631	SMb20219	Sme	COG0745	Response regulators consisting of a CheY-like receiver domain and a winged-helix DNA-binding domain	Signal transduction mechanisms	T
BrucellaGL000632	SMb20218	Sme	COG0642	Signal transduction histidine kinase	Signal transduction mechanisms	T
BrucellaGL000633	BMEII0793	Bme	COG1566	Multidrug resistance efflux pump	Defense mechanisms	V
BrucellaGL000634	AGc2011	Atu	COG0477	Permeases of the major facilitator superfamily	Carbohydrate transport and metabolism	G
BrucellaGL000635	AGc2011	Atu	COG0477	Permeases of the major facilitator superfamily	Carbohydrate transport and metabolism	G
BrucellaGL000636	BMEI0253	Bme	COG1846	Transcriptional regulators	Transcription	K
BrucellaGL000637	BMEII0797	Bme	COG0715	ABC-type nitrate/sulfonate/bicarbonate transport systems, periplasmic components	Inorganic ion transport and metabolism	P
BrucellaGL000638	BMEII0798	Bme	COG1116	ABC-type nitrate/sulfonate/bicarbonate transport system, ATPase component	Inorganic ion transport and metabolism	P
BrucellaGL000639	BMEII0799	Bme	COG0600	ABC-type nitrate/sulfonate/bicarbonate transport system, permease component	Inorganic ion transport and metabolism	P
BrucellaGL000640	BMEII0800	Bme	COG1917	Uncharacterized conserved protein, contains double-stranded beta-helix domain	Function unknown	S
BrucellaGL000641	BMEII0801	Bme	COG0842	ABC-type multidrug transport system, permease component	Defense mechanisms	V
BrucellaGL000642	BMEII0802	Bme	COG1131	ABC-type multidrug transport system, ATPase component	Defense mechanisms	V

BrucellaGL000643	RSc0163	Rso	COG0845	Membrane-fusion protein	Cell wall/membrane/envelope biogenesis	M
BrucellaGL000644	STM0819	Sty	COG1309	Transcriptional regulator	Transcription	K
BrucellaGL000645	BMEII0806	Bme	COG0628	Predicted permease	General function prediction only	R
BrucellaGL000646	PA1520	Pae	COG1802	Transcriptional regulators	Transcription	K
BrucellaGL000647	BMEII0808	Bme	COG2391	Predicted transporter component	General function prediction only	R
BrucellaGL000648	BMEII0809	Bme	COG2391	Predicted transporter component	General function prediction only	R
BrucellaGL000649	BMEII0810	Bme	COG0640	Predicted transcriptional regulators	Transcription	K
BrucellaGL000650	BMEII0811	Bme	COG2354	Uncharacterized protein conserved in bacteria	Function unknown	S
BrucellaGL000651	BMEII0812	Bme	COG0242	N-formylmethionyl-tRNA deformylase	Translation, ribosomal structure and biogenesis	J
BrucellaGL000652	BMEII0813	Bme	COG0111	Phosphoglycerate dehydrogenase and related dehydrogenases	Coenzyme transport and metabolism	H
BrucellaGL000654	SMa1454	Sme	COG2207	AraC-type DNA-binding domain-containing proteins	Transcription	K
BrucellaGL000655	BMEII0815	Bme	COG0365	Acyl-coenzyme A synthetases/AMP-(fatty) acid ligases	Lipid transport and metabolism	I
BrucellaGL000656	BMEII0815	Bme	COG0365	Acyl-coenzyme A synthetases/AMP-(fatty) acid ligases	Lipid transport and metabolism	I
BrucellaGL000657	CC0124	Ccr	COG1028	Dehydrogenases with different specificities (related to short-chain alcohol dehydrogenases)	Lipid transport and metabolism	I
BrucellaGL000658	BMEII0817	Bme	COG0183	Acetyl-CoA acetyltransferase	Lipid transport and metabolism	I
BrucellaGL000659	RSc0274	Rso	COG1960	Acyl-CoA dehydrogenases	Lipid transport and metabolism	I
BrucellaGL000660	BMEII0819	Bme	COG0229	Conserved domain frequently associated with peptide methionine sulfoxide reductase	Posttranslational modification, protein turnover, chaperones	O
BrucellaGL000661	DRA0336	Dra	COG0583	Transcriptional regulator	Transcription	K
BrucellaGL000662	BU588	Buc	COG0477	Permeases of the major facilitator superfamily	Carbohydrate transport and metabolism	G
BrucellaGL000663	STM0328	Sty	COG0477	Permeases of the major facilitator superfamily	Carbohydrate transport and metabolism	G
BrucellaGL000664	BMEII0823	Bme	COG0554	Glycerol kinase	Energy production and conversion	C
BrucellaGL000665	BMEII0824	Bme	COG0554	Glycerol kinase	Energy production and conversion	C

BrucellaGL000666	BMEII0825	Bme	COG2233	Xanthine/uracil permeases	Nucleotide transport and metabolism	F
BrucellaGL000668	BMEII0826	Bme	COG2120	Uncharacterized proteins, LmbE homologs	Function unknown	S
BrucellaGL000669	BMEII0827	Bme	COG1208	Nucleoside-diphosphate-sugar pyrophosphorylase involved in lipopolysaccharide biosynthesis/translation initiation factor 2B, gamma/epsilon subunits (eIF-2Bgamma/eIF-2Bepsilon)	Cell wall/membrane/envelope biogenesis	M
BrucellaGL000670	BMEII0829	Bme	COG0500	SAM-dependent methyltransferases	Secondary metabolites biosynthesis, transport and catabolism	Q
BrucellaGL000671	BMEII0830	Bme	COG1898	dTDP-4-dehydrorhamnose 3,5-epimerase and related enzymes	Cell wall/membrane/envelope biogenesis	M
BrucellaGL000672	BMEII0831	Bme	COG4310	Uncharacterized protein conserved in bacteria with an aminopeptidase-like domain	General function prediction only	R
BrucellaGL000673	BMEII0832	Bme	COG0451	Nucleoside-diphosphate-sugar epimerases	Cell wall/membrane/envelope biogenesis	M
BrucellaGL000676	BMEII0834	Bme	COG0001	Glutamate-1-semialdehyde aminotransferase	Coenzyme transport and metabolism	H
BrucellaGL000677	BMEII0835	Bme	COG0438	Glycosyltransferase	Cell wall/membrane/envelope biogenesis	M
BrucellaGL000678	BMEII0835	Bme	COG0438	Glycosyltransferase	Cell wall/membrane/envelope biogenesis	M
BrucellaGL000679	BMEII0836	Bme	COG2327	Uncharacterized conserved protein	Function unknown	S
BrucellaGL000680	BMEII0837	Bme	COG0463	Glycosyltransferases involved in cell wall biogenesis	Cell wall/membrane/envelope biogenesis	M
BrucellaGL000681	BMEII0838	Bme	COG2244	Membrane protein involved in the export of O-antigen and teichoic acid	General function prediction only	R
BrucellaGL000682	BMEII0839	Bme	COG0472	UDP-N-acetylmuramyl pentapeptide phosphotransferase/UDP-N-acetylglucosamine-1-phosphate transferase	Cell wall/membrane/envelope biogenesis	M
BrucellaGL000683	BMEII0840	Bme	COG0463	Glycosyltransferases involved in cell wall biogenesis	Cell wall/membrane/envelope biogenesis	M
BrucellaGL000684	BMEII0841	Bme	COG1434	Uncharacterized conserved protein	Function unknown	S
BrucellaGL000686	BMEII0843	Bme	COG0110	Acetyltransferase (isoleucine patch superfamily)	General function prediction only	R

BrucellaGL000687	BMEI0402	Bme	COG3637	Opacity protein and related surface antigens	Cell wall/membrane/envelope biogenesis	M
BrucellaGL000688	BMEII0845	Bme	COG0438	Glycosyltransferase	Cell wall/membrane/envelope biogenesis	M
BrucellaGL000689	STM2100	Sty	COG0438	Glycosyltransferase	Cell wall/membrane/envelope biogenesis	M
BrucellaGL000690	SA0155	Sau	COG0438	Glycosyltransferase	Cell wall/membrane/envelope biogenesis	M
BrucellaGL000692	BMEII0848	Bme	COG1089	GDP-D-mannose dehydratase	Cell wall/membrane/envelope biogenesis	M
BrucellaGL000693	BMEII0849	Bme	COG0451	Nucleoside-diphosphate-sugar epimerases	Cell wall/membrane/envelope biogenesis	M
BrucellaGL000694	BMEII0850	Bme	COG0451	Nucleoside-diphosphate-sugar epimerases	Cell wall/membrane/envelope biogenesis	M
BrucellaGL000695	BMEII0851_1	Bme	COG1596	Periplasmic protein involved in polysaccharide export	Cell wall/membrane/envelope biogenesis	M
BrucellaGL000696	BMEII0852	Bme	COG3206	Uncharacterized protein involved in exopolysaccharide biosynthesis	Cell wall/membrane/envelope biogenesis	M
BrucellaGL000697	BMEII0853	Bme	COG2197	Response regulator containing a CheY-like receiver domain and an HTH DNA-binding domain	Signal transduction mechanisms	T
BrucellaGL000698	BMEII0854	Bme	COG0664	cAMP-binding proteins - catabolite gene activator and regulatory subunit of cAMP-dependent protein kinases	Signal transduction mechanisms	T
BrucellaGL000701	BMEII0856	Bme	COG3055	Uncharacterized protein conserved in bacteria	Function unknown	S
BrucellaGL000702	BMEII0857_2	Bme	COG1940	Transcriptional regulator/sugar kinase	Transcription	K
BrucellaGL000703	BMEII0858	Bme	COG2186	Transcriptional regulators	Transcription	K
BrucellaGL000704	PH0807	Pho	COG0747	ABC-type dipeptide transport system, periplasmic component	Amino acid transport and metabolism	E
BrucellaGL000705	CAC0177	Cac	COG0601	ABC-type dipeptide/oligopeptide/nickel transport systems, permease components	Amino acid transport and metabolism	E
BrucellaGL000706	BS_appC	Bsu	COG1173	ABC-type dipeptide/oligopeptide/nickel transport systems, permease components	Amino acid transport and metabolism	E
BrucellaGL000707	BMEII0862	Bme	COG0329	Dihydrodipicolinate synthase/N-acetylneuraminate lyase	Amino acid transport and metabolism	E

BrucellaGL000708	DR1568	Dra	COG0444	ABC-type dipeptide/oligopeptide/nickel transport system, ATPase component	Amino acid transport and metabolism	E
BrucellaGL000709	BMEII0864	Bme	COG4608	ABC-type oligopeptide transport system, ATPase component	Amino acid transport and metabolism	E
BrucellaGL000710	PAB1139	Pab	COG0673	Predicted dehydrogenases and related proteins	General function prediction only	R
BrucellaGL000711	SSO3049	Sso	COG0673	Predicted dehydrogenases and related proteins	General function prediction only	R
BrucellaGL000712	BMEII0867	Bme	COG1062	Zn-dependent alcohol dehydrogenases, class III	Energy production and conversion	C
BrucellaGL000713	APE2521	Ape	COG0683	ABC-type branched-chain amino acid transport systems, periplasmic component	Amino acid transport and metabolism	E
BrucellaGL000714	mll1168	Mlo	COG1012	NAD-dependent aldehyde dehydrogenases	Energy production and conversion	C
BrucellaGL000715	CC0945	Ccr	COG2303	Choline dehydrogenase and related flavoproteins	Amino acid transport and metabolism	E
BrucellaGL000716	BMEII0871	Bme	COG0169	Shikimate 5-dehydrogenase	Amino acid transport and metabolism	E
BrucellaGL000717	BS_fabG	Bsu	COG1028	Dehydrogenases with different specificities (related to short-chain alcohol dehydrogenases)	Lipid transport and metabolism	I
BrucellaGL000718	BMEII0873	Bme	COG0410	ABC-type branched-chain amino acid transport systems, ATPase component	Amino acid transport and metabolism	E
BrucellaGL000719	BMEII0874_1	Bme	COG0559	Branched-chain amino acid ABC-type transport system, permease components	Amino acid transport and metabolism	E
BrucellaGL000720	APE2521	Ape	COG0683	ABC-type branched-chain amino acid transport systems, periplasmic component	Amino acid transport and metabolism	E
BrucellaGL000721	BMEII0876	Bme	COG0604	NADPH:quinone reductase and related Zn-dependent oxidoreductases	Energy production and conversion	C
BrucellaGL000722	BMEII0226	Bme	COG1802	Transcriptional regulators	Transcription	K
BrucellaGL000723	BMEII0879	Bme	COG2124	Cytochrome P450	Secondary metabolites biosynthesis, transport and catabolism	Q
BrucellaGL000725	BMEII0880	Bme	COG0282	Acetate kinase	Energy production and conversion	C
BrucellaGL000726	BMEII0881	Bme	COG3957	Phosphoketolase	Carbohydrate transport and metabolism	G
BrucellaGL000727	BMEII0882	Bme	COG0348	Polyferredoxin	Energy production and conversion	C
BrucellaGL000728	BMEII0883	Bme	COG0672	High-affinity Fe2+/Pb2+ permease	Inorganic ion transport and metabolism	P
BrucellaGL000730	BMEII0885	Bme	COG3470	Uncharacterized protein probably involved in high-affinity Fe2+ transport	Inorganic ion transport and metabolism	P

BrucellaGL000731	mlr6950_2	Mlo	COG0519	GMP synthase, PP-ATPase domain/subunit	Nucleotide transport and metabolism	F
BrucellaGL000732	BMEII0888	Bme	COG0775	Nucleoside phosphorylase	Nucleotide transport and metabolism	F
BrucellaGL000733	BMEII0889	Bme	COG2050	Uncharacterized protein, possibly involved in aromatic compounds catabolism	Secondary metabolites biosynthesis, transport and catabolism	Q
BrucellaGL000734	BMEII0890	Bme	COG0144	tRNA and rRNA cytosine-C5-methylases	Translation, ribosomal structure and biogenesis	J
BrucellaGL000735	AGc495	Atu	COG0144	tRNA and rRNA cytosine-C5-methylases	Translation, ribosomal structure and biogenesis	J
BrucellaGL000736	BMEII0891	Bme	COG1495	Disulfide bond formation protein DsbB	Posttranslational modification, protein turnover, chaperones	O
BrucellaGL000738	BMEII0893	Bme	COG0753	Catalase	Inorganic ion transport and metabolism	P
BrucellaGL000739	PM1346	Pmu	COG0583	Transcriptional regulator	Transcription	K
BrucellaGL000740	BMEII0895	Bme	COG3258	Cytochrome c	Energy production and conversion	C
BrucellaGL000741	BMEII0896_3	Bme	COG0516	IMP dehydrogenase/GMP reductase	Nucleotide transport and metabolism	F
BrucellaGL000742	BMEII0897	Bme	COG0038	Chloride channel protein EriC	Inorganic ion transport and metabolism	P
BrucellaGL000743	BMEII0898	Bme	COG3191	L-aminopeptidase/D-esterase	Amino acid transport and metabolism	E
BrucellaGL000744	BMEII0899	Bme	COG1109	Phosphomannomutase	Carbohydrate transport and metabolism	G
BrucellaGL000745	BMEII0900_1	Bme	COG0836	Mannose-1-phosphate guanylyltransferase	Cell wall/membrane/envelope biogenesis	M
BrucellaGL000748	BMEII0904	Bme	COG4943	Predicted signal transduction protein containing sensor and EAL domains	Signal transduction mechanisms	T
BrucellaGL000751	BMEII0907	Bme	COG2066	Glutaminase	Amino acid transport and metabolism	E
BrucellaGL000752	BMEII0908	Bme	COG2066	Glutaminase	Amino acid transport and metabolism	E
BrucellaGL000753	BMEII0909	Bme	COG0531	Amino acid transporters	Amino acid transport and metabolism	E
BrucellaGL000755	BMEII0910	Bme	COG0076	Glutamate decarboxylase and related PLP-dependent proteins	Amino acid transport and metabolism	E
BrucellaGL000756	BMEII0911	Bme	COG0076	Glutamate decarboxylase and related PLP-dependent proteins	Amino acid transport and metabolism	E
BrucellaGL000757	BMEII0913	Bme	COG4803	Predicted membrane protein	Function unknown	S

BrucellaGL000758	SMa1664	Sme	COG0845	Membrane-fusion protein	Cell wall/membrane/envelope biogenesis	M
BrucellaGL000759	BMEII0916	Bme	COG0841	Cation/multidrug efflux pump	Defense mechanisms	V
BrucellaGL000762	BMEII0920	Bme	COG1177	ABC-type spermidine/putrescine transport system, permease component II	Amino acid transport and metabolism	E
BrucellaGL000763	BMEII0921	Bme	COG1176	ABC-type spermidine/putrescine transport system, permease component I	Amino acid transport and metabolism	E
BrucellaGL000764	BMEII0922	Bme	COG3842	ABC-type spermidine/putrescine transport systems, ATPase components	Amino acid transport and metabolism	E
BrucellaGL000765	BMEII0923	Bme	COG0687	Spermidine/putrescine-binding periplasmic protein	Amino acid transport and metabolism	E
BrucellaGL000768	BMEII0925	Bme	COG0851	Septum formation topological specificity factor	Cell cycle control, cell division, chromosome partitioning	D
BrucellaGL000769	BMEII0926	Bme	COG2894	Septum formation inhibitor-activating ATPase	Cell cycle control, cell division, chromosome partitioning	D
BrucellaGL000770	BMEII0927	Bme	COG0850	Septum formation inhibitor	Cell cycle control, cell division, chromosome partitioning	D
BrucellaGL000771	BMEII0929	Bme	COG0208	Ribonucleotide reductase, beta subunit	Nucleotide transport and metabolism	F
BrucellaGL000772	BMEII0930	Bme	COG0209	Ribonucleotide reductase, alpha subunit	Nucleotide transport and metabolism	F
BrucellaGL000773	BMEII0931	Bme	COG1780	Protein involved in ribonucleotide reduction	Nucleotide transport and metabolism	F
BrucellaGL000774	BMEII0932	Bme	COG0695	Glutaredoxin and related proteins	Posttranslational modification, protein turnover, chaperones	O
BrucellaGL000775	AGc139	Atu	COG0477	Permeases of the major facilitator superfamily	Carbohydrate transport and metabolism	G
BrucellaGL000776	AGc139	Atu	COG0477	Permeases of the major facilitator superfamily	Carbohydrate transport and metabolism	G
BrucellaGL000779	SMb20330	Sme	COG0673	Predicted dehydrogenases and related proteins	General function prediction only	R
BrucellaGL000780	BMEII0939	Bme	COG4813	Trehalose utilization protein	Carbohydrate transport and metabolism	G
BrucellaGL000781	AGc1052	Atu	COG3839	ABC-type sugar transport systems, ATPase components	Carbohydrate transport and metabolism	G
BrucellaGL000782	SMb20327	Sme	COG0395	ABC-type sugar transport system, permease component	Carbohydrate transport and metabolism	G

BrucellaGL000783	SMb20326	Sme	COG1175	ABC-type sugar transport systems, permease components	Carbohydrate transport and metabolism	G
BrucellaGL000784	SMb20325	Sme	COG1653	ABC-type sugar transport system, periplasmic component	Carbohydrate transport and metabolism	G
BrucellaGL000785	BMEII0946	Bme	COG1609	Transcriptional regulators	Transcription	K
BrucellaGL000786	BMEII0947	Bme	COG0664	cAMP-binding proteins - catabolite gene activator and regulatory subunit of cAMP-dependent protein kinases	Signal transduction mechanisms	T
BrucellaGL000787	BMEII0948	Bme	COG2223	Nitrate/nitrite transporter	Inorganic ion transport and metabolism	P
BrucellaGL000788	BMEII0950	Bme	COG5013	Nitrate reductase alpha subunit	Energy production and conversion	C
BrucellaGL000789	BMEII0951	Bme	COG1140	Nitrate reductase beta subunit	Energy production and conversion	C
BrucellaGL000790	BMEII0952	Bme	COG2180	Nitrate reductase delta subunit	Energy production and conversion	C
BrucellaGL000791	BMEII0953	Bme	COG2181	Nitrate reductase gamma subunit	Energy production and conversion	C
BrucellaGL000792	BMEII0954	Bme	COG0760	Parvulin-like peptidyl-prolyl isomerase	Posttranslational modification, protein turnover, chaperones	O
BrucellaGL000794	BMEII0956	Bme	COG0163	3-polyprenyl-4-hydroxybenzoate decarboxylase	Coenzyme transport and metabolism	H
BrucellaGL000795	BMEII0957	Bme	COG0043	3-polyprenyl-4-hydroxybenzoate decarboxylase and related decarboxylases	Coenzyme transport and metabolism	H
BrucellaGL000796	BMEII0958	Bme	COG3154	Putative lipid carrier protein	Lipid transport and metabolism	I
BrucellaGL000797	BMEII0959	Bme	COG0826	Collagenase and related proteases	Posttranslational modification, protein turnover, chaperones	O
BrucellaGL000798	BMEII0960	Bme	COG0826	Collagenase and related proteases	Posttranslational modification, protein turnover, chaperones	O
BrucellaGL000800	BMEII0961	Bme	COG1116	ABC-type nitrate/sulfonate/bicarbonate transport system, ATPase component	Inorganic ion transport and metabolism	P
BrucellaGL000801	BMEII0961	Bme	COG1116	ABC-type nitrate/sulfonate/bicarbonate transport system, ATPase component	Inorganic ion transport and metabolism	P
BrucellaGL000802	BMEII0962	Bme	COG0600	ABC-type nitrate/sulfonate/bicarbonate transport system, permease component	Inorganic ion transport and metabolism	P
BrucellaGL000803	BMEII0963	Bme	COG0715	ABC-type nitrate/sulfonate/bicarbonate transport systems, periplasmic components	Inorganic ion transport and metabolism	P

BrucellaGL000804	BMEII0964	Bme	COG3213	Uncharacterized protein involved in response to NO	Inorganic ion transport and metabolism	P
BrucellaGL000805	BMEII0965	Bme	COG3794	Plastocyanin	Energy production and conversion	C
BrucellaGL000806	BMEII0966	Bme	COG0664	cAMP-binding proteins - catabolite gene activator and regulatory subunit of cAMP-dependent protein kinases	Signal transduction mechanisms	T
BrucellaGL000807	BMEII0967	Bme	COG1477	Membrane-associated lipoprotein involved in thiamine biosynthesis	Coenzyme transport and metabolism	H
BrucellaGL000809	BMEII0969	Bme	COG4314	Predicted lipoprotein involved in nitrous oxide reduction	Energy production and conversion	C
BrucellaGL000810	BMEII0970	Bme	COG1277	ABC-type transport system involved in multi-copper enzyme maturation, permease component	General function prediction only	R
BrucellaGL000811	BMEII0971	Bme	COG1131	ABC-type multidrug transport system, ATPase component	Defense mechanisms	V
BrucellaGL000812	BMEII0972	Bme	COG3420	Nitrous oxidase accessory protein	Inorganic ion transport and metabolism	P
BrucellaGL000813	BMEII0973	Bme	COG4263	Nitrous oxide reductase	Energy production and conversion	C
BrucellaGL000814	BMEII0974	Bme	COG4263	Nitrous oxide reductase	Energy production and conversion	C
BrucellaGL000815	BMEII0975_1	Bme	COG3901	Regulator of nitric oxide reductase transcription	Transcription	K
BrucellaGL000818	BMEII0976	Bme	COG4178	ABC-type uncharacterized transport system, permease and ATPase components	General function prediction only	R
BrucellaGL000821	BMEII0979	Bme	COG1069	Ribulose kinase	Energy production and conversion	C
BrucellaGL000822	BMEII0980	Bme	COG4221	Short-chain alcohol dehydrogenase of unknown specificity	General function prediction only	R
BrucellaGL000824	BMEII0981	Bme	COG4214	ABC-type xylose transport system, permease component	Carbohydrate transport and metabolism	G
BrucellaGL000825	mll1002	Mlo	COG1129	ABC-type sugar transport system, ATPase component	Carbohydrate transport and metabolism	G
BrucellaGL000826	BMEII0983	Bme	COG4213	ABC-type xylose transport system, periplasmic component	Carbohydrate transport and metabolism	G
BrucellaGL000827	BMEII0985	Bme	COG1609	Transcriptional regulators	Transcription	K

BrucellaGL000828	BMEII0986	Bme	COG0664	cAMP-binding proteins - catabolite gene activator and regulatory subunit of cAMP-dependent protein kinases	Signal transduction mechanisms	T
BrucellaGL000829	BMEII0987	Bme	COG1262	Uncharacterized conserved protein	Function unknown	S
BrucellaGL000830	BMEII0988	Bme	COG2132	Putative multicopper oxidases	Secondary metabolites biosynthesis, transport and catabolism	Q
BrucellaGL000831	BMEII0989	Bme	COG3319	Thioesterase domains of type I polyketide synthases or non-ribosomal peptide synthetases	Secondary metabolites biosynthesis, transport and catabolism	Q
BrucellaGL000832	BMEII0990	Bme	COG2020	Putative protein-S-isoprenylcysteine methyltransferase	Posttranslational modification, protein turnover, chaperones	O
BrucellaGL000834	BMEII0992	Bme	COG2151	Predicted metal-sulfur cluster biosynthetic enzyme	General function prediction only	R
BrucellaGL000837	BMEII0995	Bme	COG4309	Uncharacterized conserved protein	Function unknown	S
BrucellaGL000838	BMEII0996	Bme	COG4548	Nitric oxide reductase activation protein	Inorganic ion transport and metabolism	P
BrucellaGL000839	BMEII0997	Bme	COG0714	MoxR-like ATPases	General function prediction only	R
BrucellaGL000840	BMEII0998	Bme	COG3256	Nitric oxide reductase large subunit	Inorganic ion transport and metabolism	P
BrucellaGL000841	BMEII0999	Bme	COG2010	Cytochrome c, mono- and diheme variants	Energy production and conversion	C
BrucellaGL000843	BMEII1001	Bme	COG1845	Heme/copper-type cytochrome/quinol oxidase, subunit 3	Energy production and conversion	C
BrucellaGL000846	BMEII1003	Bme	COG2244	Membrane protein involved in the export of O-antigen and teichoic acid	General function prediction only	R
BrucellaGL000847	BMEII1005	Bme	COG2055	Malate/L-lactate dehydrogenases	Energy production and conversion	C
BrucellaGL000848	BMEII1006	Bme	COG0329	Dihydrodipicolinate synthase/N-acetylneuraminate lyase	Amino acid transport and metabolism	E
BrucellaGL000849	PA4185	Pae	COG1802	Transcriptional regulators	Transcription	K
BrucellaGL000850	VC0337	Vch	COG0697	Permeases of the drug/metabolite transporter (DMT) superfamily	Carbohydrate transport and metabolism	G
BrucellaGL000852	BMEII1009_1	Bme	COG2203	FOG: GAF domain	Signal transduction mechanisms	T
BrucellaGL000853	BMEII1010	Bme	COG1477	Membrane-associated lipoprotein involved in thiamine biosynthesis	Coenzyme transport and metabolism	H

BrucellaGL000854	BMEII1011	Bme	COG0369	Sulfite reductase, alpha subunit (flavoprotein)	Inorganic ion transport and metabolism	P
BrucellaGL000856	BMEII1013	Bme	COG5591	Uncharacterized conserved protein	Function unknown	S
BrucellaGL000857	HI1708	Hin	COG0745	Response regulators consisting of a CheY-like receiver domain and a winged-helix DNA-binding domain	Signal transduction mechanisms	T
BrucellaGL000858	HI1707	Hin	COG0642	Signal transduction histidine kinase	Signal transduction mechanisms	T
BrucellaGL000859	BMEII1016	Bme	COG0693	Putative intracellular protease/amidase	General function prediction only	R
BrucellaGL000860	BMEII1017	Bme	COG0789	Predicted transcriptional regulators	Transcription	K
BrucellaGL000861	BMEII1018	Bme	COG1902	NADH:flavin oxidoreductases, Old Yellow Enzyme family	Energy production and conversion	C
BrucellaGL000862	BMEII1019	Bme	COG1804	Predicted acyl-CoA transferases/carnitine dehydratase	Energy production and conversion	C
BrucellaGL000863	BMEII1020	Bme	COG1250	3-hydroxyacyl-CoA dehydrogenase	Lipid transport and metabolism	I
BrucellaGL000864	PA1629	Pae	COG1024	Enoyl-CoA hydratase/carnithine racemase	Lipid transport and metabolism	I
BrucellaGL000865	BMEII1022	Bme	COG1414	Transcriptional regulator	Transcription	K
BrucellaGL000866	BMEII0213	Bme	COG1960	Acyl-CoA dehydrogenases	Lipid transport and metabolism	I
BrucellaGL000867	HI1104	Hin	COG0477	Permeases of the major facilitator superfamily	Carbohydrate transport and metabolism	G
BrucellaGL000868	BMEII1026	Bme	COG0323	DNA mismatch repair enzyme (predicted ATPase)	Replication, recombination and repair	L
BrucellaGL000869	BMEII1027	Bme	COG3908	Uncharacterized protein conserved in bacteria	Function unknown	S
BrucellaGL000870	BMEII1028	Bme	COG1663	Tetraacyldisaccharide-1-P 4'-kinase	Cell wall/membrane/envelope biogenesis	M
BrucellaGL000871	BMEII1029	Bme	COG1519	3-deoxy-D-manno-octulosonic-acid transferase	Cell wall/membrane/envelope biogenesis	M
BrucellaGL000872	BMEII1030	Bme	COG2121	Uncharacterized protein conserved in bacteria	Function unknown	S
BrucellaGL000874	BMEII1032	Bme	COG0483	Archaeal fructose-1,6-bisphosphatase and related enzymes of inositol monophosphatase family	Carbohydrate transport and metabolism	G
BrucellaGL000875	BMEII1033	Bme	COG0312	Predicted Zn-dependent proteases and their inactivated homologs	General function prediction only	R
BrucellaGL000876	BMEII1034_1	Bme	COG0475	Kef-type K+ transport systems, membrane components	Inorganic ion transport and metabolism	P

BrucellaGL000877	BMEII1035	Bme	COG1752	Predicted esterase of the alpha-beta hydrolase superfamily	General function prediction only	R
BrucellaGL000878	BMEII1036	Bme	COG0612	Predicted Zn-dependent peptidases	General function prediction only	R
BrucellaGL000879	BMEII1037	Bme	COG0612	Predicted Zn-dependent peptidases	General function prediction only	R
BrucellaGL000880	BMEII1038	Bme	COG0742	N6-adenine-specific methylase	Replication, recombination and repair	L
BrucellaGL000881	BMEII1039	Bme	COG1187	16S rRNA uridine-516 pseudouridylate synthase and related pseudouridylate synthases	Translation, ribosomal structure and biogenesis	J
BrucellaGL000882	BMEII1040	Bme	COG0590	Cytosine/adenosine deaminases	Nucleotide transport and metabolism	F
BrucellaGL000883	BMEII1041	Bme	COG3038	Cytochrome B561	Energy production and conversion	C
BrucellaGL000885	BMEII1043	Bme	COG0060	Isoleucyl-tRNA synthetase	Translation, ribosomal structure and biogenesis	J
BrucellaGL000886	BMEII1044	Bme	COG0196	FAD synthase	Coenzyme transport and metabolism	H
BrucellaGL000887	BMEII1045	Bme	COG0647	Predicted sugar phosphatases of the HAD superfamily	Carbohydrate transport and metabolism	G
BrucellaGL000889	BMEII1047	Bme	COG0234	Co-chaperonin GroES (HSP10)	Posttranslational modification, protein turnover, chaperones	O
BrucellaGL000890	BMEII1048	Bme	COG0459	Chaperonin GroEL (HSP60 family)	Posttranslational modification, protein turnover, chaperones	O
BrucellaGL000892	BMEII1051	Bme	COG0114	Fumarase	Energy production and conversion	C
BrucellaGL000893	BMEII1052	Bme	COG2259	Predicted membrane protein	Function unknown	S
BrucellaGL000894	BMEII1053	Bme	COG0738	Fucose permease	Carbohydrate transport and metabolism	G
BrucellaGL000895	BMEII1054	Bme	COG0040	ATP phosphoribosyltransferase	Amino acid transport and metabolism	E
BrucellaGL000896	BMEII1055	Bme	COG3705	ATP phosphoribosyltransferase involved in histidine biosynthesis	Amino acid transport and metabolism	E
BrucellaGL000897	BMEII1056	Bme	COG0124	Histidyl-tRNA synthetase	Translation, ribosomal structure and biogenesis	J
BrucellaGL000898	BMEII1057	Bme	COG1280	Putative threonine efflux protein	Amino acid transport and metabolism	E
BrucellaGL000899	BMEII1058	Bme	COG2818	3-methyladenine DNA glycosylase	Replication, recombination and repair	L
BrucellaGL000900	BMEII1059	Bme	COG1376	Uncharacterized protein conserved in bacteria	Function unknown	S
BrucellaGL000902	BMEII1060	Bme	COG0656	Aldo/keto reductases, related to diketogulonate reductase	General function prediction only	R

BrucellaGL000903	BMEII1061	Bme	COG0247	Fe-S oxidoreductase	Energy production and conversion	C
BrucellaGL000904	BMEII1062	Bme	COG0277	FAD/FMN-containing dehydrogenases	Energy production and conversion	C
BrucellaGL000905	BMEII1064	Bme	COG0277	FAD/FMN-containing dehydrogenases	Energy production and conversion	C
BrucellaGL000907	AGc1186	Atu	COG0583	Transcriptional regulator	Transcription	K
BrucellaGL000908	BMEII1066	Bme	COG2186	Transcriptional regulators	Transcription	K
BrucellaGL000909	BMEII1067	Bme	COG2841	Uncharacterized protein conserved in bacteria	Function unknown	S
BrucellaGL000910	BMEII1068	Bme	COG3474	Cytochrome c2	Energy production and conversion	C
BrucellaGL000911	BMEII1069	Bme	COG3468	Type V secretory pathway, adhesin AidA	Cell wall/membrane/envelope biogenesis	M
BrucellaGL000912	BMEII0148	Bme	COG3468	Type V secretory pathway, adhesin AidA	Cell wall/membrane/envelope biogenesis	M
BrucellaGL000913	BMEII0148	Bme	COG3468	Type V secretory pathway, adhesin AidA	Cell wall/membrane/envelope biogenesis	M
BrucellaGL000914	BMEII1073	Bme	COG3038	Cytochrome B561	Energy production and conversion	C
BrucellaGL000917	BMEII1074	Bme	COG2301	Citrate lyase beta subunit	Carbohydrate transport and metabolism	G
BrucellaGL000918	BMEII1075	Bme	COG3777	Uncharacterized conserved protein	Function unknown	S
BrucellaGL000919	BMEII1076	Bme	COG1804	Predicted acyl-CoA transferases/carnitine dehydratase	Energy production and conversion	C
BrucellaGL000920	cynR	Eco	COG0583	Transcriptional regulator	Transcription	K
BrucellaGL000921	BMEII1078	Bme	COG4893	Uncharacterized protein conserved in bacteria	Function unknown	S
BrucellaGL000923	BMEII1080	Bme	COG1338	Flagellar biosynthesis pathway, component FliP	Cell motility	N
BrucellaGL000925	BMEII1082	Bme	COG2063	Flagellar basal body L-ring protein	Cell motility	N
BrucellaGL000926	BMEII1083	Bme	COG3334	Uncharacterized conserved protein	Function unknown	S
BrucellaGL000927	BMEII1084	Bme	COG1706	Flagellar basal-body P-ring protein	Cell motility	N
BrucellaGL000928	BMEII1085	Bme	COG1261	Flagellar basal body P-ring biosynthesis protein	Cell motility	N
BrucellaGL000929	BMEII1086	Bme	COG4786	Flagellar basal body rod protein	Cell motility	N
BrucellaGL000930	BMEII1087	Bme	COG1677	Flagellar hook-basal body protein	Cell motility	N
BrucellaGL000931	BMEII1088	Bme	COG1558	Flagellar basal body rod protein	Cell motility	N
BrucellaGL000932	BMEII1089	Bme	COG1815	Flagellar basal body protein	Cell motility	N
BrucellaGL000933	BMEII1090	Bme	COG2084	3-hydroxyisobutyrate dehydrogenase and related beta-hydroxyacid dehydrogenases	Lipid transport and metabolism	I

BrucellaGL000934	BMEII1091	Bme	COG3395	Uncharacterized protein conserved in bacteria	Function unknown	S
BrucellaGL000935	BMEII1092	Bme	COG3622	Hydroxypyruvate isomerase	Carbohydrate transport and metabolism	G
BrucellaGL000936	BMEII1093	Bme	COG1349	Transcriptional regulators of sugar metabolism	Transcription	K
BrucellaGL000937	YPO2238	Ype	COG0451	Nucleoside-diphosphate-sugar epimerases	Cell wall/membrane/envelope biogenesis	M
BrucellaGL000938	BMEII1095	Bme	COG0235	Ribulose-5-phosphate 4-epimerase and related epimerases and aldolases	Carbohydrate transport and metabolism	G
BrucellaGL000939	Cj0484	Cje	COG0477	Permeases of the major facilitator superfamily	Carbohydrate transport and metabolism	G
BrucellaGL000940	BMEI1384	Bme	COG2207	AraC-type DNA-binding domain-containing proteins	Transcription	K
BrucellaGL000941	BMEII1099	Bme	COG1807	4-amino-4-deoxy-L-arabinose transferase and related glycosyltransferases of PMT family	Cell wall/membrane/envelope biogenesis	M
BrucellaGL000942	BMEII1099	Bme	COG1807	4-amino-4-deoxy-L-arabinose transferase and related glycosyltransferases of PMT family	Cell wall/membrane/envelope biogenesis	M
BrucellaGL000943	BMEII1100	Bme	COG3394	Uncharacterized protein conserved in bacteria	Function unknown	S
BrucellaGL000944	BMEII1101	Bme	COG0463	Glycosyltransferases involved in cell wall biogenesis	Cell wall/membrane/envelope biogenesis	M
BrucellaGL000946	BMEII1103	Bme	COG0671	Membrane-associated phospholipid phosphatase	Lipid transport and metabolism	I
BrucellaGL000948	BMEII1105	Bme	COG1157	Flagellar biosynthesis/type III secretory pathway ATPase	Cell motility	N
BrucellaGL000949	BMEII1107	Bme	COG4786	Flagellar basal body rod protein	Cell motility	N
BrucellaGL000951	BMEII1109	Bme	COG1291	Flagellar motor component	Cell motility	N
BrucellaGL000952	BMEII1110	Bme	COG1868	Flagellar motor switch protein	Cell motility	N
BrucellaGL000954	BMEII1112	Bme	COG1886	Flagellar motor switch/type III secretory pathway protein	Cell motility	N
BrucellaGL000955	BMEII1113	Bme	COG1536	Flagellar motor switch protein	Cell motility	N
BrucellaGL000956	BMEII1114	Bme	COG1377	Flagellar biosynthesis pathway, component FlhB	Cell motility	N
BrucellaGL000958	BMEII1116	Bme	COG2771	DNA-binding HTH domain-containing proteins	Transcription	K
BrucellaGL000959	VC1408	Vch	COG1309	Transcriptional regulator	Transcription	K
BrucellaGL000960	BMEII1118	Bme	COG1566	Multidrug resistance efflux pump	Defense mechanisms	V

BrucellaGL000961	RC0200	Rco	COG0477	Permeases of the major facilitator superfamily	Carbohydrate transport and metabolism	G
BrucellaGL000962	BMEII1120	Bme	COG1840	ABC-type Fe3+ transport system, periplasmic component	Inorganic ion transport and metabolism	P
BrucellaGL000964	BMEII1121	Bme	COG4132	ABC-type uncharacterized transport system, permease component	General function prediction only	R
BrucellaGL000965	BMEII1122	Bme	COG1177	ABC-type spermidine/putrescine transport system, permease component II	Amino acid transport and metabolism	E
BrucellaGL000966	BMEII1123	Bme	COG3842	ABC-type spermidine/putrescine transport systems, ATPase components	Amino acid transport and metabolism	E
BrucellaGL000967	BMEII1124	Bme	COG0362	6-phosphogluconate dehydrogenase	Carbohydrate transport and metabolism	G
BrucellaGL000968	CAC1480	Cac	COG0673	Predicted dehydrogenases and related proteins	General function prediction only	R
BrucellaGL000970	BH0708	Bha	COG0673	Predicted dehydrogenases and related proteins	General function prediction only	R
BrucellaGL000971	BMEII1127	Bme	COG3307	Lipid A core - O-antigen ligase and related enzymes	Cell wall/membrane/envelope biogenesis	M
BrucellaGL000972	BMEII1128	Bme	COG0463	Glycosyltransferases involved in cell wall biogenesis	Cell wall/membrane/envelope biogenesis	M
BrucellaGL000974	MA1061	Mac	COG0438	Glycosyltransferase	Cell wall/membrane/envelope biogenesis	M
BrucellaGL000975	BMEII1130	Bme	COG1922	Teichoic acid biosynthesis proteins	Cell wall/membrane/envelope biogenesis	M
BrucellaGL000977	BMEII1132	Bme	COG3206	Uncharacterized protein involved in exopolysaccharide biosynthesis	Cell wall/membrane/envelope biogenesis	M
BrucellaGL000978	BMEII1133	Bme	COG0019	Diaminopimelate decarboxylase	Amino acid transport and metabolism	E
BrucellaGL000979	BMEII1134	Bme	COG0154	Asp-tRNAAsn/Glu-tRNAGln amidotransferase A subunit and related amidases	Translation, ribosomal structure and biogenesis	J
BrucellaGL000980	CC2517	Ccr	COG0583	Transcriptional regulator	Transcription	K
BrucellaGL000981	BMEII1136	Bme	COG3683	ABC-type uncharacterized transport system, periplasmic component	General function prediction only	R
BrucellaGL000982	BMEII1137	Bme	COG2215	ABC-type uncharacterized transport system, permease component	General function prediction only	R

BrucellaGL000985	BMEII0002	Bme	COG1670	Acetyltransferases, including N-acetylases of ribosomal proteins	Translation, ribosomal structure and biogenesis	J
BrucellaGL000986	BMEII0003	Bme	COG4148	ABC-type molybdate transport system, ATPase component	Inorganic ion transport and metabolism	P
BrucellaGL000987	BMEII0004	Bme	COG4149	ABC-type molybdate transport system, permease component	Inorganic ion transport and metabolism	P
BrucellaGL000988	BMEII0005	Bme	COG0725	ABC-type molybdate transport system, periplasmic component	Inorganic ion transport and metabolism	P
BrucellaGL000989	BMEII0007	Bme	COG1054	Predicted sulfurtransferase	General function prediction only	R
BrucellaGL000991	BMEII0009	Bme	COG0800	2-keto-3-deoxy-6-phosphogluconate aldolase	Carbohydrate transport and metabolism	G
BrucellaGL000992	BMEII0010_1	Bme	COG3108	Uncharacterized protein conserved in bacteria	Function unknown	S
BrucellaGL000993	BMEII0011	Bme	COG2204	Response regulator containing CheY-like receiver, AAA-type ATPase, and DNA-binding domains	Signal transduction mechanisms	T
BrucellaGL000994	BMEII0012	Bme	COG1164	Oligoendopeptidase F	Amino acid transport and metabolism	E
BrucellaGL000995	BMEII0013	Bme	COG0147	Anthranilate/para-aminobenzoate synthases component I	Amino acid transport and metabolism	E
BrucellaGL000996	BMEII0014	Bme	COG0115	Branched-chain amino acid aminotransferase/4-amino-4-deoxychorismate lyase	Amino acid transport and metabolism	E
BrucellaGL000997	BMEII0015	Bme	COG5310	Homospermidine synthase	Secondary metabolites biosynthesis, transport and catabolism	Q
BrucellaGL000998	mlr3014	Mlo	COG5310	Homospermidine synthase	Secondary metabolites biosynthesis, transport and catabolism	Q
BrucellaGL001000	BMEII0018	Bme	COG0276	Protoheme ferro-lyase (ferrochelatase)	Coenzyme transport and metabolism	H
BrucellaGL001001	BMEII0019	Bme	COG0330	Membrane protease subunits, stomatin/prohibitin homologs	Posttranslational modification, protein turnover, chaperones	O
BrucellaGL001002	BMEII0020	Bme	COG1585	Membrane protein implicated in regulation of membrane protease activity	Posttranslational modification, protein turnover, chaperones	O
BrucellaGL001003	BMEII0021_1	Bme	COG0794	Predicted sugar phosphate isomerase involved in capsule formation	Cell wall/membrane/envelope biogenesis	M
BrucellaGL001004	BMEII0022	Bme	COG5338	Uncharacterized protein conserved in bacteria	Function unknown	S

BrucellaGL001005	BMEII0023	Bme	COG1210	UDP-glucose pyrophosphorylase	Cell wall/membrane/envelope biogenesis	M
BrucellaGL001006	BMEII0024	Bme	COG2951	Membrane-bound lytic murein transglycosylase B	Cell wall/membrane/envelope biogenesis	M
BrucellaGL001009	BMEII0027	Bme	COG3702	Type IV secretory pathway, VirB3 components	Intracellular trafficking, secretion, and vesicular transport	U
BrucellaGL001010	BMEII0028	Bme	COG3451	Type IV secretory pathway, VirB4 components	Intracellular trafficking, secretion, and vesicular transport	U
BrucellaGL001012	BMEII0030	Bme	COG3704	Type IV secretory pathway, VirB6 components	Intracellular trafficking, secretion, and vesicular transport	U
BrucellaGL001013	BMEII0032	Bme	COG3736	Type IV secretory pathway, component VirB8	Intracellular trafficking, secretion, and vesicular transport	U
BrucellaGL001014	BMEII0033	Bme	COG3504	Type IV secretory pathway, VirB9 components	Intracellular trafficking, secretion, and vesicular transport	U
BrucellaGL001015	BMEII0034	Bme	COG2948	Type IV secretory pathway, VirB10 components	Intracellular trafficking, secretion, and vesicular transport	U
BrucellaGL001016	BMEII0035	Bme	COG0630	Type IV secretory pathway, VirB11 components, and related ATPases involved in archaeal flagella biosynthesis	Cell motility	N
BrucellaGL001018	BMEII0037	Bme	COG2845	Uncharacterized protein conserved in bacteria	Function unknown	S
BrucellaGL001019	BMEII0038	Bme	COG1113	Gamma-aminobutyrate permease and related permeases	Amino acid transport and metabolism	E
BrucellaGL001020	BMEII0039	Bme	COG0493	NADPH-dependent glutamate synthase beta chain and related oxidoreductases	Amino acid transport and metabolism	E
BrucellaGL001021	BMEII0040_2	Bme	COG0069	Glutamate synthase domain 2	Amino acid transport and metabolism	E
BrucellaGL001022	BMEII0041	Bme	COG2008	Threonine aldolase	Amino acid transport and metabolism	E
BrucellaGL001023	BMEII0042	Bme	COG0071	Molecular chaperone (small heat shock protein)	Posttranslational modification, protein turnover, chaperones	O
BrucellaGL001026	BMEII0047	Bme	COG2267	Lysophospholipase	Lipid transport and metabolism	I
BrucellaGL001027	BMEII0048	Bme	COG0483	Archaeal fructose-1,6-bisphosphatase and related enzymes of inositol monophosphatase family	Carbohydrate transport and metabolism	G
BrucellaGL001028	BMEII0049	Bme	COG3741	N-formylglutamate amidohydrolase	Amino acid transport and metabolism	E
BrucellaGL001029	BMEII0050	Bme	COG0784	FOG: CheY-like receiver	Signal transduction mechanisms	T

BrucellaGL001030	BMEII0051	Bme	COG4566	Response regulator	Signal transduction mechanisms	T
BrucellaGL001032	mll0989_2	Mlo	COG0642	Signal transduction histidine kinase	Signal transduction mechanisms	T
BrucellaGL001034	BMEII0053	Bme	COG1285	Uncharacterized membrane protein	Function unknown	S
BrucellaGL001036	BMEII0056	Bme	COG0474	Cation transport ATPase	Inorganic ion transport and metabolism	P
BrucellaGL001038	BMEII0059	Bme	COG0169	Shikimate 5-dehydrogenase	Amino acid transport and metabolism	E
BrucellaGL001039	BMEII0060	Bme	COG0508	Pyruvate/2-oxoglutarate dehydrogenase complex, dihydrolipoamide acyltransferase (E2) component, and related enzymes	Energy production and conversion	C
BrucellaGL001040	BMEII0061_2	Bme	COG0022	Pyruvate/2-oxoglutarate dehydrogenase complex, dehydrogenase (E1) component, eukaryotic type, beta subunit	Energy production and conversion	C
BrucellaGL001041	BMEII0061_1	Bme	COG1071	Pyruvate/2-oxoglutarate dehydrogenase complex, dehydrogenase (E1) component, eukaryotic type, alpha subunit	Energy production and conversion	C
BrucellaGL001043	L1530	Lla	COG1028	Dehydrogenases with different specificities (related to short-chain alcohol dehydrogenases)	Lipid transport and metabolism	I
BrucellaGL001044	TM1724	Tma	COG1028	Dehydrogenases with different specificities (related to short-chain alcohol dehydrogenases)	Lipid transport and metabolism	I
BrucellaGL001045	BMEII0064	Bme	COG0346	Lactoylglutathione lyase and related lyases	Amino acid transport and metabolism	E
BrucellaGL001046	BMEII0065	Bme	COG0410	ABC-type branched-chain amino acid transport systems, ATPase component	Amino acid transport and metabolism	E
BrucellaGL001047	BMEII0066	Bme	COG0411	ABC-type branched-chain amino acid transport systems, ATPase component	Amino acid transport and metabolism	E
BrucellaGL001048	BMEII0067	Bme	COG4177	ABC-type branched-chain amino acid transport system, permease component	Amino acid transport and metabolism	E
BrucellaGL001049	BMEII0068	Bme	COG0559	Branched-chain amino acid ABC-type transport system, permease components	Amino acid transport and metabolism	E
BrucellaGL001050	BMEII0069	Bme	COG0683	ABC-type branched-chain amino acid transport systems, periplasmic component	Amino acid transport and metabolism	E
BrucellaGL001051	BMEII0071	Bme	COG2186	Transcriptional regulators	Transcription	K
BrucellaGL001052	BMEII0072_2	Bme	COG4944	Uncharacterized protein conserved in bacteria	Function unknown	S

BrucellaGL001053	BMEII0072_1	Bme	COG1595	DNA-directed RNA polymerase specialized sigma subunit, sigma24 homolog	Transcription	K
BrucellaGL001055	BMEII0074	Bme	COG4117	Thiosulfate reductase cytochrome B subunit (membrane anchoring protein)	Energy production and conversion	C
BrucellaGL001056	BMEII0075	Bme	COG2041	Sulfite oxidase and related enzymes	General function prediction only	R
BrucellaGL001057	BMEII0076	Bme	COG1020	Non-ribosomal peptide synthetase modules and related proteins	Secondary metabolites biosynthesis, transport and catabolism	Q
BrucellaGL001058	BMEII0077	Bme	COG1169	Isochorismate synthase	Coenzyme transport and metabolism	H
BrucellaGL001059	BMEII0078	Bme	COG1021	Peptide arylation enzymes	Secondary metabolites biosynthesis, transport and catabolism	Q
BrucellaGL001060	BMEII0079_1	Bme	COG1535	Isochorismate hydrolase	Secondary metabolites biosynthesis, transport and catabolism	Q
BrucellaGL001061	ZentA	EcZ	COG1028	Dehydrogenases with different specificities (related to short-chain alcohol dehydrogenases)	Lipid transport and metabolism	I
BrucellaGL001062	BMEII0081	Bme	COG2977	Phosphopantetheinyl transferase component of siderophore synthetase	Secondary metabolites biosynthesis, transport and catabolism	Q
BrucellaGL001063	BMEII0082	Bme	COG5664	Predicted secreted Zn-dependent protease	Posttranslational modification, protein turnover, chaperones	O
BrucellaGL001064	BMEII0083	Bme	COG1744	Uncharacterized ABC-type transport system, periplasmic component/surface lipoprotein	General function prediction only	R
BrucellaGL001065	BMEII0084	Bme	COG1744	Uncharacterized ABC-type transport system, periplasmic component/surface lipoprotein	General function prediction only	R
BrucellaGL001066	BMEII0085	Bme	COG3845	ABC-type uncharacterized transport systems, ATPase components	General function prediction only	R
BrucellaGL001067	BMEII0086	Bme	COG4603	ABC-type uncharacterized transport system, permease component	General function prediction only	R
BrucellaGL001068	BMEII0087	Bme	COG1079	Uncharacterized ABC-type transport system, permease component	General function prediction only	R
BrucellaGL001069	BMEII0088	Bme	COG1957	Inosine-uridine nucleoside N-ribohydrolase	Nucleotide transport and metabolism	F
BrucellaGL001070	BMEII0089	Bme	COG0524	Sugar kinases, ribokinase family	Carbohydrate transport and metabolism	G
BrucellaGL001075	BMEII0092	Bme	COG1475	Predicted transcriptional regulators	Transcription	K

BrucellaGL001076	BMEII0093	Bme	COG1192	ATPases involved in chromosome partitioning	Cell cycle control, cell division, chromosome partitioning	D
BrucellaGL001079	BMEII0096	Bme	COG0635	Coproporphyrinogen III oxidase and related Fe-S oxidoreductases	Coenzyme transport and metabolism	H
BrucellaGL001080	BMEII0097	Bme	COG2217	Cation transport ATPase	Inorganic ion transport and metabolism	P
BrucellaGL001081	BMEII0098	Bme	COG0410	ABC-type branched-chain amino acid transport systems, ATPase component	Amino acid transport and metabolism	E
BrucellaGL001082	BMEII0099	Bme	COG0411	ABC-type branched-chain amino acid transport systems, ATPase component	Amino acid transport and metabolism	E
BrucellaGL001083	BMEII0100	Bme	COG0411	ABC-type branched-chain amino acid transport systems, ATPase component	Amino acid transport and metabolism	E
BrucellaGL001084	BMEII0101	Bme	COG4177	ABC-type branched-chain amino acid transport system, permease component	Amino acid transport and metabolism	E
BrucellaGL001085	BMEII0102	Bme	COG0559	Branched-chain amino acid ABC-type transport system, permease components	Amino acid transport and metabolism	E
BrucellaGL001086	PAE1392	Pya	COG0683	ABC-type branched-chain amino acid transport systems, periplasmic component	Amino acid transport and metabolism	E
BrucellaGL001087	BMEII0104	Bme	COG2207	AraC-type DNA-binding domain-containing proteins	Transcription	K
BrucellaGL001088	BMEII0105	Bme	COG1629	Outer membrane receptor proteins, mostly Fe transport	Inorganic ion transport and metabolism	P
BrucellaGL001089	BMEII0106	Bme	COG1940	Transcriptional regulator/sugar kinase	Transcription	K
BrucellaGL001090	BMEII0107	Bme	COG0600	ABC-type nitrate/sulfonate/bicarbonate transport system, permease component	Inorganic ion transport and metabolism	P
BrucellaGL001091	BMEII0108	Bme	COG1116	ABC-type nitrate/sulfonate/bicarbonate transport system, ATPase component	Inorganic ion transport and metabolism	P
BrucellaGL001092	BMEII0109	Bme	COG0715	ABC-type nitrate/sulfonate/bicarbonate transport systems, periplasmic components	Inorganic ion transport and metabolism	P
BrucellaGL001093	BMEII0110	Bme	COG3119	Arylsulfatase A and related enzymes	Inorganic ion transport and metabolism	P
BrucellaGL001094	BMEII0110	Bme	COG3119	Arylsulfatase A and related enzymes	Inorganic ion transport and metabolism	P
BrucellaGL001095	BMEII0111	Bme	COG1409	Predicted phosphohydrolases	General function prediction only	R

BrucellaGL001096	BMEII0112	Bme	COG3839	ABC-type sugar transport systems, ATPase components	Carbohydrate transport and metabolism	G
BrucellaGL001097	BMEII0113	Bme	COG1175	ABC-type sugar transport systems, permease components	Carbohydrate transport and metabolism	G
BrucellaGL001098	BMEII0114	Bme	COG0395	ABC-type sugar transport system, permease component	Carbohydrate transport and metabolism	G
BrucellaGL001099	BMEII0115	Bme	COG1653	ABC-type sugar transport system, periplasmic component	Carbohydrate transport and metabolism	G
BrucellaGL001100	BMEII0116	Bme	COG2188	Transcriptional regulators	Transcription	K
BrucellaGL001101	BMEII0117	Bme	COG0730	Predicted permeases	General function prediction only	R
BrucellaGL001102	BMEII0117	Bme	COG0730	Predicted permeases	General function prediction only	R
BrucellaGL001104	BMEII0119	Bme	COG0410	ABC-type branched-chain amino acid transport systems, ATPase component	Amino acid transport and metabolism	E
BrucellaGL001105	BMEII0120_1	Bme	COG4177	ABC-type branched-chain amino acid transport system, permease component	Amino acid transport and metabolism	E
BrucellaGL001107	BMEII0121	Bme	COG0559	Branched-chain amino acid ABC-type transport system, permease components	Amino acid transport and metabolism	E
BrucellaGL001109	BMEII0123	Bme	COG1574	Predicted metal-dependent hydrolase with the TIM-barrel fold	General function prediction only	R
BrucellaGL001110	ZaldH	EcZ	COG1012	NAD-dependent aldehyde dehydrogenases	Energy production and conversion	C
BrucellaGL001111	BMEII0126	Bme	COG0531	Amino acid transporters	Amino acid transport and metabolism	E
BrucellaGL001112	BMEII0127	Bme	COG1414	Transcriptional regulator	Transcription	K
BrucellaGL001113	BMEII0128	Bme	COG5441	Uncharacterized conserved protein	Function unknown	S
BrucellaGL001114	BMEII0129	Bme	COG1011	Predicted hydrolase (HAD superfamily)	General function prediction only	R
BrucellaGL001115	BMEII0130	Bme	COG0161	Adenosylmethionine-8-amino-7-oxononanoate aminotransferase	Coenzyme transport and metabolism	H
BrucellaGL001116	BMEII0131	Bme	COG0160	4-aminobutyrate aminotransferase and related aminotransferases	Amino acid transport and metabolism	E
BrucellaGL001117	BMEII0132	Bme	COG2334	Putative homoserine kinase type II (protein kinase fold)	General function prediction only	R
BrucellaGL001118	BMEII0133	Bme	COG1846	Transcriptional regulators	Transcription	K
BrucellaGL001119	BMEII0134	Bme	COG3232	5-carboxymethyl-2-hydroxymuconate isomerase	Amino acid transport and metabolism	E
BrucellaGL001120	DRA0220	Dra	COG1012	NAD-dependent aldehyde dehydrogenases	Energy production and conversion	C

BrucellaGL001121	SSO2054	Sso	COG0346	Lactoylglutathione lyase and related lyases	Amino acid transport and metabolism	E
BrucellaGL001122	BMEII0137	Bme	COG0179	2-keto-4-pentenoate hydratase/2-oxohepta-3-ene-1,7-dioic acid hydratase (catechol pathway)	Secondary metabolites biosynthesis, transport and catabolism	Q
BrucellaGL001123	BMEII0138	Bme	COG3971	2-keto-4-pentenoate hydratase	Secondary metabolites biosynthesis, transport and catabolism	Q
BrucellaGL001124	BMEII0139	Bme	COG3836	2,4-dihydroxyhept-2-ene-1,7-dioic acid aldolase	Carbohydrate transport and metabolism	G
BrucellaGL001125	MA4079	Mac	COG1012	NAD-dependent aldehyde dehydrogenases	Energy production and conversion	C
BrucellaGL001127	SA0162	Sau	COG1012	NAD-dependent aldehyde dehydrogenases	Energy production and conversion	C
BrucellaGL001128	BMEII0143	Bme	COG2207	AraC-type DNA-binding domain-containing proteins	Transcription	K
BrucellaGL001129	BMEII0144	Bme	COG4214	ABC-type xylose transport system, permease component	Carbohydrate transport and metabolism	G
BrucellaGL001130	BMEII0145	Bme	COG1129	ABC-type sugar transport system, ATPase component	Carbohydrate transport and metabolism	G
BrucellaGL001131	BMEII0146	Bme	COG4213	ABC-type xylose transport system, periplasmic component	Carbohydrate transport and metabolism	G
BrucellaGL001132	BMEII0147	Bme	COG3268	Uncharacterized conserved protein	Function unknown	S
BrucellaGL001133	BMEII0148	Bme	COG3468	Type V secretory pathway, adhesin AidA	Cell wall/membrane/envelope biogenesis	M
BrucellaGL001134	BMEII0148	Bme	COG3468	Type V secretory pathway, adhesin AidA	Cell wall/membrane/envelope biogenesis	M
BrucellaGL001135	BMEII0150	Bme	COG1344	Flagellin and related hook-associated proteins	Cell motility	N
BrucellaGL001137	BMEII0151	Bme	COG1766	Flagellar biosynthesis/type III secretory pathway lipoprotein	Cell motility	N
BrucellaGL001139	BMEII0152	Bme	COG1766	Flagellar biosynthesis/type III secretory pathway lipoprotein	Cell motility	N
BrucellaGL001141	BMEII0154	Bme	COG1360	Flagellar motor protein	Cell motility	N
BrucellaGL001144	BMEII0157	Bme	COG0741	Soluble lytic murein transglycosylase and related regulatory proteins (some contain LysM/invasin domains)	Cell wall/membrane/envelope biogenesis	M

BrucellaGL001145	AGc1009	Atu	COG0745	Response regulators consisting of a CheY-like receiver domain and a winged-helix DNA-binding domain	Signal transduction mechanisms	T
BrucellaGL001146	BMEII0159	Bme	COG1749	Flagellar hook protein FlgE	Cell motility	N
BrucellaGL001147	BMEII0160	Bme	COG1256	Flagellar hook-associated protein	Cell motility	N
BrucellaGL001148	BMEII0160	Bme	COG1256	Flagellar hook-associated protein	Cell motility	N
BrucellaGL001149	BMEII0161	Bme	COG1344	Flagellin and related hook-associated proteins	Cell motility	N
BrucellaGL001150	BMEII0162	Bme	COG5442	Flagellar biosynthesis regulator FlaF	Cell motility	N
BrucellaGL001151	BMEII0163	Bme	COG5443	Flagellar biosynthesis regulator FlbT	Cell motility	N
BrucellaGL001152	BMEII0164	Bme	COG1843	Flagellar hook capping protein	Cell motility	N
BrucellaGL001153	BMEII0165	Bme	COG1987	Flagellar biosynthesis pathway, component FliQ	Cell motility	N
BrucellaGL001154	BMEII0166	Bme	COG1298	Flagellar biosynthesis pathway, component FlhA	Cell motility	N
BrucellaGL001155	BMEII0167	Bme	COG1298	Flagellar biosynthesis pathway, component FlhA	Cell motility	N
BrucellaGL001156	BMEII0168	Bme	COG1684	Flagellar biosynthesis pathway, component FliR	Cell motility	N
BrucellaGL001162	BMEII0175	Bme	COG0735	Fe2+/Zn2+ uptake regulation proteins	Inorganic ion transport and metabolism	P
BrucellaGL001163	BMEII0176	Bme	COG1108	ABC-type Mn2+/Zn2+ transport systems, permease components	Inorganic ion transport and metabolism	P
BrucellaGL001164	BMEII0177	Bme	COG1121	ABC-type Mn/Zn transport systems, ATPase component	Inorganic ion transport and metabolism	P
BrucellaGL001165	BMEII0178	Bme	COG4531	ABC-type Zn2+ transport system, periplasmic component/surface adhesin	Inorganic ion transport and metabolism	P
BrucellaGL001166	BMEII0179	Bme	COG0523	Putative GTPases (G3E family)	General function prediction only	R
BrucellaGL001167	BMEII0180	Bme	COG1959	Predicted transcriptional regulator	Transcription	K
BrucellaGL001168	BMEII0181	Bme	COG2971	Predicted N-acetylglucosamine kinase	Carbohydrate transport and metabolism	G
BrucellaGL001172	BMEI0908	Bme	COG1752	Predicted esterase of the alpha-beta hydrolase superfamily	General function prediction only	R
BrucellaGL001173	BMEI0909	Bme	COG0139	Phosphoribosyl-AMP cyclohydrolase	Amino acid transport and metabolism	E
BrucellaGL001174	BMEI0910	Bme	COG0302	GTP cyclohydrolase I	Coenzyme transport and metabolism	H
BrucellaGL001175	BMEI0911	Bme	COG0822	NifU homolog involved in Fe-S cluster formation	Energy production and conversion	C
BrucellaGL001176	BMEI0912	Bme	COG0759	Uncharacterized conserved protein	Function unknown	S

BrucellaGL001177	BMEI0913	Bme	COG1686	D-alanyl-D-alanine carboxypeptidase	Cell wall/membrane/envelope biogenesis	M
BrucellaGL001178	BMEI0914	Bme	COG1686	D-alanyl-D-alanine carboxypeptidase	Cell wall/membrane/envelope biogenesis	M
BrucellaGL001179	BMEI0915	Bme	COG0441	Threonyl-tRNA synthetase	Translation, ribosomal structure and biogenesis	J
BrucellaGL001181	mll0911	Mlo	COG0778	Nitroreductase	Energy production and conversion	C
BrucellaGL001182	BMEI0919	Bme	COG1853	Conserved protein/domain typically associated with flavoprotein oxygenases, DIM6/NTAB family	General function prediction only	R
BrucellaGL001183	BMEI0920	Bme	COG1694	Predicted pyrophosphatase	General function prediction only	R
BrucellaGL001184	BMEI0921	Bme	COG1087	UDP-glucose 4-epimerase	Cell wall/membrane/envelope biogenesis	M
BrucellaGL001185	BMEI0922	Bme	COG0385	Predicted Na+-dependent transporter	General function prediction only	R
BrucellaGL001186	BMEI0923	Bme	COG4976	Predicted methyltransferase (contains TPR repeat)	General function prediction only	R
BrucellaGL001187	BMEI0924	Bme	COG0006	Xaa-Pro aminopeptidase	Amino acid transport and metabolism	E
BrucellaGL001188	BMEI0925	Bme	COG1064	Zn-dependent alcohol dehydrogenases	General function prediction only	R
BrucellaGL001189	BMEI0926	Bme	COG1566	Multidrug resistance efflux pump	Defense mechanisms	V
BrucellaGL001190	emrB	Eco	COG0477	Permeases of the major facilitator superfamily	Carbohydrate transport and metabolism	G
BrucellaGL001191	BMEI0928	Bme	COG0427	Acetyl-CoA hydrolase	Energy production and conversion	C
BrucellaGL001192	BMEI0929	Bme	COG2199	FOG: GGDEF domain	Signal transduction mechanisms	T
BrucellaGL001193	BMEI0930	Bme	COG0454	Histone acetyltransferase HPA2 and related acetyltransferases	Transcription	K
BrucellaGL001194	BMEI0931	Bme	COG2897	Rhodanese-related sulfurtransferase	Inorganic ion transport and metabolism	P
BrucellaGL001195	BMEI0932	Bme	COG2872	Predicted metal-dependent hydrolases related to alanyl-tRNA synthetase HxxxH domain	General function prediction only	R
BrucellaGL001196	BMEI0933	Bme	COG0031	Cysteine synthase	Amino acid transport and metabolism	E
BrucellaGL001197	BMEI0934	Bme	COG0513	Superfamily II DNA and RNA helicases	Replication, recombination and repair	L
BrucellaGL001198	BMEI0935	Bme	COG1171	Threonine dehydratase	Amino acid transport and metabolism	E
BrucellaGL001199	BMEI0936	Bme	COG0655	Multimeric flavodoxin WrbA	General function prediction only	R

BrucellaGL001200	BMEI0937	Bme	COG1058	Predicted nucleotide-utilizing enzyme related to molybdopterin-biosynthesis enzyme MoeA	General function prediction only	R
BrucellaGL001201	BMEI0938	Bme	COG0589	Universal stress protein UspA and related nucleotide-binding proteins	Signal transduction mechanisms	T
BrucellaGL001202	BMEI0940	Bme	COG0503	Adenine/guanine phosphoribosyltransferases and related PRPP-binding proteins	Nucleotide transport and metabolism	F
BrucellaGL001203	BMEI0941	Bme	COG0494	NTP pyrophosphohydrolases including oxidative damage repair enzymes	Replication, recombination and repair	L
BrucellaGL001204	BMEI0943	Bme	COG0209	Ribonucleotide reductase, alpha subunit	Nucleotide transport and metabolism	F
BrucellaGL001205	BMEI0944	Bme	COG0668	Small-conductance mechanosensitive channel	Cell wall/membrane/envelope biogenesis	M
BrucellaGL001206	BMEI0945	Bme	COG1680	Beta-lactamase class C and other penicillin binding proteins	Defense mechanisms	V
BrucellaGL001207	BMEI0946	Bme	COG2081	Predicted flavoproteins	General function prediction only	R
BrucellaGL001208	BMEI0947_1	Bme	COG2202	FOG: PAS/PAC domain	Signal transduction mechanisms	T
BrucellaGL001210	BMEI0949	Bme	COG1734	DnaK suppressor protein	Signal transduction mechanisms	T
BrucellaGL001211	BMEI0950	Bme	COG2062	Phosphohistidine phosphatase SixA	Signal transduction mechanisms	T
BrucellaGL001212	BMEI0951	Bme	COG3106	Predicted ATPase	General function prediction only	R
BrucellaGL001213	BMEI0952	Bme	COG3768	Predicted membrane protein	Function unknown	S
BrucellaGL001215	BMEI0954	Bme	COG0801	7,8-dihydro-6-hydroxymethylpterin-pyrophosphokinase	Coenzyme transport and metabolism	H
BrucellaGL001216	BMEI0955	Bme	COG1539	Dihydroneopterin aldolase	Coenzyme transport and metabolism	H
BrucellaGL001217	BMEI0956	Bme	COG0294	Dihydropteroate synthase and related enzymes	Coenzyme transport and metabolism	H
BrucellaGL001218	BMEI0957	Bme	COG5661	Predicted secreted Zn-dependent protease	Posttranslational modification, protein turnover, chaperones	O
BrucellaGL001219	BMEI0958	Bme	COG0492	Thioredoxin reductase	Posttranslational modification, protein turnover, chaperones	O
BrucellaGL001221	BMEI0959	Bme	COG0633	Ferredoxin	Energy production and conversion	C
BrucellaGL001224	BMEI0962	Bme	COG3218	ABC-type uncharacterized transport system, auxiliary component	General function prediction only	R
BrucellaGL001225	BMEI0963	Bme	COG1463	ABC-type transport system involved in resistance to organic solvents, periplasmic component	Secondary metabolites biosynthesis, transport and catabolism	Q

BrucellaGL001226	BMEI0964	Bme	COG1127	ABC-type transport system involved in resistance to organic solvents, ATPase component	Secondary metabolites biosynthesis, transport and catabolism	Q
BrucellaGL001227	BMEI0965	Bme	COG0767	ABC-type transport system involved in resistance to organic solvents, permease component	Secondary metabolites biosynthesis, transport and catabolism	Q
BrucellaGL001228	BMEI0966	Bme	COG4948	L-alanine-DL-glutamate epimerase and related enzymes of enolase superfamily	Cell wall/membrane/envelope biogenesis	M
BrucellaGL001230	BMEI0967_1	Bme	COG0281	Malic enzyme	Energy production and conversion	C
BrucellaGL001231	BMEI0968	Bme	COG0008	Glutamyl- and glutaminyl-tRNA synthetases	Translation, ribosomal structure and biogenesis	J
BrucellaGL001232	BMEI0969_2	Bme	COG0171	NAD synthase	Coenzyme transport and metabolism	H
BrucellaGL001233	BMEI0970	Bme	COG0818	Diacylglycerol kinase	Cell wall/membrane/envelope biogenesis	M
BrucellaGL001234	BMEI0971	Bme	COG3200	3-deoxy-D-arabino-heptulosonate 7-phosphate (DAHP) synthase	Amino acid transport and metabolism	E
BrucellaGL001235	BMEI0972	Bme	COG1249	Pyruvate/2-oxoglutarate dehydrogenase complex, dihydrolipoamide dehydrogenase (E3) component, and related enzymes	Energy production and conversion	C
BrucellaGL001236	BMEI0973	Bme	COG3184	Uncharacterized protein conserved in bacteria	Function unknown	S
BrucellaGL001237	BMEI0974	Bme	COG0120	Ribose 5-phosphate isomerase	Carbohydrate transport and metabolism	G
BrucellaGL001239	BMEI0975	Bme	COG0546	Predicted phosphatases	General function prediction only	R
BrucellaGL001241	BMEI0977_2	Bme	COG0063	Predicted sugar kinase	Carbohydrate transport and metabolism	G
BrucellaGL001242	BMEI0978	Bme	COG0347	Nitrogen regulatory protein PII	Amino acid transport and metabolism	E
BrucellaGL001243	BMEI0979	Bme	COG0174	Glutamine synthetase	Amino acid transport and metabolism	E
BrucellaGL001244	BMEI0980	Bme	COG5387	Chaperone required for the assembly of the mitochondrial F1-ATPase	Posttranslational modification, protein turnover, chaperones	O
BrucellaGL001245	BMEI0981	Bme	COG0546	Predicted phosphatases	General function prediction only	R
BrucellaGL001246	BMEI0982	Bme	COG0697	Permeases of the drug/metabolite transporter (DMT) superfamily	Carbohydrate transport and metabolism	G
BrucellaGL001247	BMEI0983	Bme	COG0564	Pseudouridylate synthases, 23S RNA-specific	Translation, ribosomal structure and biogenesis	J

BrucellaGL001248	BMEI0984	Bme	COG1132	ABC-type multidrug transport system, ATPase and permease components	Defense mechanisms	V
BrucellaGL001249	BMEI0985	Bme	COG1235	Metal-dependent hydrolases of the beta-lactamase superfamily I	General function prediction only	R
BrucellaGL001250	BMEI0986	Bme	COG0084	Mg-dependent DNase	Replication, recombination and repair	L
BrucellaGL001251	BMEI0987	Bme	COG0143	Methionyl-tRNA synthetase	Translation, ribosomal structure and biogenesis	J
BrucellaGL001253	BMEI0988	Bme	COG0470	ATPase involved in DNA replication	Replication, recombination and repair	L
BrucellaGL001254	BMEI0989	Bme	COG0125	Thymidylate kinase	Nucleotide transport and metabolism	F
BrucellaGL001255	BMEI0990	Bme	COG1686	D-alanyl-D-alanine carboxypeptidase	Cell wall/membrane/envelope biogenesis	M
BrucellaGL001256	BMEI0991	Bme	COG0797	Lipoproteins	Cell wall/membrane/envelope biogenesis	M
BrucellaGL001257	BMEI0992	Bme	COG1393	Arsenate reductase and related proteins, glutaredoxin family	Inorganic ion transport and metabolism	P
BrucellaGL001260	BMEI0995	Bme	COG3926	Putative secretion activating protein	General function prediction only	R
BrucellaGL001261	BMEI0997	Bme	COG0438	Glycosyltransferase	Cell wall/membrane/envelope biogenesis	M
BrucellaGL001262	BMEI0998	Bme	COG0438	Glycosyltransferase	Cell wall/membrane/envelope biogenesis	M
BrucellaGL001268	BMEI1004	Bme	COG2929	Uncharacterized protein conserved in bacteria	Function unknown	S
BrucellaGL001269	SMc04432	Sme	COG3514	Uncharacterized protein conserved in bacteria	Function unknown	S
BrucellaGL001271	BMEI1007	Bme	COG3637	Opacity protein and related surface antigens	Cell wall/membrane/envelope biogenesis	M
BrucellaGL001277	BMEI1012	Bme	COG0582	Integrase	Replication, recombination and repair	L
BrucellaGL001279	BMEI1013	Bme	COG2261	Predicted membrane protein	Function unknown	S
BrucellaGL001280	BMEI1014	Bme	COG2989	Uncharacterized protein conserved in bacteria	Function unknown	S
BrucellaGL001283	BMEI1016_1	Bme	COG1951	Tartrate dehydratase alpha subunit/Fumarate hydratase class I, N-terminal domain	Energy production and conversion	C
BrucellaGL001284	BMEI1017	Bme	COG0654	2-polyprenyl-6-methoxyphenol hydroxylase and related FAD-dependent oxidoreductases	Coenzyme transport and metabolism	H
BrucellaGL001285	BMEI1018	Bme	COG3536	Uncharacterized protein conserved in bacteria	Function unknown	S
BrucellaGL001286	BMEI1019	Bme	COG2896	Molybdenum cofactor biosynthesis enzyme	Coenzyme transport and metabolism	H

BrucellaGL001287	BMEI1020	Bme	COG0746	Molybdopterin-guanine dinucleotide biosynthesis protein A	Coenzyme transport and metabolism	H
BrucellaGL001288	mlr0548	Mlo	COG1763	Molybdopterin-guanine dinucleotide biosynthesis protein	Coenzyme transport and metabolism	H
BrucellaGL001290	BMEI1022	Bme	COG0834	ABC-type amino acid transport/signal transduction systems, periplasmic component/domain	Amino acid transport and metabolism	E
BrucellaGL001291	BMEI1023	Bme	COG0625	Glutathione S-transferase	Posttranslational modification, protein turnover, chaperones	O
BrucellaGL001292	BMEI1024	Bme	COG2084	3-hydroxyisobutyrate dehydrogenase and related beta-hydroxyacid dehydrogenases	Lipid transport and metabolism	I
BrucellaGL001293	BMEI1024	Bme	COG2084	3-hydroxyisobutyrate dehydrogenase and related beta-hydroxyacid dehydrogenases	Lipid transport and metabolism	I
BrucellaGL001294	BMEI1025	Bme	COG2067	Long-chain fatty acid transport protein	Lipid transport and metabolism	I
BrucellaGL001296	BMEI1027	Bme	COG0525	Valyl-tRNA synthetase	Translation, ribosomal structure and biogenesis	J
BrucellaGL001297	BMEI1028	Bme	COG3827	Uncharacterized protein conserved in bacteria	Function unknown	S
BrucellaGL001298	BMEI1029	Bme	COG1538	Outer membrane protein	Cell wall/membrane/envelope biogenesis	M
BrucellaGL001299	BMEI1030	Bme	COG2518	Protein-L-isoaspartate carboxylmethyltransferase	Posttranslational modification, protein turnover, chaperones	O
BrucellaGL001301	BMEI1032	Bme	COG2608	Copper chaperone	Inorganic ion transport and metabolism	P
BrucellaGL001303	BMEI1034	Bme	COG0316	Uncharacterized conserved protein	Function unknown	S
BrucellaGL001304	BMEI1035	Bme	COG0513	Superfamily II DNA and RNA helicases	Replication, recombination and repair	L
BrucellaGL001305	BMEI1036	Bme	COG0061	Predicted sugar kinase	Carbohydrate transport and metabolism	G
BrucellaGL001306	BMEI1037	Bme	COG0463	Glycosyltransferases involved in cell wall biogenesis	Cell wall/membrane/envelope biogenesis	M
BrucellaGL001307	BMEI1038	Bme	COG2151	Predicted metal-sulfur cluster biosynthetic enzyme	General function prediction only	R
BrucellaGL001308	BMEI1039	Bme	COG0520	Selenocysteine lyase	Amino acid transport and metabolism	E
BrucellaGL001309	BMEI1040	Bme	COG0719	ABC-type transport system involved in Fe-S cluster assembly, permease component	Posttranslational modification, protein turnover, chaperones	O

BrucellaGL001310	BMEI1041	Bme	COG0396	ABC-type transport system involved in Fe-S cluster assembly, ATPase component	Posttranslational modification, protein turnover, chaperones	O
BrucellaGL001311	BMEI1042	Bme	COG0719	ABC-type transport system involved in Fe-S cluster assembly, permease component	Posttranslational modification, protein turnover, chaperones	O
BrucellaGL001312	BMEI1043	Bme	COG1104	Cysteine sulfinate desulfinase/cysteine desulfurase and related enzymes	Amino acid transport and metabolism	E
BrucellaGL001313	BMEI1045	Bme	COG2076	Membrane transporters of cations and cationic drugs	Inorganic ion transport and metabolism	P
BrucellaGL001314	BMEI1046	Bme	COG2377	Predicted molecular chaperone distantly related to HSP70-fold metalloproteases	Posttranslational modification, protein turnover, chaperones	O
BrucellaGL001315	BMEI1047	Bme	COG0162	Tyrosyl-tRNA synthetase	Translation, ribosomal structure and biogenesis	J
BrucellaGL001318	BMEI1049	Bme	COG1225	Peroxiredoxin	Posttranslational modification, protein turnover, chaperones	O
BrucellaGL001319	BMEI1050	Bme	COG2833	Uncharacterized protein conserved in bacteria	Function unknown	S
BrucellaGL001324	BMEI1055	Bme	COG5009	Membrane carboxypeptidase/penicillin-binding protein	Cell wall/membrane/envelope biogenesis	M
BrucellaGL001326	BMEI1056	Bme	COG0860	N-acetylmuramoyl-L-alanine amidase	Cell wall/membrane/envelope biogenesis	M
BrucellaGL001328	BMEI1057	Bme	COG1530	Ribonucleases G and E	Translation, ribosomal structure and biogenesis	J
BrucellaGL001329	BMEI1058	Bme	COG0436	Aspartate/tyrosine/aromatic aminotransferase	Amino acid transport and metabolism	E
BrucellaGL001330	BMEI1059	Bme	COG4783	Putative Zn-dependent protease, contains TPR repeats	General function prediction only	R
BrucellaGL001331	BMEI1060	Bme	COG1651	Protein-disulfide isomerase	Posttranslational modification, protein turnover, chaperones	O
BrucellaGL001332	BMEI1061	Bme	COG0757	3-dehydroquinate dehydratase II	Amino acid transport and metabolism	E
BrucellaGL001333	BMEI1062	Bme	COG0511	Biotin carboxyl carrier protein	Lipid transport and metabolism	I
BrucellaGL001335	BMEI1063	Bme	COG0439	Biotin carboxylase	Lipid transport and metabolism	I
BrucellaGL001336	BMEI1064	Bme	COG2360	Leu/Phe-tRNA-protein transferase	Posttranslational modification, protein turnover, chaperones	O
BrucellaGL001337	BMEI1065	Bme	COG4765	Uncharacterized protein conserved in bacteria	Function unknown	S

BrucellaGL001339	BMEI1066	Bme	COG3761	NADH:ubiquinone oxidoreductase 17.2 kD subunit	Energy production and conversion	C
BrucellaGL001340	BMEI1067	Bme	COG0454	Histone acetyltransferase HPA2 and related acetyltransferases	Transcription	K
BrucellaGL001341	BMEI1068	Bme	COG0064	Asp-tRNAAsn/Glu-tRNAGln amidotransferase B subunit (PET112 homolog)	Translation, ribosomal structure and biogenesis	J
BrucellaGL001342	BMEI1069	Bme	COG0544	FKBP-type peptidyl-prolyl cis-trans isomerase (trigger factor)	Posttranslational modification, protein turnover, chaperones	O
BrucellaGL001344	BMEI1070	Bme	COG4929	Uncharacterized membrane-anchored protein	Function unknown	S
BrucellaGL001345	BMEI1071	Bme	COG4872	Predicted membrane protein	Function unknown	S
BrucellaGL001346	BMEI1072	Bme	COG5457	Uncharacterized conserved small protein	Function unknown	S
BrucellaGL001347	BMEI1073	Bme	COG1206	NAD(FAD)-utilizing enzyme possibly involved in translation	Translation, ribosomal structure and biogenesis	J
BrucellaGL001348	BMEI1074	Bme	COG1562	Phytoene/squalene synthetase	Lipid transport and metabolism	I
BrucellaGL001349	BMEI1075	Bme	COG3737	Uncharacterized conserved protein	Function unknown	S
BrucellaGL001350	BMEI1076_1	Bme	COG0342	Preprotein translocase subunit SecD	Intracellular trafficking, secretion, and vesicular transport	U
BrucellaGL001351	BMEI1077	Bme	COG1862	Preprotein translocase subunit YajC	Intracellular trafficking, secretion, and vesicular transport	U
BrucellaGL001352	BMEI1078	Bme	COG2607	Predicted ATPase (AAA+ superfamily)	General function prediction only	R
BrucellaGL001355	BMEI1079	Bme	COG0739	Membrane proteins related to metalloendopeptidases	Cell wall/membrane/envelope biogenesis	M
BrucellaGL001356	BMEI1080	Bme	COG2518	Protein-L-isoaspartate carboxylmethyltransferase	Posttranslational modification, protein turnover, chaperones	O
BrucellaGL001357	BMEI1081	Bme	COG0496	Predicted acid phosphatase	General function prediction only	R
BrucellaGL001358	BMEI1082	Bme	COG0172	Seryl-tRNA synthetase	Translation, ribosomal structure and biogenesis	J
BrucellaGL001359	BMEI1083	Bme	COG0805	Sec-independent protein secretion pathway component TatC	Intracellular trafficking, secretion, and vesicular transport	U
BrucellaGL001360	SMc02066	Sme	COG1826	Sec-independent protein secretion pathway components	Intracellular trafficking, secretion, and vesicular transport	U
BrucellaGL001362	BMEI1084	Bme	COG1826	Sec-independent protein secretion pathway components	Intracellular trafficking, secretion, and vesicular transport	U

BrucellaGL001363	BMEI1085	Bme	COG1386	Predicted transcriptional regulator containing the HTH domain	Transcription	K
BrucellaGL001364	BMEI1086	Bme	COG1354	Uncharacterized conserved protein	Function unknown	S
BrucellaGL001365	BMEI1087	Bme	COG1472	Beta-glucosidase-related glycosidases	Carbohydrate transport and metabolism	G
BrucellaGL001367	BMEI1089	Bme	COG0018	Arginyl-tRNA synthetase	Translation, ribosomal structure and biogenesis	J
BrucellaGL001368	BMEI1090	Bme	COG0232	dGTP triphosphohydrolase	Nucleotide transport and metabolism	F
BrucellaGL001369	BMEI1091	Bme	COG0316	Uncharacterized conserved protein	Function unknown	S
BrucellaGL001370	AGl748	Atu	COG2979	Uncharacterized protein conserved in bacteria	Function unknown	S
BrucellaGL001371	BMEI1093	Bme	COG0708	Exonuclease III	Replication, recombination and repair	L
BrucellaGL001372	BMEI1094	Bme	COG0790	FOG: TPR repeat, SEL1 subfamily	General function prediction only	R
BrucellaGL001373	BMEI1096	Bme	COG1525	Micrococcal nuclease (thermonuclease) homologs	Replication, recombination and repair	L
BrucellaGL001374	BMEI1097	Bme	COG1573	Uracil-DNA glycosylase	Replication, recombination and repair	L
BrucellaGL001375	BMEI1098	Bme	COG1522	Transcriptional regulators	Transcription	K
BrucellaGL001376	BMEI1099	Bme	COG2038	NaMN:DMB phosphoribosyltransferase	Coenzyme transport and metabolism	H
BrucellaGL001377	BMEI1100	Bme	COG0368	Cobalamin-5-phosphate synthase	Coenzyme transport and metabolism	H
BrucellaGL001378	BMEI1101	Bme	COG3313	Predicted Fe-S protein	General function prediction only	R
BrucellaGL001379	BMEI1102	Bme	COG3577	Predicted aspartyl protease	General function prediction only	R
BrucellaGL001380	BMEI1103	Bme	COG0042	tRNA-dihydrouridine synthase	Translation, ribosomal structure and biogenesis	J
BrucellaGL001381	BMEI1104	Bme	COG0834	ABC-type amino acid transport/signal transduction systems, periplasmic component/domain	Amino acid transport and metabolism	E
BrucellaGL001382	BMEI1105	Bme	COG0637	Predicted phosphatase/phosphohexomutase	General function prediction only	R
BrucellaGL001383	BMEI1106	Bme	COG3454	Metal-dependent hydrolase involved in phosphonate metabolism	Inorganic ion transport and metabolism	P
BrucellaGL001385	BMEI1108	Bme	COG0524	Sugar kinases, ribokinase family	Carbohydrate transport and metabolism	G
BrucellaGL001386	BMEI1109	Bme	COG2017	Galactose mutarotase and related enzymes	Carbohydrate transport and metabolism	G

BrucellaGL001387	BMEI1110	Bme	COG3926	Putative secretion activating protein	General function prediction only	R
BrucellaGL001388	BMEI1111	Bme	COG0236	Acyl carrier protein	Lipid transport and metabolism	I
BrucellaGL001389	BMEI1112	Bme	COG0304	3-oxoacyl-(acyl-carrier-protein) synthase	Lipid transport and metabolism	I
BrucellaGL001390	BMEI1113	Bme	COG0304	3-oxoacyl-(acyl-carrier-protein) synthase	Lipid transport and metabolism	I
BrucellaGL001391	BMEI1114	Bme	COG0604	NADPH:quinone reductase and related Zn-dependent oxidoreductases	Energy production and conversion	C
BrucellaGL001392	BMEI1115	Bme	COG1560	Lauroyl/myristoyl acyltransferase	Cell wall/membrane/envelope biogenesis	M
BrucellaGL001393	BMEI1116	Bme	COG0036	Pentose-5-phosphate-3-epimerase	Carbohydrate transport and metabolism	G
BrucellaGL001394	BMEI1117	Bme	COG0015	Adenylosuccinate lyase	Nucleotide transport and metabolism	F
BrucellaGL001395	BMEI1118	Bme	COG2514	Predicted ring-cleavage extradiol dioxygenase	General function prediction only	R
BrucellaGL001396	BMEI1119	Bme	COG3545	Predicted esterase of the alpha/beta hydrolase fold	General function prediction only	R
BrucellaGL001397	BMEI1120	Bme	COG3836	2,4-dihydroxyhept-2-ene-1,7-dioic acid aldolase	Carbohydrate transport and metabolism	G
BrucellaGL001398	BMEI1121	Bme	COG5467	Uncharacterized conserved protein	Function unknown	S
BrucellaGL001399	BMEI1122	Bme	COG0152	Phosphoribosylaminoimidazolesuccinocarboxamide (SAICAR) synthase	Nucleotide transport and metabolism	F
BrucellaGL001400	BMEI1123	Bme	COG1828	Phosphoribosylformylglycinamidine (FGAM) synthase, PurS component	Nucleotide transport and metabolism	F
BrucellaGL001401	BMEI1124	Bme	COG0047	Phosphoribosylformylglycinamidine (FGAM) synthase, glutamine amidotransferase domain	Nucleotide transport and metabolism	F
BrucellaGL001402	BMEI1125	Bme	COG0625	Glutathione S-transferase	Posttranslational modification, protein turnover, chaperones	O
BrucellaGL001404	BMEI1127	Bme	COG0046	Phosphoribosylformylglycinamidine (FGAM) synthase, synthetase domain	Nucleotide transport and metabolism	F
BrucellaGL001405	BMEI1128	Bme	COG0271	Stress-induced morphogen (activity unknown)	Signal transduction mechanisms	T
BrucellaGL001406	BMEI1129	Bme	COG0278	Glutaredoxin-related protein	Posttranslational modification, protein turnover, chaperones	O
BrucellaGL001407	mll0051	Mlo	COG0477	Permeases of the major facilitator superfamily	Carbohydrate transport and metabolism	G

BrucellaGL001408	BMEI1131	Bme	COG0483	Archaeal fructose-1,6-bisphosphatase and related enzymes of inositol monophosphatase family	Carbohydrate transport and metabolism	G
BrucellaGL001409	BMEI1132	Bme	COG0037	Predicted ATPase of the PP-loop superfamily implicated in cell cycle control	Cell cycle control, cell division, chromosome partitioning	D
BrucellaGL001410	BMEI1133	Bme	COG0522	Ribosomal protein S4 and related proteins	Translation, ribosomal structure and biogenesis	J
BrucellaGL001412	BMEI1134	Bme	COG3921	Uncharacterized protein conserved in bacteria	Function unknown	S
BrucellaGL001415	BMEI1137	Bme	COG0587	DNA polymerase III, alpha subunit	Replication, recombination and repair	L
BrucellaGL001416	BMEI1138	Bme	COG1136	ABC-type antimicrobial peptide transport system, ATPase component	Defense mechanisms	V
BrucellaGL001417	BMEI1139	Bme	COG4591	ABC-type transport system, involved in lipoprotein release, permease component	Cell wall/membrane/envelope biogenesis	M
BrucellaGL001418	BMEI1140	Bme	COG0442	Prolyl-tRNA synthetase	Translation, ribosomal structure and biogenesis	J
BrucellaGL001420	BMEI1141	Bme	COG5454	Predicted secreted protein	Function unknown	S
BrucellaGL001421	BMEI1142	Bme	COG0346	Lactoylglutathione lyase and related lyases	Amino acid transport and metabolism	E
BrucellaGL001422	BMEI1143	Bme	COG0595	Predicted hydrolase of the metallo-beta-lactamase superfamily	General function prediction only	R
BrucellaGL001423	BMEI1143	Bme	COG0595	Predicted hydrolase of the metallo-beta-lactamase superfamily	General function prediction only	R
BrucellaGL001424	BMEI1144	Bme	COG0340	Biotin-(acetyl-CoA carboxylase) ligase	Coenzyme transport and metabolism	H
BrucellaGL001425	BMEI1145	Bme	COG1007	NADH:ubiquinone oxidoreductase subunit 2 (chain N)	Energy production and conversion	C
BrucellaGL001426	BMEI1146	Bme	COG1008	NADH:ubiquinone oxidoreductase subunit 4 (chain M)	Energy production and conversion	C
BrucellaGL001427	BMEI1147	Bme	COG1009	NADH:ubiquinone oxidoreductase subunit 5 (chain L)/Multisubunit Na+/H+ antiporter, MnhA subunit	Energy production and conversion	C
BrucellaGL001428	BMEI1148	Bme	COG0713	NADH:ubiquinone oxidoreductase subunit 11 or 4L (chain K)	Energy production and conversion	C
BrucellaGL001429	BMEI1149	Bme	COG0839	NADH:ubiquinone oxidoreductase subunit 6 (chain J)	Energy production and conversion	C

BrucellaGL001430	BMEI1150	Bme	COG1143	Formate hydrogenlyase subunit 6/NADH:ubiquinone oxidoreductase 23 kD subunit (chain I)	Energy production and conversion	C
BrucellaGL001431	BMEI1151	Bme	COG1005	NADH:ubiquinone oxidoreductase subunit 1 (chain H)	Energy production and conversion	C
BrucellaGL001432	BMEI1152	Bme	COG1034	NADH dehydrogenase/NADH:ubiquinone oxidoreductase 75 kD subunit (chain G)	Energy production and conversion	C
BrucellaGL001433	BMEI1153	Bme	COG1894	NADH:ubiquinone oxidoreductase, NADH-binding (51 kD) subunit	Energy production and conversion	C
BrucellaGL001434	BMEI1154	Bme	COG1905	NADH:ubiquinone oxidoreductase 24 kD subunit	Energy production and conversion	C
BrucellaGL001435	BMEI1155	Bme	COG0649	NADH:ubiquinone oxidoreductase 49 kD subunit 7	Energy production and conversion	C
BrucellaGL001436	BMEI1156	Bme	COG0852	NADH:ubiquinone oxidoreductase 27 kD subunit	Energy production and conversion	C
BrucellaGL001437	BMEI1157	Bme	COG0377	NADH:ubiquinone oxidoreductase 20 kD subunit and related Fe-S oxidoreductases	Energy production and conversion	C
BrucellaGL001438	BMEI1158	Bme	COG0838	NADH:ubiquinone oxidoreductase subunit 3 (chain A)	Energy production and conversion	C
BrucellaGL001444	BMEI1001	Bme	COG3293	Transposase and inactivated derivatives	Replication, recombination and repair	L
BrucellaGL001445	BMEI1053	Bme	COG3293	Transposase and inactivated derivatives	Replication, recombination and repair	L
BrucellaGL001447	BMEI1166	Bme	COG2873	O-acetylhomoserine sulfhydrylase	Amino acid transport and metabolism	E
BrucellaGL001448	BMEI1167	Bme	COG2050	Uncharacterized protein, possibly involved in aromatic compounds catabolism	Secondary metabolites biosynthesis, transport and catabolism	Q
BrucellaGL001449	BMEI1168	Bme	COG0102	Ribosomal protein L13	Translation, ribosomal structure and biogenesis	J
BrucellaGL001450	BMEI1169	Bme	COG0103	Ribosomal protein S9	Translation, ribosomal structure and biogenesis	J
BrucellaGL001452	BMEI1170	Bme	COG0010	Arginase/agmatinase/formimionoglutamate hydrolase, arginase family	Amino acid transport and metabolism	E
BrucellaGL001453	BMEI1171	Bme	COG0002	Acetylglutamate semialdehyde dehydrogenase	Amino acid transport and metabolism	E
BrucellaGL001454	BMEI1172	Bme	COG1612	Uncharacterized protein required for cytochrome oxidase assembly	Posttranslational modification, protein turnover, chaperones	O
BrucellaGL001457	BMEI1174	Bme	COG0489	ATPases involved in chromosome partitioning	Cell cycle control, cell division, chromosome partitioning	D

BrucellaGL001459	BMEI1175	Bme	COG1596	Periplasmic protein involved in polysaccharide export	Cell wall/membrane/envelope biogenesis	M
BrucellaGL001460	BMEI1177	Bme	COG2148	Sugar transferases involved in lipopolysaccharide synthesis	Cell wall/membrane/envelope biogenesis	M
BrucellaGL001461	YPO0355	Ype	COG2076	Membrane transporters of cations and cationic drugs	Inorganic ion transport and metabolism	P
BrucellaGL001462	BMEI1178	Bme	COG0789	Predicted transcriptional regulators	Transcription	K
BrucellaGL001463	BMEI1179	Bme	COG0776	Bacterial nucleoid DNA-binding protein	Replication, recombination and repair	L
BrucellaGL001464	BMEI1180	Bme	COG0332	3-oxoacyl-[acyl-carrier-protein] synthase III	Lipid transport and metabolism	I
BrucellaGL001465	BMEI1181	Bme	COG0416	Fatty acid/phospholipid biosynthesis enzyme	Lipid transport and metabolism	I
BrucellaGL001467	BMEI1183	Bme	COG5452	Uncharacterized conserved protein	Function unknown	S
BrucellaGL001468	BMEI1184	Bme	COG2913	Small protein A (tmRNA-binding)	Translation, ribosomal structure and biogenesis	J
BrucellaGL001469	BMEI1185	Bme	COG3808	Inorganic pyrophosphatase	Energy production and conversion	C
BrucellaGL001470	BMEI1186	Bme	COG0781	Transcription termination factor	Transcription	K
BrucellaGL001471	BMEI1187	Bme	COG0054	Riboflavin synthase beta-chain	Coenzyme transport and metabolism	H
BrucellaGL001472	BMEI1188	Bme	COG0307	Riboflavin synthase alpha chain	Coenzyme transport and metabolism	H
BrucellaGL001473	BMEI1189_2	Bme	COG1985	Pyrimidine reductase, riboflavin biosynthesis	Coenzyme transport and metabolism	H
BrucellaGL001474	BMEI1190	Bme	COG1327	Predicted transcriptional regulator, consists of a Zn-ribbon and ATP-cone domains	Transcription	K
BrucellaGL001475	SMc01770	Sme	COG0112	Glycine/serine hydroxymethyltransferase	Amino acid transport and metabolism	E
BrucellaGL001476	BMEI1193	Bme	COG2989	Uncharacterized protein conserved in bacteria	Function unknown	S
BrucellaGL001478	BMEI1194	Bme	COG1846	Transcriptional regulators	Transcription	K
BrucellaGL001480	BMEI1196	Bme	COG1024	Enoyl-CoA hydratase/carnithine racemase	Lipid transport and metabolism	I
BrucellaGL001481	BMEI1197	Bme	COG0113	Delta-aminolevulinic acid dehydratase	Coenzyme transport and metabolism	H
BrucellaGL001482	BMEI1198	Bme	COG1714	Predicted membrane protein/domain	Function unknown	S
BrucellaGL001483	BMEI1199	Bme	COG2935	Putative arginyl-tRNA:protein arginylyltransferase	Posttranslational modification, protein turnover, chaperones	O
BrucellaGL001484	BMEI1200	Bme	COG0188	Type IIA topoisomerase (DNA gyrase/topo II, topoisomerase IV), A subunit	Replication, recombination and repair	L
BrucellaGL001486	BMEI1202	Bme	COG0173	Aspartyl-tRNA synthetase	Translation, ribosomal structure and biogenesis	J

BrucellaGL001487	BMEI1203	Bme	COG0349	Ribonuclease D	Translation, ribosomal structure and biogenesis	J
BrucellaGL001488	BMEI1204	Bme	COG0248	Exopolyphosphatase	Nucleotide transport and metabolism	F
BrucellaGL001489	BMEI1205	Bme	COG0855	Polyphosphate kinase	Inorganic ion transport and metabolism	P
BrucellaGL001490	BMEI1206	Bme	COG4391	Uncharacterized protein conserved in bacteria	Function unknown	S
BrucellaGL001491	BMEI1207	Bme	COG0654	2-polyprenyl-6-methoxyphenol hydroxylase and related FAD-dependent oxidoreductases	Coenzyme transport and metabolism	H
BrucellaGL001492	BMEI1208	Bme	COG1126	ABC-type polar amino acid transport system, ATPase component	Amino acid transport and metabolism	E
BrucellaGL001493	BMEI1209	Bme	COG0765	ABC-type amino acid transport system, permease component	Amino acid transport and metabolism	E
BrucellaGL001495	BMEI1210	Bme	COG4597	ABC-type amino acid transport system, permease component	Amino acid transport and metabolism	E
BrucellaGL001496	BMEI1211	Bme	COG0834	ABC-type amino acid transport/signal transduction systems, periplasmic component/domain	Amino acid transport and metabolism	E
BrucellaGL001497	BMEI1212	Bme	COG0671	Membrane-associated phospholipid phosphatase	Lipid transport and metabolism	I
BrucellaGL001498	BMEI1213	Bme	COG0626	Cystathionine beta-lyases/cystathionine gamma-synthases	Amino acid transport and metabolism	E
BrucellaGL001501	BMEI1215	Bme	COG4991	Uncharacterized protein with a bacterial SH3 domain homologue	Function unknown	S
BrucellaGL001502	BMEI1216	Bme	COG4916	Uncharacterized protein containing a TIR (Toll-Interleukin 1-resistance) domain	Function unknown	S
BrucellaGL001507	BMEI1223	Bme	COG2801	Transposase and inactivated derivatives	Replication, recombination and repair	L
BrucellaGL001511	BMEI1052	Bme	COG3293	Transposase and inactivated derivatives	Replication, recombination and repair	L
BrucellaGL001512	BMEI1053	Bme	COG3293	Transposase and inactivated derivatives	Replication, recombination and repair	L
BrucellaGL001516	BMEI1232	Bme	COG1620	L-lactate permease	Energy production and conversion	C
BrucellaGL001517	BMEI1233	Bme	COG1620	L-lactate permease	Energy production and conversion	C
BrucellaGL001520	AGc2465	Atu	COG1028	Dehydrogenases with different specificities (related to short-chain alcohol dehydrogenases)	Lipid transport and metabolism	I
BrucellaGL001522	BMEI1237	Bme	COG0451	Nucleoside-diphosphate-sugar epimerases	Cell wall/membrane/envelope biogenesis	M

BrucellaGL001523	BMEI1238	Bme	COG0593	ATPase involved in DNA replication initiation	Replication, recombination and repair	L
BrucellaGL001524	BMEI1239	Bme	COG0628	Predicted permease	General function prediction only	R
BrucellaGL001526	BMEI1240	Bme	COG0150	Phosphoribosylaminoimidazole (AIR) synthetase	Nucleotide transport and metabolism	F
BrucellaGL001527	BMEI1241	Bme	COG0299	Folate-dependent phosphoribosylglycinamide formyltransferase PurN	Nucleotide transport and metabolism	F
BrucellaGL001529	BMEI1243	Bme	COG0243	Anaerobic dehydrogenases, typically selenocysteine-containing	Energy production and conversion	C
BrucellaGL001531	BMEI1244	Bme	COG0697	Permeases of the drug/metabolite transporter (DMT) superfamily	Carbohydrate transport and metabolism	G
BrucellaGL001532	BMEI1245	Bme	COG2961	Protein involved in catabolism of external DNA	General function prediction only	R
BrucellaGL001533	BMEI1246	Bme	COG5511	Bacteriophage capsid protein	General function prediction only	R
BrucellaGL001534	BMEI1247	Bme	COG3719	Ribonuclease I	Translation, ribosomal structure and biogenesis	J
BrucellaGL001535	BMEI1248	Bme	COG0625	Glutathione S-transferase	Posttranslational modification, protein turnover, chaperones	O
BrucellaGL001536	BMEI1249	Bme	COG3637	Opacity protein and related surface antigens	Cell wall/membrane/envelope biogenesis	M
BrucellaGL001538	AGc2091	Atu	COG1028	Dehydrogenases with different specificities (related to short-chain alcohol dehydrogenases)	Lipid transport and metabolism	I
BrucellaGL001539	BMEI1251	Bme	COG0322	Nuclease subunit of the excinuclease complex	Replication, recombination and repair	L
BrucellaGL001540	BMEI1252	Bme	COG0558	Phosphatidylglycerophosphate synthase	Lipid transport and metabolism	I
BrucellaGL001541	BMEI1253	Bme	COG1977	Molybdopterin converting factor, small subunit	Coenzyme transport and metabolism	H
BrucellaGL001542	BMEI1254	Bme	COG0314	Molybdopterin converting factor, large subunit	Coenzyme transport and metabolism	H
BrucellaGL001543	BMEI1255_1	Bme	COG3126	Uncharacterized protein conserved in bacteria	Function unknown	S
BrucellaGL001544	BMEI1256	Bme	COG0105	Nucleoside diphosphate kinase	Nucleotide transport and metabolism	F
BrucellaGL001545	BMEI1257	Bme	COG2318	Uncharacterized protein conserved in bacteria	Function unknown	S
BrucellaGL001546	BMEI1258	Bme	COG0488	ATPase components of ABC transporters with duplicated ATPase domains	General function prediction only	R
BrucellaGL001548	BMEI1260	Bme	COG2927	DNA polymerase III, chi subunit	Replication, recombination and repair	L
BrucellaGL001549	BMEI1261	Bme	COG0260	Leucyl aminopeptidase	Amino acid transport and metabolism	E
BrucellaGL001551	BMEI1262	Bme	COG0795	Predicted permeases	General function prediction only	R
BrucellaGL001552	BMEI1263	Bme	COG0795	Predicted permeases	General function prediction only	R

BrucellaGL001553	BMEI1264	Bme	COG1452	Organic solvent tolerance protein OstA	Cell wall/membrane/envelope biogenesis	M
BrucellaGL001554	BMEI1265	Bme	COG0760	Parvulin-like peptidyl-prolyl isomerase	Posttranslational modification, protein turnover, chaperones	O
BrucellaGL001555	BMEI1266	Bme	COG1995	Pyridoxal phosphate biosynthesis protein	Coenzyme transport and metabolism	H
BrucellaGL001556	BMEI1267	Bme	COG0030	Dimethyladenosine transferase (rRNA methylation)	Translation, ribosomal structure and biogenesis	J
BrucellaGL001557	BMEI1268	Bme	COG0739	Membrane proteins related to metalloendopeptidases	Cell wall/membrane/envelope biogenesis	M
BrucellaGL001558	BMEI1268	Bme	COG0739	Membrane proteins related to metalloendopeptidases	Cell wall/membrane/envelope biogenesis	M
BrucellaGL001559	BMEI1269	Bme	COG2962	Predicted permeases	General function prediction only	R
BrucellaGL001560	BMEI1270	Bme	COG0119	Isopropylmalate/homocitrate/citramalate synthases	Amino acid transport and metabolism	E
BrucellaGL001561	BMEI1272	Bme	COG0215	Cysteinyl-tRNA synthetase	Translation, ribosomal structure and biogenesis	J
BrucellaGL001563	BMEI1274	Bme	COG1279	Lysine efflux permease	General function prediction only	R
BrucellaGL001564	BMEI1275	Bme	COG2135	Uncharacterized conserved protein	Function unknown	S
BrucellaGL001565	BMEI1276	Bme	COG1051	ADP-ribose pyrophosphatase	Nucleotide transport and metabolism	F
BrucellaGL001566	BMEI1277	Bme	COG5451	Predicted secreted protein	Function unknown	S
BrucellaGL001569	BMEI1281	Bme	COG0044	Dihydroorotase and related cyclic amidohydrolases	Nucleotide transport and metabolism	F
BrucellaGL001570	BMEI1282	Bme	COG0354	Predicted aminomethyltransferase related to GcvT	General function prediction only	R
BrucellaGL001571	BMEI1283	Bme	COG1896	Predicted hydrolases of HD superfamily	General function prediction only	R
BrucellaGL001572	BMEI1284	Bme	COG5350	Predicted protein tyrosine phosphatase	General function prediction only	R
BrucellaGL001573	BMEI1285	Bme	COG1381	Recombinational DNA repair protein (RecF pathway)	Replication, recombination and repair	L
BrucellaGL001574	BMEI1286	Bme	COG1159	GTPase	General function prediction only	R
BrucellaGL001575	BMEI1287	Bme	COG0571	dsRNA-specific ribonuclease	Transcription	K
BrucellaGL001576	BMEI1288	Bme	COG0681	Signal peptidase I	Intracellular trafficking, secretion, and vesicular transport	U

BrucellaGL001577	BMEI1289	Bme	COG0736	Phosphopantetheinyl transferase (holo-ACP synthase)	Lipid transport and metabolism	I
BrucellaGL001578	BMEI1290	Bme	COG3216	Uncharacterized protein conserved in bacteria	Function unknown	S
BrucellaGL001579	BMEI1291	Bme	COG2207	AraC-type DNA-binding domain-containing proteins	Transcription	K
BrucellaGL001580	ECs0532	Ecs	COG0477	Permeases of the major facilitator superfamily	Carbohydrate transport and metabolism	G
BrucellaGL001581	BMEI1293	Bme	COG0635	Coproporphyrinogen III oxidase and related Fe-S oxidoreductases	Coenzyme transport and metabolism	H
BrucellaGL001582	BMEI1294	Bme	COG0664	cAMP-binding proteins - catabolite gene activator and regulatory subunit of cAMP-dependent protein kinases	Signal transduction mechanisms	T
BrucellaGL001583	BMEI1295	Bme	COG0461	Orotate phosphoribosyltransferase	Nucleotide transport and metabolism	F
BrucellaGL001584	BMEI1296	Bme	COG0317	Guanosine polyphosphate pyrophosphohydrolases/synthetases	Signal transduction mechanisms	T
BrucellaGL001585	BMEI1297	Bme	COG1758	DNA-directed RNA polymerase, subunit K/omega	Transcription	K
BrucellaGL001586	BMEI1298	Bme	COG1432	Uncharacterized conserved protein	Function unknown	S
BrucellaGL001587	BMEI1299	Bme	COG1573	Uracil-DNA glycosylase	Replication, recombination and repair	L
BrucellaGL001588	BMEI1300	Bme	COG0691	tmRNA-binding protein	Posttranslational modification, protein turnover, chaperones	O
BrucellaGL001589	BMEI1301	Bme	COG0329	Dihydrodipicolinate synthase/N-acetylneuraminate lyase	Amino acid transport and metabolism	E
BrucellaGL001591	BMEI1302	Bme	COG0741	Soluble lytic murein transglycosylase and related regulatory proteins (some contain LysM/invasin domains)	Cell wall/membrane/envelope biogenesis	M
BrucellaGL001592	BMEI1303	Bme	COG3743	Uncharacterized conserved protein	Function unknown	S
BrucellaGL001597	BMEI1307	Bme	COG0582	Integrase	Replication, recombination and repair	L
BrucellaGL001598	mll7737	Mlo	COG0079	Histidinol-phosphate/aromatic aminotransferase and cobyric acid decarboxylase	Amino acid transport and metabolism	E
BrucellaGL001599	BMEI1310	Bme	COG2363	Uncharacterized small membrane protein	Function unknown	S
BrucellaGL001601	BMEI1312	Bme	COG1284	Uncharacterized conserved protein	Function unknown	S
BrucellaGL001602	BMEI1313	Bme	COG0790	FOG: TPR repeat, SEL1 subfamily	General function prediction only	R
BrucellaGL001604	BMEI1315	Bme	COG4731	Uncharacterized protein conserved in bacteria	Function unknown	S

BrucellaGL001605	BMEI1316	Bme	COG0625	Glutathione S-transferase	Posttranslational modification, protein turnover, chaperones	O
BrucellaGL001608	BMEI1318	Bme	COG0775	Nucleoside phosphorylase	Nucleotide transport and metabolism	F
BrucellaGL001609	BMEI1319	Bme	COG3021	Uncharacterized protein conserved in bacteria	Function unknown	S
BrucellaGL001610	BMEI1320	Bme	COG0644	Dehydrogenases (flavoproteins)	Energy production and conversion	C
BrucellaGL001611	BMEI1321	Bme	COG1573	Uracil-DNA glycosylase	Replication, recombination and repair	L
BrucellaGL001612	BMEI0300	Bme	COG0477	Permeases of the major facilitator superfamily	Carbohydrate transport and metabolism	G
BrucellaGL001613	BMEI1323	Bme	COG0697	Permeases of the drug/metabolite transporter (DMT) superfamily	Carbohydrate transport and metabolism	G
BrucellaGL001614	BMEI1324	Bme	COG0308	Aminopeptidase N	Amino acid transport and metabolism	E
BrucellaGL001616	BMEI1325_1	Bme	COG2202	FOG: PAS/PAC domain	Signal transduction mechanisms	T
BrucellaGL001617	BMEI1326	Bme	COG3306	Glycosyltransferase involved in LPS biosynthesis	Cell wall/membrane/envelope biogenesis	M
BrucellaGL001618	BMEI1327	Bme	COG1391	Glutamine synthetase adenylyltransferase	Posttranslational modification, protein turnover, chaperones	O
BrucellaGL001619	BMEI1328	Bme	COG0642	Signal transduction histidine kinase	Signal transduction mechanisms	T
BrucellaGL001620	BMEI1329	Bme	COG0745	Response regulators consisting of a CheY-like receiver domain and a winged-helix DNA-binding domain	Signal transduction mechanisms	T
BrucellaGL001621	BMEI1330	Bme	COG0265	Trypsin-like serine proteases, typically periplasmic, contain C-terminal PDZ domain	Posttranslational modification, protein turnover, chaperones	O
BrucellaGL001622	BMEI1331	Bme	COG3088	Uncharacterized protein involved in biosynthesis of c-type cytochromes	Posttranslational modification, protein turnover, chaperones	O
BrucellaGL001623	BMEI1332	Bme	COG1138	Cytochrome c biogenesis factor	Posttranslational modification, protein turnover, chaperones	O
BrucellaGL001624	BMEI1333	Bme	COG2332	Cytochrome c-type biogenesis protein CcmE	Posttranslational modification, protein turnover, chaperones	O
BrucellaGL001625	BMEI1334	Bme	COG4235	Cytochrome c biogenesis factor	Posttranslational modification, protein turnover, chaperones	O
BrucellaGL001626	BMEI1335	Bme	COG4520	Surface antigen	Cell wall/membrane/envelope biogenesis	M
BrucellaGL001627	BMEI1336	Bme	COG0642	Signal transduction histidine kinase	Signal transduction mechanisms	T

BrucellaGL001628	BMEI1337	Bme	COG0745	Response regulators consisting of a CheY-like receiver domain and a winged-helix DNA-binding domain	Signal transduction mechanisms	T
BrucellaGL001633	BMEI1342	Bme	COG0791	Cell wall-associated hydrolases (invasion-associated proteins)	Cell wall/membrane/envelope biogenesis	M
BrucellaGL001634	BMEI1343	Bme	COG5449	Uncharacterized conserved protein	Function unknown	S
BrucellaGL001635	BMEI1344	Bme	COG5448	Uncharacterized conserved protein	Function unknown	S
BrucellaGL001636	BMEI1345	Bme	COG5281	Phage-related minor tail protein	Function unknown	S
BrucellaGL001638	BMEI1347	Bme	COG5437	Predicted secreted protein	Function unknown	S
BrucellaGL001640	AGc1752	Atu	COG5614	Bacteriophage head-tail adaptor	General function prediction only	R
BrucellaGL001642	AGc1748	Atu	COG4653	Predicted phage phi-C31 gp36 major capsid-like protein	General function prediction only	R
BrucellaGL001644	AGc1747	Atu	COG3740	Phage head maturation protease	General function prediction only	R
BrucellaGL001645	AGc1747	Atu	COG3740	Phage head maturation protease	General function prediction only	R
BrucellaGL001647	BMEI1349	Bme	COG4695	Phage-related protein	Function unknown	S
BrucellaGL001648	BMEI1350	Bme	COG5323	Uncharacterized conserved protein	Function unknown	S
BrucellaGL001650	BMEI1351	Bme	COG0744	Membrane carboxypeptidase (penicillin-binding protein)	Cell wall/membrane/envelope biogenesis	M
BrucellaGL001651	BMEI1352	Bme	COG5402	Uncharacterized conserved protein	Function unknown	S
BrucellaGL001652	BMEI1353	Bme	COG5436	Predicted integral membrane protein	Function unknown	S
BrucellaGL001653	BMEI1354	Bme	COG5330	Uncharacterized protein conserved in bacteria	Function unknown	S
BrucellaGL001654	BMEI1355	Bme	COG5447	Uncharacterized conserved protein	Function unknown	S
BrucellaGL001655	BMEI1356	Bme	COG3409	Putative peptidoglycan-binding domain-containing protein	Cell wall/membrane/envelope biogenesis	M
BrucellaGL001656	BMEI1357	Bme	COG0642	Signal transduction histidine kinase	Signal transduction mechanisms	T
BrucellaGL001659	BMEI1359	Bme	COG2166	SufE protein probably involved in Fe-S center assembly	General function prediction only	R
BrucellaGL001660	BMEI1360	Bme	COG0154	Asp-tRNAAsn/Glu-tRNAGln amidotransferase A subunit and related amidases	Translation, ribosomal structure and biogenesis	J
BrucellaGL001662	BMEI1362	Bme	COG0593	ATPase involved in DNA replication initiation	Replication, recombination and repair	L
BrucellaGL001664	BMEI1364	Bme	COG4957	Predicted transcriptional regulator	Transcription	K
BrucellaGL001666	BMEI1365	Bme	COG1770	Protease II	Amino acid transport and metabolism	E

BrucellaGL001668	BMEI1367	Bme	COG0605	Superoxide dismutase	Inorganic ion transport and metabolism	P
BrucellaGL001669	BMEI1368	Bme	COG1011	Predicted hydrolase (HAD superfamily)	General function prediction only	R
BrucellaGL001670	BMEI1369	Bme	COG1376	Uncharacterized protein conserved in bacteria	Function unknown	S
BrucellaGL001671	BMEI1370	Bme	COG0433	Predicted ATPase	General function prediction only	R
BrucellaGL001673	BMEI1372	Bme	COG2807	Cyanate permease	Inorganic ion transport and metabolism	P
BrucellaGL001674	BMEI1373	Bme	COG2186	Transcriptional regulators	Transcription	K
BrucellaGL001675	BMEI1374	Bme	COG0598	Mg2+ and Co2+ transporters	Inorganic ion transport and metabolism	P
BrucellaGL001677	BMEI1376	Bme	COG3654	Prophage maintenance system killer protein	General function prediction only	R
BrucellaGL001678	BMEI1377	Bme	COG0782	Transcription elongation factor	Transcription	K
BrucellaGL001679	BMEI1378	Bme	COG4448	L-asparaginase II	Amino acid transport and metabolism	E
BrucellaGL001680	BMEI1379	Bme	COG1309	Transcriptional regulator	Transcription	K
BrucellaGL001681	BMEI1380	Bme	COG2303	Choline dehydrogenase and related flavoproteins	Amino acid transport and metabolism	E
BrucellaGL001682	BMEI1380	Bme	COG2303	Choline dehydrogenase and related flavoproteins	Amino acid transport and metabolism	E
BrucellaGL001683	BMEI1382	Bme	COG1012	NAD-dependent aldehyde dehydrogenases	Energy production and conversion	C
BrucellaGL001685	BMEI1384	Bme	COG2207	AraC-type DNA-binding domain-containing proteins	Transcription	K
BrucellaGL001686	BMEI1385	Bme	COG1879	ABC-type sugar transport system, periplasmic component	Carbohydrate transport and metabolism	G
BrucellaGL001687	BMEI1386	Bme	COG1070	Sugar (pentulose and hexulose) kinases	Carbohydrate transport and metabolism	G
BrucellaGL001688	BMEI1387	Bme	COG2115	Xylose isomerase	Carbohydrate transport and metabolism	G
BrucellaGL001689	BMEI1388	Bme	COG0673	Predicted dehydrogenases and related proteins	General function prediction only	R
BrucellaGL001690	BMEI1389	Bme	COG1082	Sugar phosphate isomerases/epimerases	Carbohydrate transport and metabolism	G
BrucellaGL001691	BMEI1390	Bme	COG1879	ABC-type sugar transport system, periplasmic component	Carbohydrate transport and metabolism	G
BrucellaGL001692	BMEI1391	Bme	COG1172	Ribose/xylose/arabinose/galactoside ABC-type transport systems, permease components	Carbohydrate transport and metabolism	G

BrucellaGL001693	BMEI1392	Bme	COG1129	ABC-type sugar transport system, ATPase component	Carbohydrate transport and metabolism	G
BrucellaGL001694	BMEI1392	Bme	COG1129	ABC-type sugar transport system, ATPase component	Carbohydrate transport and metabolism	G
BrucellaGL001695	BMEI1393	Bme	COG0438	Glycosyltransferase	Cell wall/membrane/envelope biogenesis	M
BrucellaGL001696	BMEI1394	Bme	COG2942	N-acyl-D-glucosamine 2-epimerase	Carbohydrate transport and metabolism	G
BrucellaGL001697	BMEI1395_1	Bme	COG0836	Mannose-1-phosphate guanylyltransferase	Cell wall/membrane/envelope biogenesis	M
BrucellaGL001698	BMEI1396	Bme	COG1109	Phosphomannomutase	Carbohydrate transport and metabolism	G
BrucellaGL001699	BMEI1397	Bme	COG2801	Transposase and inactivated derivatives	Replication, recombination and repair	L
BrucellaGL001700	BMEI1398	Bme	COG3293	Transposase and inactivated derivatives	Replication, recombination and repair	L
BrucellaGL001702	BMEI1401	Bme	COG2801	Transposase and inactivated derivatives	Replication, recombination and repair	L
BrucellaGL001704	BMEI1404	Bme	COG0438	Glycosyltransferase	Cell wall/membrane/envelope biogenesis	M
BrucellaGL001705	BMEI1052	Bme	COG3293	Transposase and inactivated derivatives	Replication, recombination and repair	L
BrucellaGL001706	BMEI1053	Bme	COG3293	Transposase and inactivated derivatives	Replication, recombination and repair	L
BrucellaGL001707	BMEI1408	Bme	COG2801	Transposase and inactivated derivatives	Replication, recombination and repair	L
BrucellaGL001709	mll5949	Mlo	COG3293	Transposase and inactivated derivatives	Replication, recombination and repair	L
BrucellaGL001710	BMEI1413	Bme	COG1089	GDP-D-mannose dehydratase	Cell wall/membrane/envelope biogenesis	M
BrucellaGL001711	BMEI1414	Bme	COG0399	Predicted pyridoxal phosphate-dependent enzyme apparently involved in regulation of cell wall biogenesis	Cell wall/membrane/envelope biogenesis	M
BrucellaGL001712	BMEI1418	Bme	COG0223	Methionyl-tRNA formyltransferase	Translation, ribosomal structure and biogenesis	J
BrucellaGL001714	mlr4020	Mlo	COG2801	Transposase and inactivated derivatives	Replication, recombination and repair	L
BrucellaGL001715	BMEI1422	Bme	COG2801	Transposase and inactivated derivatives	Replication, recombination and repair	L
BrucellaGL001716	BMEI1423	Bme	COG2801	Transposase and inactivated derivatives	Replication, recombination and repair	L

BrucellaGL001717	BMEI1426	Bme	COG0472	UDP-N-acetylmuramyl pentapeptide phosphotransferase/UDP-N-acetylglucosamine-1-phosphate transferase	Cell wall/membrane/envelope biogenesis	M
BrucellaGL001718	BMEI1427	Bme	COG1086	Predicted nucleoside-diphosphate sugar epimerases	Cell wall/membrane/envelope biogenesis	M
BrucellaGL001720	BMEI1429	Bme	COG2351	Transthyretin-like protein	General function prediction only	R
BrucellaGL001721	BMEI1430	Bme	COG3194	Ureidoglycolate hydrolase	Nucleotide transport and metabolism	F
BrucellaGL001722	BMEI1431	Bme	COG1268	Uncharacterized conserved protein	General function prediction only	R
BrucellaGL001723	BMEI1432	Bme	COG0491	Zn-dependent hydrolases, including glyoxylases	General function prediction only	R
BrucellaGL001726	BMEI1435	Bme	COG0726	Predicted xylanase/chitin deacetylase	Carbohydrate transport and metabolism	G
BrucellaGL001727	BMEI1436	Bme	COG0574	Phosphoenolpyruvate synthase/pyruvate phosphate dikinase	Carbohydrate transport and metabolism	G
BrucellaGL001728	BMEI1437	Bme	COG3324	Predicted enzyme related to lactoylglutathione lyase	General function prediction only	R
BrucellaGL001729	BMEI1438	Bme	COG1230	Co/Zn/Cd efflux system component	Inorganic ion transport and metabolism	P
BrucellaGL001730	BMEI1439	Bme	COG1196	Chromosome segregation ATPases	Cell cycle control, cell division, chromosome partitioning	D
BrucellaGL001731	BMEI1440	Bme	COG1651	Protein-disulfide isomerase	Posttranslational modification, protein turnover, chaperones	O
BrucellaGL001733	BMEI1441	Bme	COG5389	Uncharacterized protein conserved in bacteria	Function unknown	S
BrucellaGL001734	BMEI1442	Bme	COG1194	A/G-specific DNA glycosylase	Replication, recombination and repair	L
BrucellaGL001735	BMEI1443	Bme	COG1011	Predicted hydrolase (HAD superfamily)	General function prediction only	R
BrucellaGL001736	BMEI1444	Bme	COG0863	DNA modification methylase	Replication, recombination and repair	L
BrucellaGL001737	BMEI1445_1	Bme	COG0038	Chloride channel protein EriC	Inorganic ion transport and metabolism	P
BrucellaGL001738	BMEI1446	Bme	COG0637	Predicted phosphatase/phosphohexomutase	General function prediction only	R
BrucellaGL001740	BMEI1448	Bme	COG4943	Predicted signal transduction protein containing sensor and EAL domains	Signal transduction mechanisms	T
BrucellaGL001742	BMEI1450	Bme	COG0498	Threonine synthase	Amino acid transport and metabolism	E
BrucellaGL001743	BMEI1451	Bme	COG0612	Predicted Zn-dependent peptidases	General function prediction only	R

BrucellaGL001744	BMEI1452	Bme	COG1670	Acetyltransferases, including N-acetylases of ribosomal proteins	Translation, ribosomal structure and biogenesis	J
BrucellaGL001745	BMEI1453_1	Bme	COG2202	FOG: PAS/PAC domain	Signal transduction mechanisms	T
BrucellaGL001746	BMEI1454	Bme	COG1678	Putative transcriptional regulator	Transcription	K
BrucellaGL001747	BMEI1455	Bme	COG4233	Uncharacterized protein predicted to be involved in C-type cytochrome biogenesis	Posttranslational modification, protein turnover, chaperones	O
BrucellaGL001748	BMEI1456	Bme	COG0678	Peroxiredoxin	Posttranslational modification, protein turnover, chaperones	O
BrucellaGL001749	BMEI1457	Bme	COG0328	Ribonuclease HI	Replication, recombination and repair	L
BrucellaGL001750	BMEI1458	Bme	COG2334	Putative homoserine kinase type II (protein kinase fold)	General function prediction only	R
BrucellaGL001751	BMEI1459	Bme	COG0761	Penicillin tolerance protein	Lipid transport and metabolism	I
BrucellaGL001752	BMEI1460	Bme	COG3346	Uncharacterized conserved protein	Function unknown	S
BrucellaGL001753	BMEI1461	Bme	COG5349	Uncharacterized protein conserved in bacteria	Function unknown	S
BrucellaGL001754	BMEI1462	Bme	COG1845	Heme/copper-type cytochrome/quinol oxidase, subunit 3	Energy production and conversion	C
BrucellaGL001755	BMEI1463	Bme	COG3175	Cytochrome oxidase assembly factor	Posttranslational modification, protein turnover, chaperones	O
BrucellaGL001757	BMEI1464	Bme	COG0109	Polyprenyltransferase (cytochrome oxidase assembly factor)	Posttranslational modification, protein turnover, chaperones	O
BrucellaGL001758	BMEI1465	Bme	COG0843	Heme/copper-type cytochrome/quinol oxidases, subunit 1	Energy production and conversion	C
BrucellaGL001760	BMEI1466	Bme	COG1622	Heme/copper-type cytochrome/quinol oxidases, subunit 2	Energy production and conversion	C
BrucellaGL001761	BMEI1467	Bme	COG5342	Invasion protein B, involved in pathogenesis	General function prediction only	R
BrucellaGL001762	BMEI1468	Bme	COG0312	Predicted Zn-dependent proteases and their inactivated homologs	General function prediction only	R
BrucellaGL001763	BMEI1469	Bme	COG0194	Guanylate kinase	Nucleotide transport and metabolism	F
BrucellaGL001764	BMEI1470	Bme	COG1561	Uncharacterized stress-induced protein	Function unknown	S
BrucellaGL001765	BMEI1471	Bme	COG1559	Predicted periplasmic solute-binding protein	General function prediction only	R
BrucellaGL001766	BMEI1473	Bme	COG0304	3-oxoacyl-(acyl-carrier-protein) synthase	Lipid transport and metabolism	I
BrucellaGL001767	BMEI1475	Bme	COG0236	Acyl carrier protein	Lipid transport and metabolism	I

BrucellaGL001768	AGc2026	Atu	COG1028	Dehydrogenases with different specificities (related to short-chain alcohol dehydrogenases)	Lipid transport and metabolism	I
BrucellaGL001769	AGc2026	Atu	COG1028	Dehydrogenases with different specificities (related to short-chain alcohol dehydrogenases)	Lipid transport and metabolism	I
BrucellaGL001770	BMEI1478	Bme	COG0331	(acyl-carrier-protein) S-malonyltransferase	Lipid transport and metabolism	I
BrucellaGL001771	BMEI1480	Bme	COG0360	Ribosomal protein S6	Translation, ribosomal structure and biogenesis	J
BrucellaGL001772	BMEI1481	Bme	COG0238	Ribosomal protein S18	Translation, ribosomal structure and biogenesis	J
BrucellaGL001774	BMEI1483	Bme	COG0359	Ribosomal protein L9	Translation, ribosomal structure and biogenesis	J
BrucellaGL001775	BMEI1484	Bme	COG2230	Cyclopropane fatty acid synthase and related methyltransferases	Cell wall/membrane/envelope biogenesis	M
BrucellaGL001776	BMEI1485	Bme	COG0305	Replicative DNA helicase	Replication, recombination and repair	L
BrucellaGL001777	BMEI1486	Bme	COG1066	Predicted ATP-dependent serine protease	Posttranslational modification, protein turnover, chaperones	O
BrucellaGL001778	BMEI1487	Bme	COG1286	Uncharacterized membrane protein, required for colicin V production	General function prediction only	R
BrucellaGL001779	BMEI1488	Bme	COG0034	Glutamine phosphoribosylpyrophosphate amidotransferase	Nucleotide transport and metabolism	F
BrucellaGL001780	AGc1984	Atu	COG1028	Dehydrogenases with different specificities (related to short-chain alcohol dehydrogenases)	Lipid transport and metabolism	I
BrucellaGL001781	BMEI1490	Bme	COG1183	Phosphatidylserine synthase	Lipid transport and metabolism	I
BrucellaGL001782	BMEI1491	Bme	COG0688	Phosphatidylserine decarboxylase	Lipid transport and metabolism	I
BrucellaGL001783	BMEI1492	Bme	COG5265	ABC-type transport system involved in Fe-S cluster assembly, permease and ATPase components	Posttranslational modification, protein turnover, chaperones	O
BrucellaGL001784	BMEI1493	Bme	COG1652	Uncharacterized protein containing LysM domain	Function unknown	S
BrucellaGL001785	BMEI1494	Bme	COG3895	Predicted periplasmic protein	General function prediction only	R
BrucellaGL001786	BMEI1495	Bme	COG1611	Predicted Rossmann fold nucleotide-binding protein	General function prediction only	R
BrucellaGL001787	BMEI1496	Bme	COG2265	SAM-dependent methyltransferases related to tRNA (uracil-5-)-methyltransferase	Translation, ribosomal structure and biogenesis	J

BrucellaGL001788	BMEI1497	Bme	COG1189	Predicted rRNA methylase	Translation, ribosomal structure and biogenesis	J
BrucellaGL001789	BMEI1498	Bme	COG1154	Deoxyxylulose-5-phosphate synthase	Coenzyme transport and metabolism	H
BrucellaGL001790	BMEI1499	Bme	COG1741	Pirin-related protein	General function prediction only	R
BrucellaGL001791	BMEI1500	Bme	COG3753	Uncharacterized protein conserved in bacteria	Function unknown	S
BrucellaGL001792	BMEI1501	Bme	COG2261	Predicted membrane protein	Function unknown	S
BrucellaGL001793	BMEI1502	Bme	COG0586	Uncharacterized membrane-associated protein	Function unknown	S
BrucellaGL001794	BMEI1503	Bme	COG1722	Exonuclease VII small subunit	Replication, recombination and repair	L
BrucellaGL001795	BMEI1504	Bme	COG0123	Deacetylases, including yeast histone deacetylase and acetoin utilization protein	Chromatin structure and dynamics	B
BrucellaGL001796	BMEI1505_1	Bme	COG0108	3,4-dihydroxy-2-butanone 4-phosphate synthase	Coenzyme transport and metabolism	H
BrucellaGL001797	BMEI1506	Bme	COG0082	Chorismate synthase	Amino acid transport and metabolism	E
BrucellaGL001799	BMEI1508	Bme	COG2121	Uncharacterized protein conserved in bacteria	Function unknown	S
BrucellaGL001802	BMEI1510	Bme	COG1278	Cold shock proteins	Transcription	K
BrucellaGL001803	BMEI1511	Bme	COG0406	Fructose-2,6-bisphosphatase	Carbohydrate transport and metabolism	G
BrucellaGL001804	BMEI1512	Bme	COG0623	Enoyl-[acyl-carrier-protein] reductase (NADH)	Lipid transport and metabolism	I
BrucellaGL001805	BMEI1513	Bme	COG2214	DnaJ-class molecular chaperone	Posttranslational modification, protein turnover, chaperones	O
BrucellaGL001807	BMEI1517	Bme	COG0259	Pyridoxamine-phosphate oxidase	Coenzyme transport and metabolism	H
BrucellaGL001808	BMEI1518	Bme	COG0382	4-hydroxybenzoate polyprenyltransferase and related prenyltransferases	Coenzyme transport and metabolism	H
BrucellaGL001809	BMEI1519	Bme	COG0151	Phosphoribosylamine-glycine ligase	Nucleotide transport and metabolism	F
BrucellaGL001810	BMEI1520	Bme	COG2199	FOG: GGDEF domain	Signal transduction mechanisms	T
BrucellaGL001811	BMEI1521	Bme	COG1960	Acyl-CoA dehydrogenases	Lipid transport and metabolism	I
BrucellaGL001812	BMEI1522	Bme	COG1024	Enoyl-CoA hydratase/carnithine racemase	Lipid transport and metabolism	I
BrucellaGL001815	BMEI1526	Bme	COG3668	Plasmid stabilization system protein	General function prediction only	R
BrucellaGL001816	BMEI1527	Bme	COG0277	FAD/FMN-containing dehydrogenases	Energy production and conversion	C
BrucellaGL001817	BMEI1528	Bme	COG0009	Putative translation factor (SUA5)	Translation, ribosomal structure and biogenesis	J
BrucellaGL001818	BMEI1529	Bme	COG0751	Glycyl-tRNA synthetase, beta subunit	Translation, ribosomal structure and biogenesis	J

BrucellaGL001819	BMEI1530	Bme	COG0752	Glycyl-tRNA synthetase, alpha subunit	Translation, ribosomal structure and biogenesis	J
BrucellaGL001820	BMEI1531	Bme	COG0457	FOG: TPR repeat	General function prediction only	R
BrucellaGL001821	BMEI1532	Bme	COG0142	Geranylgeranyl pyrophosphate synthase	Coenzyme transport and metabolism	H
BrucellaGL001823	BMEI1534	Bme	COG4123	Predicted O-methyltransferase	General function prediction only	R
BrucellaGL001824	BMEI1535_1	Bme	COG3004	Na+/H+ antiporter	Inorganic ion transport and metabolism	P
BrucellaGL001826	BMEI1537	Bme	COG1947	4-diphosphocytidyl-2C-methyl-D-erythritol 2-phosphate synthase	Lipid transport and metabolism	I
BrucellaGL001828	BMEI1540	Bme	COG0463	Glycosyltransferases involved in cell wall biogenesis	Cell wall/membrane/envelope biogenesis	M
BrucellaGL001829	BMEI1541	Bme	COG1533	DNA repair photolyase	Replication, recombination and repair	L
BrucellaGL001830	BMEI1542	Bme	COG0164	Ribonuclease HII	Replication, recombination and repair	L
BrucellaGL001831	BMEI1543	Bme	COG0711	F0F1-type ATP synthase, subunit b	Energy production and conversion	C
BrucellaGL001832	BMEI1544	Bme	COG0711	F0F1-type ATP synthase, subunit b	Energy production and conversion	C
BrucellaGL001833	BMEI1545	Bme	COG0636	F0F1-type ATP synthase, subunit c/Archaeal/vacuolar-type H+-ATPase, subunit K	Energy production and conversion	C
BrucellaGL001834	BMEI1546	Bme	COG0356	F0F1-type ATP synthase, subunit a	Energy production and conversion	C
BrucellaGL001835	BMEI1547	Bme	COG5336	Uncharacterized protein conserved in bacteria	Function unknown	S
BrucellaGL001836	BMEI1548	Bme	COG3773	Cell wall hydrolyses involved in spore germination	Cell wall/membrane/envelope biogenesis	M
BrucellaGL001838	BMEI1549	Bme	COG0443	Molecular chaperone	Posttranslational modification, protein turnover, chaperones	O
BrucellaGL001839	BMEI1550	Bme	COG1160	Predicted GTPases	General function prediction only	R
BrucellaGL001840	BMEI1551	Bme	COG4649	Uncharacterized protein conserved in bacteria	Function unknown	S
BrucellaGL001842	BMEI1553	Bme	COG1133	ABC-type long-chain fatty acid transport system, fused permease and ATPase components	Lipid transport and metabolism	I
BrucellaGL001843	XF0268	Xfa	COG0477	Permeases of the major facilitator superfamily	Carbohydrate transport and metabolism	G
BrucellaGL001844	BMEI1556	Bme	COG4129	Predicted membrane protein	Function unknown	S
BrucellaGL001845	BMEI1557	Bme	COG1393	Arsenate reductase and related proteins, glutaredoxin family	Inorganic ion transport and metabolism	P

BrucellaGL001846	BMEI1558_1	Bme	COG2169	Adenosine deaminase	Nucleotide transport and metabolism	F
BrucellaGL001847	BMEI1559	Bme	COG1012	NAD-dependent aldehyde dehydrogenases	Energy production and conversion	C
BrucellaGL001848	BMEI1560	Bme	COG1012	NAD-dependent aldehyde dehydrogenases	Energy production and conversion	C
BrucellaGL001849	BMEI1561	Bme	COG1651	Protein-disulfide isomerase	Posttranslational modification, protein turnover, chaperones	O
BrucellaGL001850	BMEI1563	Bme	COG0735	Fe2+/Zn2+ uptake regulation proteins	Inorganic ion transport and metabolism	P
BrucellaGL001851	BMEI1564	Bme	COG3278	Cbb3-type cytochrome oxidase, subunit 1	Posttranslational modification, protein turnover, chaperones	O
BrucellaGL001852	BMEI1565	Bme	COG2993	Cbb3-type cytochrome oxidase, cytochrome c subunit	Energy production and conversion	C
BrucellaGL001853	msl6627	Mlo	COG4736	Cbb3-type cytochrome oxidase, subunit 3	Posttranslational modification, protein turnover, chaperones	O
BrucellaGL001854	BMEI1566	Bme	COG2010	Cytochrome c, mono- and diheme variants	Energy production and conversion	C
BrucellaGL001855	BMEI1566	Bme	COG2010	Cytochrome c, mono- and diheme variants	Energy production and conversion	C
BrucellaGL001856	BMEI1567	Bme	COG0348	Polyferredoxin	Energy production and conversion	C
BrucellaGL001857	BMEI1568	Bme	COG5456	Predicted integral membrane protein linked to a cation pump	Inorganic ion transport and metabolism	P
BrucellaGL001858	BMEI1569	Bme	COG2217	Cation transport ATPase	Inorganic ion transport and metabolism	P
BrucellaGL001859	msr6418	Mlo	COG3197	Uncharacterized protein, possibly involved in nitrogen fixation	Inorganic ion transport and metabolism	P
BrucellaGL001860	BMEI1570	Bme	COG2379	Putative glycerate kinase	Carbohydrate transport and metabolism	G
BrucellaGL001861	BMEI1571	Bme	COG0402	Cytosine deaminase and related metal-dependent hydrolases	Nucleotide transport and metabolism	F
BrucellaGL001862	BMEI1572	Bme	COG3748	Predicted membrane protein	Function unknown	S
BrucellaGL001863	BMEI1573	Bme	COG0583	Transcriptional regulator	Transcription	K
BrucellaGL001864	BMEI1574	Bme	COG1975	Xanthine and CO dehydrogenases maturation factor, XdhC/CoxF family	Posttranslational modification, protein turnover, chaperones	O
BrucellaGL001865	BMEI1575	Bme	COG4631	Xanthine dehydrogenase, molybdopterin-binding subunit B	Nucleotide transport and metabolism	F

BrucellaGL001866	BMEI1576	Bme	COG4630	Xanthine dehydrogenase, iron-sulfur cluster and FAD-binding subunit A	Nucleotide transport and metabolism	F
BrucellaGL001868	BMEI1577_1	Bme	COG0726	Predicted xylanase/chitin deacetylase	Carbohydrate transport and metabolism	G
BrucellaGL001869	BMEI1578	Bme	COG3257	Uncharacterized protein, possibly involved in glyoxylate utilization	General function prediction only	R
BrucellaGL001870	AGpA385	Atu	COG4663	TRAP-type mannitol/chloroaromatic compound transport system, periplasmic component	Secondary metabolites biosynthesis, transport and catabolism	Q
BrucellaGL001871	BMEI1580	Bme	COG4664	TRAP-type mannitol/chloroaromatic compound transport system, large permease component	Secondary metabolites biosynthesis, transport and catabolism	Q
BrucellaGL001872	BMEI1581	Bme	COG4665	TRAP-type mannitol/chloroaromatic compound transport system, small permease component	Secondary metabolites biosynthesis, transport and catabolism	Q
BrucellaGL001873	BMEI1582	Bme	COG2197	Response regulator containing a CheY-like receiver domain and an HTH DNA-binding domain	Signal transduction mechanisms	T
BrucellaGL001874	BMEI1583	Bme	COG4564	Signal transduction histidine kinase	Signal transduction mechanisms	T
BrucellaGL001875	BMEI1584	Bme	COG5342	Invasion protein B, involved in pathogenesis	General function prediction only	R
BrucellaGL001876	BMEI1585	Bme	COG0534	Na+-driven multidrug efflux pump	Defense mechanisms	V
BrucellaGL001878	BMEI1586	Bme	COG3938	Proline racemase	Amino acid transport and metabolism	E
BrucellaGL001879	BMEI1588	Bme	COG1748	Saccharopine dehydrogenase and related proteins	Amino acid transport and metabolism	E
BrucellaGL001881	BMEI1589	Bme	COG0019	Diaminopimelate decarboxylase	Amino acid transport and metabolism	E
BrucellaGL001883	BMEI1591	Bme	COG1018	Flavodoxin reductases (ferredoxin-NADPH reductases) family 1	Energy production and conversion	C
BrucellaGL001884	BMEI1592	Bme	COG0413	Ketopantoate hydroxymethyltransferase	Coenzyme transport and metabolism	H
BrucellaGL001885	BMEI1593	Bme	COG0414	Panthothenate synthetase	Coenzyme transport and metabolism	H
BrucellaGL001886	BMEI1594	Bme	COG4782	Uncharacterized protein conserved in bacteria	Function unknown	S
BrucellaGL001887	BMEI1596	Bme	COG1346	Putative effector of murein hydrolase	Cell wall/membrane/envelope biogenesis	M
BrucellaGL001888	BMEI1597	Bme	COG1380	Putative effector of murein hydrolase LrgA	General function prediction only	R
BrucellaGL001889	SA2330	Sau	COG0583	Transcriptional regulator	Transcription	K
BrucellaGL001893	BMEI1602	Bme	COG3306	Glycosyltransferase involved in LPS biosynthesis	Cell wall/membrane/envelope biogenesis	M

BrucellaGL001894	BMEI1603	Bme	COG0726	Predicted xylanase/chitin deacetylase	Carbohydrate transport and metabolism	G
BrucellaGL001895	BMEI1604	Bme	COG0156	7-keto-8-aminopelargonate synthetase and related enzymes	Coenzyme transport and metabolism	H
BrucellaGL001896	BMEI1605	Bme	COG1970	Large-conductance mechanosensitive channel	Cell wall/membrane/envelope biogenesis	M
BrucellaGL001898	BMEI1606_1	Bme	COG0591	Na+/proline symporter	Amino acid transport and metabolism	E
BrucellaGL001899	BMEI1607	Bme	COG2197	Response regulator containing a CheY-like receiver domain and an HTH DNA-binding domain	Signal transduction mechanisms	T
BrucellaGL001900	BMEI1608	Bme	COG0596	Predicted hydrolases or acyltransferases (alpha/beta hydrolase superfamily)	General function prediction only	R
BrucellaGL001902	BMEI1610	Bme	COG3502	Uncharacterized protein conserved in bacteria	Function unknown	S
BrucellaGL001903	BMEI1611	Bme	COG0167	Dihydroorotate dehydrogenase	Nucleotide transport and metabolism	F
BrucellaGL001904	BMEI1612	Bme	COG0534	Na+-driven multidrug efflux pump	Defense mechanisms	V
BrucellaGL001905	BMEI1613	Bme	COG2340	Uncharacterized protein with SCP/PR1 domains	Function unknown	S
BrucellaGL001907	BMEI1615	Bme	COG4977	Transcriptional regulator containing an amidase domain and an AraC-type DNA-binding HTH domain	Transcription	K
BrucellaGL001908	BMEI1616	Bme	COG0717	Deoxycytidine deaminase	Nucleotide transport and metabolism	F
BrucellaGL001909	BMEI1617	Bme	COG0626	Cystathionine beta-lyases/cystathionine gamma-synthases	Amino acid transport and metabolism	E
BrucellaGL001910	BMEI1618	Bme	COG2967	Uncharacterized protein affecting Mg2+/Co2+ transport	Inorganic ion transport and metabolism	P
BrucellaGL001911	BMEI1619	Bme	COG1281	Disulfide bond chaperones of the HSP33 family	Posttranslational modification, protein turnover, chaperones	O
BrucellaGL001912	BMEI1620	Bme	COG0078	Ornithine carbamoyltransferase	Amino acid transport and metabolism	E
BrucellaGL001913	BMEI1621	Bme	COG4992	Ornithine/acetylornithine aminotransferase	Amino acid transport and metabolism	E
BrucellaGL001914	BMEI1623	Bme	COG5352	Uncharacterized protein conserved in bacteria	Function unknown	S
BrucellaGL001915	CC0289	Ccr	COG0642	Signal transduction histidine kinase	Signal transduction mechanisms	T
BrucellaGL001916	BMEI1626	Bme	COG1215	Glycosyltransferases, probably involved in cell wall biogenesis	Cell wall/membrane/envelope biogenesis	M

BrucellaGL001918	BMEI1627	Bme	COG0834	ABC-type amino acid transport/signal transduction systems, periplasmic component/domain	Amino acid transport and metabolism	E
BrucellaGL001921	BMEI1629	Bme	COG0841	Cation/multidrug efflux pump	Defense mechanisms	V
BrucellaGL001922	BMEI1630	Bme	COG0845	Membrane-fusion protein	Cell wall/membrane/envelope biogenesis	M
BrucellaGL001923	BMEI1631	Bme	COG1309	Transcriptional regulator	Transcription	K
BrucellaGL001924	BMEI1632	Bme	COG0318	Acyl-CoA synthetases (AMP-forming)/AMP-acid ligases II	Lipid transport and metabolism	I
BrucellaGL001925	BMEI1633	Bme	COG4757	Predicted alpha/beta hydrolase	General function prediction only	R
BrucellaGL001926	BMEI1634	Bme	COG0546	Predicted phosphatases	General function prediction only	R
BrucellaGL001927	BMEI1635	Bme	COG3608	Predicted deacylase	General function prediction only	R
BrucellaGL001928	BMEI1636	Bme	COG0166	Glucose-6-phosphate isomerase	Carbohydrate transport and metabolism	G
BrucellaGL001929	BMEI1637	Bme	COG3427	Uncharacterized conserved protein	Function unknown	S
BrucellaGL001930	BMEI1638	Bme	COG0493	NADPH-dependent glutamate synthase beta chain and related oxidoreductases	Amino acid transport and metabolism	E
BrucellaGL001931	BMEI1639_1	Bme	COG0167	Dihydroorotate dehydrogenase	Nucleotide transport and metabolism	F
BrucellaGL001932	AGc4330	Atu	COG0494	NTP pyrophosphohydrolases including oxidative damage repair enzymes	Replication, recombination and repair	L
BrucellaGL001933	BMEI1641	Bme	COG1309	Transcriptional regulator	Transcription	K
BrucellaGL001934	BMEI1642	Bme	COG1309	Transcriptional regulator	Transcription	K
BrucellaGL001935	BMEI1643	Bme	COG0624	Acetylornithine deacetylase/Succinyl-diaminopimelate desuccinylase and related deacylases	Amino acid transport and metabolism	E
BrucellaGL001936	BMEI1644	Bme	COG0044	Dihydroorotase and related cyclic amidohydrolases	Nucleotide transport and metabolism	F
BrucellaGL001937	BMEI1645	Bme	COG0841	Cation/multidrug efflux pump	Defense mechanisms	V
BrucellaGL001938	BMEI1646	Bme	COG0845	Membrane-fusion protein	Cell wall/membrane/envelope biogenesis	M
BrucellaGL001939	CC0530	Ccr	COG0642	Signal transduction histidine kinase	Signal transduction mechanisms	T

BrucellaGL001940	BMEI1649	Bme	COG0378	Ni2+-binding GTPase involved in regulation of expression and maturation of urease and hydrogenase	Posttranslational modification, protein turnover, chaperones	O
BrucellaGL001941	BMEI1650	Bme	COG0830	Urease accessory protein UreF	Posttranslational modification, protein turnover, chaperones	O
BrucellaGL001942	BMEI1651	Bme	COG2371	Urease accessory protein UreE	Posttranslational modification, protein turnover, chaperones	O
BrucellaGL001943	BMEI1652	Bme	COG0804	Urea amidohydrolase (urease) alpha subunit	Amino acid transport and metabolism	E
BrucellaGL001944	BMEI1653	Bme	COG0832	Urea amidohydrolase (urease) beta subunit	Amino acid transport and metabolism	E
BrucellaGL001946	SMc01941	Sme	COG0831	Urea amidohydrolase (urease) gamma subunit	Amino acid transport and metabolism	E
BrucellaGL001947	BMEI1655	Bme	COG0829	Urease accessory protein UreH	Posttranslational modification, protein turnover, chaperones	O
BrucellaGL001949	BMEI0197	Bme	COG0739	Membrane proteins related to metalloendopeptidases	Cell wall/membrane/envelope biogenesis	M
BrucellaGL001950	BMEI0196_2	Bme	COG1376	Uncharacterized protein conserved in bacteria	Function unknown	S
BrucellaGL001951	BMEI0195	Bme	COG0542	ATPases with chaperone activity, ATP-binding subunit	Posttranslational modification, protein turnover, chaperones	O
BrucellaGL001953	BMEI0192	Bme	COG2890	Methylase of polypeptide chain release factors	Translation, ribosomal structure and biogenesis	J
BrucellaGL001954	BMEI0191	Bme	COG0216	Protein chain release factor A	Translation, ribosomal structure and biogenesis	J
BrucellaGL001955	BMEI0190	Bme	COG3605	Signal transduction protein containing GAF and PtsI domains	Signal transduction mechanisms	T
BrucellaGL001956	BMEI0189	Bme	COG0527	Aspartokinases	Amino acid transport and metabolism	E
BrucellaGL001957	BMEI0188	Bme	COG2227	2-polyprenyl-3-methyl-5-hydroxy-6-metoxy-1,4-benzoquinol methylase	Coenzyme transport and metabolism	H
BrucellaGL001958	BMEI0187	Bme	COG0697	Permeases of the drug/metabolite transporter (DMT) superfamily	Carbohydrate transport and metabolism	G
BrucellaGL001959	BMEI0186	Bme	COG5319	Uncharacterized protein conserved in bacteria	Function unknown	S
BrucellaGL001960	BMEI0185	Bme	COG0388	Predicted amidohydrolase	General function prediction only	R
BrucellaGL001961	BMEI0184	Bme	COG0695	Glutaredoxin and related proteins	Posttranslational modification, protein turnover, chaperones	O
BrucellaGL001962	BMEI0183	Bme	COG1040	Predicted amidophosphoribosyltransferases	General function prediction only	R

BrucellaGL001963	BMEI0182	Bme	COG0500	SAM-dependent methyltransferases	Secondary metabolites biosynthesis, transport and catabolism	Q
BrucellaGL001964	BMEI0181	Bme	COG0477	Permeases of the major facilitator superfamily	Carbohydrate transport and metabolism	G
BrucellaGL001965	BMEI0180	Bme	COG4103	Uncharacterized protein conserved in bacteria	Function unknown	S
BrucellaGL001966	BMEI0179	Bme	COG3898	Uncharacterized membrane-bound protein	Function unknown	S
BrucellaGL001967	BMEI0178	Bme	COG4223	Uncharacterized protein conserved in bacteria	Function unknown	S
BrucellaGL001968	BMEI0177	Bme	COG1587	Uroporphyrinogen-III synthase	Coenzyme transport and metabolism	H
BrucellaGL001969	BMEI0176	Bme	COG0181	Porphobilinogen deaminase	Coenzyme transport and metabolism	H
BrucellaGL001970	BMEI0175	Bme	COG0533	Metal-dependent proteases with possible chaperone activity	Posttranslational modification, protein turnover, chaperones	O
BrucellaGL001971	BMEI0174	Bme	COG0240	Glycerol-3-phosphate dehydrogenase	Energy production and conversion	C
BrucellaGL001972	BMEI0173	Bme	COG2350	Uncharacterized protein conserved in bacteria	Function unknown	S
BrucellaGL001973	BMEI0172	Bme	COG2947	Uncharacterized conserved protein	Function unknown	S
BrucellaGL001974	BMEI0171	Bme	COG3897	Predicted methyltransferase	General function prediction only	R
BrucellaGL001975	BMEI0170	Bme	COG0697	Permeases of the drug/metabolite transporter (DMT) superfamily	Carbohydrate transport and metabolism	G
BrucellaGL001976	BMEI0169	Bme	COG1167	Transcriptional regulators containing a DNA-binding HTH domain and an aminotransferase domain (MocR family) and their eukaryotic orthologs	Transcription	K
BrucellaGL001977	BMEI0168	Bme	COG1674	DNA segregation ATPase FtsK/SpoIIIE and related proteins	Cell cycle control, cell division, chromosome partitioning	D
BrucellaGL001978	BMEI0167	Bme	COG0004	Ammonia permease	Inorganic ion transport and metabolism	P
BrucellaGL001980	BMEI0166	Bme	COG1946	Acyl-CoA thioesterase	Lipid transport and metabolism	I
BrucellaGL001981	BMEI0165	Bme	COG0654	2-polyprenyl-6-methoxyphenol hydroxylase and related FAD-dependent oxidoreductases	Coenzyme transport and metabolism	H
BrucellaGL001982	BMEI0164	Bme	COG0346	Lactoylglutathione lyase and related lyases	Amino acid transport and metabolism	E
BrucellaGL001983	BMEI0162	Bme	COG0479	Succinate dehydrogenase/fumarate reductase, Fe-S protein subunit	Energy production and conversion	C
BrucellaGL001984	BMEI0161	Bme	COG1053	Succinate dehydrogenase/fumarate reductase, flavoprotein subunit	Energy production and conversion	C

BrucellaGL001985	BMEI0160	Bme	COG2142	Succinate dehydrogenase, hydrophobic anchor subunit	Energy production and conversion	C
BrucellaGL001986	BMEI0159	Bme	COG2009	Succinate dehydrogenase/fumarate reductase, cytochrome b subunit	Energy production and conversion	C
BrucellaGL001987	BMEI0158	Bme	COG0454	Histone acetyltransferase HPA2 and related acetyltransferases	Transcription	K
BrucellaGL001988	BMEI0157	Bme	COG0065	3-isopropylmalate dehydratase large subunit	Amino acid transport and metabolism	E
BrucellaGL001990	BMEI0156	Bme	COG0335	Ribosomal protein L19	Translation, ribosomal structure and biogenesis	J
BrucellaGL001991	SMc00922	Sme	COG1953	Cytosine/uracil/thiamine/allantoin permeases	Nucleotide transport and metabolism	F
BrucellaGL001992	BMEI0155	Bme	COG1953	Cytosine/uracil/thiamine/allantoin permeases	Nucleotide transport and metabolism	F
BrucellaGL001993	BMEI0154	Bme	COG0730	Predicted permeases	General function prediction only	R
BrucellaGL001995	BMEI0152	Bme	COG2329	Uncharacterized enzyme involved in biosynthesis of extracellular polysaccharides	General function prediction only	R
BrucellaGL001997	BMEI0150	Bme	COG0640	Predicted transcriptional regulators	Transcription	K
BrucellaGL001998	BMEI0149	Bme	COG0336	tRNA-(guanine-N1)-methyltransferase	Translation, ribosomal structure and biogenesis	J
BrucellaGL001999	BMEI0148	Bme	COG0806	RimM protein, required for 16S rRNA processing	Translation, ribosomal structure and biogenesis	J
BrucellaGL002000	BMEI0147	Bme	COG0582	Integrase	Replication, recombination and repair	L
BrucellaGL002001	BMEI0146	Bme	COG3735	Uncharacterized protein conserved in bacteria	Function unknown	S
BrucellaGL002002	BMEI0145	Bme	COG1249	Pyruvate/2-oxoglutarate dehydrogenase complex, dihydrolipoamide dehydrogenase (E3) component, and related enzymes	Energy production and conversion	C
BrucellaGL002004	BMEI0143	Bme	COG1280	Putative threonine efflux protein	Amino acid transport and metabolism	E
BrucellaGL002005	BMEI0142	Bme	COG3686	Predicted membrane protein	Function unknown	S
BrucellaGL002006	BMEI0141	Bme	COG0508	Pyruvate/2-oxoglutarate dehydrogenase complex, dihydrolipoamide acyltransferase (E2) component, and related enzymes	Energy production and conversion	C
BrucellaGL002007	BMEI0140	Bme	COG0567	2-oxoglutarate dehydrogenase complex, dehydrogenase (E1) component, and related enzymes	Energy production and conversion	C
BrucellaGL002009	BMEI0139	Bme	COG0074	Succinyl-CoA synthetase, alpha subunit	Energy production and conversion	C

BrucellaGL002010	BMEI0138	Bme	COG0045	Succinyl-CoA synthetase, beta subunit	Energy production and conversion	C
BrucellaGL002011	BMEI0137	Bme	COG0039	Malate/lactate dehydrogenases	Energy production and conversion	C
BrucellaGL002012	BMEI0136	Bme	COG1485	Predicted ATPase	General function prediction only	R
BrucellaGL002015	BMEI0133	Bme	COG0253	Diaminopimelate epimerase	Amino acid transport and metabolism	E
BrucellaGL002016	BMEI0132	Bme	COG0621	2-methylthioadenine synthetase	Translation, ribosomal structure and biogenesis	J
BrucellaGL002017	BMEI0131	Bme	COG0552	Signal recognition particle GTPase	Intracellular trafficking, secretion, and vesicular transport	U
BrucellaGL002018	BMEI0130	Bme	COG2917	Intracellular septation protein A	Cell cycle control, cell division, chromosome partitioning	D
BrucellaGL002019	BMEI0129	Bme	COG0491	Zn-dependent hydrolases, including glyoxylases	General function prediction only	R
BrucellaGL002022	BMEI0126	Bme	COG0494	NTP pyrophosphohydrolases including oxidative damage repair enzymes	Replication, recombination and repair	L
BrucellaGL002023	BMEI0125	Bme	COG0454	Histone acetyltransferase HPA2 and related acetyltransferases	Transcription	K
BrucellaGL002024	BMEI0124	Bme	COG1364	N-acetylglutamate synthase (N-acetylornithine aminotransferase)	Amino acid transport and metabolism	E
BrucellaGL002027	BMEI0123	Bme	COG0760	Parvulin-like peptidyl-prolyl isomerase	Posttranslational modification, protein turnover, chaperones	O
BrucellaGL002028	BMEI0121	Bme	COG0653	Preprotein translocase subunit SecA (ATPase, RNA helicase)	Intracellular trafficking, secretion, and vesicular transport	U
BrucellaGL002031	BMEI0118	Bme	COG2194	Predicted membrane-associated, metal-dependent hydrolase	General function prediction only	R
BrucellaGL002032	BMEI0116	Bme	COG0583	Transcriptional regulator	Transcription	K
BrucellaGL002033	BMEI0115	Bme	COG0834	ABC-type amino acid transport/signal transduction systems, periplasmic component/domain	Amino acid transport and metabolism	E
BrucellaGL002034	BMEI0114	Bme	COG0834	ABC-type amino acid transport/signal transduction systems, periplasmic component/domain	Amino acid transport and metabolism	E
BrucellaGL002035	BMEI0113	Bme	COG0765	ABC-type amino acid transport system, permease component	Amino acid transport and metabolism	E

BrucellaGL002036	BMEI0112	Bme	COG0765	ABC-type amino acid transport system, permease component	Amino acid transport and metabolism	E
BrucellaGL002037	BMEI0111	Bme	COG1126	ABC-type polar amino acid transport system, ATPase component	Amino acid transport and metabolism	E
BrucellaGL002038	BMEI0110	Bme	COG0010	Arginase/agmatinase/formimionoglutamate hydrolase, arginase family	Amino acid transport and metabolism	E
BrucellaGL002039	BMEI0109	Bme	COG1027	Aspartate ammonia-lyase	Amino acid transport and metabolism	E
BrucellaGL002040	BMEI0108	Bme	COG1126	ABC-type polar amino acid transport system, ATPase component	Amino acid transport and metabolism	E
BrucellaGL002041	BMEI0107	Bme	COG0252	L-asparaginase/archaeal Glu-tRNAGln amidotransferase subunit D	Amino acid transport and metabolism	E
BrucellaGL002042	BMEI0106	Bme	COG1802	Transcriptional regulators	Transcription	K
BrucellaGL002043	BMEI0105	Bme	COG0252	L-asparaginase/archaeal Glu-tRNAGln amidotransferase subunit D	Amino acid transport and metabolism	E
BrucellaGL002044	BMEI0104	Bme	COG0477	Permeases of the major facilitator superfamily	Carbohydrate transport and metabolism	G
BrucellaGL002046	BMEI0103	Bme	COG0626	Cystathionine beta-lyases/cystathionine gamma-synthases	Amino acid transport and metabolism	E
BrucellaGL002047	BMEI0102	Bme	COG0589	Universal stress protein UspA and related nucleotide-binding proteins	Signal transduction mechanisms	T
BrucellaGL002048	BMEI0101	Bme	COG0031	Cysteine synthase	Amino acid transport and metabolism	E
BrucellaGL002050	BMEI0099	Bme	COG1250	3-hydroxyacyl-CoA dehydrogenase	Lipid transport and metabolism	I
BrucellaGL002051	BMEI0097	Bme	COG2025	Electron transfer flavoprotein, alpha subunit	Energy production and conversion	C
BrucellaGL002052	BMEI0096	Bme	COG2086	Electron transfer flavoprotein, beta subunit	Energy production and conversion	C
BrucellaGL002053	BMEI0095	Bme	COG0603	Predicted PP-loop superfamily ATPase	General function prediction only	R
BrucellaGL002054	BMEI0094	Bme	COG0720	6-pyruvoyl-tetrahydropterin synthase	Coenzyme transport and metabolism	H
BrucellaGL002055	BMEI0093	Bme	COG0602	Organic radical activating enzymes	Posttranslational modification, protein turnover, chaperones	O
BrucellaGL002056	BMEI0092	Bme	COG2096	Uncharacterized conserved protein	Function unknown	S
BrucellaGL002058	BMEI0090	Bme	COG1028	Dehydrogenases with different specificities (related to short-chain alcohol dehydrogenases)	Lipid transport and metabolism	I
BrucellaGL002059	BMEI0089	Bme	COG1295	Predicted membrane protein	Function unknown	S
BrucellaGL002060	BMEI0088	Bme	COG1671	Uncharacterized protein conserved in bacteria	Function unknown	S

BrucellaGL002061	BMEI0087	Bme	COG0526	Thiol-disulfide isomerase and thioredoxins	Posttranslational modification, protein turnover, chaperones	O
BrucellaGL002062	BMEI0086	Bme	COG0165	Argininosuccinate lyase	Amino acid transport and metabolism	E
BrucellaGL002063	BMEI0085	Bme	COG5567	Predicted small periplasmic lipoprotein	Cell motility	N
BrucellaGL002064	BMEI0084	Bme	COG0019	Diaminopimelate decarboxylase	Amino acid transport and metabolism	E
BrucellaGL002066	BMEI0082	Bme	COG0634	Hypoxanthine-guanine phosphoribosyltransferase	Nucleotide transport and metabolism	F
BrucellaGL002067	BMEI0081	Bme	COG0500	SAM-dependent methyltransferases	Secondary metabolites biosynthesis, transport and catabolism	Q
BrucellaGL002068	BMEI0080	Bme	COG0079	Histidinol-phosphate/aromatic aminotransferase and cobyric acid decarboxylase	Amino acid transport and metabolism	E
BrucellaGL002069	BMEI0079	Bme	COG0287	Prephenate dehydrogenase	Amino acid transport and metabolism	E
BrucellaGL002070	BMEI0078	Bme	COG4093	Uncharacterized protein conserved in bacteria	Function unknown	S
BrucellaGL002071	BMEI0077	Bme	COG3703	Uncharacterized protein involved in cation transport	Inorganic ion transport and metabolism	P
BrucellaGL002072	AGc4820	Atu	COG0762	Predicted integral membrane protein	Function unknown	S
BrucellaGL002074	BMEI0076	Bme	COG0221	Inorganic pyrophosphatase	Energy production and conversion	C
BrucellaGL002075	BMEI0075	Bme	COG0204	1-acyl-sn-glycerol-3-phosphate acyltransferase	Lipid transport and metabolism	I
BrucellaGL002076	BMEI0074	Bme	COG1434	Uncharacterized conserved protein	Function unknown	S
BrucellaGL002077	BMEI0073	Bme	COG2177	Cell division protein	Cell cycle control, cell division, chromosome partitioning	D
BrucellaGL002078	BMEI0072	Bme	COG2884	Predicted ATPase involved in cell division	Cell cycle control, cell division, chromosome partitioning	D
BrucellaGL002080	BMEI0070	Bme	COG0580	Glycerol uptake facilitator and related permeases (Major Intrinsic Protein Family)	Carbohydrate transport and metabolism	G
BrucellaGL002081	BMEI0069	Bme	COG2834	Outer membrane lipoprotein-sorting protein	Cell wall/membrane/envelope biogenesis	M
BrucellaGL002082	BMEI0068	Bme	COG0708	Exonuclease III	Replication, recombination and repair	L
BrucellaGL002083	BMEI0067	Bme	COG0664	cAMP-binding proteins - catabolite gene activator and regulatory subunit of cAMP-dependent protein kinases	Signal transduction mechanisms	T
BrucellaGL002084	BMEI0066	Bme	COG0745	Response regulators consisting of a CheY-like receiver domain and a winged-helix DNA-binding domain	Signal transduction mechanisms	T

BrucellaGL002085	BMEI0065	Bme	COG3786	Uncharacterized protein conserved in bacteria	Function unknown	S
BrucellaGL002090	BMEI0058	Bme	COG3468	Type V secretory pathway, adhesin AidA	Cell wall/membrane/envelope biogenesis	M
BrucellaGL002091	BMEI0058	Bme	COG3468	Type V secretory pathway, adhesin AidA	Cell wall/membrane/envelope biogenesis	M
BrucellaGL002092	BMEI0057	Bme	COG1738	Uncharacterized conserved protein	Function unknown	S
BrucellaGL002093	BMEI0056	Bme	COG0227	Ribosomal protein L28	Translation, ribosomal structure and biogenesis	J
BrucellaGL002095	BMEI0054	Bme	COG0789	Predicted transcriptional regulators	Transcription	K
BrucellaGL002096	BMEI0053	Bme	COG2217	Cation transport ATPase	Inorganic ion transport and metabolism	P
BrucellaGL002099	BMEI0050	Bme	COG4547	Cobalamin biosynthesis protein CobT (nicotinate-mononucleotide:5, 6-dimethylbenzimidazole phosphoribosyltransferase)	Coenzyme transport and metabolism	H
BrucellaGL002100	BMEI0049	Bme	COG0714	MoxR-like ATPases	General function prediction only	R
BrucellaGL002102	BMEI0047	Bme	COG2214	DnaJ-class molecular chaperone	Posttranslational modification, protein turnover, chaperones	O
BrucellaGL002103	BMEI0045	Bme	COG0271	Stress-induced morphogen (activity unknown)	Signal transduction mechanisms	T
BrucellaGL002104	BMEI0044	Bme	COG4536	Putative Mg2+ and Co2+ transporter CorB	Inorganic ion transport and metabolism	P
BrucellaGL002105	BMEI0043	Bme	COG0337	3-dehydroquinate synthetase	Amino acid transport and metabolism	E
BrucellaGL002106	BMEI0042	Bme	COG0703	Shikimate kinase	Amino acid transport and metabolism	E
BrucellaGL002108	BMEI0040	Bme	COG4974	Site-specific recombinase XerD	Replication, recombination and repair	L
BrucellaGL002109	BMEI0039	Bme	COG0825	Acetyl-CoA carboxylase alpha subunit	Lipid transport and metabolism	I
BrucellaGL002110	BMEI0038	Bme	COG3034	Uncharacterized protein conserved in bacteria	Function unknown	S
BrucellaGL002111	BMEI0037	Bme	COG0425	Predicted redox protein, regulator of disulfide bond formation	Posttranslational modification, protein turnover, chaperones	O
BrucellaGL002112	BMEI0036	Bme	COG0523	Putative GTPases (G3E family)	General function prediction only	R
BrucellaGL002113	BMEI0035	Bme	COG1686	D-alanyl-D-alanine carboxypeptidase	Cell wall/membrane/envelope biogenesis	M
BrucellaGL002114	BMEI0034	Bme	COG1473	Metal-dependent amidase/aminoacylase/carboxypeptidase	General function prediction only	R

BrucellaGL002115	BMEI0033	Bme	COG1473	Metal-dependent amidase/aminoacylase/carboxypeptidase	General function prediction only	R
BrucellaGL002116	BMEI0032	Bme	COG1028	Dehydrogenases with different specificities (related to short-chain alcohol dehydrogenases)	Lipid transport and metabolism	I
BrucellaGL002117	BMEI0031	Bme	COG3791	Uncharacterized conserved protein	Function unknown	S
BrucellaGL002119	BMEI0029	Bme	COG2303	Choline dehydrogenase and related flavoproteins	Amino acid transport and metabolism	E
BrucellaGL002120	SMc04390	Sme	COG2303	Choline dehydrogenase and related flavoproteins	Amino acid transport and metabolism	E
BrucellaGL002121	BMEI0026	Bme	COG1028	Dehydrogenases with different specificities (related to short-chain alcohol dehydrogenases)	Lipid transport and metabolism	I
BrucellaGL002122	BMEI0025	Bme	COG2303	Choline dehydrogenase and related flavoproteins	Amino acid transport and metabolism	E
BrucellaGL002123	BMEI0024	Bme	COG1012	NAD-dependent aldehyde dehydrogenases	Energy production and conversion	C
BrucellaGL002124	BMEI0023	Bme	COG1024	Enoyl-CoA hydratase/carnithine racemase	Lipid transport and metabolism	I
BrucellaGL002125	BMEI0021	Bme	COG4670	Acyl CoA:acetate/3-ketoacid CoA transferase	Lipid transport and metabolism	I
BrucellaGL002126	BMEI0020	Bme	COG0673	Predicted dehydrogenases and related proteins	General function prediction only	R
BrucellaGL002127	BMEI0019	Bme	COG1609	Transcriptional regulators	Transcription	K
BrucellaGL002129	BMEI0017	Bme	COG2141	Coenzyme F420-dependent N5,N10-methylene tetrahydromethanopterin reductase and related flavin-dependent oxidoreductases	Energy production and conversion	C
BrucellaGL002130	BMEI0015	Bme	COG2984	ABC-type uncharacterized transport system, periplasmic component	General function prediction only	R
BrucellaGL002131	BMEI0013	Bme	COG4120	ABC-type uncharacterized transport system, permease component	General function prediction only	R
BrucellaGL002132	BMEI0012	Bme	COG1101	ABC-type uncharacterized transport system, ATPase component	General function prediction only	R
BrucellaGL002133	BMEI0011	Bme	COG1466	DNA polymerase III, delta subunit	Replication, recombination and repair	L
BrucellaGL002134	BMEI0010	Bme	COG1475	Predicted transcriptional regulators	Transcription	K
BrucellaGL002135	BMEI0009	Bme	COG1192	ATPases involved in chromosome partitioning	Cell cycle control, cell division, chromosome partitioning	D
BrucellaGL002136	BMEI0008	Bme	COG0357	Predicted S-adenosylmethionine-dependent methyltransferase involved in bacterial cell division	Cell wall/membrane/envelope biogenesis	M
BrucellaGL002137	BMEI0007	Bme	COG0445	NAD/FAD-utilizing enzyme apparently involved in cell division	Cell cycle control, cell division, chromosome partitioning	D

BrucellaGL002138	BMEI0006	Bme	COG0486	Predicted GTPase	General function prediction only	R
BrucellaGL002139	BMEI0005	Bme	COG1331	Highly conserved protein containing a thioredoxin domain	Posttranslational modification, protein turnover, chaperones	O
BrucellaGL002140	BMEI0004	Bme	COG1331	Highly conserved protein containing a thioredoxin domain	Posttranslational modification, protein turnover, chaperones	O
BrucellaGL002141	BMEI0003	Bme	COG1158	Transcription termination factor	Transcription	K
BrucellaGL002142	BMEI0002	Bme	COG1981	Predicted membrane protein	Function unknown	S
BrucellaGL002143	BMEI0001	Bme	COG0407	Uroporphyrinogen-III decarboxylase	Coenzyme transport and metabolism	H
BrucellaGL002145	BMEI2060	Bme	COG1806	Uncharacterized protein conserved in bacteria	Function unknown	S
BrucellaGL002146	BMEI2059	Bme	COG0424	Nucleotide-binding protein implicated in inhibition of septum formation	Cell cycle control, cell division, chromosome partitioning	D
BrucellaGL002147	BMEI2058	Bme	COG0169	Shikimate 5-dehydrogenase	Amino acid transport and metabolism	E
BrucellaGL002148	BMEI2057	Bme	COG0237	Dephospho-CoA kinase	Coenzyme transport and metabolism	H
BrucellaGL002149	BMEI2056	Bme	COG0847	DNA polymerase III, epsilon subunit and related 3'-5' exonucleases	Replication, recombination and repair	L
BrucellaGL002150	BMEI2055	Bme	COG1952	Preprotein translocase subunit SecB	Intracellular trafficking, secretion, and vesicular transport	U
BrucellaGL002151	BMEI2054	Bme	COG3030	Protein affecting phage T7 exclusion by the F plasmid	General function prediction only	R
BrucellaGL002153	BMEI2053	Bme	COG4395	Uncharacterized protein conserved in bacteria	Function unknown	S
BrucellaGL002154	BMEI2052	Bme	COG2821	Membrane-bound lytic murein transglycosylase	Cell wall/membrane/envelope biogenesis	M
BrucellaGL002155	BMEI2051	Bme	COG2840	Uncharacterized protein conserved in bacteria	Function unknown	S
BrucellaGL002156	BMEI2050	Bme	COG1396	Predicted transcriptional regulators	Transcription	K
BrucellaGL002158	BMEI2048	Bme	COG1220	ATP-dependent protease HslVU (ClpYQ), ATPase subunit	Posttranslational modification, protein turnover, chaperones	O
BrucellaGL002159	BMEI2047	Bme	COG5405	ATP-dependent protease HslVU (ClpYQ), peptidase subunit	Posttranslational modification, protein turnover, chaperones	O
BrucellaGL002160	BMEI2045	Bme	COG0131	Imidazoleglycerol-phosphate dehydratase	Amino acid transport and metabolism	E
BrucellaGL002162	BMEI2043	Bme	COG0118	Glutamine amidotransferase	Amino acid transport and metabolism	E
BrucellaGL002163	BMEI2042	Bme	COG0106	Phosphoribosylformimino-5-aminoimidazole carboxamide ribonucleotide (ProFAR) isomerase	Amino acid transport and metabolism	E

BrucellaGL002164	BMEI2041	Bme	COG0107	Imidazoleglycerol-phosphate synthase	Amino acid transport and metabolism	E
BrucellaGL002165	BMEI2040	Bme	COG0140	Phosphoribosyl-ATP pyrophosphohydrolase	Amino acid transport and metabolism	E
BrucellaGL002166	BMEI2039	Bme	COG1072	Panthothenate kinase	Coenzyme transport and metabolism	H
BrucellaGL002167	BMEI2038	Bme	COG1186	Protein chain release factor B	Translation, ribosomal structure and biogenesis	J
BrucellaGL002168	BMEI2037	Bme	COG1866	Phosphoenolpyruvate carboxykinase (ATP)	Energy production and conversion	C
BrucellaGL002169	BMEI2037	Bme	COG1866	Phosphoenolpyruvate carboxykinase (ATP)	Energy production and conversion	C
BrucellaGL002170	BMEI2036	Bme	COG0745	Response regulators consisting of a CheY-like receiver domain and a winged-helix DNA-binding domain	Signal transduction mechanisms	T
BrucellaGL002171	CC0238	Ccr	COG0642	Signal transduction histidine kinase	Signal transduction mechanisms	T
BrucellaGL002172	CC0238	Ccr	COG0642	Signal transduction histidine kinase	Signal transduction mechanisms	T
BrucellaGL002173	BMEI2034	Bme	COG1493	Serine kinase of the HPr protein, regulates carbohydrate metabolism	Signal transduction mechanisms	T
BrucellaGL002174	BMEI2032	Bme	COG2893	Phosphotransferase system, mannose/fructose-specific component IIA	Carbohydrate transport and metabolism	G
BrucellaGL002175	BMEI2031	Bme	COG1925	Phosphotransferase system, HPr-related proteins	Carbohydrate transport and metabolism	G
BrucellaGL002176	BMEI2029	Bme	COG0499	S-adenosylhomocysteine hydrolase	Coenzyme transport and metabolism	H
BrucellaGL002179	BMEI2027_1	Bme	COG2202	FOG: PAS/PAC domain	Signal transduction mechanisms	T
BrucellaGL002180	BMEI2026_2	Bme	COG3178	Predicted phosphotransferase related to Ser/Thr protein kinases	General function prediction only	R
BrucellaGL002181	BMEI2025	Bme	COG1208	Nucleoside-diphosphate-sugar pyrophosphorylase involved in lipopolysaccharide biosynthesis/translation initiation factor 2B, gamma/epsilon subunits (eIF-2Bgamma/eIF-2Bepsilon)	Cell wall/membrane/envelope biogenesis	M
BrucellaGL002182	BMEI2024_1	Bme	COG3893	Inactivated superfamily I helicase	Replication, recombination and repair	L
BrucellaGL002183	BMEI2023	Bme	COG1074	ATP-dependent exoDNAse (exonuclease V) beta subunit (contains helicase and exonuclease domains)	Replication, recombination and repair	L
BrucellaGL002184	BMEI2022	Bme	COG0526	Thiol-disulfide isomerase and thioredoxins	Posttranslational modification, protein turnover, chaperones	O

BrucellaGL002185	BMEI2021	Bme	COG0285	Folylpolyglutamate synthase	Coenzyme transport and metabolism	H
BrucellaGL002186	BMEI2020	Bme	COG0777	Acetyl-CoA carboxylase beta subunit	Lipid transport and metabolism	I
BrucellaGL002187	BMEI2019	Bme	COG0159	Tryptophan synthase alpha chain	Amino acid transport and metabolism	E
BrucellaGL002189	BMEI2018	Bme	COG0133	Tryptophan synthase beta chain	Amino acid transport and metabolism	E
BrucellaGL002190	BMEI2017	Bme	COG0135	Phosphoribosylanthranilate isomerase	Amino acid transport and metabolism	E
BrucellaGL002191	BMEI2016	Bme	COG1434	Uncharacterized conserved protein	Function unknown	S
BrucellaGL002192	BMEI2015	Bme	COG1451	Predicted metal-dependent hydrolase	General function prediction only	R
BrucellaGL002195	BMEI2012	Bme	COG3135	Uncharacterized protein involved in benzoate metabolism	Secondary metabolites biosynthesis, transport and catabolism	Q
BrucellaGL002196	BMEI2011	Bme	COG0596	Predicted hydrolases or acyltransferases (alpha/beta hydrolase superfamily)	General function prediction only	R
BrucellaGL002197	BMEI2010	Bme	COG0290	Translation initiation factor 3 (IF-3)	Translation, ribosomal structure and biogenesis	J
BrucellaGL002198	BMEI2009	Bme	COG2020	Putative protein-S-isoprenylcysteine methyltransferase	Posttranslational modification, protein turnover, chaperones	O
BrucellaGL002199	BMEI2008	Bme	COG0291	Ribosomal protein L35	Translation, ribosomal structure and biogenesis	J
BrucellaGL002200	BMEI2007	Bme	COG0292	Ribosomal protein L20	Translation, ribosomal structure and biogenesis	J
BrucellaGL002202	BMEI2005	Bme	COG0016	Phenylalanyl-tRNA synthetase alpha subunit	Translation, ribosomal structure and biogenesis	J
BrucellaGL002203	BMEI2004_2	Bme	COG0072	Phenylalanyl-tRNA synthetase beta subunit	Translation, ribosomal structure and biogenesis	J
BrucellaGL002204	BMEI2003	Bme	COG0673	Predicted dehydrogenases and related proteins	General function prediction only	R
BrucellaGL002205	BMEI2002	Bme	COG0443	Molecular chaperone	Posttranslational modification, protein turnover, chaperones	O
BrucellaGL002207	BMEI2001	Bme	COG0484	DnaJ-class molecular chaperone with C-terminal Zn finger domain	Posttranslational modification, protein turnover, chaperones	O
BrucellaGL002208	BMEI2000	Bme	COG3963	Phospholipid N-methyltransferase	Lipid transport and metabolism	I
BrucellaGL002209	BMEI1999	Bme	COG0284	Orotidine-5'-phosphate decarboxylase	Nucleotide transport and metabolism	F
BrucellaGL002210	BMEI1998	Bme	COG5470	Uncharacterized conserved protein	Function unknown	S
BrucellaGL002211	BMEI1997	Bme	COG3386	Gluconolactonase	Carbohydrate transport and metabolism	G

BrucellaGL002212	BMEI1996	Bme	COG0189	Glutathione synthase/Ribosomal protein S6 modification enzyme (glutaminyl transferase)	Coenzyme transport and metabolism	H
BrucellaGL002213	AGc541	Atu	COG0606	Predicted ATPase with chaperone activity	Posttranslational modification, protein turnover, chaperones	O
BrucellaGL002214	BMEI1993	Bme	COG3672	Predicted periplasmic protein	Function unknown	S
BrucellaGL002215	BMEI1992	Bme	COG0797	Lipoproteins	Cell wall/membrane/envelope biogenesis	M
BrucellaGL002216	BMEI1990	Bme	COG2832	Uncharacterized protein conserved in bacteria	Function unknown	S
BrucellaGL002217	BMEI1989	Bme	COG0226	ABC-type phosphate transport system, periplasmic component	Inorganic ion transport and metabolism	P
BrucellaGL002218	BMEI1988_2	Bme	COG0573	ABC-type phosphate transport system, permease component	Inorganic ion transport and metabolism	P
BrucellaGL002219	BMEI1987	Bme	COG0581	ABC-type phosphate transport system, permease component	Inorganic ion transport and metabolism	P
BrucellaGL002220	BMEI1986	Bme	COG1117	ABC-type phosphate transport system, ATPase component	Inorganic ion transport and metabolism	P
BrucellaGL002221	BMEI1985	Bme	COG0704	Phosphate uptake regulator	Inorganic ion transport and metabolism	P
BrucellaGL002222	BMEI1984	Bme	COG0745	Response regulators consisting of a CheY-like receiver domain and a winged-helix DNA-binding domain	Signal transduction mechanisms	T
BrucellaGL002223	BMEI1983	Bme	COG3773	Cell wall hydrolyses involved in spore germination	Cell wall/membrane/envelope biogenesis	M
BrucellaGL002225	BMEI1981	Bme	COG2309	Leucyl aminopeptidase (aminopeptidase T)	Amino acid transport and metabolism	E
BrucellaGL002226	BMEI1980	Bme	COG0783	DNA-binding ferritin-like protein (oxidative damage protectant)	Inorganic ion transport and metabolism	P
BrucellaGL002228	BMEI1979	Bme	COG1214	Inactive homolog of metal-dependent proteases, putative molecular chaperone	Posttranslational modification, protein turnover, chaperones	O
BrucellaGL002229	BMEI1978	Bme	COG0456	Acetyltransferases	General function prediction only	R
BrucellaGL002230	BMEI1977	Bme	COG0204	1-acyl-sn-glycerol-3-phosphate acyltransferase	Lipid transport and metabolism	I
BrucellaGL002231	BMEI1976	Bme	COG0621	2-methylthioadenine synthetase	Translation, ribosomal structure and biogenesis	J

BrucellaGL002232	BMEI1975	Bme	COG1702	Phosphate starvation-inducible protein PhoH, predicted ATPase	Signal transduction mechanisms	T
BrucellaGL002234	BMEI1974	Bme	COG0319	Predicted metal-dependent hydrolase	General function prediction only	R
BrucellaGL002235	BMEI1973	Bme	COG1253	Hemolysins and related proteins containing CBS domains	General function prediction only	R
BrucellaGL002236	BMEI1972	Bme	COG0815	Apolipoprotein N-acyltransferase	Cell wall/membrane/envelope biogenesis	M
BrucellaGL002237	BMEI1971	Bme	COG1396	Predicted transcriptional regulators	Transcription	K
BrucellaGL002238	BMEI1970	Bme	COG0192	S-adenosylmethionine synthetase	Coenzyme transport and metabolism	H
BrucellaGL002239	BMEI1969	Bme	COG0220	Predicted S-adenosylmethionine-dependent methyltransferase	General function prediction only	R
BrucellaGL002240	BMEI1968	Bme	COG0779	Uncharacterized protein conserved in bacteria	Function unknown	S
BrucellaGL002241	BMEI1967	Bme	COG0195	Transcription elongation factor	Transcription	K
BrucellaGL002242	BMEI1966	Bme	COG2740	Predicted nucleic-acid-binding protein implicated in transcription termination	Transcription	K
BrucellaGL002243	BMEI1965	Bme	COG0532	Translation initiation factor 2 (IF-2; GTPase)	Translation, ribosomal structure and biogenesis	J
BrucellaGL002244	BMEI1964	Bme	COG0858	Ribosome-binding factor A	Translation, ribosomal structure and biogenesis	J
BrucellaGL002245	BMEI1963	Bme	COG0130	Pseudouridine synthase	Translation, ribosomal structure and biogenesis	J
BrucellaGL002246	BMEI1962	Bme	COG0184	Ribosomal protein S15P/S13E	Translation, ribosomal structure and biogenesis	J
BrucellaGL002247	BMEI1961	Bme	COG1185	Polyribonucleotide nucleotidyltransferase (polynucleotide phosphorylase)	Translation, ribosomal structure and biogenesis	J
BrucellaGL002248	BMEI1960	Bme	COG2813	16S RNA G1207 methylase RsmC	Translation, ribosomal structure and biogenesis	J
BrucellaGL002249	BMEI1959	Bme	COG2813	16S RNA G1207 methylase RsmC	Translation, ribosomal structure and biogenesis	J
BrucellaGL002250	BMEI1958	Bme	COG0623	Enoyl-[acyl-carrier-protein] reductase (NADH)	Lipid transport and metabolism	I
BrucellaGL002251	BMEI1957	Bme	COG0304	3-oxoacyl-(acyl-carrier-protein) synthase	Lipid transport and metabolism	I
BrucellaGL002252	BMEI1956	Bme	COG0764	3-hydroxymyristoyl/3-hydroxydecanoyl-(acyl carrier protein) dehydratases	Lipid transport and metabolism	I

BrucellaGL002253	BMEI1955	Bme	COG0735	Fe2+/Zn2+ uptake regulation proteins	Inorganic ion transport and metabolism	P
BrucellaGL002254	BMEI1954	Bme	COG1464	ABC-type metal ion transport system, periplasmic component/surface antigen	Inorganic ion transport and metabolism	P
BrucellaGL002255	BMEI1953	Bme	COG3807	Uncharacterized protein conserved in bacteria	Function unknown	S
BrucellaGL002256	BMEI1952	Bme	COG1052	Lactate dehydrogenase and related dehydrogenases	Energy production and conversion	C
BrucellaGL002257	BMEI1951	Bme	COG0596	Predicted hydrolases or acyltransferases (alpha/beta hydrolase superfamily)	General function prediction only	R
BrucellaGL002258	BMEI1950	Bme	COG0791	Cell wall-associated hydrolases (invasion-associated proteins)	Cell wall/membrane/envelope biogenesis	M
BrucellaGL002259	BMEI1949	Bme	COG1846	Transcriptional regulators	Transcription	K
BrucellaGL002260	BMEI1948	Bme	COG0260	Leucyl aminopeptidase	Amino acid transport and metabolism	E
BrucellaGL002262	BMEI1946	Bme	COG0266	Formamidopyrimidine-DNA glycosylase	Replication, recombination and repair	L
BrucellaGL002263	BMEI1945	Bme	COG1024	Enoyl-CoA hydratase/carnithine racemase	Lipid transport and metabolism	I
BrucellaGL002264	BMEI1944	Bme	COG0268	Ribosomal protein S20	Translation, ribosomal structure and biogenesis	J
BrucellaGL002265	BMEI1943	Bme	COG0593	ATPase involved in DNA replication initiation	Replication, recombination and repair	L
BrucellaGL002266	BMEI1942	Bme	COG0592	DNA polymerase sliding clamp subunit (PCNA homolog)	Replication, recombination and repair	L
BrucellaGL002267	BMEI1941	Bme	COG1195	Recombinational DNA repair ATPase (RecF pathway)	Replication, recombination and repair	L
BrucellaGL002268	BMEI1940	Bme	COG0476	Dinucleotide-utilizing enzymes involved in molybdopterin and thiamine biosynthesis family 2	Coenzyme transport and metabolism	H
BrucellaGL002269	BMEI1939	Bme	COG0111	Phosphoglycerate dehydrogenase and related dehydrogenases	Coenzyme transport and metabolism	H
BrucellaGL002270	BMEI1938	Bme	COG4172	ABC-type uncharacterized transport system, duplicated ATPase component	General function prediction only	R
BrucellaGL002271	BMEI1937	Bme	COG4239	ABC-type uncharacterized transport system, permease component	General function prediction only	R
BrucellaGL002272	BMEI1936	Bme	COG4174	ABC-type uncharacterized transport system, permease component	General function prediction only	R

BrucellaGL002273	BMEI1935	Bme	COG4166	ABC-type oligopeptide transport system, periplasmic component	Amino acid transport and metabolism	E
BrucellaGL002274	BMEI1934	Bme	COG4166	ABC-type oligopeptide transport system, periplasmic component	Amino acid transport and metabolism	E
BrucellaGL002276	BMEI1930	Bme	COG0683	ABC-type branched-chain amino acid transport systems, periplasmic component	Amino acid transport and metabolism	E
BrucellaGL002277	BMEI1928	Bme	COG1024	Enoyl-CoA hydratase/carnithine racemase	Lipid transport and metabolism	I
BrucellaGL002278	BMEI1927	Bme	COG1024	Enoyl-CoA hydratase/carnithine racemase	Lipid transport and metabolism	I
BrucellaGL002279	BMEI1926	Bme	COG0119	Isopropylmalate/homocitrate/citramalate synthases	Amino acid transport and metabolism	E
BrucellaGL002280	BMEI1925	Bme	COG4770	Acetyl/propionyl-CoA carboxylase, alpha subunit	Lipid transport and metabolism	I
BrucellaGL002281	BMEI1924	Bme	COG4799	Acetyl-CoA carboxylase, carboxyltransferase component (subunits alpha and beta)	Lipid transport and metabolism	I
BrucellaGL002282	BMEI1924	Bme	COG4799	Acetyl-CoA carboxylase, carboxyltransferase component (subunits alpha and beta)	Lipid transport and metabolism	I
BrucellaGL002283	BMEI1923	Bme	COG1960	Acyl-CoA dehydrogenases	Lipid transport and metabolism	I
BrucellaGL002285	BMEI1922	Bme	COG0365	Acyl-coenzyme A synthetases/AMP-(fatty) acid ligases	Lipid transport and metabolism	I
BrucellaGL002286	BMEI1921	Bme	COG0365	Acyl-coenzyme A synthetases/AMP-(fatty) acid ligases	Lipid transport and metabolism	I
BrucellaGL002288	BMEI1919	Bme	COG1835	Predicted acyltransferases	Lipid transport and metabolism	I
BrucellaGL002289	BMEI1918	Bme	COG4530	Uncharacterized protein conserved in bacteria	Function unknown	S
BrucellaGL002290	BMEI1917	Bme	COG0128	5-enolpyruvylshikimate-3-phosphate synthase	Amino acid transport and metabolism	E
BrucellaGL002291	BMEI1916	Bme	COG0283	Cytidylate kinase	Nucleotide transport and metabolism	F
BrucellaGL002292	BMEI1915	Bme	COG0539	Ribosomal protein S1	Translation, ribosomal structure and biogenesis	J
BrucellaGL002294	BMEI1914	Bme	COG2855	Predicted membrane protein	Function unknown	S
BrucellaGL002295	AGc1204	Atu	COG0583	Transcriptional regulator	Transcription	K
BrucellaGL002296	BMEI1911	Bme	COG2951	Membrane-bound lytic murein transglycosylase B	Cell wall/membrane/envelope biogenesis	M
BrucellaGL002297	BMEI1910	Bme	COG0353	Recombinational DNA repair protein (RecF pathway)	Replication, recombination and repair	L
BrucellaGL002298	BMEI1909	Bme	COG0718	Uncharacterized protein conserved in bacteria	Function unknown	S

BrucellaGL002299	BMEI1908	Bme	COG2812	DNA polymerase III, gamma/tau subunits	Replication, recombination and repair	L
BrucellaGL002300	BMEI1907	Bme	COG0537	Diadenosine tetraphosphate (Ap4A) hydrolase and other HIT family hydrolases	Nucleotide transport and metabolism	F
BrucellaGL002301	BMEI1906	Bme	COG2816	NTP pyrophosphohydrolases containing a Zn-finger, probably nucleic-acid-binding	Replication, recombination and repair	L
BrucellaGL002302	BMEI1905	Bme	COG0077	Prephenate dehydratase	Amino acid transport and metabolism	E
BrucellaGL002304	BMEI1904	Bme	COG1212	CMP-2-keto-3-deoxyoctulosonic acid synthetase	Cell wall/membrane/envelope biogenesis	M
BrucellaGL002305	BMEI1903	Bme	COG3474	Cytochrome c2	Energy production and conversion	C
BrucellaGL002306	BMEI1902	Bme	COG0303	Molybdopterin biosynthesis enzyme	Coenzyme transport and metabolism	H
BrucellaGL002308	BMEI1901	Bme	COG1622	Heme/copper-type cytochrome/quinol oxidases, subunit 2	Energy production and conversion	C
BrucellaGL002309	BMEI1900	Bme	COG0843	Heme/copper-type cytochrome/quinol oxidases, subunit 1	Energy production and conversion	C
BrucellaGL002310	BMEI1899	Bme	COG1845	Heme/copper-type cytochrome/quinol oxidase, subunit 3	Energy production and conversion	C
BrucellaGL002311	BMEI1898	Bme	COG3125	Heme/copper-type cytochrome/quinol oxidase, subunit 4	Energy production and conversion	C
BrucellaGL002312	BMEI0502	Bme	COG4991	Uncharacterized protein with a bacterial SH3 domain homologue	Function unknown	S
BrucellaGL002314	BMEI1895	Bme	COG0729	Outer membrane protein	Cell wall/membrane/envelope biogenesis	M
BrucellaGL002315	BMEI1894	Bme	COG2911	Uncharacterized protein conserved in bacteria	Function unknown	S
BrucellaGL002316	BMEI1893	Bme	COG1376	Uncharacterized protein conserved in bacteria	Function unknown	S
BrucellaGL002317	BMEI1893	Bme	COG1376	Uncharacterized protein conserved in bacteria	Function unknown	S
BrucellaGL002321	BMEI1890_1	Bme	COG4549	Uncharacterized protein conserved in bacteria	Function unknown	S
BrucellaGL002323	BMEI1888	Bme	COG0346	Lactoylglutathione lyase and related lyases	Amino acid transport and metabolism	E
BrucellaGL002324	BMEI1887	Bme	COG0489	ATPases involved in chromosome partitioning	Cell cycle control, cell division, chromosome partitioning	D
BrucellaGL002326	BMEI1886	Bme	COG0033	Phosphoglucomutase	Carbohydrate transport and metabolism	G
BrucellaGL002327	RSc0205	Rso	COG0583	Transcriptional regulator	Transcription	K
BrucellaGL002328	BMEI1884	Bme	COG1073	Hydrolases of the alpha/beta superfamily	General function prediction only	R

BrucellaGL002329	BMEI1883	Bme	COG1072	Panthothenate kinase	Coenzyme transport and metabolism	H
BrucellaGL002330	BMEI1882	Bme	COG0179	2-keto-4-pentenoate hydratase/2-oxohepta-3-ene-1,7-dioic acid hydratase (catechol pathway)	Secondary metabolites biosynthesis, transport and catabolism	Q
BrucellaGL002334	BMEI1878	Bme	COG0741	Soluble lytic murein transglycosylase and related regulatory proteins (some contain LysM/invasin domains)	Cell wall/membrane/envelope biogenesis	M
BrucellaGL002336	BMEI1877	Bme	COG1272	Predicted membrane protein, hemolysin III homolog	General function prediction only	R
BrucellaGL002337	BMEI1876	Bme	COG0587	DNA polymerase III, alpha subunit	Replication, recombination and repair	L
BrucellaGL002338	BMEI1875	Bme	COG0389	Nucleotidyltransferase/DNA polymerase involved in DNA repair	Replication, recombination and repair	L
BrucellaGL002339	BMEI1874	Bme	COG4544	Uncharacterized conserved protein	Function unknown	S
BrucellaGL002340	BMEI1873	Bme	COG5295	Autotransporter adhesin	Intracellular trafficking, secretion, and vesicular transport	U
BrucellaGL002341	BMEI1872	Bme	COG5295	Autotransporter adhesin	Intracellular trafficking, secretion, and vesicular transport	U
BrucellaGL002343	BMEI1870	Bme	COG0137	Argininosuccinate synthase	Amino acid transport and metabolism	E
BrucellaGL002344	BMEI1869	Bme	COG1280	Putative threonine efflux protein	Amino acid transport and metabolism	E
BrucellaGL002346	BMEI1867	Bme	COG0820	Predicted Fe-S-cluster redox enzyme	General function prediction only	R
BrucellaGL002348	BMEI1865	Bme	COG3339	Uncharacterized conserved protein	Function unknown	S
BrucellaGL002349	BMEI1864	Bme	COG2154	Pterin-4a-carbinolamine dehydratase	Coenzyme transport and metabolism	H
BrucellaGL002350	BMEI1862	Bme	COG1514	2'-5' RNA ligase	Translation, ribosomal structure and biogenesis	J
BrucellaGL002351	BMEI1861	Bme	COG2755	Lysophospholipase L1 and related esterases	Amino acid transport and metabolism	E
BrucellaGL002355	BMEI1860	Bme	COG3127	Predicted ABC-type transport system involved in lysophospholipase L1 biosynthesis, permease component	Secondary metabolites biosynthesis, transport and catabolism	Q
BrucellaGL002356	BMEI1859	Bme	COG0670	Integral membrane protein, interacts with FtsH	General function prediction only	R
BrucellaGL002357	BMEI1858	Bme	COG1247	Sortase and related acyltransferases	Cell wall/membrane/envelope biogenesis	M
BrucellaGL002360	BMEI1856	Bme	COG5429	Uncharacterized secreted protein	Function unknown	S
BrucellaGL002362	BMEI1855	Bme	COG1048	Aconitase A	Energy production and conversion	C

BrucellaGL002364	BMEI1853	Bme	COG4133	ABC-type transport system involved in cytochrome c biogenesis, ATPase component	Posttranslational modification, protein turnover, chaperones	O
BrucellaGL002365	BMEI1852	Bme	COG2386	ABC-type transport system involved in cytochrome c biogenesis, permease component	Posttranslational modification, protein turnover, chaperones	O
BrucellaGL002366	BMEI1851	Bme	COG0755	ABC-type transport system involved in cytochrome c biogenesis, permease component	Posttranslational modification, protein turnover, chaperones	O
BrucellaGL002368	BMEI1849	Bme	COG0526	Thiol-disulfide isomerase and thioredoxins	Posttranslational modification, protein turnover, chaperones	O
BrucellaGL002369	BMEI1848	Bme	COG0129	Dihydroxyacid dehydratase/phosphogluconate dehydratase	Amino acid transport and metabolism	E
BrucellaGL002371	BMEI1846	Bme	COG0784	FOG: CheY-like receiver	Signal transduction mechanisms	T
BrucellaGL002373	BMEI1845	Bme	COG1522	Transcriptional regulators	Transcription	K
BrucellaGL002379	BMEI1842	Bme	COG1434	Uncharacterized conserved protein	Function unknown	S
BrucellaGL002380	BMEI1841	Bme	COG1613	ABC-type sulfate transport system, periplasmic component	Inorganic ion transport and metabolism	P
BrucellaGL002381	BMEI1840	Bme	COG0555	ABC-type sulfate transport system, permease component	Posttranslational modification, protein turnover, chaperones	O
BrucellaGL002382	BMEI1839	Bme	COG4208	ABC-type sulfate transport system, permease component	Inorganic ion transport and metabolism	P
BrucellaGL002383	BMEI1838	Bme	COG1118	ABC-type sulfate/molybdate transport systems, ATPase component	Inorganic ion transport and metabolism	P
BrucellaGL002384	BMEI1837	Bme	COG3459	Cellobiose phosphorylase	Carbohydrate transport and metabolism	G
BrucellaGL002385	BMEI1836	Bme	COG1488	Nicotinic acid phosphoribosyltransferase	Coenzyme transport and metabolism	H
BrucellaGL002386	BMEI1835	Bme	COG0384	Predicted epimerase, PhzC/PhzF homolog	General function prediction only	R
BrucellaGL002387	BMEI1834	Bme	COG0500	SAM-dependent methyltransferases	Secondary metabolites biosynthesis, transport and catabolism	Q
BrucellaGL002388	BMEI1833	Bme	COG0524	Sugar kinases, ribokinase family	Carbohydrate transport and metabolism	G
BrucellaGL002389	aq_1716	Aae	COG1028	Dehydrogenases with different specificities (related to short-chain alcohol dehydrogenases)	Lipid transport and metabolism	I
BrucellaGL002390	BMEI1831	Bme	COG0744	Membrane carboxypeptidase (penicillin-binding protein)	Cell wall/membrane/envelope biogenesis	M

BrucellaGL002391	BMEI1830	Bme	COG3637	Opacity protein and related surface antigens	Cell wall/membrane/envelope biogenesis	M
BrucellaGL002392	BMEI1829	Bme	COG3637	Opacity protein and related surface antigens	Cell wall/membrane/envelope biogenesis	M
BrucellaGL002393	BMEI1828	Bme	COG0349	Ribonuclease D	Translation, ribosomal structure and biogenesis	J
BrucellaGL002394	BMEI1827	Bme	COG0624	Acetylornithine deacetylase/Succinyl-diaminopimelate desuccinylase and related deacylases	Amino acid transport and metabolism	E
BrucellaGL002395	BMEI1825_2	Bme	COG0749	DNA polymerase I - 3'-5' exonuclease and polymerase domains	Replication, recombination and repair	L
BrucellaGL002396	BMEI1824	Bme	COG0513	Superfamily II DNA and RNA helicases	Replication, recombination and repair	L
BrucellaGL002397	BMEI1823	Bme	COG0187	Type IIA topoisomerase (DNA gyrase/topo II, topoisomerase IV), B subunit	Replication, recombination and repair	L
BrucellaGL002399	BMEI1822	Bme	COG0627	Predicted esterase	General function prediction only	R
BrucellaGL002400	BMEI1822	Bme	COG0627	Predicted esterase	General function prediction only	R
BrucellaGL002401	BMEI1821	Bme	COG4291	Predicted membrane protein	Function unknown	S
BrucellaGL002402	BMEI1821	Bme	COG4291	Predicted membrane protein	Function unknown	S
BrucellaGL002403	BMEI1820	Bme	COG2153	Predicted acyltransferase	General function prediction only	R
BrucellaGL002404	BMEI1819	Bme	COG1062	Zn-dependent alcohol dehydrogenases, class III	Energy production and conversion	C
BrucellaGL002405	yaiN	Eco	COG1937	Uncharacterized protein conserved in bacteria	Function unknown	S
BrucellaGL002406	BMEI1818	Bme	COG1643	HrpA-like helicases	Replication, recombination and repair	L
BrucellaGL002407	BMEI1818	Bme	COG1643	HrpA-like helicases	Replication, recombination and repair	L
BrucellaGL002408	BMEI1817	Bme	COG1643	HrpA-like helicases	Replication, recombination and repair	L
BrucellaGL002409	CC0248	Ccr	COG0642	Signal transduction histidine kinase	Signal transduction mechanisms	T
BrucellaGL002412	BMEI1811	Bme	COG4567	Response regulator consisting of a CheY-like receiver domain and a Fis-type HTH domain	Signal transduction mechanisms	T
BrucellaGL002413	BMEI1810	Bme	COG5321	Uncharacterized protein conserved in bacteria	Function unknown	S
BrucellaGL002414	BMEI1809	Bme	COG1376	Uncharacterized protein conserved in bacteria	Function unknown	S
BrucellaGL002415	BMEI1808	Bme	COG0694	Thioredoxin-like proteins and domains	Posttranslational modification, protein turnover, chaperones	O

BrucellaGL002416	BMEI1806	Bme	COG0180	Tryptophanyl-tRNA synthetase	Translation, ribosomal structure and biogenesis	J
BrucellaGL002417	BMEI1805	Bme	COG0728	Uncharacterized membrane protein, putative virulence factor	General function prediction only	R
BrucellaGL002418	BMEI1804	Bme	COG2844	UTP:GlnB (protein PII) uridylyltransferase	Posttranslational modification, protein turnover, chaperones	O
BrucellaGL002419	BMEI1803	Bme	COG0280	Phosphotransacetylase	Energy production and conversion	C
BrucellaGL002420	BMEI1802	Bme	COG0281	Malic enzyme	Energy production and conversion	C
BrucellaGL002421	BMEI1801	Bme	COG0249	Mismatch repair ATPase (MutS family)	Replication, recombination and repair	L
BrucellaGL002422	BMEI1800	Bme	COG3176	Putative hemolysin	General function prediction only	R
BrucellaGL002423	BMEI1799	Bme	COG0597	Lipoprotein signal peptidase	Cell wall/membrane/envelope biogenesis	M
BrucellaGL002424	BMEI1798	Bme	COG0566	rRNA methylases	Translation, ribosomal structure and biogenesis	J
BrucellaGL002425	BMEI1797	Bme	COG1092	Predicted SAM-dependent methyltransferases	General function prediction only	R
BrucellaGL002426	mlr3209	Mlo	COG1092	Predicted SAM-dependent methyltransferases	General function prediction only	R
BrucellaGL002428	BMEI1794	Bme	COG0776	Bacterial nucleoid DNA-binding protein	Replication, recombination and repair	L
BrucellaGL002429	BMEI1793	Bme	COG0616	Periplasmic serine proteases (ClpP class)	Posttranslational modification, protein turnover, chaperones	O
BrucellaGL002430	BMEI1792	Bme	COG5375	Uncharacterized protein conserved in bacteria	Function unknown	S
BrucellaGL002431	BMEI1791	Bme	COG1934	Uncharacterized protein conserved in bacteria	Function unknown	S
BrucellaGL002432	BMEI1790	Bme	COG1137	ABC-type (unclassified) transport system, ATPase component	General function prediction only	R
BrucellaGL002433	BMEI1789	Bme	COG1508	DNA-directed RNA polymerase specialized sigma subunit, sigma54 homolog	Transcription	K
BrucellaGL002434	BMEI1787	Bme	COG1544	Ribosome-associated protein Y (PSrp-1)	Translation, ribosomal structure and biogenesis	J
BrucellaGL002436	BMEI1786	Bme	COG1762	Phosphotransferase system mannitol/fructose-specific IIA domain (Ntr-type)	Carbohydrate transport and metabolism	G
BrucellaGL002437	BMEI1785	Bme	COG5568	Uncharacterized small protein	Function unknown	S
BrucellaGL002438	BMEI1784	Bme	COG0071	Molecular chaperone (small heat shock protein)	Posttranslational modification, protein turnover, chaperones	O
BrucellaGL002439	BMEI1783	Bme	COG5488	Integral membrane protein	Function unknown	S

BrucellaGL002440	BMEI1782	Bme	COG0177	Predicted EndoIII-related endonuclease	Replication, recombination and repair	L
BrucellaGL002441	BMEI1781	Bme	COG2981	Uncharacterized protein involved in cysteine biosynthesis	Amino acid transport and metabolism	E
BrucellaGL002443	BMEI1779	Bme	COG0524	Sugar kinases, ribokinase family	Carbohydrate transport and metabolism	G
BrucellaGL002444	BMEI1778	Bme	COG2860	Predicted membrane protein	Function unknown	S
BrucellaGL002445	BMEI1777	Bme	COG0576	Molecular chaperone GrpE (heat shock protein)	Posttranslational modification, protein turnover, chaperones	O
BrucellaGL002446	BMEI1776	Bme	COG1420	Transcriptional regulator of heat shock gene	Transcription	K
BrucellaGL002447	BMEI1775	Bme	COG0689	RNase PH	Translation, ribosomal structure and biogenesis	J
BrucellaGL002449	BMEI1774	Bme	COG0346	Lactoylglutathione lyase and related lyases	Amino acid transport and metabolism	E
BrucellaGL002450	BMEI1773	Bme	COG0346	Lactoylglutathione lyase and related lyases	Amino acid transport and metabolism	E
BrucellaGL002451	BMEI1772	Bme	COG0127	Xanthosine triphosphate pyrophosphatase	Nucleotide transport and metabolism	F
BrucellaGL002452	BMEI1771	Bme	COG0635	Coproporphyrinogen III oxidase and related Fe-S oxidoreductases	Coenzyme transport and metabolism	H
BrucellaGL002453	BMEI1770	Bme	COG0313	Predicted methyltransferases	General function prediction only	R
BrucellaGL002454	BMEI1769	Bme	COG0792	Predicted endonuclease distantly related to archaeal Holliday junction resolvase	Replication, recombination and repair	L
BrucellaGL002456	BMEI1768_2	Bme	COG0007	Uroporphyrinogen-III methylase	Coenzyme transport and metabolism	H
BrucellaGL002458	BMEI1766	Bme	COG0155	Sulfite reductase, beta subunit (hemoprotein)	Inorganic ion transport and metabolism	P
BrucellaGL002459	BMEI1765	Bme	COG0175	3'-phosphoadenosine 5'-phosphosulfate sulfotransferase (PAPS reductase)/FAD synthetase and related enzymes	Amino acid transport and metabolism	E
BrucellaGL002460	BMEI1764	Bme	COG3749	Uncharacterized protein conserved in bacteria	Function unknown	S
BrucellaGL002462	BMEI1762	Bme	COG0350	Methylated DNA-protein cysteine methyltransferase	Replication, recombination and repair	L
BrucellaGL002465	BMEI1759_2	Bme	COG1410	Methionine synthase I, cobalamin-binding domain	Amino acid transport and metabolism	E
BrucellaGL002466	BMEI1758	Bme	COG2771	DNA-binding HTH domain-containing proteins	Transcription	K
BrucellaGL002467	BMEI1757	Bme	COG0161	Adenosylmethionine-8-amino-7-oxononanoate aminotransferase	Coenzyme transport and metabolism	H

BrucellaGL002468	BMEI1755	Bme	COG0175	3'-phosphoadenosine 5'-phosphosulfate sulfotransferase (PAPS reductase)/FAD synthetase and related enzymes	Amino acid transport and metabolism	E
BrucellaGL002469	BMEI1754_1	Bme	COG2895	GTPases - Sulfate adenylate transferase subunit 1	Inorganic ion transport and metabolism	P
BrucellaGL002470	BMEI1753	Bme	COG1218	3'-Phosphoadenosine 5'-phosphosulfate (PAPS) 3'-phosphatase	Inorganic ion transport and metabolism	P
BrucellaGL002472	BMEI1752	Bme	COG0664	cAMP-binding proteins - catabolite gene activator and regulatory subunit of cAMP-dependent protein kinases	Signal transduction mechanisms	T
BrucellaGL002473	BMEI1751	Bme	COG2197	Response regulator containing a CheY-like receiver domain and an HTH DNA-binding domain	Signal transduction mechanisms	T
BrucellaGL002474	BMEI1750	Bme	COG1349	Transcriptional regulators of sugar metabolism	Transcription	K
BrucellaGL002475	BMEI1749	Bme	COG0578	Glycerol-3-phosphate dehydrogenase	Energy production and conversion	C
BrucellaGL002476	BMEI1748	Bme	COG3284	Transcriptional activator of acetoin/glycerol metabolism	Secondary metabolites biosynthesis, transport and catabolism	Q
BrucellaGL002477	BH0681	Bha	COG1012	NAD-dependent aldehyde dehydrogenases	Energy production and conversion	C
BrucellaGL002478	BMEI1746	Bme	COG1064	Zn-dependent alcohol dehydrogenases	General function prediction only	R
BrucellaGL002479	BMEI1745	Bme	COG3564	Uncharacterized protein conserved in bacteria	Function unknown	S
BrucellaGL002480	BMEI1744	Bme	COG1609	Transcriptional regulators	Transcription	K
BrucellaGL002481	BMEI1743	Bme	COG1132	ABC-type multidrug transport system, ATPase and permease components	Defense mechanisms	V
BrucellaGL002483	BMEI1742	Bme	COG1132	ABC-type multidrug transport system, ATPase and permease components	Defense mechanisms	V
BrucellaGL002484	BMEI1740	Bme	COG1012	NAD-dependent aldehyde dehydrogenases	Energy production and conversion	C
BrucellaGL002486	BMEI1739	Bme	COG0600	ABC-type nitrate/sulfonate/bicarbonate transport system, permease component	Inorganic ion transport and metabolism	P
BrucellaGL002487	BMEI1738	Bme	COG0819	Putative transcription activator	Transcription	K
BrucellaGL002488	BMEI1737	Bme	COG0715	ABC-type nitrate/sulfonate/bicarbonate transport systems, periplasmic components	Inorganic ion transport and metabolism	P
BrucellaGL002489	BMEI1736	Bme	COG0352	Thiamine monophosphate synthase	Coenzyme transport and metabolism	H
BrucellaGL002490	BMEI1735	Bme	COG2022	Uncharacterized enzyme of thiazole biosynthesis	Coenzyme transport and metabolism	H

BrucellaGL002491	BMEI1734	Bme	COG2104	Sulfur transfer protein involved in thiamine biosynthesis	Coenzyme transport and metabolism	H
BrucellaGL002492	BMEI1733	Bme	COG0665	Glycine/D-amino acid oxidases (deaminating)	Amino acid transport and metabolism	E
BrucellaGL002493	BMEI1732	Bme	COG0351	Hydroxymethylpyrimidine/phosphomethylpyrimidine kinase	Coenzyme transport and metabolism	H
BrucellaGL002495	BMEI1731_3	Bme	COG2200	FOG: EAL domain	Signal transduction mechanisms	T
BrucellaGL002496	BMEI1730	Bme	COG2217	Cation transport ATPase	Inorganic ion transport and metabolism	P
BrucellaGL002497	SMb21579	Sme	COG0789	Predicted transcriptional regulators	Transcription	K
BrucellaGL002498	BMEI1728	Bme	COG1174	ABC-type proline/glycine betaine transport systems, permease component	Amino acid transport and metabolism	E
BrucellaGL002499	BMEI1727	Bme	COG1125	ABC-type proline/glycine betaine transport systems, ATPase components	Amino acid transport and metabolism	E
BrucellaGL002500	BMEI1726	Bme	COG1174	ABC-type proline/glycine betaine transport systems, permease component	Amino acid transport and metabolism	E
BrucellaGL002501	BMEI1725	Bme	COG1732	Periplasmic glycine betaine/choline-binding (lipo)protein of an ABC-type transport system (osmoprotectant binding protein)	Cell wall/membrane/envelope biogenesis	M
BrucellaGL002502	BMEI1724	Bme	COG5514	Uncharacterized conserved protein	Function unknown	S
BrucellaGL002503	BMEI1723	Bme	COG0334	Glutamate dehydrogenase/leucine dehydrogenase	Amino acid transport and metabolism	E
BrucellaGL002504	BMEI1722	Bme	COG0665	Glycine/D-amino acid oxidases (deaminating)	Amino acid transport and metabolism	E
BrucellaGL002505	BMEI1721	Bme	COG4311	Sarcosine oxidase delta subunit	Amino acid transport and metabolism	E
BrucellaGL002506	BMEI1720_1	Bme	COG0446	Uncharacterized NAD(FAD)-dependent dehydrogenases	General function prediction only	R
BrucellaGL002507	BMEI1719	Bme	COG4583	Sarcosine oxidase gamma subunit	Amino acid transport and metabolism	E
BrucellaGL002508	BMEI1718	Bme	COG3618	Predicted metal-dependent hydrolase of the TIM-barrel fold	General function prediction only	R
BrucellaGL002509	BMEI1717	Bme	COG1414	Transcriptional regulator	Transcription	K
BrucellaGL002510	BMEI1716	Bme	COG1653	ABC-type sugar transport system, periplasmic component	Carbohydrate transport and metabolism	G
BrucellaGL002511	BMEI1715	Bme	COG1175	ABC-type sugar transport systems, permease components	Carbohydrate transport and metabolism	G

BrucellaGL002512	BMEI1714	Bme	COG0395	ABC-type sugar transport system, permease component	Carbohydrate transport and metabolism	G
BrucellaGL002513	BMEI1713	Bme	COG3839	ABC-type sugar transport systems, ATPase components	Carbohydrate transport and metabolism	G
BrucellaGL002514	BMEI1712	Bme	COG4948	L-alanine-DL-glutamate epimerase and related enzymes of enolase superfamily	Cell wall/membrane/envelope biogenesis	M
BrucellaGL002515	BMEI1711	Bme	COG3254	Uncharacterized conserved protein	Function unknown	S
BrucellaGL002516	BMEI1710	Bme	COG0673	Predicted dehydrogenases and related proteins	General function prediction only	R
BrucellaGL002517	YPO1565	Ype	COG1028	Dehydrogenases with different specificities (related to short-chain alcohol dehydrogenases)	Lipid transport and metabolism	I
BrucellaGL002518	BMEI1708	Bme	COG0179	2-keto-4-pentenoate hydratase/2-oxohepta-3-ene-1,7-dioic acid hydratase (catechol pathway)	Secondary metabolites biosynthesis, transport and catabolism	Q
BrucellaGL002519	BMEI1707	Bme	COG4948	L-alanine-DL-glutamate epimerase and related enzymes of enolase superfamily	Cell wall/membrane/envelope biogenesis	M
BrucellaGL002521	BMEI1702	Bme	COG0582	Integrase	Replication, recombination and repair	L
BrucellaGL002522	BMEI1700	Bme	COG3311	Predicted transcriptional regulator	Transcription	K
BrucellaGL002530	BMEI1692_1	Bme	COG1705	Muramidase (flagellum-specific)	Cell motility	N
BrucellaGL002538	BMEI1680	Bme	COG1396	Predicted transcriptional regulators	Transcription	K
BrucellaGL002545	BMEI1673	Bme	COG3024	Uncharacterized protein conserved in bacteria	Function unknown	S
BrucellaGL002546	BMEI1672	Bme	COG0424	Nucleotide-binding protein implicated in inhibition of septum formation	Cell cycle control, cell division, chromosome partitioning	D
BrucellaGL002547	BMEI1671	Bme	COG0361	Translation initiation factor 1 (IF-1)	Translation, ribosomal structure and biogenesis	J
BrucellaGL002548	BMEI1670	Bme	COG0394	Protein-tyrosine-phosphatase	Signal transduction mechanisms	T
BrucellaGL002549	BMEI1669	Bme	COG5328	Uncharacterized protein conserved in bacteria	Function unknown	S
BrucellaGL002550	BMEI1668	Bme	COG0141	Histidinol dehydrogenase	Amino acid transport and metabolism	E
BrucellaGL002552	BMEI1666	Bme	COG0766	UDP-N-acetylglucosamine enolpyruvyl transferase	Cell wall/membrane/envelope biogenesis	M
BrucellaGL002555	BMEI1664	Bme	COG0582	Integrase	Replication, recombination and repair	L
BrucellaGL002557	BMEI1663	Bme	COG3617	Prophage antirepressor	Transcription	K
BrucellaGL002558	BMEI0899	Bme	COG3617	Prophage antirepressor	Transcription	K

BrucellaGL002559	BMEI0898	Bme	COG1804	Predicted acyl-CoA transferases/carnitine dehydratase	Energy production and conversion	C
BrucellaGL002560	BMEI0897	Bme	COG1960	Acyl-CoA dehydrogenases	Lipid transport and metabolism	I
BrucellaGL002561	BMEI0896	Bme	COG0583	Transcriptional regulator	Transcription	K
BrucellaGL002562	BMEI0895	Bme	COG0583	Transcriptional regulator	Transcription	K
BrucellaGL002563	BMEI0894	Bme	COG2141	Coenzyme F420-dependent N5,N10-methylene tetrahydromethanopterin reductase and related flavin-dependent oxidoreductases	Energy production and conversion	C
BrucellaGL002564	BMEI0893	Bme	COG0841	Cation/multidrug efflux pump	Defense mechanisms	V
BrucellaGL002565	BMEI0892	Bme	COG0845	Membrane-fusion protein	Cell wall/membrane/envelope biogenesis	M
BrucellaGL002566	BMEI0891	Bme	COG1309	Transcriptional regulator	Transcription	K
BrucellaGL002567	BMEI0890	Bme	COG0343	Queuine/archaeosine tRNA-ribosyltransferase	Translation, ribosomal structure and biogenesis	J
BrucellaGL002568	BMEI0889	Bme	COG0809	S-adenosylmethionine:tRNA-ribosyltransferase-isomerase (queuine synthetase)	Translation, ribosomal structure and biogenesis	J
BrucellaGL002569	BMEI0888	Bme	COG0652	Peptidyl-prolyl cis-trans isomerase (rotamase) - cyclophilin family	Posttranslational modification, protein turnover, chaperones	O
BrucellaGL002570	BMEI0887	Bme	COG0652	Peptidyl-prolyl cis-trans isomerase (rotamase) - cyclophilin family	Posttranslational modification, protein turnover, chaperones	O
BrucellaGL002571	BMEI0886	Bme	COG0669	Phosphopantetheine adenylyltransferase	Coenzyme transport and metabolism	H
BrucellaGL002574	BMEI0884	Bme	COG0188	Type IIA topoisomerase (DNA gyrase/topo II, topoisomerase IV), A subunit	Replication, recombination and repair	L
BrucellaGL002575	BMEI0883	Bme	COG2095	Multiple antibiotic transporter	Intracellular trafficking, secretion, and vesicular transport	U
BrucellaGL002576	BMEI0882	Bme	COG3709	Uncharacterized component of phosphonate metabolism	Inorganic ion transport and metabolism	P
BrucellaGL002577	BMEI0881	Bme	COG2188	Transcriptional regulators	Transcription	K
BrucellaGL002578	BMEI0880	Bme	COG0629	Single-stranded DNA-binding protein	Replication, recombination and repair	L
BrucellaGL002579	BMEI0878	Bme	COG0178	Excinuclease ATPase subunit	Replication, recombination and repair	L
BrucellaGL002580	BMEI0877	Bme	COG0776	Bacterial nucleoid DNA-binding protein	Replication, recombination and repair	L
BrucellaGL002581	BMEI0876	Bme	COG0466	ATP-dependent Lon protease, bacterial type	Posttranslational modification, protein turnover, chaperones	O

BrucellaGL002582	BMEI0875	Bme	COG1219	ATP-dependent protease Clp, ATPase subunit	Posttranslational modification, protein turnover, chaperones	O
BrucellaGL002583	BMEI0874	Bme	COG0740	Protease subunit of ATP-dependent Clp proteases	Posttranslational modification, protein turnover, chaperones	O
BrucellaGL002584	BMEI0873	Bme	COG2262	GTPases	General function prediction only	R
BrucellaGL002585	BMEI0872	Bme	COG1923	Uncharacterized host factor I protein	General function prediction only	R
BrucellaGL002587	BMEI0869	Bme	COG0569	K+ transport systems, NAD-binding component	Inorganic ion transport and metabolism	P
BrucellaGL002588	BMEI0868	Bme	COG2204	Response regulator containing CheY-like receiver, AAA-type ATPase, and DNA-binding domains	Signal transduction mechanisms	T
BrucellaGL002589	BMEI0867	Bme	COG5000	Signal transduction histidine kinase involved in nitrogen fixation and metabolism regulation	Signal transduction mechanisms	T
BrucellaGL002590	BMEI0866	Bme	COG2204	Response regulator containing CheY-like receiver, AAA-type ATPase, and DNA-binding domains	Signal transduction mechanisms	T
BrucellaGL002591	BMEI0865	Bme	COG3852	Signal transduction histidine kinase, nitrogen specific	Signal transduction mechanisms	T
BrucellaGL002592	BMEI0864	Bme	COG0042	tRNA-dihydrouridine synthase	Translation, ribosomal structure and biogenesis	J
BrucellaGL002593	BMEI0863_1	Bme	COG1211	4-diphosphocytidyl-2-methyl-D-erithritol synthase	Lipid transport and metabolism	I
BrucellaGL002594	BMEI0862	Bme	COG1546	Uncharacterized protein (competence- and mitomycin-induced)	General function prediction only	R
BrucellaGL002595	MA0415	Mac	COG1028	Dehydrogenases with different specificities (related to short-chain alcohol dehydrogenases)	Lipid transport and metabolism	I
BrucellaGL002596	BMEI0859	Bme	COG0320	Lipoate synthase	Coenzyme transport and metabolism	H
BrucellaGL002598	BMEI0857	Bme	COG1249	Pyruvate/2-oxoglutarate dehydrogenase complex, dihydrolipoamide dehydrogenase (E3) component, and related enzymes	Energy production and conversion	C
BrucellaGL002599	BMEI0856	Bme	COG0508	Pyruvate/2-oxoglutarate dehydrogenase complex, dihydrolipoamide acyltransferase (E2) component, and related enzymes	Energy production and conversion	C

BrucellaGL002600	BMEI0855_2	Bme	COG0022	Pyruvate/2-oxoglutarate dehydrogenase complex, dehydrogenase (E1) component, eukaryotic type, beta subunit	Energy production and conversion	C
BrucellaGL002601	BMEI0854	Bme	COG1071	Pyruvate/2-oxoglutarate dehydrogenase complex, dehydrogenase (E1) component, eukaryotic type, alpha subunit	Energy production and conversion	C
BrucellaGL002602	BMEI0853	Bme	COG2919	Septum formation initiator	Cell cycle control, cell division, chromosome partitioning	D
BrucellaGL002603	BMEI0852	Bme	COG0500	SAM-dependent methyltransferases	Secondary metabolites biosynthesis, transport and catabolism	Q
BrucellaGL002604	BMEI0851	Bme	COG0148	Enolase	Carbohydrate transport and metabolism	G
BrucellaGL002605	BMEI0850	Bme	COG2877	3-deoxy-D-manno-octulosonic acid (KDO) 8-phosphate synthase	Cell wall/membrane/envelope biogenesis	M
BrucellaGL002606	BMEI0849	Bme	COG0504	CTP synthase (UTP-ammonia lyase)	Nucleotide transport and metabolism	F
BrucellaGL002607	BMEI0848	Bme	COG1376	Uncharacterized protein conserved in bacteria	Function unknown	S
BrucellaGL002608	BMEI0847	Bme	COG1314	Preprotein translocase subunit SecG	Intracellular trafficking, secretion, and vesicular transport	U
BrucellaGL002609	BMEI0846	Bme	COG0149	Triosephosphate isomerase	Carbohydrate transport and metabolism	G
BrucellaGL002610	BMEI0845	Bme	COG0760	Parvulin-like peptidyl-prolyl isomerase	Posttranslational modification, protein turnover, chaperones	O
BrucellaGL002611	BMEI0844	Bme	COG0547	Anthranilate phosphoribosyltransferase	Amino acid transport and metabolism	E
BrucellaGL002612	BMEI0843	Bme	COG0134	Indole-3-glycerol phosphate synthase	Amino acid transport and metabolism	E
BrucellaGL002613	BMEI0842	Bme	COG0315	Molybdenum cofactor biosynthesis enzyme	Coenzyme transport and metabolism	H
BrucellaGL002614	BMEI0841	Bme	COG0303	Molybdopterin biosynthesis enzyme	Coenzyme transport and metabolism	H
BrucellaGL002615	BMEI0840	Bme	COG1974	SOS-response transcriptional repressors (RecA-mediated autopeptidases)	Transcription	K
BrucellaGL002616	BMEI0839	Bme	COG0658	Predicted membrane metal-binding protein	General function prediction only	R
BrucellaGL002617	mll0627	Mlo	COG0658	Predicted membrane metal-binding protein	General function prediction only	R
BrucellaGL002619	BMEI0837	Bme	COG0008	Glutamyl- and glutaminyl-tRNA synthetases	Translation, ribosomal structure and biogenesis	J
BrucellaGL002620	BMEI0836	Bme	COG0372	Citrate synthase	Energy production and conversion	C

BrucellaGL002621	BMEI0835	Bme	COG0763	Lipid A disaccharide synthetase	Cell wall/membrane/envelope biogenesis	M
BrucellaGL002622	BMEI0834	Bme	COG3494	Uncharacterized protein conserved in bacteria	Function unknown	S
BrucellaGL002623	BMEI0833	Bme	COG1043	Acyl-[acyl carrier protein]--UDP-N-acetylglucosamine O-acyltransferase	Cell wall/membrane/envelope biogenesis	M
BrucellaGL002624	BMEI0832	Bme	COG0764	3-hydroxymyristoyl/3-hydroxydecanoyl-(acyl carrier protein) dehydratases	Lipid transport and metabolism	I
BrucellaGL002625	BMEI0831	Bme	COG1044	UDP-3-O-[3-hydroxymyristoyl] glucosamine N-acyltransferase	Cell wall/membrane/envelope biogenesis	M
BrucellaGL002626	BMEI0830	Bme	COG4775	Outer membrane protein/protective antigen OMA87	Cell wall/membrane/envelope biogenesis	M
BrucellaGL002627	BMEI0829	Bme	COG0750	Predicted membrane-associated Zn-dependent proteases 1	Cell wall/membrane/envelope biogenesis	M
BrucellaGL002628	BMEI0828	Bme	COG0575	CDP-diglyceride synthetase	Lipid transport and metabolism	I
BrucellaGL002629	BMEI0827	Bme	COG0020	Undecaprenyl pyrophosphate synthase	Lipid transport and metabolism	I
BrucellaGL002630	BMEI0826	Bme	COG0233	Ribosome recycling factor	Translation, ribosomal structure and biogenesis	J
BrucellaGL002631	BMEI0825	Bme	COG0528	Uridylate kinase	Nucleotide transport and metabolism	F
BrucellaGL002632	BMEI0824	Bme	COG0264	Translation elongation factor Ts	Translation, ribosomal structure and biogenesis	J
BrucellaGL002633	BMEI0823	Bme	COG0052	Ribosomal protein S2	Translation, ribosomal structure and biogenesis	J
BrucellaGL002634	BMEI0822	Bme	COG4875	Uncharacterized protein conserved in bacteria with a cystatin-like fold	Function unknown	S
BrucellaGL002636	BMEI0820	Bme	COG0251	Putative translation initiation inhibitor, yjgF family	Translation, ribosomal structure and biogenesis	J
BrucellaGL002637	BMEI0819	Bme	COG0584	Glycerophosphoryl diester phosphodiesterase	Energy production and conversion	C
BrucellaGL002638	BMEI0818	Bme	COG3146	Uncharacterized protein conserved in bacteria	Function unknown	S
BrucellaGL002639	BMEI0817	Bme	COG0537	Diadenosine tetraphosphate (Ap4A) hydrolase and other HIT family hydrolases	Nucleotide transport and metabolism	F
BrucellaGL002640	BMEI0816	Bme	COG0542	ATPases with chaperone activity, ATP-binding subunit	Posttranslational modification, protein turnover, chaperones	O
BrucellaGL002641	BMEI0815	Bme	COG2127	Uncharacterized conserved protein	Function unknown	S

BrucellaGL002643	BMEI0814	Bme	COG1686	D-alanyl-D-alanine carboxypeptidase	Cell wall/membrane/envelope biogenesis	M
BrucellaGL002645	BMEI0812	Bme	COG5458	Uncharacterized conserved protein	Function unknown	S
BrucellaGL002646	BMEI0811	Bme	COG1760	L-serine deaminase	Amino acid transport and metabolism	E
BrucellaGL002648	BMEI0810	Bme	COG1434	Uncharacterized conserved protein	Function unknown	S
BrucellaGL002650	AGc2846	Atu	COG3503	Predicted membrane protein	Function unknown	S
BrucellaGL002651	BMEI0808	Bme	COG0789	Predicted transcriptional regulators	Transcription	K
BrucellaGL002655	BMEI0804	Bme	COG0780	Enzyme related to GTP cyclohydrolase I	General function prediction only	R
BrucellaGL002657	BMEI0803	Bme	COG3803	Uncharacterized protein conserved in bacteria	Function unknown	S
BrucellaGL002658	BMEI0802_2	Bme	COG3800	Predicted transcriptional regulator	General function prediction only	R
BrucellaGL002659	BMEI0801	Bme	COG4799	Acetyl-CoA carboxylase, carboxyltransferase component (subunits alpha and beta)	Lipid transport and metabolism	I
BrucellaGL002660	BMEI0800	Bme	COG4770	Acetyl/propionyl-CoA carboxylase, alpha subunit	Lipid transport and metabolism	I
BrucellaGL002661	BMEI0799_1	Bme	COG1884	Methylmalonyl-CoA mutase, N-terminal domain/subunit	Lipid transport and metabolism	I
BrucellaGL002663	BMEI0797	Bme	COG4666	TRAP-type uncharacterized transport system, fused permease components	General function prediction only	R
BrucellaGL002665	BMEI0796	Bme	COG2358	TRAP-type uncharacterized transport system, periplasmic component	General function prediction only	R
BrucellaGL002666	BMEI0795	Bme	COG0796	Glutamate racemase	Cell wall/membrane/envelope biogenesis	M
BrucellaGL002667	BMEI0794	Bme	COG0565	rRNA methylase	Translation, ribosomal structure and biogenesis	J
BrucellaGL002668	BMEI0793	Bme	COG0693	Putative intracellular protease/amidase	General function prediction only	R
BrucellaGL002669	BMEI0792	Bme	COG4321	Uncharacterized protein related to arylsulfate sulfotransferase involved in siderophore biosynthesis	General function prediction only	R
BrucellaGL002670	BMEI0791	Bme	COG0538	Isocitrate dehydrogenases	Energy production and conversion	C
BrucellaGL002673	BMEI0790	Bme	COG1785	Alkaline phosphatase	Inorganic ion transport and metabolism	P
BrucellaGL002674	BMEI0789	Bme	COG0013	Alanyl-tRNA synthetase	Translation, ribosomal structure and biogenesis	J

BrucellaGL002675	BMEI0787	Bme	COG0468	RecA/RadA recombinase	Replication, recombination and repair	L
BrucellaGL002677	BMEI0786	Bme	COG2885	Outer membrane protein and related peptidoglycan-associated (lipo)proteins	Cell wall/membrane/envelope biogenesis	M
BrucellaGL002678	BMEI0785	Bme	COG4764	Uncharacterized protein conserved in bacteria	Function unknown	S
BrucellaGL002679	BMEI0784	Bme	COG2256	ATPase related to the helicase subunit of the Holliday junction resolvase	Replication, recombination and repair	L
BrucellaGL002680	BMEI0783	Bme	COG0265	Trypsin-like serine proteases, typically periplasmic, contain C-terminal PDZ domain	Posttranslational modification, protein turnover, chaperones	O
BrucellaGL002681	BMEI0782	Bme	COG0203	Ribosomal protein L17	Translation, ribosomal structure and biogenesis	J
BrucellaGL002683	BMEI0781	Bme	COG0202	DNA-directed RNA polymerase, alpha subunit/40 kD subunit	Transcription	K
BrucellaGL002684	BMEI0780	Bme	COG0100	Ribosomal protein S11	Translation, ribosomal structure and biogenesis	J
BrucellaGL002685	BMEI0779	Bme	COG0099	Ribosomal protein S13	Translation, ribosomal structure and biogenesis	J
BrucellaGL002686	BMEI0778	Bme	COG0563	Adenylate kinase and related kinases	Nucleotide transport and metabolism	F
BrucellaGL002687	BMEI0777	Bme	COG0201	Preprotein translocase subunit SecY	Intracellular trafficking, secretion, and vesicular transport	U
BrucellaGL002688	BMEI0776	Bme	COG0200	Ribosomal protein L15	Translation, ribosomal structure and biogenesis	J
BrucellaGL002689	BMEI0775	Bme	COG1841	Ribosomal protein L30/L7E	Translation, ribosomal structure and biogenesis	J
BrucellaGL002690	BMEI0774	Bme	COG0098	Ribosomal protein S5	Translation, ribosomal structure and biogenesis	J
BrucellaGL002691	BMEI0773	Bme	COG0256	Ribosomal protein L18	Translation, ribosomal structure and biogenesis	J
BrucellaGL002692	BMEI0772	Bme	COG0097	Ribosomal protein L6P/L9E	Translation, ribosomal structure and biogenesis	J
BrucellaGL002693	BMEI0771	Bme	COG0096	Ribosomal protein S8	Translation, ribosomal structure and biogenesis	J
BrucellaGL002694	BMEI0770	Bme	COG0199	Ribosomal protein S14	Translation, ribosomal structure and biogenesis	J

BrucellaGL002695	BMEI0769	Bme	COG0094	Ribosomal protein L5	Translation, ribosomal structure and biogenesis	J
BrucellaGL002696	BMEI0768	Bme	COG0198	Ribosomal protein L24	Translation, ribosomal structure and biogenesis	J
BrucellaGL002697	BMEI0767	Bme	COG0093	Ribosomal protein L14	Translation, ribosomal structure and biogenesis	J
BrucellaGL002698	BMEI0766	Bme	COG0186	Ribosomal protein S17	Translation, ribosomal structure and biogenesis	J
BrucellaGL002700	BMEI0763	Bme	COG0092	Ribosomal protein S3	Translation, ribosomal structure and biogenesis	J
BrucellaGL002701	BMEI0762	Bme	COG0091	Ribosomal protein L22	Translation, ribosomal structure and biogenesis	J
BrucellaGL002702	SMc01305	Sme	COG0185	Ribosomal protein S19	Translation, ribosomal structure and biogenesis	J
BrucellaGL002703	BMEI0760	Bme	COG0090	Ribosomal protein L2	Translation, ribosomal structure and biogenesis	J
BrucellaGL002704	BMEI0759	Bme	COG0089	Ribosomal protein L23	Translation, ribosomal structure and biogenesis	J
BrucellaGL002705	mlr0292	Mlo	COG0088	Ribosomal protein L4	Translation, ribosomal structure and biogenesis	J
BrucellaGL002706	BMEI0757	Bme	COG0087	Ribosomal protein L3	Translation, ribosomal structure and biogenesis	J
BrucellaGL002707	BMEI0756	Bme	COG0051	Ribosomal protein S10	Translation, ribosomal structure and biogenesis	J
BrucellaGL002708	BMEI0742	Bme	COG0050	GTPases - translation elongation factors	Translation, ribosomal structure and biogenesis	J
BrucellaGL002709	BMEI0754	Bme	COG0480	Translation elongation factors (GTPases)	Translation, ribosomal structure and biogenesis	J
BrucellaGL002710	BMEI0753	Bme	COG0049	Ribosomal protein S7	Translation, ribosomal structure and biogenesis	J
BrucellaGL002713	BMEI0750	Bme	COG0086	DNA-directed RNA polymerase, beta' subunit/160 kD subunit	Transcription	K
BrucellaGL002714	BMEI0749	Bme	COG0085	DNA-directed RNA polymerase, beta subunit/140 kD subunit	Transcription	K

BrucellaGL002715	BMEI0748	Bme	COG0222	Ribosomal protein L7/L12	Translation, ribosomal structure and biogenesis	J
BrucellaGL002716	BMEI0747	Bme	COG0244	Ribosomal protein L10	Translation, ribosomal structure and biogenesis	J
BrucellaGL002717	BMEI0746	Bme	COG0081	Ribosomal protein L1	Translation, ribosomal structure and biogenesis	J
BrucellaGL002718	BMEI0745	Bme	COG0080	Ribosomal protein L11	Translation, ribosomal structure and biogenesis	J
BrucellaGL002719	BMEI0744	Bme	COG0250	Transcription antiterminator	Transcription	K
BrucellaGL002720	BMEI0743	Bme	COG0690	Preprotein translocase subunit SecE	Intracellular trafficking, secretion, and vesicular transport	U
BrucellaGL002722	BMEI0741	Bme	COG0566	rRNA methylases	Translation, ribosomal structure and biogenesis	J
BrucellaGL002723	BMEI0740	Bme	COG0517	FOG: CBS domain	General function prediction only	R
BrucellaGL002724	BMEI0739	Bme	COG0705	Uncharacterized membrane protein (homolog of Drosophila rhomboid)	General function prediction only	R
BrucellaGL002725	BMEI0738	Bme	COG5388	Uncharacterized protein conserved in bacteria	Function unknown	S
BrucellaGL002726	BMEI0737	Bme	COG3672	Predicted periplasmic protein	Function unknown	S
BrucellaGL002727	BMEI0736	Bme	COG0663	Carbonic anhydrases/acetyltransferases, isoleucine patch superfamily	General function prediction only	R
BrucellaGL002729	BMEI1001	Bme	COG3293	Transposase and inactivated derivatives	Replication, recombination and repair	L
BrucellaGL002731	BMEI0734	Bme	COG1045	Serine acetyltransferase	Amino acid transport and metabolism	E
BrucellaGL002732	BMEI0733	Bme	COG0596	Predicted hydrolases or acyltransferases (alpha/beta hydrolase superfamily)	General function prediction only	R
BrucellaGL002733	BMEI0732	Bme	COG1430	Uncharacterized conserved protein	Function unknown	S
BrucellaGL002734	BMEI0731	Bme	COG1278	Cold shock proteins	Transcription	K
BrucellaGL002736	BMEI0730	Bme	COG0346	Lactoylglutathione lyase and related lyases	Amino acid transport and metabolism	E
BrucellaGL002737	BMEI0729	Bme	COG4395	Uncharacterized protein conserved in bacteria	Function unknown	S
BrucellaGL002738	BMEI0728	Bme	COG0608	Single-stranded DNA-specific exonuclease	Replication, recombination and repair	L
BrucellaGL002740	BMEI0727	Bme	COG1181	D-alanine-D-alanine ligase and related ATP-grasp enzymes	Cell wall/membrane/envelope biogenesis	M
BrucellaGL002742	BMEI0726	Bme	COG1494	Fructose-1,6-bisphosphatase/sedoheptulose 1,7-bisphosphatase and related proteins	Carbohydrate transport and metabolism	G

BrucellaGL002743	BMEI0725	Bme	COG0460	Homoserine dehydrogenase	Amino acid transport and metabolism	E
BrucellaGL002744	BMEI0724	Bme	COG0436	Aspartate/tyrosine/aromatic aminotransferase	Amino acid transport and metabolism	E
BrucellaGL002747	BMEI0722	Bme	COG3108	Uncharacterized protein conserved in bacteria	Function unknown	S
BrucellaGL002749	BMEI0721	Bme	COG3553	Uncharacterized protein conserved in bacteria	Function unknown	S
BrucellaGL002750	BMEI0720	Bme	COG1489	DNA-binding protein, stimulates sugar fermentation	General function prediction only	R
BrucellaGL002751	BMEI0719	Bme	COG0024	Methionine aminopeptidase	Translation, ribosomal structure and biogenesis	J
BrucellaGL002752	BMEI0718	Bme	COG2003	DNA repair proteins	Replication, recombination and repair	L
BrucellaGL002753	BMEI0717	Bme	COG3637	Opacity protein and related surface antigens	Cell wall/membrane/envelope biogenesis	M
BrucellaGL002755	BMEI0716_1	Bme	COG2241	Precorrin-6B methylase 1	Coenzyme transport and metabolism	H
BrucellaGL002756	BMEI0715	Bme	COG0155	Sulfite reductase, beta subunit (hemoprotein)	Inorganic ion transport and metabolism	P
BrucellaGL002757	BMEI0714	Bme	COG2082	Precorrin isomerase	Coenzyme transport and metabolism	H
BrucellaGL002758	BMEI0713	Bme	COG2243	Precorrin-2 methylase	Coenzyme transport and metabolism	H
BrucellaGL002759	BMEI0712_1	Bme	COG2073	Cobalamin biosynthesis protein CbiG	Coenzyme transport and metabolism	H
BrucellaGL002764	BMEI0709	Bme	COG1853	Conserved protein/domain typically associated with flavoprotein oxygenases, DIM6/NTAB family	General function prediction only	R
BrucellaGL002765	BMEI0708	Bme	COG0683	ABC-type branched-chain amino acid transport systems, periplasmic component	Amino acid transport and metabolism	E
BrucellaGL002766	BMEI0707	Bme	COG1270	Cobalamin biosynthesis protein CobD/CbiB	Coenzyme transport and metabolism	H
BrucellaGL002767	BMEI0706	Bme	COG0079	Histidinol-phosphate/aromatic aminotransferase and cobyric acid decarboxylase	Amino acid transport and metabolism	E
BrucellaGL002768	BMEI0705	Bme	COG1797	Cobyrinic acid a,c-diamide synthase	Coenzyme transport and metabolism	H
BrucellaGL002769	BMEI0704	Bme	COG0007	Uroporphyrinogen-III methylase	Coenzyme transport and metabolism	H
BrucellaGL002770	BMEI0703	Bme	COG1903	Cobalamin biosynthesis protein CbiD	Coenzyme transport and metabolism	H
BrucellaGL002771	BMEI0702	Bme	COG2099	Precorrin-6x reductase	Coenzyme transport and metabolism	H
BrucellaGL002772	BMEI0701	Bme	COG2875	Precorrin-4 methylase	Coenzyme transport and metabolism	H
BrucellaGL002773	BMEI0700	Bme	COG2073	Cobalamin biosynthesis protein CbiG	Coenzyme transport and metabolism	H
BrucellaGL002774	BMEI0699	Bme	COG4103	Uncharacterized protein conserved in bacteria	Function unknown	S
BrucellaGL002775	mlr1193	Mlo	COG0730	Predicted permeases	General function prediction only	R

BrucellaGL002776	BMEI0697	Bme	COG2510	Predicted membrane protein	Function unknown	S
BrucellaGL002777	BMEI0696	Bme	COG2109	ATP:corrinoid adenosyltransferase	Coenzyme transport and metabolism	H
BrucellaGL002778	BMEI0695	Bme	COG1429	Cobalamin biosynthesis protein CobN and related Mg-chelatases	Coenzyme transport and metabolism	H
BrucellaGL002779	BMEI0694	Bme	COG0523	Putative GTPases (G3E family)	General function prediction only	R
BrucellaGL002780	BMEI0693	Bme	COG2087	Adenosyl cobinamide kinase/adenosyl cobinamide phosphate guanylyltransferase	Coenzyme transport and metabolism	H
BrucellaGL002781	BMEI0692	Bme	COG5446	Predicted integral membrane protein	Function unknown	S
BrucellaGL002783	BMEI0690	Bme	COG1492	Cobyric acid synthase	Coenzyme transport and metabolism	H
BrucellaGL002784	BMEI0689	Bme	COG1960	Acyl-CoA dehydrogenases	Lipid transport and metabolism	I
BrucellaGL002785	BMEI0688	Bme	COG2084	3-hydroxyisobutyrate dehydrogenase and related beta-hydroxyacid dehydrogenases	Lipid transport and metabolism	I
BrucellaGL002787	BMEI0686	Bme	COG0583	Transcriptional regulator	Transcription	K
BrucellaGL002788	BMEI0685	Bme	COG4977	Transcriptional regulator containing an amidase domain and an AraC-type DNA-binding HTH domain	Transcription	K
BrucellaGL002789	mll1258_1	Mlo	COG0665	Glycine/D-amino acid oxidases (deaminating)	Amino acid transport and metabolism	E
BrucellaGL002790	BMEI0684_1	Bme	COG0665	Glycine/D-amino acid oxidases (deaminating)	Amino acid transport and metabolism	E
BrucellaGL002791	BMEI0684_2	Bme	COG0404	Glycine cleavage system T protein (aminomethyltransferase)	Amino acid transport and metabolism	E
BrucellaGL002793	BMEI0682	Bme	COG3264	Small-conductance mechanosensitive channel	Cell wall/membrane/envelope biogenesis	M
BrucellaGL002795	BMEI0680_1	Bme	COG0342	Preprotein translocase subunit SecD	Intracellular trafficking, secretion, and vesicular transport	U
BrucellaGL002796	BMEI0679_1	Bme	COG4651	Kef-type K+ transport system, predicted NAD-binding component	Inorganic ion transport and metabolism	P
BrucellaGL002797	BMEI0678	Bme	COG0392	Predicted integral membrane protein	Function unknown	S
BrucellaGL002798	BMEI0677	Bme	COG3545	Predicted esterase of the alpha/beta hydrolase fold	General function prediction only	R
BrucellaGL002799	BMEI0676	Bme	COG3545	Predicted esterase of the alpha/beta hydrolase fold	General function prediction only	R
BrucellaGL002800	BMEI0675	Bme	COG4208	ABC-type sulfate transport system, permease component	Inorganic ion transport and metabolism	P

BrucellaGL002801	BMEI0674	Bme	COG0555	ABC-type sulfate transport system, permease component	Posttranslational modification, protein turnover, chaperones	O
BrucellaGL002802	BMEI0673	Bme	COG4150	ABC-type sulfate transport system, periplasmic component	Inorganic ion transport and metabolism	P
BrucellaGL002804	BMEI0671_2	Bme	COG1253	Hemolysins and related proteins containing CBS domains	General function prediction only	R
BrucellaGL002805	BMEI0670	Bme	COG0586	Uncharacterized membrane-associated protein	Function unknown	S
BrucellaGL002807	BMEI0667	Bme	COG4154	Fucose dissimilation pathway protein FucU	Carbohydrate transport and metabolism	G
BrucellaGL002808	TM0325	Tma	COG1028	Dehydrogenases with different specificities (related to short-chain alcohol dehydrogenases)	Lipid transport and metabolism	I
BrucellaGL002809	BMEI0665	Bme	COG1129	ABC-type sugar transport system, ATPase component	Carbohydrate transport and metabolism	G
BrucellaGL002810	BMEI0664	Bme	COG1172	Ribose/xylose/arabinose/galactoside ABC-type transport systems, permease components	Carbohydrate transport and metabolism	G
BrucellaGL002811	BMEI0663	Bme	COG1879	ABC-type sugar transport system, periplasmic component	Carbohydrate transport and metabolism	G
BrucellaGL002812	mll1004	Mlo	COG1879	ABC-type sugar transport system, periplasmic component	Carbohydrate transport and metabolism	G
BrucellaGL002813	BMEI0661	Bme	COG0673	Predicted dehydrogenases and related proteins	General function prediction only	R
BrucellaGL002814	BMEI0660	Bme	COG1120	ABC-type cobalamin/Fe3+-siderophores transport systems, ATPase components	Inorganic ion transport and metabolism	P
BrucellaGL002815	BMEI0659	Bme	COG0609	ABC-type Fe3+-siderophore transport system, permease component	Inorganic ion transport and metabolism	P
BrucellaGL002816	BMEI0658	Bme	COG0614	ABC-type Fe3+-hydroxamate transport system, periplasmic component	Inorganic ion transport and metabolism	P
BrucellaGL002817	BMEI0657	Bme	COG4206	Outer membrane cobalamin receptor protein	Coenzyme transport and metabolism	H
BrucellaGL002818	BMEI0656	Bme	COG0842	ABC-type multidrug transport system, permease component	Defense mechanisms	V
BrucellaGL002819	BMEI0655	Bme	COG0842	ABC-type multidrug transport system, permease component	Defense mechanisms	V
BrucellaGL002820	BMEI0654_1	Bme	COG1131	ABC-type multidrug transport system, ATPase component	Defense mechanisms	V

BrucellaGL002821	BMEI0653	Bme	COG0845	Membrane-fusion protein	Cell wall/membrane/envelope biogenesis	M
BrucellaGL002822	BMEI0651	Bme	COG1649	Uncharacterized protein conserved in bacteria	Function unknown	S
BrucellaGL002824	BMEI0649	Bme	COG0831	Urea amidohydrolase (urease) gamma subunit	Amino acid transport and metabolism	E
BrucellaGL002825	BMEI0648	Bme	COG0832	Urea amidohydrolase (urease) beta subunit	Amino acid transport and metabolism	E
BrucellaGL002826	BMEI0647	Bme	COG0804	Urea amidohydrolase (urease) alpha subunit	Amino acid transport and metabolism	E
BrucellaGL002827	BMEI0646	Bme	COG2371	Urease accessory protein UreE	Posttranslational modification, protein turnover, chaperones	O
BrucellaGL002828	BMEI0645	Bme	COG0830	Urease accessory protein UreF	Posttranslational modification, protein turnover, chaperones	O
BrucellaGL002829	BMEI0644	Bme	COG0378	Ni2+-binding GTPase involved in regulation of expression and maturation of urease and hydrogenase	Posttranslational modification, protein turnover, chaperones	O
BrucellaGL002830	BMEI0643	Bme	COG0829	Urease accessory protein UreH	Posttranslational modification, protein turnover, chaperones	O
BrucellaGL002831	BMEI0642	Bme	COG4413	Urea transporter	Amino acid transport and metabolism	E
BrucellaGL002832	BMEI0641	Bme	COG5266	ABC-type Co2+ transport system, periplasmic component	Inorganic ion transport and metabolism	P
BrucellaGL002833	BMEI0640	Bme	COG0310	ABC-type Co2+ transport system, permease component	Inorganic ion transport and metabolism	P
BrucellaGL002835	BMEI0637	Bme	COG0619	ABC-type cobalt transport system, permease component CbiQ and related transporters	Inorganic ion transport and metabolism	P
BrucellaGL002836	BMEI0635	Bme	COG1122	ABC-type cobalt transport system, ATPase component	Inorganic ion transport and metabolism	P
BrucellaGL002837	BMEI0634_1	Bme	COG0239	Integral membrane protein possibly involved in chromosome condensation	Cell cycle control, cell division, chromosome partitioning	D
BrucellaGL002838	BMEI0633	Bme	COG0239	Integral membrane protein possibly involved in chromosome condensation	Cell cycle control, cell division, chromosome partitioning	D
BrucellaGL002840	BMEI0631	Bme	COG3467	Predicted flavin-nucleotide-binding protein	General function prediction only	R
BrucellaGL002842	BMEI0630	Bme	COG0384	Predicted epimerase, PhzC/PhzF homolog	General function prediction only	R
BrucellaGL002843	BMEI0629	Bme	COG0494	NTP pyrophosphohydrolases including oxidative damage repair enzymes	Replication, recombination and repair	L

BrucellaGL002846	BMEI0626	Bme	COG1167	Transcriptional regulators containing a DNA-binding HTH domain and an aminotransferase domain (MocR family) and their eukaryotic orthologs	Transcription	K
BrucellaGL002847	BMEI0624	Bme	COG0059	Ketol-acid reductoisomerase	Amino acid transport and metabolism	E
BrucellaGL002848	BMEI0623	Bme	COG1309	Transcriptional regulator	Transcription	K
BrucellaGL002850	BMEI0622	Bme	COG3158	K+ transporter	Inorganic ion transport and metabolism	P
BrucellaGL002851	BMEI0621	Bme	COG0854	Pyridoxal phosphate biosynthesis protein	Coenzyme transport and metabolism	H
BrucellaGL002853	BMEI0619	Bme	COG0507	ATP-dependent exoDNAse (exonuclease V), alpha subunit - helicase superfamily I member	Replication, recombination and repair	L
BrucellaGL002855	BMEI0618	Bme	COG0440	Acetolactate synthase, small (regulatory) subunit	Amino acid transport and metabolism	E
BrucellaGL002856	BMEI0617	Bme	COG0028	Thiamine pyrophosphate-requiring enzymes [acetolactate synthase, pyruvate dehydrogenase (cytochrome), glyoxylate carboligase, phosphonopyruvate decarboxylase]	Amino acid transport and metabolism	E
BrucellaGL002858	BMEI0616	Bme	COG0324	tRNA delta(2)-isopentenylpyrophosphate transferase	Translation, ribosomal structure and biogenesis	J
BrucellaGL002859	BMEI0615	Bme	COG0560	Phosphoserine phosphatase	Amino acid transport and metabolism	E
BrucellaGL002860	BMEI0614	Bme	COG2081	Predicted flavoproteins	General function prediction only	R
BrucellaGL002861	BMEI0614	Bme	COG2081	Predicted flavoproteins	General function prediction only	R
BrucellaGL002862	BMEI0613	Bme	COG0265	Trypsin-like serine proteases, typically periplasmic, contain C-terminal PDZ domain	Posttranslational modification, protein turnover, chaperones	O
BrucellaGL002863	BMEI0612	Bme	COG3242	Uncharacterized protein conserved in bacteria	Function unknown	S
BrucellaGL002864	BMEI0611	Bme	COG0330	Membrane protease subunits, stomatin/prohibitin homologs	Posttranslational modification, protein turnover, chaperones	O
BrucellaGL002865	BMEI0610	Bme	COG0330	Membrane protease subunits, stomatin/prohibitin homologs	Posttranslational modification, protein turnover, chaperones	O
BrucellaGL002866	BMEI0609	Bme	COG0262	Dihydrofolate reductase	Coenzyme transport and metabolism	H
BrucellaGL002867	BMEI0608	Bme	COG0207	Thymidylate synthase	Nucleotide transport and metabolism	F
BrucellaGL002870	mll1470	Mlo	COG0477	Permeases of the major facilitator superfamily	Carbohydrate transport and metabolism	G
BrucellaGL002872	BMEI0604	Bme	COG1309	Transcriptional regulator	Transcription	K

BrucellaGL002873	BMEI0603	Bme	COG3814	Uncharacterized protein conserved in bacteria	Function unknown	S
BrucellaGL002876	BMEI0601	Bme	COG4321	Uncharacterized protein related to arylsulfate sulfotransferase involved in siderophore biosynthesis	General function prediction only	R
BrucellaGL002877	RC0472	Rco	COG2982	Uncharacterized protein involved in outer membrane biogenesis	Cell wall/membrane/envelope biogenesis	M
BrucellaGL002879	BMEI0599	Bme	COG0277	FAD/FMN-containing dehydrogenases	Energy production and conversion	C
BrucellaGL002880	BMEI0598	Bme	COG4702	Uncharacterized conserved protein	Function unknown	S
BrucellaGL002882	BMEI0596	Bme	COG0210	Superfamily I DNA and RNA helicases	Replication, recombination and repair	L
BrucellaGL002883	BMEI0595	Bme	COG3045	Uncharacterized protein conserved in bacteria	Function unknown	S
BrucellaGL002884	BMEI0594	Bme	COG0110	Acetyltransferase (isoleucine patch superfamily)	General function prediction only	R
BrucellaGL002885	BMEI0593	Bme	COG1999	Uncharacterized protein SCO1/SenC/PrrC, involved in biogenesis of respiratory and photosynthetic systems	General function prediction only	R
BrucellaGL002886	BMEI0592	Bme	COG2264	Ribosomal protein L11 methylase	Translation, ribosomal structure and biogenesis	J
BrucellaGL002887	BMEI0591	Bme	COG0006	Xaa-Pro aminopeptidase	Amino acid transport and metabolism	E
BrucellaGL002888	BMEI0590	Bme	COG5587	Uncharacterized conserved protein	Function unknown	S
BrucellaGL002889	BMEI0589	Bme	COG0272	NAD-dependent DNA ligase (contains BRCT domain type II)	Replication, recombination and repair	L
BrucellaGL002890	BMEI0588	Bme	COG0497	ATPase involved in DNA repair	Replication, recombination and repair	L
BrucellaGL002891	BMEI0587	Bme	COG4105	DNA uptake lipoprotein	General function prediction only	R
BrucellaGL002892	BMEI0586	Bme	COG0774	UDP-3-O-acyl-N-acetylglucosamine deacetylase	Cell wall/membrane/envelope biogenesis	M
BrucellaGL002893	BMEI0585	Bme	COG0206	Cell division GTPase	Cell cycle control, cell division, chromosome partitioning	D
BrucellaGL002894	BMEI0584	Bme	COG0849	Actin-like ATPase involved in cell division	Cell cycle control, cell division, chromosome partitioning	D
BrucellaGL002895	BMEI0583	Bme	COG1589	Cell division septal protein	Cell wall/membrane/envelope biogenesis	M
BrucellaGL002896	BMEI0582	Bme	COG1181	D-alanine-D-alanine ligase and related ATP-grasp enzymes	Cell wall/membrane/envelope biogenesis	M

BrucellaGL002897	BMEI0581	Bme	COG0812	UDP-N-acetylmuramate dehydrogenase	Cell wall/membrane/envelope biogenesis	M
BrucellaGL002898	BMEI0580	Bme	COG0773	UDP-N-acetylmuramate-alanine ligase	Cell wall/membrane/envelope biogenesis	M
BrucellaGL002899	BMEI0579	Bme	COG0707	UDP-N-acetylglucosamine:LPS N-acetylglucosamine transferase	Cell wall/membrane/envelope biogenesis	M
BrucellaGL002900	BMEI0578	Bme	COG0772	Bacterial cell division membrane protein	Cell cycle control, cell division, chromosome partitioning	D
BrucellaGL002901	BMEI0577	Bme	COG0771	UDP-N-acetylmuramoylalanine-D-glutamate ligase	Cell wall/membrane/envelope biogenesis	M
BrucellaGL002902	BMEI0576	Bme	COG0472	UDP-N-acetylmuramyl pentapeptide phosphotransferase/UDP-N-acetylglucosamine-1-phosphate transferase	Cell wall/membrane/envelope biogenesis	M
BrucellaGL002903	BMEI0575	Bme	COG0770	UDP-N-acetylmuramyl pentapeptide synthase	Cell wall/membrane/envelope biogenesis	M
BrucellaGL002904	BMEI0574	Bme	COG0769	UDP-N-acetylmuramyl tripeptide synthase	Cell wall/membrane/envelope biogenesis	M
BrucellaGL002905	BMEI0573	Bme	COG0768	Cell division protein FtsI/penicillin-binding protein 2	Cell wall/membrane/envelope biogenesis	M
BrucellaGL002906	BMEI0572	Bme	COG5462	Predicted secreted (periplasmic) protein	Function unknown	S
BrucellaGL002907	BMEI0571	Bme	COG0275	Predicted S-adenosylmethionine-dependent methyltransferase involved in cell envelope biogenesis	Cell wall/membrane/envelope biogenesis	M
BrucellaGL002909	BMEI0569	Bme	COG1914	Mn2+ and Fe2+ transporters of the NRAMP family	Inorganic ion transport and metabolism	P
BrucellaGL002910	BMEI0566	Bme	COG0741	Soluble lytic murein transglycosylase and related regulatory proteins (some contain LysM/invasin domains)	Cell wall/membrane/envelope biogenesis	M
BrucellaGL002911	BMEI0565	Bme	COG3023	Negative regulator of beta-lactamase expression	Defense mechanisms	V
BrucellaGL002912	BMEI0564	Bme	COG1076	DnaJ-domain-containing proteins 1	Posttranslational modification, protein turnover, chaperones	O
BrucellaGL002914	BMEI0562	Bme	COG3757	Lyzozyme M1 (1,4-beta-N-acetylmuramidase)	Cell wall/membrane/envelope biogenesis	M

BrucellaGL002915	BMEI0561	Bme	COG2951	Membrane-bound lytic murein transglycosylase B	Cell wall/membrane/envelope biogenesis	M
BrucellaGL002916	BMEI0560	Bme	COG0679	Predicted permeases	General function prediction only	R
BrucellaGL002918	BMEI0559	Bme	COG0685	5,10-methylenetetrahydrofolate reductase	Amino acid transport and metabolism	E
BrucellaGL002919	BMEI0558_2	Bme	COG0500	SAM-dependent methyltransferases	Secondary metabolites biosynthesis, transport and catabolism	Q
BrucellaGL002920	BMEI0557	Bme	COG5586	Uncharacterized conserved protein	Function unknown	S
BrucellaGL002921	BMEI0556	Bme	COG0477	Permeases of the major facilitator superfamily	Carbohydrate transport and metabolism	G
BrucellaGL002922	BMEI0554	Bme	COG4106	Trans-aconitate methyltransferase	General function prediction only	R
BrucellaGL002923	BMEI0553	Bme	COG0488	ATPase components of ABC transporters with duplicated ATPase domains	General function prediction only	R
BrucellaGL002925	BMEI0552	Bme	COG2267	Lysophospholipase	Lipid transport and metabolism	I
BrucellaGL002927	BMEI0550	Bme	COG3822	ABC-type sugar transport system, auxiliary component	General function prediction only	R
BrucellaGL002928	BMEI0549	Bme	COG4638	Phenylpropionate dioxygenase and related ring-hydroxylating dioxygenases, large terminal subunit	Inorganic ion transport and metabolism	P
BrucellaGL002929	BMEI0548	Bme	COG1018	Flavodoxin reductases (ferredoxin-NADPH reductases) family 1	Energy production and conversion	C
BrucellaGL002930	BMEI0547	Bme	COG2824	Uncharacterized Zn-ribbon-containing protein involved in phosphonate metabolism	Inorganic ion transport and metabolism	P
BrucellaGL002931	BMEI0546	Bme	COG1335	Amidases related to nicotinamidase	Secondary metabolites biosynthesis, transport and catabolism	Q
BrucellaGL002932	BMEI0545	Bme	COG2899	Uncharacterized protein conserved in bacteria	Function unknown	S
BrucellaGL002934	BMEI0543	Bme	COG3049	Penicillin V acylase and related amidases	Cell wall/membrane/envelope biogenesis	M
BrucellaGL002940	BMEI0537	Bme	COG0582	Integrase	Replication, recombination and repair	L
BrucellaGL002941	BMEI0536	Bme	COG2968	Uncharacterized conserved protein	Function unknown	S
BrucellaGL002943	BMEI0534	Bme	COG2983	Uncharacterized conserved protein	Function unknown	S
BrucellaGL002944	BMEI0533	Bme	COG5453	Uncharacterized conserved protein	Function unknown	S
BrucellaGL002945	BMEI0532	Bme	COG0568	DNA-directed RNA polymerase, sigma subunit (sigma70/sigma32)	Transcription	K

BrucellaGL002946	BMEI0530	Bme	COG0358	DNA primase (bacterial type)	Replication, recombination and repair	L
BrucellaGL002947	mlr2470	Mlo	COG1283	Na+/phosphate symporter	Inorganic ion transport and metabolism	P
BrucellaGL002948	BMEI0527	Bme	COG1610	Uncharacterized conserved protein	Function unknown	S
BrucellaGL002949	BMEI0526	Bme	COG0505	Carbamoylphosphate synthase small subunit	Amino acid transport and metabolism	E
BrucellaGL002951	BMEI0525	Bme	COG2321	Predicted metalloprotease	General function prediction only	R
BrucellaGL002952	BMEI0524	Bme	COG5395	Predicted membrane protein	Function unknown	S
BrucellaGL002953	BMEI0523	Bme	COG3279	Response regulator of the LytR/AlgR family	Transcription	K
BrucellaGL002954	BMEI0522	Bme	COG0458	Carbamoylphosphate synthase large subunit (split gene in MJ)	Amino acid transport and metabolism	E
BrucellaGL002955	BMEI0520	Bme	COG4178	ABC-type uncharacterized transport system, permease and ATPase components	General function prediction only	R
BrucellaGL002956	BMEI0519	Bme	COG0494	NTP pyrophosphohydrolases including oxidative damage repair enzymes	Replication, recombination and repair	L
BrucellaGL002957	BMEI0517	Bme	COG0625	Glutathione S-transferase	Posttranslational modification, protein turnover, chaperones	O
BrucellaGL002958	BMEI0516	Bme	COG0436	Aspartate/tyrosine/aromatic aminotransferase	Amino acid transport and metabolism	E
BrucellaGL002960	BMEI0513	Bme	COG0583	Transcriptional regulator	Transcription	K
BrucellaGL002961	BMEI0512	Bme	COG0492	Thioredoxin reductase	Posttranslational modification, protein turnover, chaperones	O
BrucellaGL002962	BMEI0511	Bme	COG0168	Trk-type K+ transport systems, membrane components	Inorganic ion transport and metabolism	P
BrucellaGL002963	BMEI0510	Bme	COG1522	Transcriptional regulators	Transcription	K
BrucellaGL002964	BMEI0509	Bme	COG0438	Glycosyltransferase	Cell wall/membrane/envelope biogenesis	M
BrucellaGL002965	BMEI0508	Bme	COG0782	Transcription elongation factor	Transcription	K
BrucellaGL002968	BMEI0506	Bme	COG0697	Permeases of the drug/metabolite transporter (DMT) superfamily	Carbohydrate transport and metabolism	G
BrucellaGL002969	BMEI0505	Bme	COG3169	Uncharacterized protein conserved in bacteria	Function unknown	S
BrucellaGL002970	BMEI0504	Bme	COG3921	Uncharacterized protein conserved in bacteria	Function unknown	S
BrucellaGL002971	BMEI0503	Bme	COG1607	Acyl-CoA hydrolase	Lipid transport and metabolism	I

BrucellaGL002972	BMEI0502	Bme	COG4991	Uncharacterized protein with a bacterial SH3 domain homologue	Function unknown	S
BrucellaGL002973	BMEI0501	Bme	COG0556	Helicase subunit of the DNA excision repair complex	Replication, recombination and repair	L
BrucellaGL002974	BMEI0500	Bme	COG0741	Soluble lytic murein transglycosylase and related regulatory proteins (some contain LysM/invasin domains)	Cell wall/membrane/envelope biogenesis	M
BrucellaGL002978	BMEI0496	Bme	COG0491	Zn-dependent hydrolases, including glyoxylases	General function prediction only	R
BrucellaGL002981	BMEI0494	Bme	COG1846	Transcriptional regulators	Transcription	K
BrucellaGL002982	BMEI0493	Bme	COG0745	Response regulators consisting of a CheY-like receiver domain and a winged-helix DNA-binding domain	Signal transduction mechanisms	T
BrucellaGL002983	BMEI0492	Bme	COG0642	Signal transduction histidine kinase	Signal transduction mechanisms	T
BrucellaGL002985	BMEI0491	Bme	COG0073	EMAP domain	General function prediction only	R
BrucellaGL002986	BMEI0490	Bme	COG5465	Uncharacterized conserved protein	Function unknown	S
BrucellaGL002988	BMEI0489	Bme	COG2960	Uncharacterized protein conserved in bacteria	Function unknown	S
BrucellaGL002989	BMEI0488	Bme	COG0682	Prolipoprotein diacylglyceryltransferase	Cell wall/membrane/envelope biogenesis	M
BrucellaGL002990	BMEI0487	Bme	COG1565	Uncharacterized conserved protein	Function unknown	S
BrucellaGL002991	BMEI0486	Bme	COG1496	Uncharacterized conserved protein	Function unknown	S
BrucellaGL002992	BMEI0485	Bme	COG0006	Xaa-Pro aminopeptidase	Amino acid transport and metabolism	E
BrucellaGL002994	BMEI0483	Bme	COG0462	Phosphoribosylpyrophosphate synthetase	Nucleotide transport and metabolism	F
BrucellaGL002995	BMEI0482	Bme	COG0433	Predicted ATPase	General function prediction only	R
BrucellaGL002996	BMEI0481	Bme	COG1825	Ribosomal protein L25 (general stress protein Ctc)	Translation, ribosomal structure and biogenesis	J
BrucellaGL002997	BMEI0480	Bme	COG0193	Peptidyl-tRNA hydrolase	Translation, ribosomal structure and biogenesis	J
BrucellaGL002998	BMEI0479	Bme	COG0012	Predicted GTPase, probable translation factor	Translation, ribosomal structure and biogenesis	J
BrucellaGL002999	BMEI0478	Bme	COG2030	Acyl dehydratase	Lipid transport and metabolism	I
BrucellaGL003000	BMEI0477	Bme	COG2030	Acyl dehydratase	Lipid transport and metabolism	I

BrucellaGL003001	BMEI0476	Bme	COG0503	Adenine/guanine phosphoribosyltransferases and related PRPP-binding proteins	Nucleotide transport and metabolism	F
BrucellaGL003003	BMEI0475	Bme	COG2857	Cytochrome c1	Energy production and conversion	C
BrucellaGL003004	BMEI0474	Bme	COG1290	Cytochrome b subunit of the bc complex	Energy production and conversion	C
BrucellaGL003005	BMEI0473	Bme	COG0723	Rieske Fe-S protein	Energy production and conversion	C
BrucellaGL003007	BMEI0472	Bme	COG1132	ABC-type multidrug transport system, ATPase and permease components	Defense mechanisms	V
BrucellaGL003008	BMEI0471	Bme	COG1132	ABC-type multidrug transport system, ATPase and permease components	Defense mechanisms	V
BrucellaGL003009	BMEI0470	Bme	COG0219	Predicted rRNA methylase (SpoU class)	Translation, ribosomal structure and biogenesis	J
BrucellaGL003010	BMEI0469	Bme	COG5042	Purine nucleoside permease	Nucleotide transport and metabolism	F
BrucellaGL003012	BMEI0467	Bme	COG0408	Coproporphyrinogen III oxidase	Coenzyme transport and metabolism	H
BrucellaGL003013	BMEI0466	Bme	COG3153	Predicted acetyltransferase	General function prediction only	R
BrucellaGL003014	BMEI0465	Bme	COG3555	Aspartyl/asparaginyl beta-hydroxylase and related dioxygenases	Posttranslational modification, protein turnover, chaperones	O
BrucellaGL003015	BMEI0464	Bme	COG3555	Aspartyl/asparaginyl beta-hydroxylase and related dioxygenases	Posttranslational modification, protein turnover, chaperones	O
BrucellaGL003016	BMEI0463	Bme	COG0617	tRNA nucleotidyltransferase/poly(A) polymerase	Translation, ribosomal structure and biogenesis	J
BrucellaGL003017	BMEI0462	Bme	COG0494	NTP pyrophosphohydrolases including oxidative damage repair enzymes	Replication, recombination and repair	L
BrucellaGL003018	BMEI0461	Bme	COG3816	Uncharacterized protein conserved in bacteria	Function unknown	S
BrucellaGL003019	BMEI0460	Bme	COG0714	MoxR-like ATPases	General function prediction only	R
BrucellaGL003020	BMEI0459	Bme	COG1721	Uncharacterized conserved protein (some members contain a von Willebrand factor type A (vWA) domain)	General function prediction only	R
BrucellaGL003023	BMEI0456	Bme	COG3153	Predicted acetyltransferase	General function prediction only	R
BrucellaGL003024	BMEI0455	Bme	COG0435	Predicted glutathione S-transferase	Posttranslational modification, protein turnover, chaperones	O
BrucellaGL003025	BMEI0454	Bme	COG3047	Outer membrane protein W	Cell wall/membrane/envelope biogenesis	M

BrucellaGL003026	BMEI0453	Bme	COG0494	NTP pyrophosphohydrolases including oxidative damage repair enzymes	Replication, recombination and repair	L
BrucellaGL003027	BMEI0452	Bme	COG1409	Predicted phosphohydrolases	General function prediction only	R
BrucellaGL003028	BMEI0451	Bme	COG0119	Isopropylmalate/homocitrate/citramalate synthases	Amino acid transport and metabolism	E
BrucellaGL003029	BMEI0450	Bme	COG0053	Predicted Co/Zn/Cd cation transporters	Inorganic ion transport and metabolism	P
BrucellaGL003031	BMEI0449_1	Bme	COG0147	Anthranilate/para-aminobenzoate synthases component I	Amino acid transport and metabolism	E
BrucellaGL003032	BMEI0447	Bme	COG1522	Transcriptional regulators	Transcription	K
BrucellaGL003033	BMEI0446	Bme	COG1846	Transcriptional regulators	Transcription	K
BrucellaGL003034	BMEI0445	Bme	COG0477	Permeases of the major facilitator superfamily	Carbohydrate transport and metabolism	G
BrucellaGL003035	BMEI0444	Bme	COG5459	Predicted rRNA methylase	Translation, ribosomal structure and biogenesis	J
BrucellaGL003036	BMEI0443	Bme	COG5345	Uncharacterized protein conserved in bacteria	Function unknown	S
BrucellaGL003038	BMEI0441	Bme	COG2113	ABC-type proline/glycine betaine transport systems, periplasmic components	Amino acid transport and metabolism	E
BrucellaGL003039	BMEI0440	Bme	COG4176	ABC-type proline/glycine betaine transport system, permease component	Amino acid transport and metabolism	E
BrucellaGL003040	BMEI0439	Bme	COG4175	ABC-type proline/glycine betaine transport system, ATPase component	Amino acid transport and metabolism	E
BrucellaGL003041	BMEI0438	Bme	COG4608	ABC-type oligopeptide transport system, ATPase component	Amino acid transport and metabolism	E
BrucellaGL003042	BMEI0437	Bme	COG0444	ABC-type dipeptide/oligopeptide/nickel transport system, ATPase component	Amino acid transport and metabolism	E
BrucellaGL003043	BMEI0436	Bme	COG1173	ABC-type dipeptide/oligopeptide/nickel transport systems, permease components	Amino acid transport and metabolism	E
BrucellaGL003044	BMEI0435	Bme	COG0601	ABC-type dipeptide/oligopeptide/nickel transport systems, permease components	Amino acid transport and metabolism	E
BrucellaGL003045	BMEI0434	Bme	COG0747	ABC-type dipeptide transport system, periplasmic component	Amino acid transport and metabolism	E
BrucellaGL003046	BMEI0433	Bme	COG0747	ABC-type dipeptide transport system, periplasmic component	Amino acid transport and metabolism	E

BrucellaGL003047	BMEI0430	Bme	COG0640	Predicted transcriptional regulators	Transcription	K
BrucellaGL003048	BMEI0429	Bme	COG0566	rRNA methylases	Translation, ribosomal structure and biogenesis	J
BrucellaGL003049	BMEI0428	Bme	COG0482	Predicted tRNA(5-methylaminomethyl-2-thiouridylate) methyltransferase, contains the PP-loop ATPase domain	Translation, ribosomal structure and biogenesis	J
BrucellaGL003053	BMEI0425	Bme	COG5400	Uncharacterized protein conserved in bacteria	Function unknown	S
BrucellaGL003055	BMEI0424	Bme	COG5385	Uncharacterized protein conserved in bacteria	Function unknown	S
BrucellaGL003056	BMEI0423	Bme	COG0745	Response regulators consisting of a CheY-like receiver domain and a winged-helix DNA-binding domain	Signal transduction mechanisms	T
BrucellaGL003058	BMEI0421	Bme	COG0399	Predicted pyridoxal phosphate-dependent enzyme apparently involved in regulation of cell wall biogenesis	Cell wall/membrane/envelope biogenesis	M
BrucellaGL003060	BMEI0420	Bme	COG0673	Predicted dehydrogenases and related proteins	General function prediction only	R
BrucellaGL003061	BMEI0418	Bme	COG3306	Glycosyltransferase involved in LPS biosynthesis	Cell wall/membrane/envelope biogenesis	M
BrucellaGL003062	BMEI0417_1	Bme	COG2202	FOG: PAS/PAC domain	Signal transduction mechanisms	T
BrucellaGL003063	BMEI0415	Bme	COG2982	Uncharacterized protein involved in outer membrane biogenesis	Cell wall/membrane/envelope biogenesis	M
BrucellaGL003064	BMEI0414	Bme	COG1177	ABC-type spermidine/putrescine transport system, permease component II	Amino acid transport and metabolism	E
BrucellaGL003065	BMEI0413	Bme	COG1176	ABC-type spermidine/putrescine transport system, permease component I	Amino acid transport and metabolism	E
BrucellaGL003066	BMEI0412	Bme	COG3842	ABC-type spermidine/putrescine transport systems, ATPase components	Amino acid transport and metabolism	E
BrucellaGL003067	BMEI0411	Bme	COG0687	Spermidine/putrescine-binding periplasmic protein	Amino acid transport and metabolism	E
BrucellaGL003069	BMEI0410_2	Bme	COG3800	Predicted transcriptional regulator	General function prediction only	R
BrucellaGL003070	AGc1081_1	Atu	COG1396	Predicted transcriptional regulators	Transcription	K
BrucellaGL003071	BMEI0409	Bme	COG2224	Isocitrate lyase	Energy production and conversion	C
BrucellaGL003074	hdhA	Eco	COG1028	Dehydrogenases with different specificities (related to short-chain alcohol dehydrogenases)	Lipid transport and metabolism	I

BrucellaGL003075	BMEI0405	Bme	COG1028	Dehydrogenases with different specificities (related to short-chain alcohol dehydrogenases)	Lipid transport and metabolism	I
BrucellaGL003077	BMEI0403	Bme	COG2095	Multiple antibiotic transporter	Intracellular trafficking, secretion, and vesicular transport	U
BrucellaGL003078	BMEI0402	Bme	COG3637	Opacity protein and related surface antigens	Cell wall/membrane/envelope biogenesis	M
BrucellaGL003081	BMEI0399	Bme	COG2376	Dihydroxyacetone kinase	Carbohydrate transport and metabolism	G
BrucellaGL003082	BMEI0398_1	Bme	COG2390	Transcriptional regulator, contains sigma factor-related N-terminal domain	Transcription	K
BrucellaGL003083	BMEI0397	Bme	COG2376	Dihydroxyacetone kinase	Carbohydrate transport and metabolism	G
BrucellaGL003084	BMEI0396	Bme	COG2376	Dihydroxyacetone kinase	Carbohydrate transport and metabolism	G
BrucellaGL003085	BMEI0395	Bme	COG1028	Dehydrogenases with different specificities (related to short-chain alcohol dehydrogenases)	Lipid transport and metabolism	I
BrucellaGL003086	BMEI0394	Bme	COG1028	Dehydrogenases with different specificities (related to short-chain alcohol dehydrogenases)	Lipid transport and metabolism	I
BrucellaGL003087	BMEI0393	Bme	COG1879	ABC-type sugar transport system, periplasmic component	Carbohydrate transport and metabolism	G
BrucellaGL003088	BMEI0392	Bme	COG1172	Ribose/xylose/arabinose/galactoside ABC-type transport systems, permease components	Carbohydrate transport and metabolism	G
BrucellaGL003090	BMEI0390	Bme	COG5591	Uncharacterized conserved protein	Function unknown	S
BrucellaGL003091	BMEI0391	Bme	COG1129	ABC-type sugar transport system, ATPase component	Carbohydrate transport and metabolism	G
BrucellaGL003093	BMEI0388	Bme	COG2303	Choline dehydrogenase and related flavoproteins	Amino acid transport and metabolism	E
BrucellaGL003096	BMEI0200	Bme	COG3316	Transposase and inactivated derivatives	Replication, recombination and repair	L
BrucellaGL003097	BMEI0200	Bme	COG3316	Transposase and inactivated derivatives	Replication, recombination and repair	L
BrucellaGL003098	PAE3208	Pya	COG1396	Predicted transcriptional regulators	Transcription	K
BrucellaGL003099	BMEI0200	Bme	COG3316	Transposase and inactivated derivatives	Replication, recombination and repair	L
BrucellaGL003100	BMEI0201	Bme	COG0261	Ribosomal protein L21	Translation, ribosomal structure and biogenesis	J

BrucellaGL003101	SMc03772	Sme	COG0211	Ribosomal protein L27	Translation, ribosomal structure and biogenesis	J
BrucellaGL003102	BMEI0203	Bme	COG1670	Acetyltransferases, including N-acetylases of ribosomal proteins	Translation, ribosomal structure and biogenesis	J
BrucellaGL003103	mll4016	Mlo	COG1670	Acetyltransferases, including N-acetylases of ribosomal proteins	Translation, ribosomal structure and biogenesis	J
BrucellaGL003104	BMEI0204	Bme	COG0454	Histone acetyltransferase HPA2 and related acetyltransferases	Transcription	K
BrucellaGL003105	BMEI0206	Bme	COG0536	Predicted GTPase	General function prediction only	R
BrucellaGL003106	BMEI0207	Bme	COG0263	Glutamate 5-kinase	Amino acid transport and metabolism	E
BrucellaGL003107	BMEI0208	Bme	COG0014	Gamma-glutamyl phosphate reductase	Amino acid transport and metabolism	E
BrucellaGL003108	BMEI0209	Bme	COG1057	Nicotinic acid mononucleotide adenylyltransferase	Coenzyme transport and metabolism	H
BrucellaGL003109	BMEI0210	Bme	COG0799	Uncharacterized homolog of plant Iojap protein	Function unknown	S
BrucellaGL003110	BMEI0211	Bme	COG1576	Uncharacterized conserved protein	Function unknown	S
BrucellaGL003112	BMEI0213	Bme	COG4942	Membrane-bound metallopeptidase	Cell cycle control, cell division, chromosome partitioning	D
BrucellaGL003113	BMEI0214	Bme	COG0793	Periplasmic protease	Cell wall/membrane/envelope biogenesis	M
BrucellaGL003114	BMEI0215	Bme	COG0494	NTP pyrophosphohydrolases including oxidative damage repair enzymes	Replication, recombination and repair	L
BrucellaGL003115	BMEI0216	Bme	COG2261	Predicted membrane protein	Function unknown	S
BrucellaGL003116	BMEI0217	Bme	COG3619	Predicted membrane protein	Function unknown	S
BrucellaGL003117	BMEI0218	Bme	COG0583	Transcriptional regulator	Transcription	K
BrucellaGL003118	BMEI0219	Bme	COG1012	NAD-dependent aldehyde dehydrogenases	Energy production and conversion	C
BrucellaGL003121	BMEI0221	Bme	COG2240	Pyridoxal/pyridoxine/pyridoxamine kinase	Coenzyme transport and metabolism	H
BrucellaGL003122	BMEI0222	Bme	COG0288	Carbonic anhydrase	Inorganic ion transport and metabolism	P
BrucellaGL003123	BMEI0223	Bme	COG2951	Membrane-bound lytic murein transglycosylase B	Cell wall/membrane/envelope biogenesis	M
BrucellaGL003124	BMEI0225	Bme	COG0541	Signal recognition particle GTPase	Intracellular trafficking, secretion, and vesicular transport	U
BrucellaGL003125	BMEI0226	Bme	COG1605	Chorismate mutase	Amino acid transport and metabolism	E

BrucellaGL003126	BMEI0227	Bme	COG0228	Ribosomal protein S16	Translation, ribosomal structure and biogenesis	J
BrucellaGL003128	BMEI0228	Bme	COG1704	Uncharacterized conserved protein	Function unknown	S
BrucellaGL003129	BMEI0229	Bme	COG1512	Beta-propeller domains of methanol dehydrogenase type	General function prediction only	R
BrucellaGL003130	BMEI0230	Bme	COG3762	Predicted membrane protein	Function unknown	S
BrucellaGL003131	BMEI0231	Bme	COG2902	NAD-specific glutamate dehydrogenase	Amino acid transport and metabolism	E
BrucellaGL003132	BMEI0232	Bme	COG2270	Permeases of the major facilitator superfamily	General function prediction only	R
BrucellaGL003133	BMEI0233	Bme	COG0138	AICAR transformylase/IMP cyclohydrolase PurH (only IMP cyclohydrolase domain in Aful)	Nucleotide transport and metabolism	F
BrucellaGL003136	BMEI0234	Bme	COG5360	Uncharacterized protein conserved in bacteria	Function unknown	S
BrucellaGL003137	BMEI0235	Bme	COG0144	tRNA and rRNA cytosine-C5-methylases	Translation, ribosomal structure and biogenesis	J
BrucellaGL003138	BMEI0236	Bme	COG0501	Zn-dependent protease with chaperone function	Posttranslational modification, protein turnover, chaperones	O
BrucellaGL003140	BMEI0237	Bme	COG5508	Uncharacterized conserved small protein	Function unknown	S
BrucellaGL003141	BMEI0238	Bme	COG0365	Acyl-coenzyme A synthetases/AMP-(fatty) acid ligases	Lipid transport and metabolism	I
BrucellaGL003142	BMEI0239	Bme	COG5425	Usg protein, probable subunit of phosphoribosylanthranilate isomerase	Amino acid transport and metabolism	E
BrucellaGL003143	BMEI0240	Bme	COG1289	Predicted membrane protein	Function unknown	S
BrucellaGL003144	BMEI0241	Bme	COG0325	Predicted enzyme with a TIM-barrel fold	General function prediction only	R
BrucellaGL003145	BMEI0242	Bme	COG0495	Leucyl-tRNA synthetase	Translation, ribosomal structure and biogenesis	J
BrucellaGL003146	BMEI0243	Bme	COG5468	Predicted secreted (periplasmic) protein	Function unknown	S
BrucellaGL003147	BMEI0244	Bme	COG0176	Transaldolase	Carbohydrate transport and metabolism	G
BrucellaGL003148	mll4071	Mlo	COG1198	Primosomal protein N' (replication factor Y) - superfamily II helicase	Replication, recombination and repair	L
BrucellaGL003150	BMEI0248	Bme	COG0712	F0F1-type ATP synthase, delta subunit (mitochondrial oligomycin sensitivity protein)	Energy production and conversion	C
BrucellaGL003151	BMEI0249	Bme	COG0056	F0F1-type ATP synthase, alpha subunit	Energy production and conversion	C
BrucellaGL003152	BMEI0250	Bme	COG0224	F0F1-type ATP synthase, gamma subunit	Energy production and conversion	C

BrucellaGL003153	BMEI0251	Bme	COG0055	F0F1-type ATP synthase, beta subunit	Energy production and conversion	C
BrucellaGL003154	BMEI0252	Bme	COG0355	F0F1-type ATP synthase, epsilon subunit (mitochondrial delta subunit)	Energy production and conversion	C
BrucellaGL003155	BMEI0253	Bme	COG1846	Transcriptional regulators	Transcription	K
BrucellaGL003156	BMEI0254	Bme	COG1802	Transcriptional regulators	Transcription	K
BrucellaGL003157	BMEI0256	Bme	COG0665	Glycine/D-amino acid oxidases (deaminating)	Amino acid transport and metabolism	E
BrucellaGL003158	BMEI0256	Bme	COG0665	Glycine/D-amino acid oxidases (deaminating)	Amino acid transport and metabolism	E
BrucellaGL003159	BMEI0257	Bme	COG3938	Proline racemase	Amino acid transport and metabolism	E
BrucellaGL003160	BMEI0258	Bme	COG0559	Branched-chain amino acid ABC-type transport system, permease components	Amino acid transport and metabolism	E
BrucellaGL003161	BMEI0259	Bme	COG4177	ABC-type branched-chain amino acid transport system, permease component	Amino acid transport and metabolism	E
BrucellaGL003162	BMEI0260_1	Bme	COG0411	ABC-type branched-chain amino acid transport systems, ATPase component	Amino acid transport and metabolism	E
BrucellaGL003163	BMEI0261	Bme	COG0410	ABC-type branched-chain amino acid transport systems, ATPase component	Amino acid transport and metabolism	E
BrucellaGL003165	BMEI0263	Bme	COG0683	ABC-type branched-chain amino acid transport systems, periplasmic component	Amino acid transport and metabolism	E
BrucellaGL003167	BMEI0263	Bme	COG0683	ABC-type branched-chain amino acid transport systems, periplasmic component	Amino acid transport and metabolism	E
BrucellaGL003168	BMEI0266	Bme	COG1038	Pyruvate carboxylase	Energy production and conversion	C
BrucellaGL003169	BMEI0267	Bme	COG0477	Permeases of the major facilitator superfamily	Carbohydrate transport and metabolism	G
BrucellaGL003170	BMEI0268	Bme	COG1028	Dehydrogenases with different specificities (related to short-chain alcohol dehydrogenases)	Lipid transport and metabolism	I
BrucellaGL003171	BMEI0269	Bme	COG0821	Enzyme involved in the deoxyxylulose pathway of isoprenoid biosynthesis	Lipid transport and metabolism	I
BrucellaGL003172	BMEI0270	Bme	COG0142	Geranylgeranyl pyrophosphate synthase	Coenzyme transport and metabolism	H
BrucellaGL003173	BMEI0271	Bme	COG0744	Membrane carboxypeptidase (penicillin-binding protein)	Cell wall/membrane/envelope biogenesis	M
BrucellaGL003174	BMEI0272	Bme	COG0333	Ribosomal protein L32	Translation, ribosomal structure and biogenesis	J

BrucellaGL003175	BMEI0273	Bme	COG3193	Uncharacterized protein, possibly involved in utilization of glycolate and propanediol	General function prediction only	R
BrucellaGL003177	BMEI0274	Bme	COG0183	Acetyl-CoA acetyltransferase	Lipid transport and metabolism	I
BrucellaGL003178	BMEI0275	Bme	COG0513	Superfamily II DNA and RNA helicases	Replication, recombination and repair	L
BrucellaGL003179	BMEI0277	Bme	COG1188	Ribosome-associated heat shock protein implicated in the recycling of the 50S subunit (S4 paralog)	Translation, ribosomal structure and biogenesis	J
BrucellaGL003180	BMEI0278	Bme	COG1146	Ferredoxin	Energy production and conversion	C
BrucellaGL003181	BMEI0279	Bme	COG1329	Transcriptional regulators, similar to M. xanthus CarD	Transcription	K
BrucellaGL003182	BMEI0280	Bme	COG0568	DNA-directed RNA polymerase, sigma subunit (sigma70/sigma32)	Transcription	K
BrucellaGL003183	BMEI0281	Bme	COG2258	Uncharacterized protein conserved in bacteria	Function unknown	S
BrucellaGL003184	BMEI0282	Bme	COG4784	Putative Zn-dependent protease	General function prediction only	R
BrucellaGL003185	BMEI0283	Bme	COG3840	ABC-type thiamine transport system, ATPase component	Coenzyme transport and metabolism	H
BrucellaGL003186	BMEI0284	Bme	COG1178	ABC-type Fe3+ transport system, permease component	Inorganic ion transport and metabolism	P
BrucellaGL003187	BMEI0285	Bme	COG4143	ABC-type thiamine transport system, periplasmic component	Coenzyme transport and metabolism	H
BrucellaGL003188	BMEI0286	Bme	COG1564	Thiamine pyrophosphokinase	Coenzyme transport and metabolism	H
BrucellaGL003190	BMEI0288	Bme	COG0488	ATPase components of ABC transporters with duplicated ATPase domains	General function prediction only	R
BrucellaGL003191	BMEI0289	Bme	COG3750	Uncharacterized protein conserved in bacteria	Function unknown	S
BrucellaGL003192	BMEI0290	Bme	COG3931	Predicted N-formylglutamate amidohydrolase	Amino acid transport and metabolism	E
BrucellaGL003193	BMEI0291	Bme	COG5480	Predicted integral membrane protein	Function unknown	S
BrucellaGL003194	BMEI0292	Bme	COG0469	Pyruvate kinase	Carbohydrate transport and metabolism	G
BrucellaGL003195	BMEI0293	Bme	COG0457	FOG: TPR repeat	General function prediction only	R
BrucellaGL003196	BMEI0294	Bme	COG0257	Ribosomal protein L36	Translation, ribosomal structure and biogenesis	J
BrucellaGL003197	BMEI0295	Bme	COG0026	Phosphoribosylaminoimidazole carboxylase (NCAIR synthetase)	Nucleotide transport and metabolism	F

BrucellaGL003198	BMEI0296	Bme	COG0041	Phosphoribosylcarboxyaminoimidazole (NCAIR) mutase	Nucleotide transport and metabolism	F
BrucellaGL003199	BMEI0297	Bme	COG5481	Uncharacterized conserved small protein containing a coiled-coil domain	Function unknown	S
BrucellaGL003200	BMEI0299	Bme	COG5570	Uncharacterized small protein	Function unknown	S
BrucellaGL003201	BMEI0300	Bme	COG0477	Permeases of the major facilitator superfamily	Carbohydrate transport and metabolism	G
BrucellaGL003202	BMEI0301	Bme	COG5509	Uncharacterized small protein containing a coiled-coil domain	Function unknown	S
BrucellaGL003204	BMEI0302	Bme	COG0604	NADPH:quinone reductase and related Zn-dependent oxidoreductases	Energy production and conversion	C
BrucellaGL003205	BMEI0303	Bme	COG3820	Uncharacterized protein conserved in bacteria	Function unknown	S
BrucellaGL003206	BMEI0304	Bme	COG2050	Uncharacterized protein, possibly involved in aromatic compounds catabolism	Secondary metabolites biosynthesis, transport and catabolism	Q
BrucellaGL003207	BMEI0305	Bme	COG2378	Predicted transcriptional regulator	Transcription	K
BrucellaGL003209	BMEI0307	Bme	COG0365	Acyl-coenzyme A synthetases/AMP-(fatty) acid ligases	Lipid transport and metabolism	I
BrucellaGL003211	BMEI0309	Bme	COG0126	3-phosphoglycerate kinase	Carbohydrate transport and metabolism	G
BrucellaGL003212	BMEI0310	Bme	COG0057	Glyceraldehyde-3-phosphate dehydrogenase/erythrose-4-phosphate dehydrogenase	Carbohydrate transport and metabolism	G
BrucellaGL003213	BMEI0311	Bme	COG0021	Transketolase	Carbohydrate transport and metabolism	G
BrucellaGL003215	BMEI0313	Bme	COG3027	Uncharacterized protein conserved in bacteria	Function unknown	S
BrucellaGL003218	BMEI0315	Bme	COG0212	5-formyltetrahydrofolate cyclo-ligase	Coenzyme transport and metabolism	H
BrucellaGL003219	BMEI0316	Bme	COG1692	Uncharacterized protein conserved in bacteria	Function unknown	S
BrucellaGL003220	BMEI0317	Bme	COG0861	Membrane protein TerC, possibly involved in tellurium resistance	Inorganic ion transport and metabolism	P
BrucellaGL003221	BMEI0318	Bme	COG3543	Uncharacterized conserved protein	Function unknown	S
BrucellaGL003222	BMEI0319	Bme	COG1268	Uncharacterized conserved protein	General function prediction only	R
BrucellaGL003223	BMEI0320	Bme	COG1802	Transcriptional regulators	Transcription	K
BrucellaGL003225	BMEI0321	Bme	COG0217	Uncharacterized conserved protein	Function unknown	S

BrucellaGL003226	BMEI0322	Bme	COG0254	Ribosomal protein L31	Translation, ribosomal structure and biogenesis	J
BrucellaGL003227	BMEI0323	Bme	COG1132	ABC-type multidrug transport system, ATPase and permease components	Defense mechanisms	V
BrucellaGL003228	BMEI0324	Bme	COG1360	Flagellar motor protein	Cell motility	N
BrucellaGL003230	BMEI0326	Bme	COG0483	Archaeal fructose-1,6-bisphosphatase and related enzymes of inositol monophosphatase family	Carbohydrate transport and metabolism	G
BrucellaGL003231	BMEI0327	Bme	COG0231	Translation elongation factor P (EF-P)/translation initiation factor 5A (eIF-5A)	Translation, ribosomal structure and biogenesis	J
BrucellaGL003232	BMEI0328	Bme	COG0790	FOG: TPR repeat, SEL1 subfamily	General function prediction only	R
BrucellaGL003233	BMEI0329	Bme	COG0352	Thiamine monophosphate synthase	Coenzyme transport and metabolism	H
BrucellaGL003234	BMEI0330	Bme	COG4645	Uncharacterized protein conserved in bacteria	Function unknown	S
BrucellaGL003236	BMEI0331	Bme	COG5317	Uncharacterized protein conserved in bacteria	Function unknown	S
BrucellaGL003237	BMEI0332	Bme	COG0817	Holliday junction resolvasome, endonuclease subunit	Replication, recombination and repair	L
BrucellaGL003238	BMEI0333	Bme	COG0632	Holliday junction resolvasome, DNA-binding subunit	Replication, recombination and repair	L
BrucellaGL003239	BMEI0334	Bme	COG2255	Holliday junction resolvasome, helicase subunit	Replication, recombination and repair	L
BrucellaGL003240	BMEI0335	Bme	COG0824	Predicted thioesterase	General function prediction only	R
BrucellaGL003241	BMEI0336	Bme	COG0811	Biopolymer transport proteins	Intracellular trafficking, secretion, and vesicular transport	U
BrucellaGL003242	BMEI0337	Bme	COG0848	Biopolymer transport protein	Intracellular trafficking, secretion, and vesicular transport	U
BrucellaGL003244	BMEI0339	Bme	COG0823	Periplasmic component of the Tol biopolymer transport system	Intracellular trafficking, secretion, and vesicular transport	U
BrucellaGL003245	BMEI0340	Bme	COG2885	Outer membrane protein and related peptidoglycan-associated (lipo)proteins	Cell wall/membrane/envelope biogenesis	M
BrucellaGL003247	BMEI0341	Bme	COG1729	Uncharacterized protein conserved in bacteria	Function unknown	S
BrucellaGL003248	BMEI0342	Bme	COG0037	Predicted ATPase of the PP-loop superfamily implicated in cell cycle control	Cell cycle control, cell division, chromosome partitioning	D
BrucellaGL003249	BMEI0343	Bme	COG0465	ATP-dependent Zn proteases	Posttranslational modification, protein turnover, chaperones	O

BrucellaGL003250	BMEI0344	Bme	COG1109	Phosphomannomutase	Carbohydrate transport and metabolism	G
BrucellaGL003252	BMEI0345	Bme	COG3637	Opacity protein and related surface antigens	Cell wall/membrane/envelope biogenesis	M
BrucellaGL003253	BMEI0346	Bme	COG0385	Predicted Na+-dependent transporter	General function prediction only	R
BrucellaGL003254	BMEI0347	Bme	COG1932	Phosphoserine aminotransferase	Coenzyme transport and metabolism	H
BrucellaGL003256	BMEI0349	Bme	COG0111	Phosphoglycerate dehydrogenase and related dehydrogenases	Coenzyme transport and metabolism	H
BrucellaGL003258	BMEI0351	Bme	COG0104	Adenylosuccinate synthase	Nucleotide transport and metabolism	F
BrucellaGL003260	BMEI0353	Bme	COG0477	Permeases of the major facilitator superfamily	Carbohydrate transport and metabolism	G
BrucellaGL003262	BMEI0354	Bme	COG1633	Uncharacterized conserved protein	Function unknown	S
BrucellaGL003263	BMEI0355	Bme	COG0121	Predicted glutamine amidotransferase	General function prediction only	R
BrucellaGL003265	BMEI0357	Bme	COG1522	Transcriptional regulators	Transcription	K
BrucellaGL003266	BMEI0358	Bme	COG0756	dUTPase	Nucleotide transport and metabolism	F
BrucellaGL003267	BMEI0359	Bme	COG0845	Membrane-fusion protein	Cell wall/membrane/envelope biogenesis	M
BrucellaGL003268	BMEI0360_1	Bme	COG1136	ABC-type antimicrobial peptide transport system, ATPase component	Defense mechanisms	V
BrucellaGL003269	BMEI0360_2	Bme	COG0577	ABC-type antimicrobial peptide transport system, permease component	Defense mechanisms	V
BrucellaGL003270	BMEI0361	Bme	COG0577	ABC-type antimicrobial peptide transport system, permease component	Defense mechanisms	V
BrucellaGL003271	BMEI0362	Bme	COG3698	Predicted periplasmic protein	Function unknown	S
BrucellaGL003272	BMEI0363	Bme	COG0810	Periplasmic protein TonB, links inner and outer membranes	Cell wall/membrane/envelope biogenesis	M
BrucellaGL003273	BMEI0364	Bme	COG0848	Biopolymer transport protein	Intracellular trafficking, secretion, and vesicular transport	U
BrucellaGL003274	BMEI0365	Bme	COG0811	Biopolymer transport proteins	Intracellular trafficking, secretion, and vesicular transport	U
BrucellaGL003281	BMEI0370	Bme	COG3920	Signal transduction histidine kinase	Signal transduction mechanisms	T
BrucellaGL003283	BMEI0371	Bme	COG1595	DNA-directed RNA polymerase specialized sigma subunit, sigma24 homolog	Transcription	K

BrucellaGL003285	BMEI0372_1	Bme	COG1595	DNA-directed RNA polymerase specialized sigma subunit, sigma24 homolog	Transcription	K
BrucellaGL003286	msr3702	Mlo	COG5487	Small integral membrane protein	Function unknown	S
BrucellaGL003288	BMEI0374	Bme	COG3920	Signal transduction histidine kinase	Signal transduction mechanisms	T
BrucellaGL003289	BMEI0375	Bme	COG0735	Fe2+/Zn2+ uptake regulation proteins	Inorganic ion transport and metabolism	P
BrucellaGL003291	BMEI0377	Bme	COG0564	Pseudouridylate synthases, 23S RNA-specific	Translation, ribosomal structure and biogenesis	J
BrucellaGL003292	BMEI0378	Bme	COG0568	DNA-directed RNA polymerase, sigma subunit (sigma70/sigma32)	Transcription	K
BrucellaGL003293	BMEI0379	Bme	COG1247	Sortase and related acyltransferases	Cell wall/membrane/envelope biogenesis	M
BrucellaGL003294	BMEI0380	Bme	COG2225	Malate synthase	Energy production and conversion	C
BrucellaGL003295	BMEI0381	Bme	COG0008	Glutamyl- and glutaminyl-tRNA synthetases	Translation, ribosomal structure and biogenesis	J
BrucellaGL003296	BMEI0382	Bme	COG0122	3-methyladenine DNA glycosylase/8-oxoguanine DNA glycosylase	Replication, recombination and repair	L
BrucellaGL003297	BMEI0383	Bme	COG1403	Restriction endonuclease	Defense mechanisms	V
BrucellaGL003298	BMEI0384	Bme	COG1495	Disulfide bond formation protein DsbB	Posttranslational modification, protein turnover, chaperones	O
BrucellaGL003299	BMEI0385	Bme	COG1238	Predicted membrane protein	Function unknown	S
BrucellaGL003300	BMEI0386	Bme	COG1012	NAD-dependent aldehyde dehydrogenases	Energy production and conversion	C
BrucellaGL003302	BMEI0387	Bme	COG1414	Transcriptional regulator	Transcription	K
BrucellaGL003304	BMEI0901	Bme	COG1961	Site-specific recombinases, DNA invertase Pin homologs	Replication, recombination and repair	L
