# Supplementary material for: Comparative Genomic Analysis of Brucella melitensis Vaccine Strain M5 Provides Insights into Virulence Attenuation
Source: PLoS One. 2013 Aug 14;8(8):e70852. doi: 10.1371/journal.pone.0070852 (PMC3743847; doi:10.1371/journal.pone.0070852)
Supplement: Table S3 — SNPs detected in the B. melitensis vaccine strain M5 compared with the virulent strain 16 M. (PDF) [file pone.0070852.s004.pdf]

Table S3: SNPs of *B. melitensis* vaccine strain M5 and wild strain 16M

16M Chromosom	Position	SNP Type	Base on 16M	Base on M5	Residue on 16M	Residue on M5	Gene on 16M	COG Annotation
AE008917	179	intergenic	C	G	-	-	-	-
AE008917	1279	intergenic	A	C	-	-	-	-
AE008917	4622	syn	G	A	L	L	BMEI0005	Highly conserved protein containing a thioredoxin domain
AE008917	7088	nsyn	C	T	S	L	BMEI0007	NAD/FAD-utilizing enzyme apparently involved in cell division
AE008917	7275	syn	C	A	I	I	BMEI0007	NAD/FAD-utilizing enzyme apparently involved in cell division
AE008917	7405	nsyn	T	G	S	A	BMEI0007	NAD/FAD-utilizing enzyme apparently involved in cell division
AE008917	7623	syn	C	T	P	P	BMEI0007	NAD/FAD-utilizing enzyme apparently involved in cell division
AE008917	7948	nsyn	A	G	N	D	BMEI0007	NAD/FAD-utilizing enzyme apparently involved in cell division
AE008917	8208	syn	A	G	T	T	BMEI0007	NAD/FAD-utilizing enzyme apparently involved in cell division
AE008917	11352	nsyn	A	G	H	R	BMEI0010	Predicted transcriptional regulators
AE008917	15668	nsyn	A	C	L	R	BMEI0015	ABC-type uncharacterized transport system, periplasmic component
AE008917	16453	syn	C	T	I	I	BMEI0017	Coenzyme F420-dependent N5,N10-methylene tetrahydromethanopterin reductase and related flavin-dependent oxidoreductases
AE008917	18471	nsyn	T	A	H	L	BMEI0018	NA
AE008917	19997	nsyn	G	C	G	R	BMEI0020	Predicted dehydrogenases and related proteins
AE008917	20504	nsyn	G	A	G	S	BMEI0020	Predicted dehydrogenases and related proteins
AE008917	24681	nsyn	C	G	A	G	BMEI0025	Choline dehydrogenase and related flavoproteins
AE008917	24775	syn	T	A	G	G	BMEI0025	Choline dehydrogenase and related flavoproteins
AE008917	25761	nsyn	A	C	E	A	BMEI0025	Choline dehydrogenase and related flavoproteins
AE008917	25973	nsyn	T	G	S	A	BMEI0025	Choline dehydrogenase and related flavoproteins
AE008917	26245	nsyn	A	G	K	E	BMEI0026	Dehydrogenases with different specificities (related to short-chain alcohol dehydrogenases)
AE008917	27160	nsyn	A	C	N	T	BMEI0028	Choline dehydrogenase and related flavoproteins
AE008917	27386	syn	C	T	T	T	BMEI0028	Choline dehydrogenase and related flavoproteins
AE008917	28875	intergenic	A	G	-	-	-	-
AE008917	30764	syn	T	C	Q	Q	BMEI0033	Metal-dependent amidase/aminoacylase/carboxypeptidase
AE008917	30972	nsyn	G	A	A	V	BMEI0033	Metal-dependent amidase/aminoacylase/carboxypeptidase
AE008917	31088	syn	G	A	H	H	BMEI0033	Metal-dependent amidase/aminoacylase/carboxypeptidase
AE008917	33842	syn	C	T	H	H	BMEI0036	Putative GTPases (G3E family)
AE008917	33906	nsyn	C	T	R	C	BMEI0036	Putative GTPases (G3E family)

AE008917	35577	nsyn	G	T	P	T	BMEI0038	Uncharacterized protein conserved in bacteria
AE008917	35700	nsyn	A	C	C	G	BMEI0038	Uncharacterized protein conserved in bacteria
AE008917	35781	intergenic	C	T	-	-	-	-
AE008917	37878	syn	A	G	Y	Y	BMEI0040	Site-specific recombinase XerD
AE008917	37969	nsyn	A	C	*	E	BMEI0041	NA
AE008917	37978	nsyn	A	G	S	P	BMEI0041	NA
AE008917	38489	syn	A	T	T	T	BMEI0042	Shikimate kinase
AE008917	39370	syn	T	C	I	I	BMEI0043	3-dehydroquinate synthetase
AE008917	41545	nsyn	G	A	P	S	BMEI0045	Stress-induced morphogen (activity unknown)
AE008917	43949	intergenic	C	G	-	-	-	-
AE008917	46202	nsyn	A	G	*	Q	BMEI0051	NA
AE008917	47067	syn	C	T	E	E	BMEI0051	NA
AE008917	49703	nsyn	C	T	E	K	BMEI0053	Cation transport ATPase
AE008917	55576	nsyn	G	A	Q	*	BMEI0058	Type V secretory pathway, adhesin AidA
AE008917	55869	nsyn	G	A	T	I	BMEI0058	Type V secretory pathway, adhesin AidA
AE008917	58081	intergenic	T	C	-	-	-	-
AE008917	58413	intergenic	T	C	-	-	-	-
AE008917	58573	intergenic	T	C	-	-	-	-
AE008917	59287	nsyn	A	C	K	T	BMEI0060	NA
AE008917	60495	nsyn	T	C	F	S	BMEI0061	NA
AE008917	61931	nsyn	A	C	R	S	BMEI0062	NA
AE008917	61980	nsyn	A	G	S	G	BMEI0062	NA
AE008917	62160	nsyn	G	T	D	Y	BMEI0062	NA
AE008917	62516	intergenic	A	G	-	-	-	-
AE008917	62624	intergenic	C	T	-	-	-	-
AE008917	64807	intergenic	C	T	-	-	-	-
AE008917	66488	nsyn	G	C	D	E	BMEI0068	Exonuclease III
AE008917	66491	syn	G	C	G	G	BMEI0068	Exonuclease III
AE008917	66494	syn	G	C	G	G	BMEI0068	Exonuclease III
AE008917	68745	intergenic	T	C	-	-	-	-
AE008917	70522	nsyn	G	A	G	S	BMEI0073	Cell division protein
AE008917	72981	syn	C	T	Y	Y	BMEI0075	1-acyl-sn-glycerol-3-phosphate acyltransferase
AE008917	79314	syn	G	C	P	P	BMEI0081	SAM-dependent methyltransferases
AE008917	80531	nsyn	T	C	Q	R	BMEI0083	NA
AE008917	80748	nsyn	A	C	C	G	BMEI0083	NA

AE008917	83050	nsyn	C	A	G	V	BMEI0084	Diaminopimelate decarboxylase
AE008917	83491	nsyn	G	T	P	D	BMEI0084	Diaminopimelate decarboxylase
AE008917	83492	nsyn	G	C	P	D	BMEI0084	Diaminopimelate decarboxylase
AE008917	86566	nsyn	A	G	S	G	BMEI0087	Thiol-disulfide isomerase and thioredoxins
AE008917	87866	nsyn	G	A	A	V	BMEI0089	Predicted membrane protein
AE008917	89187	intergenic	C	G	-	-	-	-
AE008917	89776	syn	A	G	S	S	BMEI0092	Uncharacterized conserved protein
AE008917	90650	nsyn	T	C	N	S	BMEI0093	Organic radical activating enzymes
AE008917	90664	nsyn	C	G	W	C	BMEI0093	Organic radical activating enzymes
AE008917	92982	intergenic	G	C	-	-	-	-
AE008917	94474	intergenic	G	C	-	-	-	-
AE008917	94488	intergenic	T	G	-	-	-	-
AE008917	94489	intergenic	C	G	-	-	-	-
AE008917	94748	nsyn	G	C	E	Q	BMEI0099	3-hydroxyacyl-CoA dehydrogenase
AE008917	95420	intergenic	C	G	-	-	-	-
AE008917	95421	intergenic	T	G	-	-	-	-
AE008917	95424	intergenic	T	G	-	-	-	-
AE008917	95425	intergenic	T	G	-	-	-	-
AE008917	95426	intergenic	C	G	-	-	-	-
AE008917	95429	intergenic	A	G	-	-	-	-
AE008917	95432	intergenic	A	G	-	-	-	-
AE008917	95433	intergenic	A	C	-	-	-	-
AE008917	95436	intergenic	A	G	-	-	-	-
AE008917	98448	syn	C	G	V	V	BMEI0103	Cystathionine beta-lyases/cystathionine gamma-synthases
AE008917	98571	syn	A	G	P	P	BMEI0103	Cystathionine beta-lyases/cystathionine gamma-synthases
AE008917	98645	nsyn	G	A	R	H	BMEI0103	Cystathionine beta-lyases/cystathionine gamma-synthases
AE008917	99386	intergenic	G	A	-	-	-	-
AE008917	101499	nsyn	C	T	A	V	BMEI0105	L-asparaginase/archaeal Glu-tRNAGln amidotransferase subunit D
AE008917	101863	syn	T	C	Y	Y	BMEI0105	L-asparaginase/archaeal Glu-tRNAGln amidotransferase subunit D
AE008917	102017	nsyn	A	G	M	V	BMEI0105	L-asparaginase/archaeal Glu-tRNAGln amidotransferase subunit D
AE008917	104931	syn	G	A	I	I	BMEI0109	Aspartate ammonia-lyase
AE008917	105042	syn	C	G	G	G	BMEI0109	Aspartate ammonia-lyase
AE008917	105310	nsyn	A	T	V	E	BMEI0109	Aspartate ammonia-lyase
AE008917	105421	nsyn	T	C	D	G	BMEI0109	Aspartate ammonia-lyase
AE008917	105866	nsyn	T	C	N	D	BMEI0109	Aspartate ammonia-lyase

AE008917	106348	nsyn	G	C	L	V	BMEI0110	Arginase/agmatinase/formimionoglutamate hydrolase, arginase family
AE008917	106788	nsyn	A	G	V	A	BMEI0110	Arginase/agmatinase/formimionoglutamate hydrolase, arginase family
AE008917	107298	nsyn	T	C	T	A	BMEI0111	ABC-type polar amino acid transport system, ATPase component
AE008917	107584	syn	G	A	A	A	BMEI0111	ABC-type polar amino acid transport system, ATPase component
AE008917	110172	nsyn	C	T	G	D	BMEI0115	ABC-type amino acid transport/signal transduction systems, periplasmic component/domain
AE008917	110523	intergenic	G	A	-	-	-	-
AE008917	110837	nsyn	T	C	L	P	BMEI0116	Transcriptional regulator
AE008917	112139	intergenic	T	C	-	-	-	-
AE008917	112565	intergenic	A	C	-	-	-	-
AE008917	112589	intergenic	G	T	-	-	-	-
AE008917	116261	intergenic	C	T	-	-	-	-
AE008917	116819	nsyn	C	T	A	T	BMEI0121	Preprotein translocase subunit SecA (ATPase, RNA helicase)
AE008917	119728	nsyn	C	G	N	K	BMEI0123	Parvulin-like peptidyl-prolyl isomerase
AE008917	120936	intergenic	T	A	-	-	-	-
AE008917	122835	nsyn	T	G	D	E	BMEI0125	Histone acetyltransferase HPA2 and related acetyltransferases
AE008917	125444	intergenic	T	C	-	-	-	-
AE008917	125848	syn	A	C	L	L	BMEI0130	Intracellular septation protein A
AE008917	125862	nsyn	C	G	A	P	BMEI0130	Intracellular septation protein A
AE008917	125932	syn	A	C	L	L	BMEI0130	Intracellular septation protein A
AE008917	126102	intergenic	C	T	-	-	-	-
AE008917	126844	nsyn	T	C	H	R	BMEI0131	Signal recognition particle GTPase
AE008917	128761	nsyn	T	C	K	R	BMEI0132	2-methylthioadenine synthetase
AE008917	129297	nsyn	T	C	D	G	BMEI0133	Diaminopimelate epimerase
AE008917	129365	syn	A	G	R	R	BMEI0133	Diaminopimelate epimerase
AE008917	129850	syn	A	G	Q	Q	BMEI0134	NA
AE008917	130041	intergenic	T	C	-	-	-	-
AE008917	130677	intergenic	G	A	-	-	-	-
AE008917	131475	nsyn	C	T	R	C	BMEI0136	Predicted ATPase
AE008917	132590	syn	G	C	G	G	BMEI0137	Malate/lactate dehydrogenases
AE008917	132933	nsyn	T	C	S	P	BMEI0137	Malate/lactate dehydrogenases
AE008917	140814	nsyn	A	T	N	Y	BMEI0143	Putative threonine efflux protein
AE008917	143104	nsyn	A	G	N	D	BMEI0145	Pyruvate/2-oxoglutarate dehydrogenase complex, dihydrolipoamide dehydrogenase (E3) component, and related enzymes

AE008917	143168	nsyn	G	A	G	D	BMEI0145	Pyruvate/2-oxoglutarate dehydrogenase complex, dihydrolipoamide dehydrogenase (E3) component, and related enzymes
AE008917	143754	nsyn	C	A	Q	H	BMEI0146	Uncharacterized protein conserved in bacteria
AE008917	144918	nsyn	A	G	L	P	BMEI0147	Integrase
AE008917	145626	intergenic	T	C	-	-	-	-
AE008917	145749	intergenic	T	A	-	-	-	-
AE008917	150336	syn	C	A	L	L	BMEI0155	Cytosine/uracil/thiamine/allantoin permeases
AE008917	154772	nsyn	T	C	S	P	BMEI0160	Succinate dehydrogenase, hydrophobic anchor subunit
AE008917	155812	nsyn	G	C	G	A	BMEI0161	Succinate dehydrogenase/fumarate reductase, flavoprotein subunit
AE008917	161169	intergenic	G	A	-	-	-	-
AE008917	161232	nsyn	A	G	M	V	BMEI0167	Ammonia permease
AE008917	161873	syn	T	C	T	T	BMEI0167	Ammonia permease
AE008917	163667	nsyn	C	T	H	Y	BMEI0168	DNA segregation ATPase FtsK/SpoIIIE and related proteins
AE008917	163749	nsyn	C	A	A	E	BMEI0168	DNA segregation ATPase FtsK/SpoIIIE and related proteins
AE008917	167651	nsyn	T	G	V	G	BMEI0170	Permeases of the drug/metabolite transporter (DMT) superfamily
AE008917	172559	syn	T	C	G	G	BMEI0177	Uroporphyrinogen-III synthase
AE008917	174033	syn	A	C	L	L	BMEI0178	Uncharacterized protein conserved in bacteria
AE008917	175344	syn	C	A	S	S	BMEI0179	Uncharacterized membrane-bound protein
AE008917	176191	intergenic	C	A	-	-	-	-
AE008917	177263	nsyn	T	G	I	L	BMEI0181	Permeases of the major facilitator superfamily
AE008917	179078	nsyn	T	C	K	E	BMEI0182	SAM-dependent methyltransferases
AE008917	185279	intergenic	A	G	-	-	-	-
AE008917	186348	syn	G	A	E	E	BMEI0190	Signal transduction protein containing GAF and PtsI domains
AE008917	188086	syn	A	G	L	L	BMEI0191	Protein chain release factor A
AE008917	188464	syn	T	C	R	R	BMEI0191	Protein chain release factor A
AE008917	189338	nsyn	A	C	K	T	BMEI0192	Methylase of polypeptide chain release factors
AE008917	193110	syn	C	T	G	G	BMEI0195	ATPases with chaperone activity, ATP-binding subunit
AE008917	194011	intergenic	T	A	-	-	-	-
AE008917	194012	intergenic	C	T	-	-	-	-
AE008917	194014	intergenic	C	T	-	-	-	-
AE008917	194037	intergenic	A	G	-	-	-	-
AE008917	194045	intergenic	G	T	-	-	-	-
AE008917	194072	intergenic	A	G	-	-	-	-
AE008917	194124	intergenic	T	C	-	-	-	-
AE008917	195499	syn	T	C	V	V	BMEI0196	Uncharacterized protein conserved in bacteria

AE008917	207294	intergenic	C	T	-	-	-	-
AE008917	210364	syn	A	G	E	E	BMEI0204	Histone acetyltransferase HPA2 and related acetyltransferases
AE008917	210723	nsyn	G	A	G	E	BMEI0204	Histone acetyltransferase HPA2 and related acetyltransferases
AE008917	214413	nsyn	A	G	N	S	BMEI0208	Gamma-glutamyl phosphate reductase
AE008917	218687	nsyn	T	C	F	L	BMEI0213	Membrane-bound metallopeptidase
AE008917	220733	syn	C	T	A	A	BMEI0214	Periplasmic protease
AE008917	222322	syn	T	A	T	T	BMEI0217	Predicted membrane protein
AE008917	226006	intergenic	G	A	-	-	-	-
AE008917	226676	nsyn	C	T	A	V	BMEI0221	Pyridoxal/pyridoxine/pyridoxamine kinase
AE008917	233670	nsyn	G	A	A	T	BMEI0230	Predicted membrane protein
AE008917	236219	syn	C	A	P	P	BMEI0231	NAD-specific glutamate dehydrogenase
AE008917	238008	nsyn	G	T	V	F	BMEI0231	NAD-specific glutamate dehydrogenase
AE008917	239021	syn	T	C	S	S	BMEI0231	NAD-specific glutamate dehydrogenase
AE008917	241864	syn	T	C	A	A	BMEI0233	AICAR transformylase/IMP cyclohydrolase PurH (only IMP cyclohydrolase domain in Aful)
AE008917	245149	nsyn	T	C	S	G	BMEI0235	tRNA and rRNA cytosine-C5-methylases
AE008917	247249	intergenic	T	C	-	-	-	-
AE008917	247934	nsyn	C	A	G	C	BMEI0238	Acyl-coenzyme A synthetases/AMP-(fatty) acid ligases
AE008917	250250	nsyn	T	G	Y	D	BMEI0240	Predicted membrane protein
AE008917	251042	syn	C	T	A	A	BMEI0241	Predicted enzyme with a TIM-barrel fold
AE008917	252640	syn	T	C	L	L	BMEI0242	Leucyl-tRNA synthetase
AE008917	252840	syn	A	C	G	G	BMEI0242	Leucyl-tRNA synthetase
AE008917	254137	syn	T	C	T	T	BMEI0243	Predicted secreted (periplasmic) protein
AE008917	254753	nsyn	A	C	Y	D	BMEI0244	Transaldolase
AE008917	256131	syn	C	T	A	A	BMEI0245	Primosomal protein N' (replication factor Y) - superfamily II helicase
AE008917	257308	syn	G	A	Q	Q	BMEI0246	Primosomal protein N' (replication factor Y) - superfamily II helicase
AE008917	264420	nsyn	C	T	T	M	BMEI0253	Transcriptional regulators
AE008917	266739	nsyn	T	C	L	P	BMEI0256	Glycine/D-amino acid oxidases (deaminating)
AE008917	267539	nsyn	C	T	A	V	BMEI0257	Proline racemase
AE008917	267758	nsyn	C	T	A	V	BMEI0257	Proline racemase
AE008917	267774	intergenic	G	T	-	-	-	-
AE008917	268492	nsyn	G	T	G	V	BMEI0258	Branched-chain amino acid ABC-type transport system, permease components
AE008917	268952	nsyn	C	A	D	E	BMEI0258	Branched-chain amino acid ABC-type transport system, permease components

AE008917	270103	syn	C	T	V	V	BMEI0259	ABC-type branched-chain amino acid transport system, permease component
AE008917	270226	syn	G	C	G	G	BMEI0259	ABC-type branched-chain amino acid transport system, permease component
AE008917	272031	nsyn	G	A	D	N	BMEI0261	ABC-type branched-chain amino acid transport systems , ATPase component
AE008917	272490	nsyn	C	A	L	I	BMEI0262	NA
AE008917	274290	intergenic	G	A	-	-	-	-
AE008917	274905	nsyn	T	C	S	P	BMEI0264	ABC-type branched-chain amino acid transport systems, periplasmic component
AE008917	275135	nsyn	G	C	*	Y	BMEI0264	ABC-type branched-chain amino acid transport systems, periplasmic component
AE008917	276192	syn	T	C	S	S	BMEI0266	Pyruvate carboxylase
AE008917	276213	syn	C	T	D	D	BMEI0266	Pyruvate carboxylase
AE008917	276243	syn	C	T	H	H	BMEI0266	Pyruvate carboxylase
AE008917	276942	syn	C	T	T	T	BMEI0266	Pyruvate carboxylase
AE008917	277415	nsyn	T	A	L	Q	BMEI0266	Pyruvate carboxylase
AE008917	278903	nsyn	A	G	D	G	BMEI0266	Pyruvate carboxylase
AE008917	279277	nsyn	G	A	G	S	BMEI0266	Pyruvate carboxylase
AE008917	281201	nsyn	C	A	R	L	BMEI0268	Dehydrogenases with different specificities (related to short-chain alcohol dehydrogenases)
AE008917	283579	syn	A	C	G	G	BMEI0270	Geranylgeranyl pyrophosphate synthase
AE008917	284377	nsyn	C	T	P	S	BMEI0271	Membrane carboxypeptidase (penicillin-binding protein)
AE008917	284487	syn	T	C	G	G	BMEI0271	Membrane carboxypeptidase (penicillin-binding protein)
AE008917	284615	nsyn	G	A	R	K	BMEI0271	Membrane carboxypeptidase (penicillin-binding protein)
AE008917	285808	intergenic	G	A	-	-	-	-
AE008917	286261	syn	G	A	Q	Q	BMEI0274	Acetyl-CoA acetyltransferase
AE008917	286981	syn	G	C	G	G	BMEI0274	Acetyl-CoA acetyltransferase
AE008917	286990	syn	C	T	A	A	BMEI0274	Acetyl-CoA acetyltransferase
AE008917	287013	nsyn	G	C	C	S	BMEI0274	Acetyl-CoA acetyltransferase
AE008917	287229	nsyn	C	G	P	R	BMEI0274	Acetyl-CoA acetyltransferase
AE008917	287703	syn	C	T	R	R	BMEI0275	Superfamily II DNA and RNA helicases
AE008917	287892	syn	T	G	R	R	BMEI0275	Superfamily II DNA and RNA helicases
AE008917	289002	syn	T	C	N	N	BMEI0275	Superfamily II DNA and RNA helicases
AE008917	289173	syn	A	T	R	R	BMEI0275	Superfamily II DNA and RNA helicases
AE008917	289207	nsyn	C	T	L	F	BMEI0275	Superfamily II DNA and RNA helicases

AE008917	289344	syn	C	T	V	V	BMEI0275	Superfamily II DNA and RNA helicases
AE008917	289346	nsyn	A	C	D	A	BMEI0275	Superfamily II DNA and RNA helicases
AE008917	289914	syn	T	C	I	I	BMEI0275	Superfamily II DNA and RNA helicases
AE008917	291063	intergenic	G	A	-	-	-	-
AE008917	293256	nsyn	A	T	S	C	BMEI0280	DNA-directed RNA polymerase, sigma subunit (sigma70/sigma32)
AE008917	293802	nsyn	T	C	S	P	BMEI0280	DNA-directed RNA polymerase, sigma subunit (sigma70/sigma32)
AE008917	294036	nsyn	C	G	Q	E	BMEI0280	DNA-directed RNA polymerase, sigma subunit (sigma70/sigma32)
AE008917	294532	nsyn	T	C	H	R	BMEI0281	Uncharacterized protein conserved in bacteria
AE008917	295207	nsyn	G	T	L	I	BMEI0282	Putative Zn-dependent protease
AE008917	296169	nsyn	G	A	A	V	BMEI0282	Putative Zn-dependent protease
AE008917	296491	intergenic	G	T	-	-	-	-
AE008917	298244	nsyn	C	G	E	Q	BMEI0284	ABC-type Fe3+ transport system, permease component
AE008917	301413	nsyn	G	A	D	N	BMEI0287	NA
AE008917	302210	nsyn	A	C	L	F	BMEI0288	ATPase components of ABC transporters with duplicated ATPase domains
AE008917	302389	nsyn	A	C	E	A	BMEI0288	ATPase components of ABC transporters with duplicated ATPase domains
AE008917	302972	syn	C	T	S	S	BMEI0288	ATPase components of ABC transporters with duplicated ATPase domains
AE008917	303802	intergenic	T	C	-	-	-	-
AE008917	305847	syn	G	A	K	K	BMEI0292	Pyruvate kinase
AE008917	306473	nsyn	C	T	S	L	BMEI0292	Pyruvate kinase
AE008917	306709	nsyn	A	G	I	V	BMEI0292	Pyruvate kinase
AE008917	307313	nsyn	A	G	C	R	BMEI0293	FOG: TPR repeat
AE008917	308877	nsyn	A	G	I	T	BMEI0295	Phosphoribosylaminoimidazole carboxylase (NCAIR synthetase)
AE008917	310997	syn	G	T	A	A	BMEI0300	Permeases of the major facilitator superfamily
AE008917	311486	syn	T	G	S	S	BMEI0300	Permeases of the major facilitator superfamily
AE008917	311765	syn	G	T	S	S	BMEI0300	Permeases of the major facilitator superfamily
AE008917	313326	nsyn	G	A	A	T	BMEI0302	NADPH:quinone reductase and related Zn-dependent oxidoreductases
AE008917	313373	syn	C	T	S	S	BMEI0302	NADPH:quinone reductase and related Zn-dependent oxidoreductases
AE008917	317791	nsyn	T	C	E	G	BMEI0307	Acyl-coenzyme A synthetases/AMP-(fatty) acid ligases
AE008917	319036	intergenic	C	T	-	-	-	-
AE008917	319207	nsyn	C	T	D	N	BMEI0309	3-phosphoglycerate kinase
AE008917	319582	nsyn	T	C	I	V	BMEI0309	3-phosphoglycerate kinase

AE008917	321731	nsyn	G	T	F	L	BMEI0311	Transketolase
AE008917	321875	syn	A	G	G	G	BMEI0311	Transketolase
AE008917	324882	nsyn	C	T	A	V	BMEI0314	NA
AE008917	325313	nsyn	C	T	A	V	BMEI0315	5-formyltetrahydrofolate cyclo-ligase
AE008917	327158	nsyn	G	T	G	V	BMEI0317	Membrane protein TerC, possibly involved in tellurium resistance
AE008917	327506	nsyn	C	A	T	K	BMEI0317	Membrane protein TerC, possibly involved in tellurium resistance
AE008917	328013	syn	T	C	A	A	BMEI0319	Uncharacterized conserved protein
AE008917	329341	syn	G	T	I	I	BMEI0320	Transcriptional regulators
AE008917	330937	intergenic	A	G	-	-	-	-
AE008917	332968	syn	T	C	*	*	BMEI0324	Flagellar motor protein
AE008917	335057	intergenic	T	C	-	-	-	-
AE008917	335225	intergenic	T	C	-	-	-	-
AE008917	338492	nsyn	C	G	M	I	BMEI0329	Thiamine monophosphate synthase
AE008917	339834	nsyn	G	A	V	M	BMEI0330	Uncharacterized protein conserved in bacteria
AE008917	343838	nsyn	A	G	H	R	BMEI0335	Predicted thioesterase
AE008917	344303	intergenic	C	T	-	-	-	-
AE008917	345411	nsyn	G	T	M	I	BMEI0337	Biopolymer transport protein
AE008917	347023	nsyn	C	A	T	K	BMEI0339	Periplasmic component of the Tol biopolymer transport system
AE008917	348389	intergenic	T	C	-	-	-	-
AE008917	350297	nsyn	A	G	T	A	BMEI0341	Uncharacterized protein conserved in bacteria
AE008917	355172	syn	C	T	P	P	BMEI0344	Phosphomannomutase
AE008917	356938	intergenic	T	G	-	-	-	-
AE008917	357966	nsyn	A	C	E	D	BMEI0346	Predicted Na+-dependent transporter
AE008917	358634	nsyn	T	C	V	A	BMEI0347	Phosphoserine aminotransferase
AE008917	358890	syn	C	A	I	I	BMEI0347	Phosphoserine aminotransferase
AE008917	359359	nsyn	C	T	S	L	BMEI0348	NA
AE008917	361494	nsyn	G	A	G	S	BMEI0350	NA
AE008917	362659	syn	T	G	A	A	BMEI0351	Adenylosuccinate synthase
AE008917	363091	syn	A	G	Q	Q	BMEI0351	Adenylosuccinate synthase
AE008917	363883	intergenic	C	A	-	-	-	-
AE008917	366498	nsyn	G	A	Q	*	BMEI0353	Permeases of the major facilitator superfamily
AE008917	367739	syn	A	G	V	V	BMEI0355	Predicted glutamine amidotransferase
AE008917	368190	nsyn	A	T	V	E	BMEI0355	Predicted glutamine amidotransferase
AE008917	368861	intergenic	G	T	-	-	-	-
AE008917	370320	intergenic	C	A	-	-	-	-

AE008917	372166	nsyn	C	T	Q	*	BMEI0360	ABC-type antimicrobial peptide transport system, ATPase component
AE008917	374560	nsyn	G	A	T	I	BMEI0363	Periplasmic protein TonB, links inner and outer membranes
AE008917	375094	intergenic	G	A	-	-	-	-
AE008917	375573	nsyn	A	G	V	A	BMEI0364	Biopolymer transport protein
AE008917	376222	syn	G	C	A	A	BMEI0365	Biopolymer transport proteins
AE008917	376643	intergenic	T	C	-	-	-	-
AE008917	378750	nsyn	C	T	L	F	BMEI0368	NA
AE008917	379142	nsyn	C	T	L	F	BMEI0369	NA
AE008917	380902	intergenic	A	G	-	-	-	-
AE008917	382985	nsyn	T	G	F	V	BMEI0373	Small integral membrane protein
AE008917	383582	nsyn	G	C	V	L	BMEI0374	Signal transduction histidine kinase
AE008917	384843	syn	G	A	I	I	BMEI0375	Fe2+/Zn2+ uptake regulation proteins
AE008917	385776	intergenic	A	C	-	-	-	-
AE008917	388355	nsyn	T	C	S	G	BMEI0379	Sortase and related acyltransferases
AE008917	388763	intergenic	T	C	-	-	-	-
AE008917	388786	intergenic	G	C	-	-	-	-
AE008917	388793	intergenic	A	T	-	-	-	-
AE008917	388798	intergenic	T	C	-	-	-	-
AE008917	388802	intergenic	G	C	-	-	-	-
AE008917	389166	nsyn	A	T	L	H	BMEI0380	Malate synthase
AE008917	391399	nsyn	A	C	L	R	BMEI0381	Glutamyl- and glutaminyl-tRNA synthetases
AE008917	393072	intergenic	T	C	-	-	-	-
AE008917	394207	nsyn	C	T	M	I	BMEI0384	Disulfide bond formation protein DsbB
AE008917	395148	nsyn	C	A	G	*	BMEI0386	NAD-dependent aldehyde dehydrogenases
AE008917	396015	nsyn	T	C	I	V	BMEI0386	NAD-dependent aldehyde dehydrogenases
AE008917	396167	nsyn	A	G	I	T	BMEI0386	NAD-dependent aldehyde dehydrogenases
AE008917	396174	nsyn	T	G	T	P	BMEI0386	NAD-dependent aldehyde dehydrogenases
AE008917	396732	nsyn	G	T	M	I	BMEI0387	Transcriptional regulator
AE008917	396833	nsyn	G	T	R	M	BMEI0387	Transcriptional regulator
AE008917	405914	nsyn	A	G	D	G	BMEI0389	NA
AE008917	406837	nsyn	G	T	S	I	BMEI0391	ABC-type sugar transport system, ATPase component
AE008917	406843	nsyn	A	C	N	T	BMEI0391	ABC-type sugar transport system, ATPase component
AE008917	406910	syn	C	T	G	G	BMEI0391	ABC-type sugar transport system, ATPase component
AE008917	411318	nsyn	C	G	A	G	BMEI0395	Dehydrogenases with different specificities (related to short-chain alcohol dehydrogenases)

AE008917	412449	syn	T	C	S	S	BMEI0396	Dihydroxyacetone kinase
AE008917	412625	nsyn	G	T	E	D	BMEI0397	Dihydroxyacetone kinase
AE008917	414370	nsyn	A	T	K	N	BMEI0398	Transcriptional regulator, contains sigma factor-related N-terminal domain
AE008917	415541	nsyn	G	T	R	L	BMEI0399	Dihydroxyacetone kinase
AE008917	418688	nsyn	G	A	S	N	BMEI0401	NA
AE008917	418801	intergenic	G	T	-	-	-	-
AE008917	418803	intergenic	C	T	-	-	-	-
AE008917	420149	nsyn	T	C	Q	R	BMEI0403	Multiple antibiotic transporter
AE008917	420289	syn	A	T	G	G	BMEI0403	Multiple antibiotic transporter
AE008917	420975	nsyn	A	G	S	P	BMEI0404	NA
AE008917	421397	nsyn	G	A	S	F	BMEI0404	NA
AE008917	422242	syn	A	G	P	P	BMEI0405	Dehydrogenases with different specificities (related to short-chain alcohol dehydrogenases)
AE008917	423118	nsyn	G	T	T	K	BMEI0406	Dehydrogenases with different specificities (related to short-chain alcohol dehydrogenases)
AE008917	423129	nsyn	C	T	M	I	BMEI0406	Dehydrogenases with different specificities (related to short-chain alcohol dehydrogenases)
AE008917	424367	nsyn	G	C	G	R	BMEI0407	NA
AE008917	424387	syn	C	A	P	P	BMEI0407	NA
AE008917	424659	intergenic	T	C	-	-	-	-
AE008917	424710	intergenic	G	A	-	-	-	-
AE008917	424742	syn	C	G	P	P	BMEI0408	NA
AE008917	424813	nsyn	A	C	F	V	BMEI0408	NA
AE008917	431524	syn	A	G	V	V	BMEI0413	ABC-type spermidine/putrescine transport system, permease component I
AE008917	432088	nsyn	C	T	A	V	BMEI0414	ABC-type spermidine/putrescine transport system, permease component II
AE008917	432341	syn	C	A	I	I	BMEI0414	ABC-type spermidine/putrescine transport system, permease component II
AE008917	433543	syn	T	C	R	R	BMEI0415	Uncharacterized protein involved in outer membrane biogenesis
AE008917	434738	intergenic	T	G	-	-	-	-
AE008917	436791	syn	G	A	F	F	BMEI0417	FOG: PAS/PAC domain
AE008917	436881	syn	C	T	T	T	BMEI0417	FOG: PAS/PAC domain
AE008917	437160	syn	T	A	R	R	BMEI0417	FOG: PAS/PAC domain
AE008917	438852	intergenic	A	G	-	-	-	-

AE008917	439625	syn	A	G	L	L	BMEI0418	Glycosyltransferase involved in LPS biosynthesis
AE008917	441667	nsyn	A	G	N	S	BMEI0421	Predicted pyridoxal phosphate-dependent enzyme apparently I nvolved in regulation of cell wall biogenesis
AE008917	448659	syn	C	A	V	V	BMEI0428	Predicted tRNA(5-methylaminomethyl-2-thiouridylate) methyltransferase, contains the PP-loop ATPase domain
AE008917	449629	nsyn	T	C	C	R	BMEI0428	Predicted tRNA(5-methylaminomethyl-2-thiouridylate) methyltransferase, contains the PP-loop ATPase domain
AE008917	452138	syn	A	G	R	R	BMEI0433	ABC-type dipeptide transport system, periplasmic component
AE008917	453375	nsyn	A	G	K	E	BMEI0434	ABC-type dipeptide transport system, periplasmic component
AE008917	453780	intergenic	A	C	-	-	-	-
AE008917	453819	intergenic	G	A	-	-	-	-
AE008917	455123	nsyn	A	T	L	F	BMEI0436	ABC-type dipeptide/oligopeptide/nickel transport systems, permease components
AE008917	458362	syn	G	A	A	A	BMEI0439	ABC-type proline/glycine betaine transport system, ATPase component
AE008917	460362	nsyn	T	G	E	D	BMEI0441	ABC-type proline/glycine betaine transport systems, periplasmic components
AE008917	460724	nsyn	G	C	L	V	BMEI0441	ABC-type proline/glycine betaine transport systems, periplasmic components
AE008917	461238	syn	G	C	L	L	BMEI0442	NA
AE008917	462954	nsyn	C	T	A	V	BMEI0444	Predicted rRNA methylase
AE008917	465195	intergenic	A	C	-	-	-	-
AE008917	465731	nsyn	G	A	P	S	BMEI0447	Transcriptional regulators
AE008917	466112	intergenic	C	T	-	-	-	-
AE008917	471374	nsyn	G	T	G	V	BMEI0451	Isopropylmalate/homocitrate/citramalate synthases
AE008917	473598	syn	C	T	L	L	BMEI0453	NTP pyrophosphohydrolases including oxidative damage repair enzymes
AE008917	475259	nsyn	C	A	R	L	BMEI0455	Predicted glutathione S-transferase
AE008917	477801	syn	G	A	A	A	BMEI0457	NA
AE008917	479641	nsyn	T	C	H	R	BMEI0458	NA
AE008917	484613	nsyn	A	G	T	A	BMEI0462	NTP pyrophosphohydrolases including oxidative damage repair enzymes
AE008917	486611	nsyn	A	G	K	E	BMEI0464	Aspartyl/asparaginyl beta-hydroxylase and related dioxygenases
AE008917	488071	syn	A	G	F	F	BMEI0467	Coproporphyrinogen III oxidase
AE008917	488983	intergenic	T	G	-	-	-	-
AE008917	489005	syn	C	A	A	A	BMEI0468	NA

AE008917	489017	syn	T	G	P	P	BMEI0468	NA
AE008917	489024	nsyn	C	G	Q	E	BMEI0468	NA
AE008917	489029	syn	T	A	G	G	BMEI0468	NA
AE008917	489033	nsyn	A	C	S	R	BMEI0468	NA
AE008917	489047	nsyn	C	G	D	E	BMEI0468	NA
AE008917	490287	syn	A	G	T	T	BMEI0469	Purine nucleoside permease
AE008917	491327	nsyn	T	C	L	P	BMEI0471	ABC-type multidrug transport system, ATPase and permease components
AE008917	491748	syn	C	T	R	R	BMEI0471	ABC-type multidrug transport system, ATPase and permease components
AE008917	492135	syn	T	C	I	I	BMEI0471	ABC-type multidrug transport system, ATPase and permease components
AE008917	499012	nsyn	A	C	N	K	BMEI0477	Acyl dehydratase
AE008917	499063	nsyn	C	T	M	I	BMEI0477	Acyl dehydratase
AE008917	501736	syn	C	T	E	E	BMEI0480	Peptidyl-tRNA hydrolase
AE008917	506225	intergenic	A	G	-	-	-	-
AE008917	506284	nsyn	G	C	S	W	BMEI0484	NA
AE008917	506671	nsyn	T	G	N	T	BMEI0484	NA
AE008917	506706	syn	C	T	K	K	BMEI0484	NA
AE008917	507550	nsyn	C	T	V	I	BMEI0485	Xaa-Pro aminopeptidase
AE008917	508924	nsyn	G	T	A	D	BMEI0486	Uncharacterized conserved protein
AE008917	515291	syn	C	T	E	E	BMEI0493	Response regulators consisting of a CheY-like receiver domain and a winged-helix DNA-binding domain
AE008917	516343	intergenic	G	A	-	-	-	-
AE008917	517106	intergenic	T	C	-	-	-	-
AE008917	517841	nsyn	C	A	T	K	BMEI0496	Zn-dependent hydrolases, including glyoxylases
AE008917	518504	nsyn	C	A	K	N	BMEI0497	NA
AE008917	518589	intergenic	C	T	-	-	-	-
AE008917	520690	nsyn	T	C	K	E	BMEI0501	Helicase subunit of the DNA excision repair complex
AE008917	520776	nsyn	G	C	P	R	BMEI0501	Helicase subunit of the DNA excision repair complex
AE008917	521312	syn	T	G	V	V	BMEI0501	Helicase subunit of the DNA excision repair complex
AE008917	525399	nsyn	G	T	A	E	BMEI0504	Uncharacterized protein conserved in bacteria
AE008917	525749	intergenic	T	G	-	-	-	-
AE008917	527460	intergenic	T	G	-	-	-	-
AE008917	530313	syn	A	G	E	E	BMEI0509	Glycosyltransferase
AE008917	530788	syn	G	A	D	D	BMEI0510	Transcriptional regulators

AE008917	532722	intergenic	G	A	-	-	-	-
AE008917	536026	syn	T	C	P	P	BMEI0516	Aspartate/tyrosine/aromatic aminotransferase
AE008917	539486	nsyn	C	G	F	L	BMEI0520	ABC-type uncharacterized transport system, permease and ATPase components
AE008917	539720	syn	C	T	D	D	BMEI0520	ABC-type uncharacterized transport system, permease and ATPase components
AE008917	540732	nsyn	G	T	S	G	BMEI0521	NA
AE008917	540733	nsyn	G	C	S	G	BMEI0521	NA
AE008917	540734	nsyn	A	C	S	G	BMEI0521	NA
AE008917	540786	intergenic	T	G	-	-	-	-
AE008917	540977	intergenic	C	A	-	-	-	-
AE008917	542578	nsyn	C	A	D	Y	BMEI0522	Carbamoylphosphate synthase large subunit (split gene in MJ)
AE008917	545020	nsyn	T	G	*	Y	BMEI0523	Response regulator of the LytR/AlgR family
AE008917	546729	syn	T	C	G	G	BMEI0525	Predicted metalloprotease
AE008917	548163	nsyn	C	T	A	T	BMEI0526	Carbamoylphosphate synthase small subunit
AE008917	549148	syn	A	G	L	L	BMEI0527	Uncharacterized conserved protein
AE008917	549680	nsyn	T	C	I	M	BMEI0528	Na+/phosphate symporter
AE008917	550252	nsyn	A	G	Y	H	BMEI0528	Na+/phosphate symporter
AE008917	550549	nsyn	A	C	*	E	BMEI0529	Na+/phosphate symporter
AE008917	550646	syn	A	G	A	A	BMEI0529	Na+/phosphate symporter
AE008917	551104	syn	T	G	R	R	BMEI0529	Na+/phosphate symporter
AE008917	554534	nsyn	A	G	I	V	BMEI0532	DNA-directed RNA polymerase, sigma subunit (sigma70/sigma32)
AE008917	556811	syn	C	T	D	D	BMEI0534	Uncharacterized conserved protein
AE008917	557424	nsyn	C	G	P	A	BMEI0535	NA
AE008917	559007	nsyn	C	G	L	F	BMEI0537	Integrase
AE008917	564634	intergenic	T	A	-	-	-	-
AE008917	565144	syn	C	T	G	G	BMEI0545	Uncharacterized protein conserved in bacteria
AE008917	565913	nsyn	A	C	M	L	BMEI0545	Uncharacterized protein conserved in bacteria
AE008917	566622	nsyn	T	G	I	L	BMEI0546	Amidases related to nicotinamidase
AE008917	566634	nsyn	T	C	R	G	BMEI0546	Amidases related to nicotinamidase
AE008917	566805	nsyn	G	T	D	Y	BMEI0547	Uncharacterized Zn-ribbon-containing protein involved in phosphonate metabolism
AE008917	567250	nsyn	T	C	T	A	BMEI0548	Flavodoxin reductases (ferredoxin-NADPH reductases) family 1
AE008917	567619	nsyn	G	A	P	S	BMEI0548	Flavodoxin reductases (ferredoxin-NADPH reductases) family 1
AE008917	568280	syn	C	T	Q	Q	BMEI0549	Phenylpropionate dioxygenase and related ring-hydroxylating dioxygenases, large terminal subunit

AE008917	568291	nsyn	T	G	T	P	BMEI0549	Phenylpropionate dioxygenase and related ring-hydroxylating dioxygenases, large terminal subunit
AE008917	568583	syn	C	G	R	R	BMEI0549	Phenylpropionate dioxygenase and related ring-hydroxylating dioxygenases, large terminal subunit
AE008917	568846	nsyn	A	G	S	P	BMEI0549	Phenylpropionate dioxygenase and related ring-hydroxylating dioxygenases, large terminal subunit
AE008917	568888	nsyn	T	C	N	D	BMEI0549	Phenylpropionate dioxygenase and related ring-hydroxylating dioxygenases, large terminal subunit
AE008917	571955	nsyn	A	G	V	A	BMEI0552	Lysophospholipase
AE008917	572922	nsyn	G	C	K	N	BMEI0553	ATPase components of ABC transporters with duplicated ATPase domains
AE008917	573372	syn	T	C	R	R	BMEI0553	ATPase components of ABC transporters with duplicated ATPase domains
AE008917	574222	nsyn	C	T	L	F	BMEI0553	ATPase components of ABC transporters with duplicated ATPase domains
AE008917	575211	intergenic	G	A	-	-	-	-
AE008917	575272	intergenic	C	T	-	-	-	-
AE008917	578502	intergenic	A	C	-	-	-	-
AE008917	581978	syn	G	T	P	P	BMEI0561	Membrane-bound lytic murein transglycosylase B
AE008917	582436	nsyn	T	C	E	G	BMEI0562	Lyzozyme M1 (1,4-beta-N-acetylmuramidase)
AE008917	582445	nsyn	A	C	V	G	BMEI0562	Lyzozyme M1 (1,4-beta-N-acetylmuramidase)
AE008917	583399	nsyn	C	G	A	G	BMEI0563	NA
AE008917	584723	syn	C	A	A	A	BMEI0565	Negative regulator of beta-lactamase expression
AE008917	585524	intergenic	T	C	-	-	-	-
AE008917	590319	nsyn	T	C	V	A	BMEI0573	Cell division protein FtsI/penicillin-binding protein 2
AE008917	592138	intergenic	T	C	-	-	-	-
AE008917	592167	nsyn	T	G	V	G	BMEI0574	UDP-N-acetylmuramyl tripeptide synthase
AE008917	592174	syn	C	T	G	G	BMEI0574	UDP-N-acetylmuramyl tripeptide synthase
AE008917	592178	nsyn	G	A	A	T	BMEI0574	UDP-N-acetylmuramyl tripeptide synthase
AE008917	593219	nsyn	G	A	V	I	BMEI0574	UDP-N-acetylmuramyl tripeptide synthase
AE008917	596683	nsyn	T	C	L	P	BMEI0577	UDP-N-acetylmuramoylalanine-D-glutamate ligase
AE008917	597745	syn	C	G	A	A	BMEI0578	Bacterial cell division membrane protein
AE008917	602316	syn	C	T	G	G	BMEI0581	UDP-N-acetylmuramate dehydrogenase
AE008917	603916	syn	C	T	V	V	BMEI0583	Cell division septal protein
AE008917	605388	syn	T	C	V	V	BMEI0584	Actin-like ATPase involved in cell division
AE008917	605591	nsyn	C	T	T	M	BMEI0584	Actin-like ATPase involved in cell division

AE008917	606063	syn	G	A	L	L	BMEI0585	Cell division GTPase
AE008917	607177	nsyn	T	C	S	P	BMEI0585	Cell division GTPase
AE008917	607625	nsyn	G	C	G	A	BMEI0585	Cell division GTPase
AE008917	607626	nsyn	C	G	G	A	BMEI0585	Cell division GTPase
AE008917	607657	intergenic	G	C	-	-	-	-
AE008917	607805	intergenic	T	C	-	-	-	-
AE008917	609212	intergenic	A	G	-	-	-	-
AE008917	611478	nsyn	G	A	D	N	BMEI0588	ATPase involved in DNA repair
AE008917	612513	nsyn	T	C	I	T	BMEI0589	NAD-dependent DNA ligase (contains BRCT domain type II)
AE008917	612876	nsyn	A	C	E	A	BMEI0589	NAD-dependent DNA ligase (contains BRCT domain type II)
AE008917	613985	nsyn	A	G	K	E	BMEI0589	NAD-dependent DNA ligase (contains BRCT domain type II)
AE008917	616658	nsyn	G	T	T	N	BMEI0591	Xaa-Pro aminopeptidase
AE008917	619257	nsyn	C	T	L	F	BMEI0594	Acetyltransferase (isoleucine patch superfamily)
AE008917	619406	nsyn	G	A	R	H	BMEI0595	Uncharacterized protein conserved in bacteria
AE008917	619427	nsyn	C	T	A	V	BMEI0595	Uncharacterized protein conserved in bacteria
AE008917	619845	syn	C	T	L	L	BMEI0595	Uncharacterized protein conserved in bacteria
AE008917	623961	nsyn	G	A	G	D	BMEI0598	Uncharacterized conserved protein
AE008917	626080	nsyn	A	G	I	V	BMEI0600	NA
AE008917	627258	syn	A	G	L	L	BMEI0600	NA
AE008917	627530	nsyn	G	A	R	H	BMEI0600	NA
AE008917	627948	syn	G	A	V	V	BMEI0600	NA
AE008917	628448	nsyn	T	C	V	A	BMEI0600	NA
AE008917	629505	syn	T	C	F	F	BMEI0600	NA
AE008917	630938	syn	T	C	S	S	BMEI0603	Uncharacterized protein conserved in bacteria
AE008917	631508	nsyn	G	A	S	L	BMEI0604	Transcriptional regulator
AE008917	631998	intergenic	C	T	-	-	-	-
AE008917	632134	intergenic	A	C	-	-	-	-
AE008917	639198	nsyn	G	A	V	M	BMEI0613	Trypsin-like serine proteases, typically periplasmic, contain C-terminal PDZ domain
AE008917	639224	syn	T	C	L	L	BMEI0613	Trypsin-like serine proteases, typically periplasmic, contain C-terminal PDZ domain
AE008917	639377	nsyn	A	C	R	S	BMEI0613	Trypsin-like serine proteases, typically periplasmic, contain C-terminal PDZ domain
AE008917	640569	nsyn	T	C	C	R	BMEI0614	Predicted flavoproteins
AE008917	640577	syn	C	T	V	V	BMEI0614	Predicted flavoproteins
AE008917	640908	nsyn	G	T	E	*	BMEI0614	Predicted flavoproteins

AE008917	641439	nsyn	C	G	V	L	BMEI0615	Phosphoserine phosphatase
AE008917	642787	nsyn	T	A	V	E	BMEI0616	tRNA delta(2)-isopentenylpyrophosphate transferase
AE008917	644021	syn	T	C	V	V	BMEI0617	Thiamine pyrophosphate-requiring enzymes [acetolactate synthase, pyruvate dehydrogenase (cytochrome), glyoxylate carboligase, phosphonopyruvate decarboxylase]
AE008917	646672	nsyn	C	T	A	V	BMEI0619	ATP-dependent exoDNAse (exonuclease V), alpha subunit - helicase superfamily I member
AE008917	651889	syn	T	C	V	V	BMEI0624	Ketol-acid reductoisomerase
AE008917	654448	intergenic	T	G	-	-	-	-
AE008917	654451	intergenic	G	C	-	-	-	-
AE008917	654454	intergenic	C	G	-	-	-	-
AE008917	654783	nsyn	G	A	P	L	BMEI0627	NA
AE008917	655656	nsyn	A	G	T	A	BMEI0628	NA
AE008917	657762	syn	C	T	A	A	BMEI0630	Predicted epimerase, PhzC/PhzF homolog
AE008917	658761	syn	T	C	V	V	BMEI0632	NA
AE008917	659010	syn	A	G	G	G	BMEI0632	NA
AE008917	659067	syn	G	A	I	I	BMEI0632	NA
AE008917	661577	nsyn	A	G	*	R	BMEI0636	ABC-type cobalt transport system, ATPase component
AE008917	663772	nsyn	G	C	F	S	BMEI0640	ABC-type Co2+ transport system, permease component
AE008917	663773	nsyn	A	G	F	S	BMEI0640	ABC-type Co2+ transport system, permease component
AE008917	664027	nsyn	C	T	E	K	BMEI0641	ABC-type Co2+ transport system, periplasmic component
AE008917	665971	syn	T	C	P	P	BMEI0643	Urease accessory protein UreH
AE008917	666897	nsyn	A	C	D	E	BMEI0644	Ni2+-binding GTPase involved in regulation of expression and maturation of urease and hydrogenase
AE008917	668014	nsyn	T	G	H	P	BMEI0645	Urease accessory protein UreF
AE008917	668111	nsyn	T	C	D	G	BMEI0646	Urease accessory protein UreE
AE008917	669551	nsyn	C	T	V	M	BMEI0647	Urea amidohydrolase (urease) alpha subunit
AE008917	670725	syn	A	G	F	F	BMEI0648	Urea amidohydrolase (urease) beta subunit
AE008917	671661	nsyn	C	A	E	*	BMEI0650	NA
AE008917	671746	syn	A	C	R	R	BMEI0650	NA
AE008917	672002	intergenic	T	C	-	-	-	-
AE008917	672507	syn	G	C	A	A	BMEI0651	Uncharacterized protein conserved in bacteria
AE008917	675399	nsyn	T	C	C	R	BMEI0654	ABC-type multidrug transport system, ATPase component
AE008917	676290	syn	C	T	L	L	BMEI0654	ABC-type multidrug transport system, ATPase component
AE008917	679658	syn	T	C	G	G	BMEI0657	Outer membrane cobalamin receptor protein
AE008917	680337	nsyn	T	C	W	R	BMEI0657	Outer membrane cobalamin receptor protein

AE008917	680810	syn	A	G	A	A	BMEI0657	Outer membrane cobalamin receptor protein
AE008917	680981	syn	A	G	E	E	BMEI0657	Outer membrane cobalamin receptor protein
AE008917	682900	syn	C	T	H	H	BMEI0659	ABC-type Fe3+-siderophore transport system, permease component
AE008917	685154	intergenic	T	G	-	-	-	-
AE008917	687394	nsyn	A	C	E	A	BMEI0664	Ribose/xylose/arabinose/galactoside ABC-type transport systems, permease components
AE008917	687673	nsyn	A	G	H	R	BMEI0664	Ribose/xylose/arabinose/galactoside ABC-type transport systems, permease components
AE008917	688003	nsyn	G	A	D	N	BMEI0665	ABC-type sugar transport system, ATPase component
AE008917	688371	syn	C	T	G	G	BMEI0665	ABC-type sugar transport system, ATPase component
AE008917	688703	nsyn	G	T	K	N	BMEI0666	Dehydrogenases with different specificities (related to short-chain alcohol dehydrogenases)
AE008917	690016	syn	A	G	H	H	BMEI0668	NA
AE008917	690281	nsyn	A	T	V	D	BMEI0668	NA
AE008917	692092	intergenic	G	A	-	-	-	-
AE008917	692700	nsyn	C	T	R	C	BMEI0671	Hemolysins and related proteins containing CBS domains
AE008917	693434	syn	T	C	L	L	BMEI0671	Hemolysins and related proteins containing CBS domains
AE008917	693539	syn	T	C	V	V	BMEI0671	Hemolysins and related proteins containing CBS domains
AE008917	695628	nsyn	G	A	E	K	BMEI0673	ABC-type sulfate transport system, periplasmic component
AE008917	698293	intergenic	A	C	-	-	-	-
AE008917	700641	nsyn	T	G	T	P	BMEI0679	Kef-type K+ transport system, predicted NAD-binding component
AE008917	701906	nsyn	T	C	I	V	BMEI0680	Preprotein translocase subunit SecD
AE008917	702815	nsyn	C	T	V	I	BMEI0680	Preprotein translocase subunit SecD
AE008917	703907	intergenic	G	T	-	-	-	-
AE008917	703969	nsyn	G	A	A	V	BMEI0681	NA
AE008917	705394	nsyn	A	C	D	A	BMEI0682	Small-conductance mechanosensitive channel
AE008917	706430	syn	C	T	L	L	BMEI0682	Small-conductance mechanosensitive channel
AE008917	710028	intergenic	T	C	-	-	-	-
AE008917	710298	intergenic	T	G	-	-	-	-
AE008917	710566	nsyn	G	C	S	C	BMEI0685	Transcriptional regulator containing an amidase domain and an AraC-type DNA-binding HTH domain
AE008917	710578	nsyn	G	A	A	V	BMEI0685	Transcriptional regulator containing an amidase domain and an AraC-type DNA-binding HTH domain
AE008917	712147	nsyn	T	C	C	R	BMEI0686	Transcriptional regulator

AE008917	714199	nsyn	C	T	D	N	BMEI0688	3-hydroxyisobutyrate dehydrogenase and related beta-hydroxyacid dehydrogenases
AE008917	714311	syn	C	T	E	E	BMEI0688	3-hydroxyisobutyrate dehydrogenase and related beta-hydroxyacid dehydrogenases
AE008917	715289	nsyn	C	T	E	K	BMEI0689	Acyl-CoA dehydrogenases
AE008917	716928	syn	T	G	P	P	BMEI0690	Cobyric acid synthase
AE008917	717943	intergenic	G	A	-	-	-	-
AE008917	718559	syn	A	G	S	S	BMEI0692	Predicted integral membrane protein
AE008917	719339	nsyn	C	G	R	G	BMEI0694	Putative GTPases (G3E family)
AE008917	720225	nsyn	A	C	D	A	BMEI0694	Putative GTPases (G3E family)
AE008917	720741	syn	A	G	P	P	BMEI0695	Cobalamin biosynthesis protein CobN and related Mg-chelatases
AE008917	722665	nsyn	A	G	K	E	BMEI0695	Cobalamin biosynthesis protein CobN and related Mg-chelatases
AE008917	725220	syn	A	G	F	F	BMEI0698	Predicted permeases
AE008917	725590	intergenic	A	C	-	-	-	-
AE008917	727848	nsyn	T	C	R	G	BMEI0702	Precorrin-6x reductase
AE008917	728615	syn	G	A	I	I	BMEI0703	Cobalamin biosynthesis protein CbiD
AE008917	729020	syn	G	A	P	P	BMEI0703	Cobalamin biosynthesis protein CbiD
AE008917	729136	nsyn	G	T	P	T	BMEI0703	Cobalamin biosynthesis protein CbiD
AE008917	729781	syn	T	C	A	A	BMEI0704	Uroporphyrinogen-III methylase
AE008917	730882	nsyn	C	T	L	F	BMEI0705	Cobyrinic acid a,c-diamide synthase
AE008917	731840	syn	T	C	G	G	BMEI0706	Histidinol-phosphate/aromatic aminotransferase and cobyric acid decarboxylase
AE008917	733445	syn	T	C	L	L	BMEI0707	Cobalamin biosynthesis protein CobD/CbiB
AE008917	736028	syn	T	C	I	I	BMEI0710	NA
AE008917	736091	syn	A	G	G	G	BMEI0710	NA
AE008917	741159	nsyn	A	G	F	L	BMEI0715	Sulfite reductase, beta subunit (hemoprotein)
AE008917	744313	nsyn	C	T	A	T	BMEI0718	DNA repair proteins
AE008917	746936	intergenic	G	A	-	-	-	-
AE008917	747222	intergenic	A	G	-	-	-	-
AE008917	747573	nsyn	T	G	N	H	BMEI0722	Uncharacterized protein conserved in bacteria
AE008917	748616	intergenic	A	G	-	-	-	-
AE008917	752037	nsyn	C	G	D	E	BMEI0725	Homoserine dehydrogenase
AE008917	752426	intergenic	C	G	-	-	-	-
AE008917	752745	nsyn	G	C	A	P	BMEI0726	Fructose-1,6-bisphosphatase/sedoheptulose 1,7-bisphosphatase and related proteins

AE008917	753083	syn	A	G	V	V	BMEI0726	Fructose-1,6-bisphosphatase/sedoheptulose 1,7-bisphosphatase and related proteins
AE008917	753960	nsyn	C	T	P	L	BMEI0727	D-alanine-D-alanine ligase and related ATP-grasp enzymes
AE008917	754749	nsyn	T	C	F	S	BMEI0727	D-alanine-D-alanine ligase and related ATP-grasp enzymes
AE008917	763610	nsyn	T	C	N	D	BMEI0737	Predicted periplasmic protein
AE008917	764372	nsyn	C	T	G	R	BMEI0738	Uncharacterized protein conserved in bacteria
AE008917	764394	syn	T	C	V	V	BMEI0738	Uncharacterized protein conserved in bacteria
AE008917	766266	nsyn	T	C	C	R	BMEI0740	FOG: CBS domain
AE008917	766749	syn	A	G	G	G	BMEI0741	rRNA methylases
AE008917	766951	nsyn	T	C	H	R	BMEI0741	rRNA methylases
AE008917	769839	intergenic	G	A	-	-	-	-
AE008917	771360	nsyn	A	G	H	R	BMEI0746	Ribosomal protein L1
AE008917	773255	syn	C	T	L	L	BMEI0748	Ribosomal protein L7/L12
AE008917	775455	nsyn	C	T	A	V	BMEI0749	DNA-directed RNA polymerase, beta subunit/140 kD subunit
AE008917	776523	nsyn	C	T	A	V	BMEI0749	DNA-directed RNA polymerase, beta subunit/140 kD subunit
AE008917	777496	syn	G	A	L	L	BMEI0749	DNA-directed RNA polymerase, beta subunit/140 kD subunit
AE008917	781391	syn	A	G	P	P	BMEI0750	DNA-directed RNA polymerase, beta' subunit/160 kD subunit
AE008917	781929	nsyn	A	G	T	A	BMEI0750	DNA-directed RNA polymerase, beta' subunit/160 kD subunit
AE008917	782037	nsyn	A	G	N	D	BMEI0750	DNA-directed RNA polymerase, beta' subunit/160 kD subunit
AE008917	782886	intergenic	C	T	-	-	-	-
AE008917	783547	nsyn	C	A	T	K	BMEI0753	Ribosomal protein S7
AE008917	784884	nsyn	A	G	T	A	BMEI0754	Translation elongation factors (GTPases)
AE008917	785607	nsyn	G	A	A	T	BMEI0754	Translation elongation factors (GTPases)
AE008917	787355	syn	G	A	V	V	BMEI0756	Ribosomal protein S10
AE008917	789701	nsyn	T	A	M	K	BMEI0760	Ribosomal protein L2
AE008917	790062	syn	C	T	F	F	BMEI0760	Ribosomal protein L2
AE008917	794358	nsyn	T	C	V	A	BMEI0771	Ribosomal protein S8
AE008917	794678	nsyn	G	A	R	K	BMEI0772	Ribosomal protein L6P/L9E
AE008917	796849	intergenic	C	G	-	-	-	-
AE008917	798723	nsyn	C	G	A	G	BMEI0778	Adenylate kinase and related kinases
AE008917	799982	syn	C	T	G	G	BMEI0780	Ribosomal protein S11
AE008917	800394	syn	C	T	V	V	BMEI0781	DNA-directed RNA polymerase, alpha subunit/40 kD subunit
AE008917	801066	syn	G	T	T	T	BMEI0781	DNA-directed RNA polymerase, alpha subunit/40 kD subunit
AE008917	801853	intergenic	G	A	-	-	-	-
AE008917	802171	nsyn	A	C	Q	P	BMEI0783	Trypsin-like serine proteases, typically periplasmic, contain C-terminal PDZ domain

AE008917	802174	nsyn	A	G	D	G	BMEI0783	Trypsin-like serine proteases, typically periplasmic, contain C-terminal PDZ domain
AE008917	802175	nsyn	T	A	D	G	BMEI0783	Trypsin-like serine proteases, typically periplasmic, contain C-terminal PDZ domain
AE008917	802184	syn	A	C	A	A	BMEI0783	Trypsin-like serine proteases, typically periplasmic, contain C-terminal PDZ domain
AE008917	802188	nsyn	G	C	A	P	BMEI0783	Trypsin-like serine proteases, typically periplasmic, contain C-terminal PDZ domain
AE008917	804386	nsyn	A	G	I	V	BMEI0784	ATPase related to the helicase subunit of the Holliday junction resolvase
AE008917	804542	nsyn	T	G	S	A	BMEI0784	ATPase related to the helicase subunit of the Holliday junction resolvase
AE008917	808003	intergenic	A	G	-	-	-	-
AE008917	808612	nsyn	A	G	D	G	BMEI0789	Alanyl-tRNA synthetase
AE008917	811741	syn	A	G	T	T	BMEI0790	Alkaline phosphatase
AE008917	812125	syn	C	T	P	P	BMEI0790	Alkaline phosphatase
AE008917	812594	intergenic	A	C	-	-	-	-
AE008917	820547	intergenic	C	T	-	-	-	-
AE008917	821873	nsyn	A	G	V	A	BMEI0799	Methylmalonyl-CoA mutase, N-terminal domain/subunit
AE008917	829976	intergenic	C	T	-	-	-	-
AE008917	830025	intergenic	G	T	-	-	-	-
AE008917	831270	intergenic	G	A	-	-	-	-
AE008917	831352	intergenic	T	C	-	-	-	-
AE008917	831462	syn	G	A	T	T	BMEI0808	Predicted transcriptional regulators
AE008917	833430	syn	T	G	T	T	BMEI0810	Uncharacterized conserved protein
AE008917	834012	intergenic	C	T	-	-	-	-
AE008917	834278	intergenic	T	C	-	-	-	-
AE008917	834408	nsyn	C	T	L	F	BMEI0811	L-serine deaminase
AE008917	834581	nsyn	G	A	M	I	BMEI0811	L-serine deaminase
AE008917	834955	nsyn	T	C	V	A	BMEI0811	L-serine deaminase
AE008917	837169	syn	A	G	N	N	BMEI0814	D-alanyl-D-alanine carboxypeptidase
AE008917	837910	syn	T	C	L	L	BMEI0814	D-alanyl-D-alanine carboxypeptidase
AE008917	841859	nsyn	A	G	V	A	BMEI0817	Diadenosine tetraphosphate (Ap4A) hydrolase and other HIT family hydrolases
AE008917	843459	syn	C	T	P	P	BMEI0819	Glycerophosphoryl diester phosphodiesterase
AE008917	844402	syn	A	G	S	S	BMEI0820	Putative translation initiation inhibitor, yjgF family

AE008917	845034	syn	T	C	R	R	BMEI0821	NA
AE008917	845838	syn	T	C	P	P	BMEI0822	Uncharacterized protein conserved in bacteria with a cystatin-like fold [illegible]
AE008917	846545	nsyn	A	T	Y	F	BMEI0823	Ribosomal protein S2
AE008917	846590	nsyn	G	C	G	A	BMEI0823	Ribosomal protein S2
AE008917	848713	syn	T	C	C	C	BMEI0825	Uridylate kinase
AE008917	848725	syn	G	A	S	S	BMEI0825	Uridylate kinase
AE008917	848939	nsyn	G	A	E	K	BMEI0825	Uridylate kinase
AE008917	850535	intergenic	G	A	-	-	-	-
AE008917	851140	syn	G	A	A	A	BMEI0828	CDP-diglyceride synthetase
AE008917	851144	nsyn	A	G	T	A	BMEI0828	CDP-diglyceride synthetase
AE008917	852831	intergenic	C	T	-	-	-	-
AE008917	854307	syn	T	G	G	G	BMEI0830	Outer membrane protein/protective antigen OMA87
AE008917	855331	intergenic	C	G	-	-	-	-
AE008917	855338	intergenic	A	C	-	-	-	-
AE008917	855340	intergenic	G	C	-	-	-	-
AE008917	855605	syn	G	A	P	P	BMEI0831	UDP-3-O-[3-hydroxymyristoyl] glucosamine N-acyltransferase
AE008917	856034	syn	C	T	N	N	BMEI0831	UDP-3-O-[3-hydroxymyristoyl] glucosamine N-acyltransferase
AE008917	857814	syn	A	G	A	A	BMEI0834	Uncharacterized protein conserved in bacteria
AE008917	859118	syn	T	C	G	G	BMEI0835	Lipid A disaccharide synthetase
AE008917	859925	intergenic	G	C	-	-	-	-
AE008917	859926	intergenic	G	C	-	-	-	-
AE008917	859928	intergenic	A	C	-	-	-	-
AE008917	859929	intergenic	G	C	-	-	-	-
AE008917	859932	intergenic	T	C	-	-	-	-
AE008917	859933	intergenic	T	C	-	-	-	-
AE008917	859935	intergenic	G	C	-	-	-	-
AE008917	859937	intergenic	T	C	-	-	-	-
AE008917	859938	intergenic	G	C	-	-	-	-
AE008917	861334	intergenic	C	T	-	-	-	-
AE008917	861373	intergenic	T	A	-	-	-	-
AE008917	865862	nsyn	T	C	I	V	BMEI0840	SOS-response transcriptional repressors (RecA-mediated autopeptidases)
AE008917	867487	nsyn	T	G	K	N	BMEI0841	Molybdopterin biosynthesis enzyme
AE008917	872775	syn	C	T	I	I	BMEI0846	Triosephosphate isomerase
AE008917	872902	nsyn	A	G	I	V	BMEI0846	Triosephosphate isomerase

AE008917	873406	nsyn	A	C	T	P	BMEI0847	Preprotein translocase subunit SecG
AE008917	874025	nsyn	T	C	V	A	BMEI0848	Uncharacterized protein conserved in bacteria
AE008917	876133	nsyn	A	G	D	G	BMEI0849	CTP synthase (UTP-ammonia lyase)
AE008917	876926	syn	T	C	F	F	BMEI0850	3-deoxy-D-manno-octulosonic acid (KDO) 8-phosphate synthase
AE008917	880053	intergenic	A	G	-	-	-	-
AE008917	880339	nsyn	G	C	G	R	BMEI0853	Septum formation initiator
AE008917	881028	syn	T	C	I	I	BMEI0854	Pyruvate/2-oxoglutarate dehydrogenase complex, dehydrogenase (E1) component, eukaryotic type, alpha subunit
AE008917	881268	syn	C	T	G	G	BMEI0854	Pyruvate/2-oxoglutarate dehydrogenase complex, dehydrogenase (E1) component, eukaryotic type, alpha subunit
AE008917	881366	nsyn	G	A	R	K	BMEI0854	Pyruvate/2-oxoglutarate dehydrogenase complex, dehydrogenase (E1) component, eukaryotic type, alpha subunit
AE008917	883373	nsyn	C	A	D	E	BMEI0856	Pyruvate/2-oxoglutarate dehydrogenase complex, dehydrogenase (E1) component, eukaryotic type, alpha subunit
AE008917	883570	nsyn	G	A	R	H	BMEI0856	Pyruvate/2-oxoglutarate dehydrogenase complex, dehydrogenase (E1) component, eukaryotic type, alpha subunit
AE008917	884894	nsyn	C	T	T	I	BMEI0857	Pyruvate/2-oxoglutarate dehydrogenase complex, dehydrogenase (E1) component, eukaryotic type, alpha subunit
AE008917	884937	syn	A	C	G	G	BMEI0857	Pyruvate/2-oxoglutarate dehydrogenase complex, dehydrogenase (E1) component, eukaryotic type, alpha subunit
AE008917	885291	syn	C	T	N	N	BMEI0857	Pyruvate/2-oxoglutarate dehydrogenase complex, dehydrogenase (E1) component, eukaryotic type, alpha subunit
AE008917	886200	nsyn	C	T	P	L	BMEI0858	NA
AE008917	889330	syn	T	C	P	P	BMEI0863	4-diphosphocytidyl-2-methyl-D-erithritol synthase
AE008917	893179	nsyn	G	A	G	S	BMEI0866	Response regulator containing CheY-like receiver, AAA-type ATPase, and DNA-binding domains
AE008917	893668	nsyn	G	T	V	L	BMEI0866	Response regulator containing CheY-like receiver, AAA-type ATPase, and DNA-binding domains
AE008917	894543	syn	T	C	R	R	BMEI0867	Signal transduction histidine kinase involved in nitrogen fixation and metabolism regulation
AE008917	896005	nsyn	C	A	Q	K	BMEI0867	Signal transduction histidine kinase involved in nitrogen fixation and metabolism regulation
AE008917	899473	syn	C	T	I	I	BMEI0870	NA
AE008917	899614	nsyn	G	A	M	I	BMEI0870	NA
AE008917	899620	nsyn	T	G	I	M	BMEI0870	NA
AE008917	900915	nsyn	A	G	K	E	BMEI0873	GTPases
AE008917	901522	nsyn	C	A	T	K	BMEI0873	GTPases

AE008917	901585	nsyn	T	C	M	T	BMEI0873	GTPases
AE008917	904720	intergenic	C	T	-	-	-	-
AE008917	907161	syn	T	C	I	I	BMEI0876	ATP-dependent Lon protease, bacterial type
AE008917	907578	intergenic	C	T	-	-	-	-
AE008917	907587	intergenic	A	C	-	-	-	-
AE008917	908022	intergenic	G	A	-	-	-	-
AE008917	908823	nsyn	T	C	I	V	BMEI0878	Excinuclease ATPase subunit
AE008917	909677	nsyn	T	A	N	F	BMEI0878	Excinuclease ATPase subunit
AE008917	909678	nsyn	T	A	N	F	BMEI0878	Excinuclease ATPase subunit
AE008917	910846	syn	C	T	L	L	BMEI0878	Excinuclease ATPase subunit
AE008917	912350	nsyn	A	G	S	G	BMEI0880	Single-stranded DNA-binding protein
AE008917	913526	syn	G	A	L	L	BMEI0881	Transcriptional regulators
AE008917	913659	syn	G	A	I	I	BMEI0881	Transcriptional regulators
AE008917	914397	syn	C	T	L	L	BMEI0882	Uncharacterized component of phosphonate metabolism
AE008917	915475	intergenic	C	T	-	-	-	-
AE008917	917510	nsyn	C	G	L	V	BMEI0884	Type IIA topoisomerase (DNA gyrase/topo II, topoisomerase IV), A subunit
AE008917	920007	syn	G	A	V	V	BMEI0887	Peptidyl-prolyl cis-trans isomerase (rotamase) - cyclophilin family
AE008917	920142	nsyn	C	T	Q	*	BMEI0888	Peptidyl-prolyl cis-trans isomerase (rotamase) - cyclophilin family
AE008917	920926	nsyn	C	T	P	F	BMEI0889	S-adenosylmethionine:tRNA-ribosyltransferase-isomerase (queuine synthetase)
AE008917	920927	nsyn	C	T	P	F	BMEI0889	S-adenosylmethionine:tRNA-ribosyltransferase-isomerase (queuine synthetase)
AE008917	922412	syn	T	C	F	F	BMEI0890	Queuine/archaeosine tRNA-ribosyltransferase
AE008917	922946	syn	G	A	G	G	BMEI0890	Queuine/archaeosine tRNA-ribosyltransferase
AE008917	922949	nsyn	A	T	K	N	BMEI0890	Queuine/archaeosine tRNA-ribosyltransferase
AE008917	923006	syn	G	A	A	A	BMEI0890	Queuine/archaeosine tRNA-ribosyltransferase
AE008917	923993	nsyn	T	G	L	V	BMEI0892	Membrane-fusion protein
AE008917	924188	nsyn	A	T	M	S	BMEI0892	Membrane-fusion protein
AE008917	924189	nsyn	T	C	M	S	BMEI0892	Membrane-fusion protein
AE008917	924191	nsyn	C	G	P	A	BMEI0892	Membrane-fusion protein
AE008917	924217	syn	G	T	V	V	BMEI0892	Membrane-fusion protein
AE008917	924218	nsyn	A	G	K	E	BMEI0892	Membrane-fusion protein
AE008917	925513	syn	G	A	A	A	BMEI0893	Cation/multidrug efflux pump
AE008917	926065	syn	C	T	N	N	BMEI0893	Cation/multidrug efflux pump
AE008917	927419	nsyn	T	G	C	G	BMEI0893	Cation/multidrug efflux pump

AE008917	927473	nsyn	T	C	F	L	BMEI0893	Cation/multidrug efflux pump
AE008917	928035	intergenic	T	C	-	-	-	-
AE008917	928725	syn	T	C	P	P	BMEI0894	Coenzyme F420-dependent N5,N10-methylene tetrahydromethanopterin reductase and related flavin-dependent oxidoreductases
AE008917	930782	nsyn	A	G	N	S	BMEI0897	Acyl-CoA dehydrogenases
AE008917	930814	nsyn	T	C	F	L	BMEI0897	Acyl-CoA dehydrogenases
AE008917	931073	nsyn	G	A	G	D	BMEI0897	Acyl-CoA dehydrogenases
AE008917	931476	nsyn	G	T	W	C	BMEI0898	Predicted acyl-CoA transferases/carnitine dehydratase
AE008917	931827	syn	G	A	Q	Q	BMEI0898	Predicted acyl-CoA transferases/carnitine dehydratase
AE008917	932771	intergenic	T	C	-	-	-	-
AE008917	933378	nsyn	T	G	L	R	BMEI0899	Prophage antirepressor
AE008917	933552	nsyn	C	A	P	H	BMEI0899	Prophage antirepressor
AE008917	938268	intergenic	T	C	-	-	-	-
AE008917	938694	intergenic	A	C	-	-	-	-
AE008917	938760	intergenic	C	T	-	-	-	-
AE008917	938780	intergenic	A	G	-	-	-	-
AE008917	940002	intergenic	A	C	-	-	-	-
AE008917	940120	intergenic	A	G	-	-	-	-
AE008917	942036	nsyn	C	A	R	L	BMEI0908	Predicted esterase of the alpha-beta hydrolase superfamily
AE008917	943897	syn	A	G	L	L	BMEI0911	NifU homolog involved in Fe-S cluster formation
AE008917	944716	nsyn	T	G	S	A	BMEI0913	D-alanyl-D-alanine carboxypeptidase
AE008917	947323	syn	C	T	T	T	BMEI0915	Threonyl-tRNA synthetase
AE008917	947364	nsyn	T	A	L	H	BMEI0915	Threonyl-tRNA synthetase
AE008917	949658	nsyn	T	C	S	P	BMEI0917	Nitroreductase
AE008917	951023	nsyn	C	G	V	L	BMEI0920	Predicted pyrophosphatase
AE008917	951405	syn	A	G	N	N	BMEI0920	Predicted pyrophosphatase
AE008917	956638	nsyn	A	G	F	L	BMEI0925	Zn-dependent alcohol dehydrogenases
AE008917	957249	intergenic	T	C	-	-	-	-
AE008917	959486	intergenic	T	C	-	-	-	-
AE008917	961192	intergenic	C	G	-	-	-	-
AE008917	961438	intergenic	T	C	-	-	-	-
AE008917	963326	nsyn	A	G	V	A	BMEI0931	Rhodanese-related sulfurtransferase
AE008917	963879	intergenic	T	C	-	-	-	-
AE008917	965352	nsyn	A	G	V	A	BMEI0933	Cysteine synthase
AE008917	966554	nsyn	A	C	V	G	BMEI0934	Superfamily II DNA and RNA helicases

AE008917	966946	syn	T	C	L	L	BMEI0934	Superfamily II DNA and RNA helicases
AE008917	969228	nsyn	T	C	Y	C	BMEI0936	Multimeric flavodoxin WrbA
AE008917	970937	nsyn	A	C	T	P	BMEI0938	Universal stress protein UspA and related nucleotide-binding proteins
AE008917	970949	nsyn	A	C	T	P	BMEI0938	Universal stress protein UspA and related nucleotide-binding proteins
AE008917	972520	intergenic	T	C	-	-	-	-
AE008917	974814	syn	A	C	L	L	BMEI0943	Ribonucleotide reductase, alpha subunit
AE008917	976312	nsyn	C	A	L	M	BMEI0943	Ribonucleotide reductase, alpha subunit
AE008917	976702	nsyn	C	G	L	V	BMEI0943	Ribonucleotide reductase, alpha subunit
AE008917	981047	nsyn	A	G	V	A	BMEI0946	Predicted flavoproteins
AE008917	981555	intergenic	G	C	-	-	-	-
AE008917	981862	syn	T	G	G	G	BMEI0947	FOG: PAS/PAC domain
AE008917	983354	nsyn	C	G	G	A	BMEI0947	FOG: PAS/PAC domain
AE008917	983647	syn	G	A	G	G	BMEI0947	FOG: PAS/PAC domain
AE008917	983811	nsyn	G	C	P	A	BMEI0947	FOG: PAS/PAC domain
AE008917	985043	nsyn	G	C	K	N	BMEI0948	NA
AE008917	985417	nsyn	T	C	F	S	BMEI0948	NA
AE008917	988915	syn	A	G	K	K	BMEI0952	Predicted membrane protein
AE008917	989396	nsyn	T	A	S	T	BMEI0952	Predicted membrane protein
AE008917	989446	nsyn	C	A	D	E	BMEI0952	Predicted membrane protein
AE008917	990146	intergenic	G	A	-	-	-	-
AE008917	990879	intergenic	G	A	-	-	-	-
AE008917	992006	nsyn	A	G	F	L	BMEI0956	Dihydropteroate synthase and related enzymes
AE008917	992666	intergenic	T	C	-	-	-	-
AE008917	995448	intergenic	G	A	-	-	-	-
AE008917	998349	nsyn	G	A	A	T	BMEI0961	NA
AE008917	998921	syn	T	C	R	R	BMEI0961	NA
AE008917	1002091	syn	G	A	R	R	BMEI0964	ABC-type transport system involved in resistance to organic solvents, ATPase component
AE008917	1002276	nsyn	G	C	H	D	BMEI0964	ABC-type transport system involved in resistance to organic solvents, ATPase component

AE008917	1002322	syn	G	A	I	I	BMEI0964	ABC-type transport system involved in resistance to organic solvents, ATPase component
AE008917	1007126	nsyn	A	G	N	S	BMEI0967	Malic enzyme
AE008917	1007364	nsyn	C	G	N	K	BMEI0967	Malic enzyme
AE008917	1008498	nsyn	T	C	H	R	BMEI0968	Glutamyl- and glutaminyl-tRNA synthetases
AE008917	1011676	nsyn	G	T	P	T	BMEI0970	Diacylglycerol kinase
AE008917	1013354	intergenic	C	T	-	-	-	-
AE008917	1013608	nsyn	G	A	R	C	BMEI0972	Pyruvate/2-oxoglutarate dehydrogenase complex, dihydrolipoamide dehydrogenase (E3) component, and related enzymes
AE008917	1015369	nsyn	T	C	T	A	BMEI0973	Uncharacterized protein conserved in bacteria
AE008917	1016661	syn	C	T	R	R	BMEI0975	Predicted phosphatases
AE008917	1019035	nsyn	T	C	Y	C	BMEI0977	Predicted sugar kinase
AE008917	1020897	nsyn	T	G	F	V	BMEI0979	Glutamine synthetase
AE008917	1021287	intergenic	G	A	-	-	-	-
AE008917	1021787	nsyn	T	C	H	R	BMEI0980	Chaperone required for the assembly of the mitochondrial F1-ATPase
AE008917	1022248	nsyn	T	C	D	G	BMEI0981	Predicted phosphatases
AE008917	1026744	syn	A	G	L	L	BMEI0984	ABC-type multidrug transport system, ATPase and permease components
AE008917	1027544	syn	C	T	T	T	BMEI0985	Metal-dependent hydrolases of the beta-lactamase superfamily I
AE008917	1028635	nsyn	G	A	A	V	BMEI0987	Methionyl-tRNA synthetase
AE008917	1030085	nsyn	T	G	N	H	BMEI0987	Methionyl-tRNA synthetase
AE008917	1030502	syn	C	T	K	K	BMEI0988	ATPase involved in DNA replication
AE008917	1032892	syn	T	C	S	S	BMEI0990	D-alanyl-D-alanine carboxypeptidase
AE008917	1033330	nsyn	T	C	D	G	BMEI0991	Lipoproteins
AE008917	1034792	intergenic	A	C	-	-	-	-
AE008917	1034793	intergenic	A	C	-	-	-	-
AE008917	1036425	nsyn	A	C	N	K	BMEI0995	Putative secretion activating protein
AE008917	1036646	nsyn	T	C	N	D	BMEI0995	Putative secretion activating protein
AE008917	1036733	nsyn	G	C	L	V	BMEI0995	Putative secretion activating protein
AE008917	1037831	nsyn	C	G	R	P	BMEI0997	Glycosyltransferase
AE008917	1038587	nsyn	A	G	L	S	BMEI0997	Glycosyltransferase
AE008917	1040101	nsyn	A	G	V	T	BMEI0998	Glycosyltransferase
AE008917	1040102	nsyn	C	T	V	T	BMEI0998	Glycosyltransferase
AE008917	1040194	intergenic	G	A	-	-	-	-
AE008917	1040301	intergenic	A	G	-	-	-	-

AE008917	1041386	nsyn	A	T	V	D	BMEI0999	NA
AE008917	1042352	nsyn	C	T	W	*	BMEI1000	NA
AE008917	1044810	nsyn	T	A	F	I	BMEI1004	Uncharacterized protein conserved in bacteria
AE008917	1045241	intergenic	C	G	-	-	-	-
AE008917	1045648	nsyn	C	A	H	N	BMEI1006	Uncharacterized conserved protein
AE008917	1047125	nsyn	G	A	Q	*	BMEI1008	NA
AE008917	1049774	nsyn	A	G	T	A	BMEI1012	Integrase
AE008917	1050320	nsyn	T	C	Y	H	BMEI1012	Integrase
AE008917	1050370	syn	T	C	G	G	BMEI1012	Integrase
AE008917	1050687	intergenic	C	T	-	-	-	-
AE008917	1052926	nsyn	T	G	K	Q	BMEI1014	Uncharacterized protein conserved in bacteria
AE008917	1054034	intergenic	C	T	-	-	-	-
AE008917	1054972	syn	T	G	P	P	BMEI1016	Tartrate dehydratase alpha subunit/Fumarate hydratase class I, N-terminal domain
AE008917	1056580	syn	T	C	L	L	BMEI1017	2-polyprenyl-6-methoxyphenol hydroxylase and related FAD-dependent oxidoreductases
AE008917	1061797	nsyn	G	A	V	I	BMEI1024	3-hydroxyisobutyrate dehydrogenase and related beta-hydroxyacid dehydrogenases
AE008917	1061884	nsyn	C	A	L	I	BMEI1024	3-hydroxyisobutyrate dehydrogenase and related beta-hydroxyacid dehydrogenases
AE008917	1062333	syn	T	C	L	L	BMEI1024	3-hydroxyisobutyrate dehydrogenase and related beta-hydroxyacid dehydrogenases
AE008917	1064054	intergenic	T	C	-	-	-	-
AE008917	1064258	nsyn	G	C	L	V	BMEI1027	Valyl-tRNA synthetase
AE008917	1066010	nsyn	T	G	T	P	BMEI1027	Valyl-tRNA synthetase
AE008917	1066015	nsyn	A	G	I	T	BMEI1027	Valyl-tRNA synthetase
AE008917	1066383	syn	T	C	K	K	BMEI1027	Valyl-tRNA synthetase
AE008917	1066689	syn	T	C	Q	Q	BMEI1027	Valyl-tRNA synthetase
AE008917	1067837	syn	C	T	A	A	BMEI1028	Uncharacterized protein conserved in bacteria
AE008917	1072179	syn	G	A	*	*	BMEI1034	Uncharacterized conserved protein
AE008917	1072203	intergenic	G	A	-	-	-	-
AE008917	1072374	intergenic	T	G	-	-	-	-
AE008917	1072376	intergenic	T	C	-	-	-	-
AE008917	1072386	intergenic	G	C	-	-	-	-
AE008917	1074664	syn	G	T	V	V	BMEI1036	Predicted sugar kinase

AE008917	1078692	syn	A	G	S	S	BMEI1040	ABC-type transport system involved in Fe-S cluster assembly, permease component
AE008917	1081851	intergenic	G	A	-	-	-	-
AE008917	1082863	nsyn	G	A	P	S	BMEI1043	Cysteine sulfinate desulfinase/cysteine desulfurase and related enzymes
AE008917	1083310	intergenic	A	G	-	-	-	-
AE008917	1084015	nsyn	G	T	*	Y	BMEI1044	Predicted hydrolase of the alpha/beta superfamily
AE008917	1084265	nsyn	C	A	L	M	BMEI1045	Membrane transporters of cations and cationic drugs
AE008917	1084420	syn	G	A	V	V	BMEI1045	Membrane transporters of cations and cationic drugs
AE008917	1084626	nsyn	G	C	Q	E	BMEI1046	Predicted molecular chaperone distantly related to HSP70-fold metalloproteases
AE008917	1085041	syn	C	G	G	G	BMEI1046	Predicted molecular chaperone distantly related to HSP70-fold metalloproteases
AE008917	1086482	nsyn	A	G	T	A	BMEI1047	Tyrosyl-tRNA synthetase
AE008917	1086953	intergenic	A	G	-	-	-	-
AE008917	1088013	nsyn	A	G	S	P	BMEI1048	NA
AE008917	1088893	syn	A	G	G	G	BMEI1048	NA
AE008917	1089208	syn	A	G	S	S	BMEI1048	NA
AE008917	1090857	nsyn	C	A	T	K	BMEI1049	Peroxiredoxin
AE008917	1091445	nsyn	C	T	A	V	BMEI1050	Uncharacterized protein conserved in bacteria
AE008917	1092652	intergenic	A	C	-	-	-	-
AE008917	1097525	intergenic	A	G	-	-	-	-
AE008917	1098277	nsyn	T	A	N	I	BMEI1056	N-acetylmuramoyl-L-alanine amidase
AE008917	1098726	syn	T	G	V	V	BMEI1056	N-acetylmuramoyl-L-alanine amidase
AE008917	1099153	intergenic	A	T	-	-	-	-
AE008917	1099252	intergenic	G	A	-	-	-	-
AE008917	1105736	syn	T	C	H	H	BMEI1060	Protein-disulfide isomerase
AE008917	1106610	nsyn	A	G	T	A	BMEI1061	3-dehydroquinate dehydratase II
AE008917	1110914	nsyn	C	T	G	S	BMEI1067	Histone acetyltransferase HPA2 and related acetyltransferases
AE008917	1112614	intergenic	G	C	-	-	-	-
AE008917	1112616	intergenic	G	C	-	-	-	-
AE008917	1112618	intergenic	A	C	-	-	-	-
AE008917	1112619	intergenic	A	C	-	-	-	-
AE008917	1112621	intergenic	G	C	-	-	-	-
AE008917	1112623	intergenic	G	C	-	-	-	-
AE008917	1112625	intergenic	G	C	-	-	-	-

AE008917	1112626	intergenic	G	C	-	-	-	-
AE008917	1115811	nsyn	A	G	Y	H	BMEI1071	Predicted membrane protein
AE008917	1119167	nsyn	G	A	A	V	BMEI1075	Uncharacterized conserved protein
AE008917	1119421	syn	A	G	C	C	BMEI1075	Uncharacterized conserved protein
AE008917	1121655	syn	G	A	G	G	BMEI1076	Preprotein translocase subunit SecD
AE008917	1121735	nsyn	G	A	L	F	BMEI1076	Preprotein translocase subunit SecD
AE008917	1123733	syn	G	A	F	F	BMEI1079	Membrane proteins related to metalloendopeptidases
AE008917	1123766	syn	A	G	R	R	BMEI1079	Membrane proteins related to metalloendopeptidases
AE008917	1124183	syn	T	C	A	A	BMEI1079	Membrane proteins related to metalloendopeptidases
AE008917	1124333	syn	A	G	V	V	BMEI1079	Membrane proteins related to metalloendopeptidases
AE008917	1124636	syn	A	G	V	V	BMEI1079	Membrane proteins related to metalloendopeptidases
AE008917	1126826	syn	T	C	E	E	BMEI1082	Seryl-tRNA synthetase
AE008917	1128560	nsyn	G	A	L	F	BMEI1083	Sec-independent protein secretion pathway component TatC
AE008917	1129698	syn	G	A	L	L	BMEI1084	Sec-independent protein secretion pathway components
AE008917	1129928	syn	T	C	G	G	BMEI1085	Predicted transcriptional regulator containing the HTH domain
AE008917	1130287	nsyn	T	C	T	A	BMEI1085	Predicted transcriptional regulator containing the HTH domain
AE008917	1130934	syn	A	G	A	A	BMEI1086	Uncharacterized conserved protein
AE008917	1134169	nsyn	T	C	T	A	BMEI1088	NA
AE008917	1136644	nsyn	T	C	H	R	BMEI1089	Arginyl-tRNA synthetase
AE008917	1136730	syn	A	G	D	D	BMEI1089	Arginyl-tRNA synthetase
AE008917	1138115	syn	G	A	R	R	BMEI1090	dGTP triphosphohydrolase
AE008917	1140126	nsyn	G	A	A	V	BMEI1092	Uncharacterized protein conserved in bacteria
AE008917	1144820	intergenic	G	C	-	-	-	-
AE008917	1144825	intergenic	A	G	-	-	-	-
AE008917	1144826	intergenic	T	G	-	-	-	-
AE008917	1144830	intergenic	C	G	-	-	-	-
AE008917	1144834	intergenic	C	T	-	-	-	-
AE008917	1145211	syn	C	T	P	P	BMEI1099	NaMN:DMB phosphoribosyltransferase
AE008917	1145248	nsyn	A	T	L	Q	BMEI1099	NaMN:DMB phosphoribosyltransferase
AE008917	1145844	syn	G	A	D	D	BMEI1099	NaMN:DMB phosphoribosyltransferase
AE008917	1147248	nsyn	C	A	L	I	BMEI1102	Predicted aspartyl protease
AE008917	1147550	syn	G	A	A	A	BMEI1102	Predicted aspartyl protease
AE008917	1147784	syn	C	A	A	A	BMEI1103	tRNA-dihydrouridine synthase
AE008917	1150114	syn	G	A	V	V	BMEI1105	Predicted phosphatase/phosphohexomutase
AE008917	1150159	syn	G	T	V	V	BMEI1105	Predicted phosphatase/phosphohexomutase

AE008917	1152088	syn	T	C	E	E	BMEI1107	NA
AE008917	1153375	nsyn	G	T	G	V	BMEI1108	Sugar kinases, ribokinase family
AE008917	1153671	nsyn	A	G	I	T	BMEI1109	Galactose mutarotase and related enzymes
AE008917	1154038	nsyn	T	C	R	G	BMEI1109	Galactose mutarotase and related enzymes
AE008917	1154377	nsyn	G	A	R	C	BMEI1109	Galactose mutarotase and related enzymes
AE008917	1154789	intergenic	T	C	-	-	-	-
AE008917	1154802	intergenic	C	A	-	-	-	-
AE008917	1154809	intergenic	T	G	-	-	-	-
AE008917	1154823	intergenic	C	T	-	-	-	-
AE008917	1156169	intergenic	C	T	-	-	-	-
AE008917	1157087	syn	C	A	R	R	BMEI1112	3-oxoacyl-(acyl-carrier-protein) synthase
AE008917	1160091	nsyn	T	A	V	E	BMEI1115	Lauroyl/myristoyl acyltransferase
AE008917	1160512	syn	C	G	S	S	BMEI1115	Lauroyl/myristoyl acyltransferase
AE008917	1166799	syn	T	C	A	A	BMEI1122	Phosphoribosylaminoimidazolesuccinocarboxamide (SAICAR) synthase
AE008917	1168261	intergenic	A	G	-	-	-	-
AE008917	1168805	nsyn	G	C	V	L	BMEI1125	Glutathione S-transferase
AE008917	1169041	syn	A	G	Q	Q	BMEI1125	Glutathione S-transferase
AE008917	1170582	syn	T	C	D	D	BMEI1127	Phosphoribosylformylglycinamidine (FGAM) synthase, synthetase domain
AE008917	1171147	nsyn	A	C	T	P	BMEI1127	Phosphoribosylformylglycinamidine (FGAM) synthase, synthetase domain
AE008917	1171153	nsyn	A	C	T	P	BMEI1127	Phosphoribosylformylglycinamidine (FGAM) synthase, synthetase domain
AE008917	1172033	intergenic	T	G	-	-	-	-
AE008917	1172678	nsyn	C	T	T	M	BMEI1129	Glutaredoxin-related protein
AE008917	1176275	nsyn	C	G	V	L	BMEI1132	Predicted ATPase of the PP-loop superfamily implicated in cell cycle control
AE008917	1176737	intergenic	A	G	-	-	-	-
AE008917	1176851	intergenic	G	C	-	-	-	-
AE008917	1176853	intergenic	T	C	-	-	-	-
AE008917	1176854	intergenic	G	C	-	-	-	-
AE008917	1176857	intergenic	T	C	-	-	-	-
AE008917	1176860	intergenic	A	C	-	-	-	-
AE008917	1176862	intergenic	A	G	-	-	-	-
AE008917	1176864	intergenic	C	G	-	-	-	-

AE008917	1176866	intergenic	C	G	-	-	-	-
AE008917	1178540	nsyn	G	A	G	S	BMEI1134	Uncharacterized protein conserved in bacteria
AE008917	1183675	intergenic	T	C	-	-	-	-
AE008917	1183767	intergenic	T	C	-	-	-	-
AE008917	1184649	nsyn	C	T	V	I	BMEI1138	ABC-type antimicrobial peptide transport system, ATPase component
AE008917	1185546	syn	C	T	A	A	BMEI1139	ABC-type transport system, involved in lipoprotein release, permease component
AE008917	1185632	nsyn	T	C	N	D	BMEI1139	ABC-type transport system, involved in lipoprotein release, permease component
AE008917	1185734	nsyn	G	A	R	C	BMEI1139	ABC-type transport system, involved in lipoprotein release, permease component
AE008917	1185738	syn	A	C	G	G	BMEI1139	ABC-type transport system, involved in lipoprotein release, permease component
AE008917	1185756	syn	G	A	V	V	BMEI1139	ABC-type transport system, involved in lipoprotein release, permease component
AE008917	1187406	intergenic	G	A	-	-	-	-
AE008917	1189265	syn	C	T	V	V	BMEI1143	Predicted hydrolase of the metallo-beta-lactamase superfamily
AE008917	1191273	syn	T	C	K	K	BMEI1144	Biotin-(acetyl-CoA carboxylase) ligase
AE008917	1191929	nsyn	G	A	T	I	BMEI1145	NADH:ubiquinone oxidoreductase subunit 2 (chain N)
AE008917	1193494	syn	T	C	S	S	BMEI1146	NADH:ubiquinone oxidoreductase subunit 4 (chain M)
AE008917	1195222	nsyn	C	G	G	A	BMEI1147	NADH:ubiquinone oxidoreductase subunit 5 (chain L)/Multisubunit Na+/H+ antiporter, MnhA subunit
AE008917	1197656	nsyn	T	C	H	R	BMEI1150	Formate hydrogenlyase subunit 6/NADH:ubiquinone oxidoreductase 23 kD subunit (chain I)
AE008917	1198880	nsyn	A	G	F	S	BMEI1151	NADH:ubiquinone oxidoreductase subunit 1 (chain H)
AE008917	1198970	nsyn	C	G	R	A	BMEI1151	NADH:ubiquinone oxidoreductase subunit 1 (chain H)
AE008917	1198971	nsyn	G	C	R	A	BMEI1151	NADH:ubiquinone oxidoreductase subunit 1 (chain H)
AE008917	1199316	nsyn	C	G	G	A	BMEI1152	NADH dehydrogenase/NADH:ubiquinone oxidoreductase 75 kD subunit (chain G)
AE008917	1199346	nsyn	C	G	R	A	BMEI1152	NADH dehydrogenase/NADH:ubiquinone oxidoreductase 75 kD subunit (chain G)
AE008917	1199347	nsyn	G	C	R	A	BMEI1152	NADH dehydrogenase/NADH:ubiquinone oxidoreductase 75 kD subunit (chain G)
AE008917	1199492	syn	T	C	P	P	BMEI1152	NADH dehydrogenase/NADH:ubiquinone oxidoreductase 75 kD subunit (chain G)

AE008917	1200119	syn	G	T	I	I	BMEI1152	NADH dehydrogenase/NADH:ubiquinone oxidoreductase 75 kD subunit (chain G)
AE008917	1200559	nsyn	C	T	V	M	BMEI1152	NADH dehydrogenase/NADH:ubiquinone oxidoreductase 75 kD subunit (chain G)
AE008917	1202811	nsyn	C	T	E	K	BMEI1154	NADH:ubiquinone oxidoreductase 24 kD subunit
AE008917	1203444	syn	A	G	L	L	BMEI1155	NADH:ubiquinone oxidoreductase 49 kD subunit 7
AE008917	1203796	nsyn	A	G	V	A	BMEI1155	NADH:ubiquinone oxidoreductase 49 kD subunit 7
AE008917	1207721	intergenic	A	G	-	-	-	-
AE008917	1208184	syn	T	C	N	N	BMEI1160	NA
AE008917	1208308	intergenic	C	T	-	-	-	-
AE008917	1209745	nsyn	A	G	L	S	BMEI1163	Transposase and inactivated derivatives
AE008917	1209804	syn	G	A	G	G	BMEI1163	Transposase and inactivated derivatives
AE008917	1209833	nsyn	C	T	D	N	BMEI1163	Transposase and inactivated derivatives
AE008917	1209873	syn	C	T	A	A	BMEI1163	Transposase and inactivated derivatives
AE008917	1209902	nsyn	C	T	E	K	BMEI1163	Transposase and inactivated derivatives
AE008917	1209903	syn	T	C	L	L	BMEI1163	Transposase and inactivated derivatives
AE008917	1210366	nsyn	A	G	F	L	BMEI1164	Transposase and inactivated derivatives
AE008917	1213768	syn	T	G	V	V	BMEI1168	Ribosomal protein L13
AE008917	1214112	nsyn	C	T	A	V	BMEI1168	Ribosomal protein L13
AE008917	1214204	nsyn	C	T	A	V	BMEI1169	Ribosomal protein S9
AE008917	1215199	nsyn	A	G	T	A	BMEI1170	Arginase/agmatinase/formimionoglutamate hydrolase, arginase family
AE008917	1215420	syn	A	G	A	A	BMEI1170	Arginase/agmatinase/formimionoglutamate hydrolase, arginase family
AE008917	1216056	nsyn	A	G	T	A	BMEI1171	Acetylglutamate semialdehyde dehydrogenase
AE008917	1218079	intergenic	C	T	-	-	-	-
AE008917	1218154	intergenic	A	G	-	-	-	-
AE008917	1218157	intergenic	A	G	-	-	-	-
AE008917	1220641	syn	G	A	L	L	BMEI1177	Sugar transferases involved in lipopolysaccharide synthesis
AE008917	1220988	nsyn	A	G	H	R	BMEI1177	Sugar transferases involved in lipopolysaccharide synthesis
AE008917	1222428	intergenic	C	T	-	-	-	-
AE008917	1222920	syn	C	G	T	T	BMEI1178	Predicted transcriptional regulators
AE008917	1223243	intergenic	A	T	-	-	-	-
AE008917	1223266	intergenic	C	T	-	-	-	-
AE008917	1224216	syn	A	C	V	V	BMEI1180	3-oxoacyl-[acyl-carrier-protein] synthase III
AE008917	1224756	syn	A	T	A	A	BMEI1180	3-oxoacyl-[acyl-carrier-protein] synthase III

AE008917	1224995	nsyn	C	T	V	I	BMEI1180	3-oxoacyl-[acyl-carrier-protein] synthase III
AE008917	1228344	intergenic	A	C	-	-	-	-
AE008917	1228774	syn	T	C	E	E	BMEI1185	Inorganic pyrophosphatase
AE008917	1229452	syn	A	G	R	R	BMEI1185	Inorganic pyrophosphatase
AE008917	1229769	syn	G	A	L	L	BMEI1185	Inorganic pyrophosphatase
AE008917	1230440	nsyn	G	A	T	M	BMEI1185	Inorganic pyrophosphatase
AE008917	1231472	nsyn	T	G	Q	P	BMEI1186	Transcription termination factor
AE008917	1231698	syn	G	A	S	S	BMEI1187	Riboflavin synthase beta-chain
AE008917	1231911	syn	G	A	A	A	BMEI1187	Riboflavin synthase beta-chain
AE008917	1232352	nsyn	C	T	A	T	BMEI1188	Riboflavin synthase alpha chain
AE008917	1232756	nsyn	T	C	T	A	BMEI1189	Pyrimidine reductase, riboflavin biosynthesis
AE008917	1233627	syn	G	T	G	G	BMEI1189	Pyrimidine reductase, riboflavin biosynthesis
AE008917	1234747	nsyn	C	T	A	T	BMEI1191	Glycine/serine hydroxymethyltransferase
AE008917	1235229	nsyn	C	T	C	Y	BMEI1191	Glycine/serine hydroxymethyltransferase
AE008917	1237367	intergenic	A	G	-	-	-	-
AE008917	1237429	intergenic	T	C	-	-	-	-
AE008917	1237623	intergenic	T	G	-	-	-	-
AE008917	1237835	nsyn	T	C	D	G	BMEI1194	Transcriptional regulators
AE008917	1240426	nsyn	C	G	T	S	BMEI1197	Delta-aminolevulinic acid dehydratase
AE008917	1246088	nsyn	A	G	I	M	BMEI1201	NA
AE008917	1250160	intergenic	A	G	-	-	-	-
AE008917	1250764	nsyn	T	C	E	G	BMEI1204	Exopolyphosphatase
AE008917	1251303	syn	C	A	G	G	BMEI1204	Exopolyphosphatase
AE008917	1252265	syn	C	T	S	S	BMEI1205	Polyphosphate kinase
AE008917	1254302	nsyn	A	G	K	E	BMEI1206	Uncharacterized protein conserved in bacteria
AE008917	1254380	nsyn	A	G	N	D	BMEI1206	Uncharacterized protein conserved in bacteria
AE008917	1256394	syn	G	T	G	G	BMEI1208	ABC-type polar amino acid transport system, ATPase component
AE008917	1257183	nsyn	G	A	P	L	BMEI1209	ABC-type amino acid transport system, permease component
AE008917	1258410	syn	C	A	G	G	BMEI1210	ABC-type amino acid transport system, permease component
AE008917	1258429	nsyn	G	A	S	L	BMEI1210	ABC-type amino acid transport system, permease component
AE008917	1258903	nsyn	G	A	P	L	BMEI1210	ABC-type amino acid transport system, permease component
AE008917	1259769	nsyn	A	G	V	A	BMEI1211	ABC-type amino acid transport/signal transduction systems, periplasmic component/domain
AE008917	1262348	syn	C	A	A	A	BMEI1213	Cystathionine beta-lyases/cystathionine gamma-synthases
AE008917	1263598	nsyn	G	A	A	T	BMEI1215	Uncharacterized protein with a bacterial SH3 domain homologue

AE008917	1265791	syn	T	C	K	K	BMEI1217	NA
AE008917	1265907	nsyn	T	C	T	A	BMEI1217	NA
AE008917	1266271	nsyn	A	G	I	V	BMEI1217	NA
AE008917	1266273	nsyn	T	C	I	V	BMEI1217	NA
AE008917	1266613	syn	G	A	R	R	BMEI1217	NA
AE008917	1268180	nsyn	G	A	A	V	BMEI1218	NA
AE008917	1268565	intergenic	C	G	-	-	-	-
AE008917	1268662	intergenic	C	G	-	-	-	-
AE008917	1270217	intergenic	A	T	-	-	-	-
AE008917	1270745	intergenic	G	A	-	-	-	-
AE008917	1271708	syn	C	T	R	R	BMEI1222	NA
AE008917	1271940	syn	C	T	L	L	BMEI1223	Transposase and inactivated derivatives
AE008917	1271995	nsyn	G	A	A	V	BMEI1223	Transposase and inactivated derivatives
AE008917	1272039	syn	C	T	Q	Q	BMEI1223	Transposase and inactivated derivatives
AE008917	1273636	nsyn	C	A	R	L	BMEI1223	Transposase and inactivated derivatives
AE008917	1273643	nsyn	T	C	K	E	BMEI1223	Transposase and inactivated derivatives
AE008917	1273648	nsyn	C	A	R	L	BMEI1223	Transposase and inactivated derivatives
AE008917	1275244	nsyn	T	C	V	A	BMEI1225	NA
AE008917	1276292	syn	G	A	P	P	BMEI1226	Uncharacterized conserved protein
AE008917	1276451	nsyn	A	G	E	G	BMEI1227	NA
AE008917	1276596	nsyn	G	C	K	N	BMEI1227	NA
AE008917	1276977	nsyn	G	C	W	C	BMEI1227	NA
AE008917	1277047	nsyn	A	G	K	E	BMEI1227	NA
AE008917	1280223	intergenic	A	C	-	-	-	-
AE008917	1285081	nsyn	A	C	Y	S	BMEI1236	NA
AE008917	1288741	intergenic	A	G	-	-	-	-
AE008917	1289010	syn	G	A	K	K	BMEI1240	Phosphoribosylaminoimidazole (AIR) synthetase
AE008917	1289160	syn	T	C	L	L	BMEI1240	Phosphoribosylaminoimidazole (AIR) synthetase
AE008917	1290841	nsyn	G	A	D	N	BMEI1242	NA
AE008917	1291540	nsyn	C	T	V	I	BMEI1243	Anaerobic dehydrogenases, typically selenocysteine-containing
AE008917	1292134	nsyn	C	T	A	T	BMEI1243	Anaerobic dehydrogenases, typically selenocysteine-containing
AE008917	1295360	nsyn	C	G	D	E	BMEI1245	Protein involved in catabolism of external DNA
AE008917	1296994	nsyn	A	G	N	D	BMEI1248	Glutathione S-transferase
AE008917	1297891	syn	A	G	G	G	BMEI1249	Opacity protein and related surface antigens

AE008917	1299272	syn	T	C	G	G	BMEI1250	Dehydrogenases with different specificities (related to short-chain alcohol dehydrogenases)
AE008917	1299920	nsyn	G	A	A	T	BMEI1251	Nuclease subunit of the excinuclease complex
AE008917	1300405	syn	A	G	L	L	BMEI1251	Nuclease subunit of the excinuclease complex
AE008917	1300786	syn	T	C	R	R	BMEI1251	Nuclease subunit of the excinuclease complex
AE008917	1302850	intergenic	T	C	-	-	-	-
AE008917	1304093	nsyn	C	T	A	T	BMEI1256	Nucleoside diphosphate kinase
AE008917	1305524	nsyn	C	A	H	Q	BMEI1258	ATPase components of ABC transporters with duplicated ATPase domains
AE008917	1305722	syn	T	C	L	L	BMEI1258	ATPase components of ABC transporters with duplicated ATPase domains
AE008917	1308930	syn	G	A	F	F	BMEI1261	Leucyl aminopeptidase
AE008917	1309256	nsyn	C	T	V	I	BMEI1261	Leucyl aminopeptidase
AE008917	1309506	syn	A	G	V	V	BMEI1261	Leucyl aminopeptidase
AE008917	1309597	nsyn	A	G	V	A	BMEI1261	Leucyl aminopeptidase
AE008917	1309665	syn	G	C	S	S	BMEI1261	Leucyl aminopeptidase
AE008917	1312173	syn	A	G	S	S	BMEI1263	Predicted permeases
AE008917	1313094	nsyn	G	A	E	K	BMEI1264	Organic solvent tolerance protein OstA
AE008917	1313326	nsyn	A	C	Q	P	BMEI1264	Organic solvent tolerance protein OstA
AE008917	1315417	nsyn	T	G	L	R	BMEI1265	Parvulin-like peptidyl-prolyl isomerase
AE008917	1315446	nsyn	A	G	N	D	BMEI1265	Parvulin-like peptidyl-prolyl isomerase
AE008917	1316721	syn	A	G	E	E	BMEI1266	Pyridoxal phosphate biosynthesis protein
AE008917	1317697	nsyn	T	C	L	P	BMEI1267	Dimethyladenosine transferase (rRNA methylation)
AE008917	1317948	syn	T	C	L	L	BMEI1267	Dimethyladenosine transferase (rRNA methylation)
AE008917	1318369	syn	C	T	V	V	BMEI1268	Membrane proteins related to metalloendopeptidases
AE008917	1319083	syn	A	C	T	T	BMEI1268	Membrane proteins related to metalloendopeptidases
AE008917	1320266	nsyn	T	C	E	G	BMEI1269	Predicted permeases
AE008917	1324862	nsyn	C	A	Q	K	BMEI1273	NA
AE008917	1324948	intergenic	C	T	-	-	-	-
AE008917	1325597	nsyn	C	A	G	W	BMEI1274	Lysine efflux permease
AE008917	1330234	nsyn	C	T	T	M	BMEI1282	Predicted aminomethyltransferase related to GcvT
AE008917	1330511	syn	A	T	P	P	BMEI1282	Predicted aminomethyltransferase related to GcvT
AE008917	1332429	nsyn	G	T	T	K	BMEI1285	Recombinational DNA repair protein (RecF pathway)
AE008917	1333034	nsyn	T	A	Q	L	BMEI1286	GTPase
AE008917	1333427	nsyn	C	T	G	E	BMEI1286	GTPase
AE008917	1337726	intergenic	A	C	-	-	-	-

AE008917	1339397	nsyn	T	C	T	A	BMEI1293	Coproporphyrinogen III oxidase and related Fe-S oxidoreductases
AE008917	1341227	nsyn	T	C	I	M	BMEI1294	cAMP-binding proteins - catabolite gene activator and regulatory subunit of cAMP-dependent protein kinases
AE008917	1341279	nsyn	G	A	S	L	BMEI1294	cAMP-binding proteins - catabolite gene activator and regulatory subunit of cAMP-dependent protein kinases
AE008917	1344444	syn	T	G	L	L	BMEI1296	Guanosine polyphosphate pyrophosphohydrolases/synthetases
AE008917	1344509	nsyn	G	C	R	G	BMEI1296	Guanosine polyphosphate pyrophosphohydrolases/synthetases
AE008917	1346306	syn	C	G	A	A	BMEI1299	Uracil-DNA glycosylase
AE008917	1348110	nsyn	T	C	I	V	BMEI1301	Dihydrodipicolinate synthase/N-acetylneuraminate lyase
AE008917	1348435	syn	A	T	A	A	BMEI1301	Dihydrodipicolinate synthase/N-acetylneuraminate lyase
AE008917	1353067	syn	C	G	T	T	BMEI1305	NA
AE008917	1353280	syn	C	T	K	K	BMEI1305	NA
AE008917	1353300	nsyn	C	G	D	H	BMEI1305	NA
AE008917	1353301	syn	A	C	P	P	BMEI1305	NA
AE008917	1353310	syn	G	A	G	G	BMEI1305	NA
AE008917	1353329	nsyn	G	A	T	I	BMEI1305	NA
AE008917	1353347	nsyn	C	A	G	V	BMEI1305	NA
AE008917	1353403	syn	A	G	T	T	BMEI1305	NA
AE008917	1353415	nsyn	C	G	S	A	BMEI1305	NA
AE008917	1353417	nsyn	A	C	S	A	BMEI1305	NA
AE008917	1353791	nsyn	A	G	I	T	BMEI1305	NA
AE008917	1353864	nsyn	G	A	P	S	BMEI1305	NA
AE008917	1354828	syn	C	T	A	A	BMEI1306	NA
AE008917	1354886	nsyn	C	T	P	S	BMEI1306	NA
AE008917	1356684	nsyn	A	G	N	D	BMEI1307	Integrase
AE008917	1357590	nsyn	G	A	S	N	BMEI1308	Histidinol-phosphate/aromatic aminotransferase and cobyric acid decarboxylase
AE008917	1358640	nsyn	C	A	R	S	BMEI1310	Uncharacterized small membrane protein
AE008917	1359437	syn	C	G	A	A	BMEI1311	NA
AE008917	1365288	intergenic	C	T	-	-	-	-
AE008917	1365385	intergenic	A	T	-	-	-	-
AE008917	1365799	nsyn	A	G	T	A	BMEI1319	Uncharacterized protein conserved in bacteria
AE008917	1366098	syn	T	C	F	F	BMEI1319	Uncharacterized protein conserved in bacteria
AE008917	1367896	syn	G	A	V	V	BMEI1320	Dehydrogenases (flavoproteins)
AE008917	1368275	intergenic	C	T	-	-	-	-
AE008917	1373018	nsyn	T	C	S	P	BMEI1324	Aminopeptidase N

AE008917	1374652	intergenic	C	T	-	-	-	-
AE008917	1375192	syn	C	T	I	I	BMEI1325	FOG: PAS/PAC domain
AE008917	1377022	syn	C	T	R	R	BMEI1325	FOG: PAS/PAC domain
AE008917	1377430	intergenic	T	C	-	-	-	-
AE008917	1377804	nsyn	C	T	S	L	BMEI1326	Glycosyltransferase involved in LPS biosynthesis
AE008917	1380462	nsyn	T	G	K	Q	BMEI1327	Glutamine synthetase adenylyltransferase
AE008917	1388626	syn	G	A	G	G	BMEI1332	Cytochrome c biogenesis factor
AE008917	1389989	nsyn	G	A	P	L	BMEI1334	Cytochrome c biogenesis factor
AE008917	1390204	syn	A	G	G	G	BMEI1334	Cytochrome c biogenesis factor
AE008917	1390358	nsyn	C	A	G	V	BMEI1334	Cytochrome c biogenesis factor
AE008917	1391188	nsyn	A	G	F	L	BMEI1335	Surface antigen
AE008917	1393209	nsyn	C	T	A	T	BMEI1337	Response regulators consisting of a CheY-like receiver domain and a winged-helix DNA-binding domain
AE008917	1394408	nsyn	A	G	L	P	BMEI1340	NA
AE008917	1395339	nsyn	C	T	E	K	BMEI1340	NA
AE008917	1397043	nsyn	G	A	P	L	BMEI1341	NA
AE008917	1397696	syn	G	A	A	A	BMEI1341	NA
AE008917	1400726	intergenic	G	A	-	-	-	-
AE008917	1402735	syn	T	C	P	P	BMEI1349	Phage-related protein
AE008917	1405331	intergenic	C	A	-	-	-	-
AE008917	1406857	nsyn	C	A	P	H	BMEI1351	Membrane carboxypeptidase (penicillin-binding protein)
AE008917	1408299	intergenic	G	A	-	-	-	-
AE008917	1409002	nsyn	G	A	S	N	BMEI1354	Uncharacterized protein conserved in bacteria
AE008917	1409838	nsyn	C	T	P	S	BMEI1354	Uncharacterized protein conserved in bacteria
AE008917	1412467	nsyn	A	G	M	T	BMEI1357	Signal transduction histidine kinase
AE008917	1417004	intergenic	G	C	-	-	-	-
AE008917	1417145	intergenic	A	G	-	-	-	-
AE008917	1417260	nsyn	C	T	A	V	BMEI1363	NA
AE008917	1418186	syn	C	T	L	L	BMEI1364	Predicted transcriptional regulator
AE008917	1419666	syn	C	T	T	T	BMEI1365	Protease II
AE008917	1419867	syn	A	G	E	E	BMEI1365	Protease II
AE008917	1423033	nsyn	C	T	R	C	BMEI1368	Predicted hydrolase (HAD superfamily)
AE008917	1423075	nsyn	C	G	L	V	BMEI1368	Predicted hydrolase (HAD superfamily)
AE008917	1424006	intergenic	T	A	-	-	-	-
AE008917	1425454	syn	C	T	L	L	BMEI1370	Predicted ATPase

AE008917	1426373	syn	C	T	G	G	BMEI1371	NA
AE008917	1426594	nsyn	T	C	I	V	BMEI1372	Cyanate permease
AE008917	1427179	syn	G	A	L	L	BMEI1372	Cyanate permease
AE008917	1427320	nsyn	T	C	R	G	BMEI1372	Cyanate permease
AE008917	1427624	syn	A	G	A	A	BMEI1372	Cyanate permease
AE008917	1429961	nsyn	T	G	L	V	BMEI1376	Prophage maintenance system killer protein
AE008917	1431978	intergenic	T	C	-	-	-	-
AE008917	1432919	nsyn	G	A	W	*	BMEI1380	Choline dehydrogenase and related flavoproteins
AE008917	1434147	nsyn	G	A	A	T	BMEI1380	Choline dehydrogenase and related flavoproteins
AE008917	1434426	syn	G	A	Q	Q	BMEI1382	NAD-dependent aldehyde dehydrogenases
AE008917	1435747	nsyn	C	T	P	S	BMEI1382	NAD-dependent aldehyde dehydrogenases
AE008917	1436063	intergenic	T	G	-	-	-	-
AE008917	1437437	intergenic	A	G	-	-	-	-
AE008917	1438284	nsyn	C	A	D	E	BMEI1385	ABC-type sugar transport system, periplasmic component
AE008917	1439177	syn	T	C	I	I	BMEI1386	Sugar (pentulose and hexulose) kinases
AE008917	1444735	syn	G	T	G	G	BMEI1390	ABC-type sugar transport system, periplasmic component
AE008917	1445097	nsyn	C	T	G	S	BMEI1391	Ribose/xylose/arabinose/galactoside ABC-type transport systems, permease components
AE008917	1446754	nsyn	T	C	Q	R	BMEI1392	ABC-type sugar transport system, ATPase component
AE008917	1447484	syn	G	T	R	R	BMEI1392	ABC-type sugar transport system, ATPase component
AE008917	1447642	intergenic	G	A	-	-	-	-
AE008917	1447740	intergenic	T	C	-	-	-	-
AE008917	1448356	syn	C	T	L	L	BMEI1393	Glycosyltransferase
AE008917	1450088	syn	A	G	L	L	BMEI1394	N-acyl-D-glucosamine 2-epimerase
AE008917	1450845	nsyn	G	A	L	F	BMEI1395	Mannose-1-phosphate guanylyltransferase
AE008917	1452527	syn	T	C	R	R	BMEI1396	Phosphomannomutase
AE008917	1453033	nsyn	A	G	F	L	BMEI1396	Phosphomannomutase
AE008917	1453929	intergenic	A	G	-	-	-	-
AE008917	1454642	nsyn	C	G	C	S	BMEI1398	Transposase and inactivated derivatives
AE008917	1454748	syn	C	T	K	K	BMEI1399	Transposase and inactivated derivatives
AE008917	1454753	nsyn	G	A	P	S	BMEI1399	Transposase and inactivated derivatives
AE008917	1454759	nsyn	T	C	T	A	BMEI1399	Transposase and inactivated derivatives
AE008917	1454765	nsyn	A	G	F	L	BMEI1399	Transposase and inactivated derivatives
AE008917	1454842	nsyn	G	A	S	F	BMEI1399	Transposase and inactivated derivatives
AE008917	1454938	nsyn	G	A	P	S	BMEI1400	Transposase and inactivated derivatives

AE008917	1454986	nsyn	G	A	R	C	BMEI1400	Transposase and inactivated derivatives
AE008917	1455022	intergenic	T	C	-	-	-	-
AE008917	1456026	syn	A	G	R	R	BMEI1402	Transposase and inactivated derivatives
AE008917	1458095	nsyn	G	T	R	S	BMEI1405	Transposase and inactivated derivatives
AE008917	1458240	intergenic	A	G	-	-	-	-
AE008917	1458281	nsyn	A	G	L	S	BMEI1406	Transposase and inactivated derivatives
AE008917	1458319	syn	A	G	C	C	BMEI1406	Transposase and inactivated derivatives
AE008917	1458409	syn	C	T	A	A	BMEI1406	Transposase and inactivated derivatives
AE008917	1458556	nsyn	C	A	G	V	BMEI1407	Transposase and inactivated derivatives
AE008917	1458594	syn	T	C	K	K	BMEI1407	Transposase and inactivated derivatives
AE008917	1458636	syn	G	A	D	D	BMEI1407	Transposase and inactivated derivatives
AE008917	1458714	syn	G	T	R	R	BMEI1407	Transposase and inactivated derivatives
AE008917	1458807	syn	G	A	D	D	BMEI1407	Transposase and inactivated derivatives
AE008917	1458917	intergenic	C	A	-	-	-	-
AE008917	1458931	intergenic	T	C	-	-	-	-
AE008917	1458954	intergenic	G	A	-	-	-	-
AE008917	1458956	intergenic	A	G	-	-	-	-
AE008917	1459604	syn	A	G	G	G	BMEI1409	NA
AE008917	1460470	nsyn	G	A	P	S	BMEI1412	Transposase and inactivated derivatives
AE008917	1464166	nsyn	A	C	N	T	BMEI1416	ABC-type polysaccharide/polyol phosphate transport system, ATPase component
AE008917	1466635	nsyn	G	T	A	D	BMEI1419	NA
AE008917	1467865	intergenic	T	C	-	-	-	-
AE008917	1468629	nsyn	T	C	T	A	BMEI1423	Transposase and inactivated derivatives
AE008917	1469842	intergenic	G	A	-	-	-	-
AE008917	1471327	nsyn	T	G	L	R	BMEI1426	UDP-N-acetylmuramyl pentapeptide phosphotransferase/ UDP-N-acetylglucosamine-1-phosphate transferase
AE008917	1471368	nsyn	T	C	K	E	BMEI1427	Predicted nucleoside-diphosphate sugar epimerases
AE008917	1471849	syn	T	C	K	K	BMEI1427	Predicted nucleoside-diphosphate sugar epimerases
AE008917	1474825	syn	A	G	G	G	BMEI1429	Transthyretin-like protein
AE008917	1475199	syn	G	A	A	A	BMEI1430	Ureidoglycolate hydrolase
AE008917	1476229	nsyn	G	A	A	V	BMEI1431	Uncharacterized conserved protein
AE008917	1476817	syn	G	A	A	A	BMEI1432	Zn-dependent hydrolases, including glyoxylases
AE008917	1481105	syn	G	C	R	R	BMEI1436	Phosphoenolpyruvate synthase/pyruvate phosphate dikinase
AE008917	1481825	syn	A	G	V	V	BMEI1436	Phosphoenolpyruvate synthase/pyruvate phosphate dikinase
AE008917	1482134	syn	G	A	N	N	BMEI1436	Phosphoenolpyruvate synthase/pyruvate phosphate dikinase

AE008917	1482308	syn	A	G	D	D	BMEI1436	Phosphoenolpyruvate synthase/pyruvate phosphate dikinase
AE008917	1488639	intergenic	A	G	-	-	-	-
AE008917	1492723	intergenic	T	G	-	-	-	-
AE008917	1497949	nsyn	G	C	R	G	BMEI1449	NA
AE008917	1500062	syn	G	A	E	E	BMEI1450	Threonine synthase
AE008917	1500616	syn	G	C	T	T	BMEI1451	Predicted Zn-dependent peptidases
AE008917	1501464	nsyn	C	A	T	K	BMEI1451	Predicted Zn-dependent peptidases
AE008917	1501504	intergenic	T	C	-	-	-	-
AE008917	1502225	nsyn	C	T	T	M	BMEI1452	Acetyltransferases, including N-acetylases of ribosomal proteins
AE008917	1503255	nsyn	G	A	A	T	BMEI1453	FOG: PAS/PAC domain
AE008917	1503971	syn	A	G	V	V	BMEI1453	FOG: PAS/PAC domain
AE008917	1504742	syn	C	T	L	L	BMEI1453	FOG: PAS/PAC domain
AE008917	1507715	syn	A	G	G	G	BMEI1457	Ribonuclease HI
AE008917	1508014	nsyn	G	A	G	A	BMEI1458	Putative homoserine kinase type II (protein kinase fold)
AE008917	1508015	nsyn	C	G	G	A	BMEI1458	Putative homoserine kinase type II (protein kinase fold)
AE008917	1510528	nsyn	C	T	R	H	BMEI1460	Uncharacterized conserved protein
AE008917	1512338	intergenic	G	T	-	-	-	-
AE008917	1515650	nsyn	G	A	T	M	BMEI1465	Heme/copper-type cytochrome/quinol oxidases, subunit 1
AE008917	1518340	nsyn	G	A	S	N	BMEI1468	Predicted Zn-dependent proteases and their inactivated homologs
AE008917	1521458	intergenic	G	A	-	-	-	-
AE008917	1523763	syn	G	A	R	R	BMEI1473	3-oxoacyl-(acyl-carrier-protein) synthase
AE008917	1524776	nsyn	A	T	D	V	BMEI1476	NA
AE008917	1526104	nsyn	A	G	S	P	BMEI1478	(acyl-carrier-protein) S-malonyltransferase
AE008917	1527443	nsyn	A	C	S	R	BMEI1480	Ribosomal protein S6
AE008917	1527915	intergenic	T	C	-	-	-	-
AE008917	1528805	nsyn	A	G	N	S	BMEI1482	NA
AE008917	1528852	nsyn	C	T	R	C	BMEI1482	NA
AE008917	1529282	syn	A	T	A	A	BMEI1483	Ribosomal protein L9
AE008917	1532908	syn	T	C	F	F	BMEI1485	Replicative DNA helicase
AE008917	1534229	nsyn	C	G	P	A	BMEI1486	Predicted ATP-dependent serine protease
AE008917	1538089	nsyn	A	G	F	L	BMEI1490	Phosphatidylserine synthase
AE008917	1539210	nsyn	C	A	M	I	BMEI1491	Phosphatidylserine decarboxylase
AE008917	1540652	syn	T	C	E	E	BMEI1492	ABC-type transport system involved in Fe-S cluster assembly, permease and ATPase components
AE008917	1542074	nsyn	C	T	D	N	BMEI1493	Uncharacterized protein containing LysM domain

AE008917	1542135	syn	A	G	G	G	BMEI1493	Uncharacterized protein containing LysM domain
AE008917	1543435	nsyn	T	C	I	T	BMEI1495	Predicted Rossmann fold nucleotide-binding protein
AE008917	1544299	syn	G	A	G	G	BMEI1496	SAM-dependent methyltransferases related to tRNA (uracil-5-)-methyltransferase
AE008917	1544622	nsyn	T	C	I	V	BMEI1496	SAM-dependent methyltransferases related to tRNA (uracil-5-)-methyltransferase
AE008917	1545684	syn	G	A	A	A	BMEI1497	Predicted rRNA methylase
AE008917	1547474	syn	C	T	A	A	BMEI1498	Deoxyxylulose-5-phosphate synthase
AE008917	1548359	nsyn	T	C	K	E	BMEI1499	Pirin-related protein
AE008917	1548710	nsyn	C	T	V	I	BMEI1499	Pirin-related protein
AE008917	1550203	syn	C	T	H	H	BMEI1501	Predicted membrane protein
AE008917	1550229	nsyn	G	A	G	D	BMEI1501	Predicted membrane protein
AE008917	1552698	syn	T	C	Q	Q	BMEI1505	3,4-dihydroxy-2-butanone 4-phosphate synthase
AE008917	1553825	nsyn	C	T	D	N	BMEI1506	Chorismate synthase
AE008917	1554087	syn	G	A	Y	Y	BMEI1506	Chorismate synthase
AE008917	1555856	nsyn	G	C	L	V	BMEI1508	Uncharacterized protein conserved in bacteria
AE008917	1555918	nsyn	G	A	P	L	BMEI1508	Uncharacterized protein conserved in bacteria
AE008917	1556707	intergenic	C	T	-	-	-	-
AE008917	1556797	intergenic	C	A	-	-	-	-
AE008917	1559401	syn	T	C	P	P	BMEI1513	DnaJ-class molecular chaperone
AE008917	1559558	nsyn	C	T	G	D	BMEI1513	DnaJ-class molecular chaperone
AE008917	1559653	syn	T	G	G	G	BMEI1513	DnaJ-class molecular chaperone
AE008917	1560383	nsyn	G	A	P	S	BMEI1514	NA
AE008917	1560690	intergenic	C	T	-	-	-	-
AE008917	1561687	nsyn	A	G	V	A	BMEI1518	4-hydroxybenzoate polyprenyltransferase and related prenyltransferases
AE008917	1562548	intergenic	G	A	-	-	-	-
AE008917	1562734	nsyn	T	C	L	P	BMEI1519	Phosphoribosylamine-glycine ligase
AE008917	1563558	nsyn	G	A	V	I	BMEI1519	Phosphoribosylamine-glycine ligase
AE008917	1568062	intergenic	T	C	-	-	-	-
AE008917	1568696	nsyn	C	T	P	S	BMEI1524	NA
AE008917	1572306	syn	C	T	G	G	BMEI1529	Glycyl-tRNA synthetase, beta subunit
AE008917	1577419	intergenic	G	T	-	-	-	-
AE008917	1581211	nsyn	G	A	G	S	BMEI1535	Na+/H+ antiporter
AE008917	1581827	nsyn	C	T	A	V	BMEI1535	Na+/H+ antiporter
AE008917	1581875	nsyn	C	T	A	V	BMEI1535	Na+/H+ antiporter

AE008917	1582372	nsyn	C	T	P	S	BMEI1535	Na+/H+ antiporter
AE008917	1583176	nsyn	G	A	A	T	BMEI1537	4-diphosphocytidyl-2C-methyl-D-erythritol 2-phosphate synthase
AE008917	1583977	nsyn	A	G	K	E	BMEI1537	4-diphosphocytidyl-2C-methyl-D-erythritol 2-phosphate synthase
AE008917	1584985	nsyn	G	C	A	G	BMEI1540	Glycosyltransferases involved in cell wall biogenesis
AE008917	1586588	intergenic	A	G	-	-	-	-
AE008917	1586709	intergenic	A	C	-	-	-	-
AE008917	1588358	syn	C	G	L	L	BMEI1544	F0F1-type ATP synthase, subunit b
AE008917	1588405	nsyn	T	G	T	P	BMEI1544	F0F1-type ATP synthase, subunit b
AE008917	1588713	intergenic	A	G	-	-	-	-
AE008917	1590194	nsyn	C	T	M	I	BMEI1547	Uncharacterized protein conserved in bacteria
AE008917	1591611	nsyn	A	G	I	T	BMEI1548	Cell wall hydrolyses involved in spore germination
AE008917	1592149	intergenic	A	C	-	-	-	-
AE008917	1593198	nsyn	G	C	F	L	BMEI1549	Molecular chaperone
AE008917	1593642	syn	A	G	D	D	BMEI1549	Molecular chaperone
AE008917	1597280	intergenic	A	G	-	-	-	-
AE008917	1597476	syn	T	C	L	L	BMEI1553	ABC-type long-chain fatty acid transport system, fused permease and ATPase components
AE008917	1597483	nsyn	T	C	D	G	BMEI1553	ABC-type long-chain fatty acid transport system, fused permease and ATPase components
AE008917	1598689	intergenic	A	G	-	-	-	-
AE008917	1599366	nsyn	T	C	T	A	BMEI1555	Permeases of the major facilitator superfamily
AE008917	1600943	syn	G	T	I	I	BMEI1556	Predicted membrane protein
AE008917	1601345	intergenic	T	C	-	-	-	-
AE008917	1602799	syn	A	G	A	A	BMEI1558	Adenosine deaminase
AE008917	1603161	intergenic	T	C	-	-	-	-
AE008917	1605098	nsyn	G	T	R	S	BMEI1561	Protein-disulfide isomerase
AE008917	1605349	nsyn	G	T	T	K	BMEI1561	Protein-disulfide isomerase
AE008917	1605352	nsyn	G	A	A	V	BMEI1561	Protein-disulfide isomerase
AE008917	1605577	nsyn	A	C	V	G	BMEI1562	NA
AE008917	1613421	nsyn	C	A	A	D	BMEI1569	Cation transport ATPase
AE008917	1614824	syn	C	T	T	T	BMEI1570	Putative glycerate kinase
AE008917	1615184	syn	G	A	S	S	BMEI1570	Putative glycerate kinase
AE008917	1616050	nsyn	A	G	M	T	BMEI1571	Cytosine deaminase and related metal-dependent hydrolases
AE008917	1617460	nsyn	G	A	R	C	BMEI1572	Predicted membrane protein
AE008917	1617951	nsyn	T	C	N	S	BMEI1572	Predicted membrane protein
AE008917	1619410	intergenic	G	A	-	-	-	-

AE008917	1619784	nsyn	G	A	P	S	BMEI1574	Xanthine and CO dehydrogenases maturation factor, XdhC/CoxF family
AE008917	1621129	nsyn	T	G	T	P	BMEI1575	Xanthine dehydrogenase, molybdopterin-binding subunit B
AE008917	1622418	nsyn	G	T	P	Q	BMEI1575	Xanthine dehydrogenase, molybdopterin-binding subunit B
AE008917	1624396	intergenic	G	A	-	-	-	-
AE008917	1625444	syn	A	G	E	E	BMEI1577	Predicted xylanase/chitin deacetylase
AE008917	1625826	nsyn	C	A	P	T	BMEI1577	Predicted xylanase/chitin deacetylase
AE008917	1626841	nsyn	T	G	L	W	BMEI1578	Uncharacterized protein, possibly involved in glyoxylate utilization
AE008917	1627138	intergenic	A	G	-	-	-	-
AE008917	1627585	nsyn	A	G	F	L	BMEI1579	TRAP-type mannitol/chloroaromatic compound transport system, periplasmic component
AE008917	1627737	nsyn	C	A	R	L	BMEI1579	TRAP-type mannitol/chloroaromatic compound transport system, periplasmic component
AE008917	1628835	syn	G	A	V	V	BMEI1580	TRAP-type mannitol/chloroaromatic compound transport system, large permease component
AE008917	1630265	nsyn	A	C	V	G	BMEI1581	TRAP-type mannitol/chloroaromatic compound transport system, small permease component
AE008917	1632807	intergenic	G	T	-	-	-	-
AE008917	1634003	nsyn	C	T	A	T	BMEI1585	Na+-driven multidrug efflux pump
AE008917	1635152	intergenic	C	T	-	-	-	-
AE008917	1635488	nsyn	A	G	K	E	BMEI1586	Proline racemase
AE008917	1636076	nsyn	T	C	F	L	BMEI1586	Proline racemase
AE008917	1638548	syn	A	G	S	S	BMEI1589	Diaminopimelate decarboxylase
AE008917	1644279	syn	C	T	R	R	BMEI1595	NA
AE008917	1644364	nsyn	G	T	S	Y	BMEI1595	NA
AE008917	1645398	syn	G	A	S	S	BMEI1597	Putative effector of murein hydrolase LrgA
AE008917	1646959	intergenic	A	G	-	-	-	-
AE008917	1647420	syn	G	A	P	P	BMEI1601	NA
AE008917	1647736	intergenic	C	A	-	-	-	-
AE008917	1648676	syn	C	A	P	P	BMEI1603	Predicted xylanase/chitin deacetylase
AE008917	1651582	intergenic	T	C	-	-	-	-
AE008917	1652069	syn	T	G	S	S	BMEI1606	Na+/proline symporter
AE008917	1652091	nsyn	G	A	D	N	BMEI1606	Na+/proline symporter
AE008917	1654981	nsyn	C	G	T	S	BMEI1606	Na+/proline symporter
AE008917	1654982	nsyn	G	C	T	S	BMEI1606	Na+/proline symporter
AE008917	1657924	nsyn	A	G	S	G	BMEI1610	Uncharacterized protein conserved in bacteria
AE008917	1658312	syn	A	G	V	V	BMEI1611	Dihydroorotate dehydrogenase

AE008917	1661011	syn	T	C	Q	Q	BMEI1613	Uncharacterized protein with SCP/PR1 domains
AE008917	1661149	syn	T	C	K	K	BMEI1613	Uncharacterized protein with SCP/PR1 domains
AE008917	1663836	syn	C	T	A	A	BMEI1616	Deoxycytidine deaminase
AE008917	1667296	nsyn	G	C	A	G	BMEI1619	Disulfide bond chaperones of the HSP33 family
AE008917	1667458	syn	G	A	G	G	BMEI1620	Ornithine carbamoyltransferase
AE008917	1667929	syn	A	G	R	R	BMEI1620	Ornithine carbamoyltransferase
AE008917	1671572	syn	G	T	A	A	BMEI1624	Signal transduction histidine kinase
AE008917	1672348	nsyn	G	A	G	D	BMEI1625	Uncharacterized conserved protein
AE008917	1673468	nsyn	G	T	H	Q	BMEI1626	Glycosyltransferases, probably involved in cell wall biogenesis
AE008917	1673726	syn	G	A	V	V	BMEI1626	Glycosyltransferases, probably involved in cell wall biogenesis
AE008917	1674899	intergenic	A	G	-	-	-	-
AE008917	1675168	intergenic	T	C	-	-	-	-
AE008917	1676131	nsyn	A	C	W	G	BMEI1628	NA
AE008917	1676924	nsyn	A	G	T	A	BMEI1629	Cation/multidrug efflux pump
AE008917	1676926	nsyn	T	C	T	A	BMEI1629	Cation/multidrug efflux pump
AE008917	1677162	nsyn	A	G	V	A	BMEI1629	Cation/multidrug efflux pump
AE008917	1677814	nsyn	A	T	Y	N	BMEI1629	Cation/multidrug efflux pump
AE008917	1678499	syn	T	C	L	L	BMEI1629	Cation/multidrug efflux pump
AE008917	1679131	nsyn	T	C	N	D	BMEI1629	Cation/multidrug efflux pump
AE008917	1680667	syn	G	A	R	R	BMEI1630	Membrane-fusion protein
AE008917	1683387	syn	G	A	T	T	BMEI1632	Acyl-CoA synthetases (AMP-forming)/AMP-acid ligases II
AE008917	1686586	syn	T	C	A	A	BMEI1635	Predicted deacylase
AE008917	1690553	nsyn	C	G	D	E	BMEI1638	NADPH-dependent glutamate synthase beta chain and related oxidoreductases
AE008917	1691019	nsyn	G	A	G	S	BMEI1639	Dihydroorotate dehydrogenase
AE008917	1697706	nsyn	C	A	R	L	BMEI1645	Cation/multidrug efflux pump
AE008917	1701827	syn	T	C	L	L	BMEI1648	Signal transduction histidine kinase
AE008917	1704141	syn	A	G	L	L	BMEI1650	Urease accessory protein UreF
AE008917	1706536	intergenic	G	A	-	-	-	-
AE008917	1707366	intergenic	C	A	-	-	-	-
AE008917	1709684	intergenic	T	C	-	-	-	-
AE008917	1715069	syn	C	T	E	E	BMEI1664	Integrase
AE008917	1716422	nsyn	G	T	D	E	BMEI1664	Integrase
AE008917	1716431	syn	G	A	D	D	BMEI1664	Integrase
AE008917	1717515	syn	C	G	V	V	BMEI1666	UDP-N-acetylglucosamine enolpyruvyl transferase

AE008917	1722834	nsyn	G	A	A	T	BMEI1673	Uncharacterized protein conserved in bacteria
AE008917	1724787	syn	A	G	K	K	BMEI1674	NA
AE008917	1724862	intergenic	C	T	-	-	-	-
AE008917	1725587	nsyn	T	C	H	R	BMEI1676	NA
AE008917	1726078	syn	A	G	F	F	BMEI1676	NA
AE008917	1726603	syn	T	C	T	T	BMEI1677	NA
AE008917	1727610	intergenic	C	T	-	-	-	-
AE008917	1728133	nsyn	C	T	R	W	BMEI1681	NA
AE008917	1728972	intergenic	C	A	-	-	-	-
AE008917	1729390	nsyn	G	A	T	M	BMEI1683	Predicted Zn peptidase
AE008917	1729910	syn	A	T	A	A	BMEI1684	NA
AE008917	1730758	nsyn	A	G	V	A	BMEI1686	NA
AE008917	1730971	nsyn	G	A	A	V	BMEI1686	NA
AE008917	1731548	syn	C	T	V	V	BMEI1688	NA
AE008917	1732720	intergenic	T	C	-	-	-	-
AE008917	1732756	intergenic	C	T	-	-	-	-
AE008917	1733198	syn	A	C	A	A	BMEI1691	NA
AE008917	1734166	nsyn	C	T	A	T	BMEI1691	NA
AE008917	1735065	nsyn	A	G	W	R	BMEI1692	Muramidase (flagellum-specific)
AE008917	1735266	nsyn	G	A	Q	*	BMEI1692	Muramidase (flagellum-specific)
AE008917	1735959	nsyn	C	A	A	S	BMEI1692	Muramidase (flagellum-specific)
AE008917	1736934	intergenic	A	G	-	-	-	-
AE008917	1737730	nsyn	C	T	E	K	BMEI1694	NA
AE008917	1738004	nsyn	T	G	E	D	BMEI1694	NA
AE008917	1738191	nsyn	A	G	F	S	BMEI1694	NA
AE008917	1739153	nsyn	C	T	W	*	BMEI1696	NA
AE008917	1739334	nsyn	A	G	C	R	BMEI1696	NA
AE008917	1740335	nsyn	A	G	F	S	BMEI1696	NA
AE008917	1740424	syn	T	C	L	L	BMEI1696	NA
AE008917	1742713	intergenic	T	G	-	-	-	-
AE008917	1743064	syn	A	G	S	S	BMEI1702	Integrase
AE008917	1744650	nsyn	C	G	S	T	BMEI1704	NA
AE008917	1744888	nsyn	G	A	R	*	BMEI1704	NA
AE008917	1746194	syn	A	G	A	A	BMEI1707	L-alanine-DL-glutamate epimerase and related enzymes of enolase superfamily

AE008917	1747494	syn	T	C	P	P	BMEI1708	2-keto-4-pentenoate hydratase/2-oxohepta-3-ene-1,7-dioic acid hydratase (catechol pathway)
AE008917	1747656	syn	G	C	V	V	BMEI1708	2-keto-4-pentenoate hydratase/2-oxohepta-3-ene-1,7-dioic acid hydratase (catechol pathway)
AE008917	1748255	nsyn	G	A	L	F	BMEI1708	2-keto-4-pentenoate hydratase/2-oxohepta-3-ene-1,7-dioic acid hydratase (catechol pathway)
AE008917	1748526	nsyn	T	C	S	G	BMEI1709	Dehydrogenases with different specificities (related to short-chain alcohol dehydrogenases)
AE008917	1748945	nsyn	A	G	V	A	BMEI1709	Dehydrogenases with different specificities (related to short-chain alcohol dehydrogenases)
AE008917	1748986	syn	G	C	A	A	BMEI1709	Dehydrogenases with different specificities (related to short-chain alcohol dehydrogenases)
AE008917	1750937	nsyn	C	T	A	T	BMEI1712	L-alanine-DL-glutamate epimerase and related enzymes of enolase superfamily
AE008917	1751614	nsyn	C	T	R	K	BMEI1712	L-alanine-DL-glutamate epimerase and related enzymes of enolase superfamily
AE008917	1751732	syn	G	A	A	A	BMEI1713	ABC-type sugar transport systems, ATPase components
AE008917	1753863	nsyn	T	A	E	V	BMEI1715	ABC-type sugar transport systems, permease components
AE008917	1757256	nsyn	G	T	D	Y	BMEI1718	Predicted metal-dependent hydrolase of the TIM-barrel fold
AE008917	1765586	intergenic	A	C	-	-	-	-
AE008917	1765591	intergenic	T	G	-	-	-	-
AE008917	1765592	intergenic	C	G	-	-	-	-
AE008917	1769130	nsyn	A	G	T	A	BMEI1728	ABC-type proline/glycine betaine transport systems, permease component
AE008917	1769632	intergenic	T	C	-	-	-	-
AE008917	1769705	intergenic	T	G	-	-	-	-
AE008917	1769731	nsyn	G	T	G	V	BMEI1729	Predicted transcriptional regulators
AE008917	1771390	nsyn	A	C	V	G	BMEI1730	Cation transport ATPase
AE008917	1772361	syn	G	A	F	F	BMEI1730	Cation transport ATPase
AE008917	1772815	nsyn	C	A	D	Y	BMEI1731	FOG: EAL domain
AE008917	1774466	syn	A	G	D	D	BMEI1731	FOG: EAL domain
AE008917	1774645	intergenic	T	C	-	-	-	-
AE008917	1778203	syn	T	C	R	R	BMEI1736	Thiamine monophosphate synthase
AE008917	1779295	nsyn	A	G	K	E	BMEI1737	ABC-type nitrate/sulfonate/bicarbonate transport systems, periplasmic components
AE008917	1779663	nsyn	G	T	A	S	BMEI1738	Putative transcription activator

AE008917	1780642	nsyn	A	C	E	D	BMEI1739	ABC-type nitrate/sulfonate/bicarbonate transport system, permease component
AE008917	1780666	syn	G	A	G	G	BMEI1739	ABC-type nitrate/sulfonate/bicarbonate transport system, permease component
AE008917	1781826	syn	G	A	V	V	BMEI1740	NAD-dependent aldehyde dehydrogenases
AE008917	1782296	nsyn	C	T	A	T	BMEI1740	NAD-dependent aldehyde dehydrogenases
AE008917	1784730	syn	C	T	R	R	BMEI1743	ABC-type multidrug transport system, ATPase and permease components
AE008917	1785332	syn	A	C	L	L	BMEI1744	Transcriptional regulators
AE008917	1785600	nsyn	G	A	S	F	BMEI1744	Transcriptional regulators
AE008917	1788075	nsyn	A	C	H	Q	BMEI1747	NAD-dependent aldehyde dehydrogenases
AE008917	1788361	nsyn	T	C	N	G	BMEI1747	NAD-dependent aldehyde dehydrogenases
AE008917	1788362	nsyn	T	C	N	G	BMEI1747	NAD-dependent aldehyde dehydrogenases
AE008917	1788488	nsyn	T	A	I	F	BMEI1747	NAD-dependent aldehyde dehydrogenases
AE008917	1788834	syn	C	T	V	V	BMEI1747	NAD-dependent aldehyde dehydrogenases
AE008917	1790025	nsyn	A	C	F	V	BMEI1748	Transcriptional activator of acetoin/glycerol metabolism
AE008917	1791170	nsyn	A	G	W	R	BMEI1749	Glycerol-3-phosphate dehydrogenase
AE008917	1791595	nsyn	T	C	Q	R	BMEI1749	Glycerol-3-phosphate dehydrogenase
AE008917	1792553	nsyn	T	C	T	A	BMEI1750	Transcriptional regulators of sugar metabolism
AE008917	1799548	nsyn	G	C	A	P	BMEI1756	NA
AE008917	1799550	nsyn	T	C	A	P	BMEI1756	NA
AE008917	1799553	syn	T	A	P	P	BMEI1756	NA
AE008917	1799554	nsyn	G	A	E	K	BMEI1756	NA
AE008917	1799558	nsyn	C	A	T	K	BMEI1756	NA
AE008917	1799561	nsyn	A	G	E	G	BMEI1756	NA
AE008917	1799565	syn	G	C	R	R	BMEI1756	NA
AE008917	1801726	nsyn	T	C	E	G	BMEI1758	DNA-binding HTH domain-containing proteins
AE008917	1802519	syn	G	A	A	A	BMEI1759	Methionine synthase I, cobalamin-binding domain
AE008917	1802970	nsyn	G	A	P	L	BMEI1759	Methionine synthase I, cobalamin-binding domain
AE008917	1803853	nsyn	G	A	R	W	BMEI1759	Methionine synthase I, cobalamin-binding domain
AE008917	1805371	nsyn	C	G	V	L	BMEI1759	Methionine synthase I, cobalamin-binding domain
AE008917	1811134	nsyn	G	A	A	V	BMEI1767	NA
AE008917	1811480	syn	A	G	I	I	BMEI1768	Uroporphyrinogen-III methylase
AE008917	1813226	syn	G	T	R	R	BMEI1769	Predicted endonuclease distantly related to archaeal Holliday junction resolvase
AE008917	1813529	nsyn	C	A	R	L	BMEI1770	Predicted methyltransferases

AE008917	1818946	intergenic	T	C	-	-	-	-
AE008917	1820226	syn	T	C	V	V	BMEI1778	Predicted membrane protein
AE008917	1822241	nsyn	A	G	T	A	BMEI1781	Uncharacterized protein involved in cysteine biosynthesis
AE008917	1823263	syn	A	G	R	R	BMEI1783	Integral membrane protein
AE008917	1823516	nsyn	C	T	R	C	BMEI1783	Integral membrane protein
AE008917	1826435	syn	A	G	Q	Q	BMEI1788	NA
AE008917	1828107	nsyn	T	C	N	D	BMEI1789	DNA-directed RNA polymerase specialized sigma subunit, sigma54 homolog
AE008917	1828577	intergenic	G	A	-	-	-	-
AE008917	1829259	nsyn	G	A	A	V	BMEI1790	ABC-type (unclassified) transport system, ATPase component
AE008917	1829347	nsyn	C	T	G	S	BMEI1790	ABC-type (unclassified) transport system, ATPase component
AE008917	1832763	nsyn	G	A	G	S	BMEI1794	Bacterial nucleoid DNA-binding protein
AE008917	1834550	nsyn	C	T	P	S	BMEI1798	rRNA methylases
AE008917	1838788	nsyn	C	T	G	S	BMEI1801	Mismatch repair ATPase (MutS family)
AE008917	1841024	nsyn	A	G	N	D	BMEI1802	Malic enzyme
AE008917	1843097	nsyn	T	C	Y	H	BMEI1804	UTP:GlnB (protein PII) uridylyltransferase
AE008917	1843297	syn	G	C	P	P	BMEI1804	UTP:GlnB (protein PII) uridylyltransferase
AE008917	1845214	syn	T	C	V	V	BMEI1804	UTP:GlnB (protein PII) uridylyltransferase
AE008917	1845521	syn	A	G	A	A	BMEI1805	Uncharacterized membrane protein, putative virulence factor
AE008917	1845783	nsyn	G	A	A	T	BMEI1805	Uncharacterized membrane protein, putative virulence factor
AE008917	1848098	syn	A	G	S	S	BMEI1806	Tryptophanyl-tRNA synthetase
AE008917	1849413	nsyn	G	A	V	I	BMEI1808	Thioredoxin-like proteins and domains
AE008917	1849612	nsyn	T	C	I	V	BMEI1809	Uncharacterized protein conserved in bacteria
AE008917	1852113	syn	T	C	*	*	BMEI1814	Transposase and inactivated derivatives
AE008917	1852151	syn	A	G	G	G	BMEI1814	Transposase and inactivated derivatives
AE008917	1852321	nsyn	C	T	D	N	BMEI1814	Transposase and inactivated derivatives
AE008917	1852361	syn	C	T	A	A	BMEI1814	Transposase and inactivated derivatives
AE008917	1852390	nsyn	C	T	E	K	BMEI1814	Transposase and inactivated derivatives
AE008917	1852391	syn	T	C	L	L	BMEI1814	Transposase and inactivated derivatives
AE008917	1853626	syn	C	T	Q	Q	BMEI1816	Signal transduction histidine kinase
AE008917	1853789	nsyn	C	G	R	P	BMEI1816	Signal transduction histidine kinase
AE008917	1856185	nsyn	T	C	Q	R	BMEI1818	HrpA-like helicases
AE008917	1856702	nsyn	G	C	L	V	BMEI1818	HrpA-like helicases
AE008917	1856912	nsyn	A	G	W	R	BMEI1818	HrpA-like helicases
AE008917	1856952	syn	A	G	R	R	BMEI1818	HrpA-like helicases

AE008917	1857548	nsyn	C	T	A	V	BMEI1819	Zn-dependent alcohol dehydrogenases, class III
AE008917	1857817	nsyn	A	G	I	V	BMEI1819	Zn-dependent alcohol dehydrogenases, class III
AE008917	1858831	nsyn	T	C	F	S	BMEI1821	Predicted membrane protein
AE008917	1859197	nsyn	G	A	W	*	BMEI1821	Predicted membrane protein
AE008917	1859446	nsyn	C	T	A	V	BMEI1821	Predicted membrane protein
AE008917	1860009	syn	C	A	G	G	BMEI1822	Predicted esterase
AE008917	1863374	nsyn	C	T	A	T	BMEI1824	Superfamily II DNA and RNA helicases
AE008917	1863597	syn	G	A	V	V	BMEI1824	Superfamily II DNA and RNA helicases
AE008917	1865846	nsyn	T	C	I	V	BMEI1825	DNA polymerase I - 3'-5' exonuclease and polymerase domains
AE008917	1866622	nsyn	C	T	G	D	BMEI1825	DNA polymerase I - 3'-5' exonuclease and polymerase domains
AE008917	1867806	syn	G	A	T	T	BMEI1825	DNA polymerase I - 3'-5' exonuclease and polymerase domains
AE008917	1872207	nsyn	A	C	N	H	BMEI1830	Opacity protein and related surface antigens
AE008917	1872455	syn	G	A	Q	Q	BMEI1830	Opacity protein and related surface antigens
AE008917	1877772	nsyn	T	C	L	S	BMEI1834	SAM-dependent methyltransferases
AE008917	1877836	nsyn	C	G	N	K	BMEI1834	SAM-dependent methyltransferases
AE008917	1877896	intergenic	G	A	-	-	-	-
AE008917	1877955	intergenic	G	A	-	-	-	-
AE008917	1879833	nsyn	C	A	C	F	BMEI1836	Nicotinic acid phosphoribosyltransferase
AE008917	1880170	intergenic	C	A	-	-	-	-
AE008917	1881304	syn	G	C	S	S	BMEI1837	Cellobiose phosphorylase
AE008917	1882261	nsyn	C	A	E	D	BMEI1837	Cellobiose phosphorylase
AE008917	1883865	nsyn	C	T	A	T	BMEI1837	Cellobiose phosphorylase
AE008917	1884223	syn	G	A	D	D	BMEI1837	Cellobiose phosphorylase
AE008917	1885079	nsyn	G	C	P	R	BMEI1837	Cellobiose phosphorylase
AE008917	1885081	syn	A	C	L	L	BMEI1837	Cellobiose phosphorylase
AE008917	1885086	nsyn	C	G	G	R	BMEI1837	Cellobiose phosphorylase
AE008917	1885087	nsyn	G	C	D	E	BMEI1837	Cellobiose phosphorylase
AE008917	1885267	syn	A	G	F	F	BMEI1837	Cellobiose phosphorylase
AE008917	1886077	syn	T	C	A	A	BMEI1837	Cellobiose phosphorylase
AE008917	1886305	syn	G	A	F	F	BMEI1837	Cellobiose phosphorylase
AE008917	1889025	intergenic	A	G	-	-	-	-
AE008917	1889288	syn	G	A	G	G	BMEI1838	ABC-type sulfate/molybdate transport systems, ATPase component
AE008917	1892175	intergenic	A	G	-	-	-	-
AE008917	1892987	nsyn	G	T	S	R	BMEI1841	ABC-type sulfate transport system, periplasmic component
AE008917	1895311	syn	T	C	E	E	BMEI1844	NA

AE008917	1895452	syn	T	C	A	A	BMEI1844	NA
AE008917	1895791	intergenic	C	T	-	-	-	-
AE008917	1897315	nsyn	T	C	V	A	BMEI1847	NA
AE008917	1899148	nsyn	A	G	H	R	BMEI1848	Dihydroxyacid dehydratase/phosphogluconate dehydratase
AE008917	1902014	nsyn	C	T	V	I	BMEI1852	ABC-type transport system involved in cytochrome c biogenesis, permease component
AE008917	1903856	syn	A	G	Q	Q	BMEI1855	Aconitase A
AE008917	1904771	syn	T	C	L	L	BMEI1855	Aconitase A
AE008917	1906811	syn	T	C	I	I	BMEI1856	Uncharacterized secreted protein
AE008917	1907355	intergenic	C	T	-	-	-	-
AE008917	1908032	nsyn	C	T	G	E	BMEI1858	Sortase and related acyltransferases
AE008917	1908520	intergenic	T	C	-	-	-	-
AE008917	1909736	nsyn	G	A	R	W	BMEI1860	Predicted ABC-type transport system involved in lysophospholipase L1 biosynthesis, permease component
AE008917	1910046	syn	T	C	L	L	BMEI1860	Predicted ABC-type transport system involved in lysophospholipase L1 biosynthesis, permease component
AE008917	1910129	nsyn	C	T	D	N	BMEI1860	Predicted ABC-type transport system involved in lysophospholipase L1 biosynthesis, permease component
AE008917	1911443	nsyn	C	T	A	T	BMEI1860	Predicted ABC-type transport system involved in lysophospholipase L1 biosynthesis, permease component
AE008917	1911980	nsyn	C	A	A	S	BMEI1860	Predicted ABC-type transport system involved in lysophospholipase L1 biosynthesis, permease component
AE008917	1911983	nsyn	C	G	G	R	BMEI1860	Predicted ABC-type transport system involved in lysophospholipase L1 biosynthesis, permease component
AE008917	1913777	intergenic	G	A	-	-	-	-
AE008917	1915062	intergenic	T	C	-	-	-	-
AE008917	1917527	nsyn	G	A	G	S	BMEI1867	Predicted Fe-S-cluster redox enzyme
AE008917	1918870	syn	A	G	G	G	BMEI1868	NA
AE008917	1921409	nsyn	G	C	C	W	BMEI1871	NA
AE008917	1925915	nsyn	C	T	T	I	BMEI1875	Nucleotidyltransferase/DNA polymerase involved in DNA repair
AE008917	1928146	nsyn	C	T	A	V	BMEI1876	DNA polymerase III, alpha subunit
AE008917	1931141	syn	G	A	G	G	BMEI1877	Predicted membrane protein, hemolysin III homolog
AE008917	1933763	syn	C	G	P	P	BMEI1882	2-keto-4-pentenoate hydratase/2-oxohepta-3-ene-1, 7-dioic acid hydratase (catechol pathway)
AE008917	1935453	nsyn	A	G	L	P	BMEI1884	Hydrolases of the alpha/beta superfamily
AE008917	1935487	nsyn	T	C	T	A	BMEI1884	Hydrolases of the alpha/beta superfamily

AE008917	1935649	nsyn	A	G	Y	H	BMEI1884	Hydrolases of the alpha/beta superfamily
AE008917	1936182	nsyn	G	A	D	N	BMEI1885	Transcriptional regulator
AE008917	1937610	nsyn	A	G	H	R	BMEI1886	Phosphoglucomutase
AE008917	1943886	nsyn	C	T	G	E	BMEI1893	Uncharacterized protein conserved in bacteria
AE008917	1944435	intergenic	A	G	-	-	-	-
AE008917	1949159	syn	G	A	G	G	BMEI1894	Uncharacterized protein conserved in bacteria
AE008917	1949590	syn	G	A	G	G	BMEI1895	Outer membrane protein
AE008917	1949983	syn	T	G	T	T	BMEI1895	Outer membrane protein
AE008917	1950323	nsyn	G	A	P	L	BMEI1895	Outer membrane protein
AE008917	1953477	nsyn	A	G	V	A	BMEI1899	Heme/copper-type cytochrome/quinol oxidase, subunit 3
AE008917	1953571	nsyn	G	A	H	Y	BMEI1899	Heme/copper-type cytochrome/quinol oxidase, subunit 3
AE008917	1953869	syn	T	C	A	A	BMEI1900	Heme/copper-type cytochrome/quinol oxidases, subunit 1
AE008917	1957128	intergenic	A	G	-	-	-	-
AE008917	1958535	syn	G	A	N	N	BMEI1903	Cytochrome c2
AE008917	1959685	syn	C	T	D	D	BMEI1904	CMP-2-keto-3-deoxyoctulosonic acid synthetase
AE008917	1961465	nsyn	T	A	L	F	BMEI1906	NTP pyrophosphohydrolases containing a Zn-finger, probably nucleic-acid-binding
AE008917	1961646	nsyn	C	G	R	P	BMEI1906	NTP pyrophosphohydrolases containing a Zn-finger, probably nucleic-acid-binding
AE008917	1963993	syn	T	C	R	R	BMEI1908	DNA polymerase III, gamma/tau subunits
AE008917	1964877	nsyn	C	G	A	G	BMEI1909	Uncharacterized protein conserved in bacteria
AE008917	1965600	nsyn	G	A	V	M	BMEI1910	Recombinational DNA repair protein (RecF pathway)
AE008917	1967071	syn	G	A	T	T	BMEI1912	NA
AE008917	1967369	nsyn	A	G	R	G	BMEI1912	NA
AE008917	1967526	nsyn	A	G	K	R	BMEI1912	NA
AE008917	1968039	nsyn	A	T	I	N	BMEI1913	Transcriptional regulator
AE008917	1968282	nsyn	A	T	L	H	BMEI1913	Transcriptional regulator
AE008917	1969283	syn	G	A	K	K	BMEI1914	Predicted membrane protein
AE008917	1971543	syn	C	T	P	P	BMEI1915	Ribosomal protein S1
AE008917	1972593	nsyn	A	G	V	A	BMEI1916	Cytidylate kinase
AE008917	1972790	nsyn	C	G	G	A	BMEI1917	5-enolpyruvylshikimate-3-phosphate synthase
AE008917	1973623	syn	G	A	S	S	BMEI1917	5-enolpyruvylshikimate-3-phosphate synthase
AE008917	1974962	intergenic	A	G	-	-	-	-
AE008917	1976417	intergenic	T	C	-	-	-	-
AE008917	1976585	intergenic	T	A	-	-	-	-
AE008917	1978797	syn	C	A	G	G	BMEI1922	Acyl-coenzyme A synthetases/AMP-(fatty) acid ligases

AE008917	1978943	nsyn	G	A	G	D	BMEI1922	Acyl-coenzyme A synthetases/AMP-(fatty) acid ligases
AE008917	1980252	nsyn	A	G	M	V	BMEI1923	Acyl-CoA dehydrogenases
AE008917	1980664	syn	T	C	P	P	BMEI1924	Acetyl-CoA carboxylase, carboxyltransferase component (subunits alpha and beta)
AE008917	1981514	nsyn	A	G	S	G	BMEI1924	Acetyl-CoA carboxylase, carboxyltransferase component (subunits alpha and beta)
AE008917	1981797	nsyn	T	A	L	Q	BMEI1924	Acetyl-CoA carboxylase, carboxyltransferase component (subunits alpha and beta)
AE008917	1982925	syn	T	C	R	R	BMEI1925	Acetyl/propionyl-CoA carboxylase, alpha subunit
AE008917	1984503	nsyn	T	C	V	A	BMEI1926	Isopropylmalate/homocitrate/citramalate synthases
AE008917	1984683	nsyn	C	A	T	K	BMEI1926	Isopropylmalate/homocitrate/citramalate synthases
AE008917	1985655	nsyn	T	C	S	P	BMEI1928	Enoyl-CoA hydratase/carnithine racemase
AE008917	1985933	syn	A	G	L	L	BMEI1928	Enoyl-CoA hydratase/carnithine racemase
AE008917	1986382	syn	G	A	V	V	BMEI1930	ABC-type branched-chain amino acid transport systems, periplasmic component
AE008917	1986577	nsyn	C	G	D	E	BMEI1930	ABC-type branched-chain amino acid transport systems, periplasmic component
AE008917	1987140	nsyn	T	A	F	Y	BMEI1930	ABC-type branched-chain amino acid transport systems, periplasmic component
AE008917	1987540	intergenic	C	T	-	-	-	-
AE008917	1987882	nsyn	A	G	I	M	BMEI1931	NA
AE008917	1989447	syn	G	A	R	R	BMEI1934	ABC-type oligopeptide transport system, periplasmic component
AE008917	1991443	nsyn	C	G	D	E	BMEI1935	ABC-type oligopeptide transport system, periplasmic component
AE008917	1995717	nsyn	A	G	T	A	BMEI1938	ABC-type uncharacterized transport system, duplicated ATPase component
AE008917	1997145	nsyn	T	G	V	G	BMEI1939	Phosphoglycerate dehydrogenase and related dehydrogenases
AE008917	1997829	syn	G	A	A	A	BMEI1940	Dinucleotide-utilizing enzymes involved in molybdopterin and thiamine biosynthesis family 2
AE008917	2001854	syn	A	G	D	D	BMEI1943	ATPase involved in DNA replication initiation
AE008917	2003328	intergenic	A	G	-	-	-	-
AE008917	2003985	intergenic	A	C	-	-	-	-
AE008917	2004287	nsyn	T	G	E	A	BMEI1945	Enoyl-CoA hydratase/carnithine racemase
AE008917	2004719	nsyn	A	G	L	S	BMEI1945	Enoyl-CoA hydratase/carnithine racemase
AE008917	2009914	intergenic	C	A	-	-	-	-
AE008917	2011837	intergenic	T	C	-	-	-	-
AE008917	2011847	intergenic	T	G	-	-	-	-

AE008917	2012053	nsyn	A	G	L	P	BMEI1954	ABC-type metal ion transport system, periplasmic component/surface antigen
AE008917	2012281	nsyn	A	C	I	S	BMEI1954	ABC-type metal ion transport system, periplasmic component/surface antigen
AE008917	2013425	intergenic	T	C	-	-	-	-
AE008917	2014262	nsyn	G	C	V	L	BMEI1957	3-oxoacyl-(acyl-carrier-protein) synthase
AE008917	2014307	nsyn	A	G	K	E	BMEI1957	3-oxoacyl-(acyl-carrier-protein) synthase
AE008917	2016985	syn	A	G	S	S	BMEI1960	16S RNA G1207 methylase RsmC
AE008917	2017382	intergenic	C	A	-	-	-	-
AE008917	2017383	intergenic	G	C	-	-	-	-
AE008917	2017394	intergenic	C	G	-	-	-	-
AE008917	2020187	nsyn	T	C	D	G	BMEI1962	Ribosomal protein S15P/S13E
AE008917	2021508	syn	G	A	D	D	BMEI1964	Ribosome-binding factor A
AE008917	2023009	syn	A	G	A	A	BMEI1965	Translation initiation factor 2 (IF-2; GTPase)
AE008917	2024845	intergenic	A	G	-	-	-	-
AE008917	2025914	intergenic	A	G	-	-	-	-
AE008917	2026546	nsyn	C	A	Q	H	BMEI1967	Transcription elongation factor
AE008917	2026607	nsyn	G	A	S	L	BMEI1967	Transcription elongation factor
AE008917	2028427	intergenic	C	A	-	-	-	-
AE008917	2028547	nsyn	C	G	K	N	BMEI1969	Predicted S-adenosylmethionine-dependent methyltransferase
AE008917	2029303	nsyn	A	G	F	S	BMEI1970	S-adenosylmethionine synthetase
AE008917	2029350	syn	A	G	I	I	BMEI1970	S-adenosylmethionine synthetase
AE008917	2029479	syn	T	C	Q	Q	BMEI1970	S-adenosylmethionine synthetase
AE008917	2029582	nsyn	A	G	F	S	BMEI1970	S-adenosylmethionine synthetase
AE008917	2032819	nsyn	T	C	N	S	BMEI1972	Apolipoprotein N-acyltransferase
AE008917	2033946	nsyn	G	A	P	L	BMEI1973	Hemolysins and related proteins containing CBS domains
AE008917	2034633	nsyn	A	G	Y	H	BMEI1975	Phosphate starvation-inducible protein PhoH, predicted ATPase
AE008917	2034847	syn	G	A	F	F	BMEI1975	Phosphate starvation-inducible protein PhoH, predicted ATPase
AE008917	2035672	intergenic	C	T	-	-	-	-
AE008917	2037871	syn	A	G	S	S	BMEI1977	1-acyl-sn-glycerol-3-phosphate acyltransferase
AE008917	2038483	nsyn	C	G	W	S	BMEI1978	Acetyltransferases
AE008917	2039950	nsyn	A	G	K	E	BMEI1980	DNA-binding ferritin-like protein (oxidative damage protectant)
AE008917	2040759	nsyn	C	G	G	A	BMEI1981	Leucyl aminopeptidase (aminopeptidase T)
AE008917	2040761	syn	A	C	T	T	BMEI1981	Leucyl aminopeptidase (aminopeptidase T)
AE008917	2040765	nsyn	C	G	C	S	BMEI1981	Leucyl aminopeptidase (aminopeptidase T)
AE008917	2041371	nsyn	A	G	V	A	BMEI1981	Leucyl aminopeptidase (aminopeptidase T)

AE008917	2042068	syn	C	T	A	A	BMEI1983	Cell wall hydrolyses involved in spore germination
AE008917	2042870	nsyn	A	G	F	S	BMEI1983	Cell wall hydrolyses involved in spore germination
AE008917	2043603	nsyn	A	G	S	P	BMEI1984	Response regulators consisting of a CheY-like receiver domain and a winged-helix DNA-binding domain
AE008917	2044510	syn	T	C	A	A	BMEI1985	Phosphate uptake regulator
AE008917	2046140	nsyn	C	A	R	L	BMEI1987	ABC-type phosphate transport system, permease component
AE008917	2050065	intergenic	G	T	-	-	-	-
AE008917	2052238	nsyn	A	T	S	C	BMEI1993	Predicted periplasmic protein
AE008917	2053678	nsyn	T	G	*	C	BMEI1995	Predicted ATPase with chaperone activity
AE008917	2054129	nsyn	C	T	W	*	BMEI1995	Predicted ATPase with chaperone activity
AE008917	2054800	nsyn	T	C	T	A	BMEI1996	Glutathione synthase/Ribosomal protein S6 modification enzyme (glutaminyl transferase)
AE008917	2055129	nsyn	A	C	V	G	BMEI1996	Glutathione synthase/Ribosomal protein S6 modification enzyme (glutaminyl transferase)
AE008917	2055387	intergenic	A	G	-	-	-	-
AE008917	2057393	nsyn	G	A	A	V	BMEI1999	Orotidine-5'-phosphate decarboxylase
AE008917	2061559	nsyn	T	G	M	L	BMEI2002	Molecular chaperone
AE008917	2062684	nsyn	T	C	T	A	BMEI2003	Predicted dehydrogenases and related proteins
AE008917	2064820	nsyn	T	C	N	D	BMEI2004	Phenylalanyl-tRNA synthetase beta subunit
AE008917	2064823	nsyn	A	G	F	L	BMEI2004	Phenylalanyl-tRNA synthetase beta subunit
AE008917	2064901	nsyn	A	G	F	L	BMEI2004	Phenylalanyl-tRNA synthetase beta subunit
AE008917	2064902	nsyn	G	T	D	E	BMEI2004	Phenylalanyl-tRNA synthetase beta subunit
AE008917	2065431	nsyn	C	T	G	D	BMEI2005	Phenylalanyl-tRNA synthetase alpha subunit
AE008917	2066390	syn	G	A	L	L	BMEI2005	Phenylalanyl-tRNA synthetase alpha subunit
AE008917	2066492	intergenic	G	A	-	-	-	-
AE008917	2068415	nsyn	T	G	N	T	BMEI2009	Putative protein-S-isoprenylcysteine methyltransferase
AE008917	2070858	nsyn	T	G	S	A	BMEI2012	Uncharacterized protein involved in benzoate metabolism
AE008917	2071236	nsyn	G	A	G	N	BMEI2012	Uncharacterized protein involved in benzoate metabolism
AE008917	2071237	nsyn	G	A	G	N	BMEI2012	Uncharacterized protein involved in benzoate metabolism
AE008917	2071962	intergenic	A	G	-	-	-	-
AE008917	2074365	syn	T	C	L	L	BMEI2017	Phosphoribosylanthranilate isomerase
AE008917	2076520	syn	C	T	V	V	BMEI2019	Tryptophan synthase alpha chain
AE008917	2076719	nsyn	G	A	E	K	BMEI2019	Tryptophan synthase alpha chain
AE008917	2077209	nsyn	C	T	A	V	BMEI2019	Tryptophan synthase alpha chain
AE008917	2083492	nsyn	T	G	M	L	BMEI2023	ATP-dependent exoDNAse (exonuclease V) beta subunit (contains helicase and exonuclease domains)

AE008917	2083507	nsyn	T	C	K	E	BMEI2023	ATP-dependent exoDNAse (exonuclease V) beta subunit (contains helicase and exonuclease domains)
AE008917	2083536	nsyn	C	G	S	T	BMEI2023	ATP-dependent exoDNAse (exonuclease V) beta subunit (contains helicase and exonuclease domains)
AE008917	2083543	nsyn	C	G	V	L	BMEI2023	ATP-dependent exoDNAse (exonuclease V) beta subunit (contains helicase and exonuclease domains)
AE008917	2084465	nsyn	A	G	L	P	BMEI2024	Inactivated superfamily I helicase
AE008917	2085423	nsyn	C	T	E	K	BMEI2024	Inactivated superfamily I helicase
AE008917	2089854	syn	G	A	L	L	BMEI2027	FOG: PAS/PAC domain
AE008917	2090811	syn	A	G	D	D	BMEI2027	FOG: PAS/PAC domain
AE008917	2091018	syn	T	C	A	A	BMEI2027	FOG: PAS/PAC domain
AE008917	2091291	syn	G	C	S	S	BMEI2027	FOG: PAS/PAC domain
AE008917	2091639	intergenic	A	T	-	-	-	-
AE008917	2092211	intergenic	A	G	-	-	-	-
AE008917	2096512	syn	A	G	L	L	BMEI2035	Signal transduction histidine kinase
AE008917	2096714	nsyn	C	G	W	C	BMEI2035	Signal transduction histidine kinase
AE008917	2096721	nsyn	G	A	A	V	BMEI2035	Signal transduction histidine kinase
AE008917	2096723	syn	A	C	P	P	BMEI2035	Signal transduction histidine kinase
AE008917	2096748	nsyn	A	T	L	Q	BMEI2035	Signal transduction histidine kinase
AE008917	2097835	syn	C	A	G	G	BMEI2036	Response regulators consisting of a CheY-like receiver domain and a winged-helix DNA-binding domain
AE008917	2099579	syn	C	A	L	L	BMEI2037	Phosphoenolpyruvate carboxykinase (ATP)
AE008917	2100247	intergenic	A	G	-	-	-	-
AE008917	2101887	syn	G	A	G	G	BMEI2040	Phosphoribosyl-ATP pyrophosphohydrolase
AE008917	2104152	syn	C	T	A	A	BMEI2043	Glutamine amidotransferase
AE008917	2106163	nsyn	A	G	T	A	BMEI2047	ATP-dependent protease HslVU (ClpYQ), peptidase subunit
AE008917	2106848	nsyn	A	G	T	A	BMEI2048	ATP-dependent protease HslVU (ClpYQ), ATPase subunit
AE008917	2107864	intergenic	T	C	-	-	-	-
AE008917	2108455	nsyn	G	C	A	P	BMEI2049	NA
AE008917	2109007	nsyn	A	C	V	G	BMEI2050	Predicted transcriptional regulators
AE008917	2109953	intergenic	T	C	-	-	-	-
AE008917	2110117	nsyn	C	T	A	T	BMEI2052	Membrane-bound lytic murein transglycosylase
AE008917	2111979	intergenic	A	G	-	-	-	-
AE008917	2112056	intergenic	T	C	-	-	-	-
AE008917	2113742	syn	A	C	P	P	BMEI2056	DNA polymerase III, epsilon subunit and related 3'-5' exonucleases
AE008917	2114786	nsyn	T	C	T	A	BMEI2058	Shikimate 5-dehydrogenase

AE008917	2115588	nsyn	A	C	S	A	BMEI2059	Nucleotide-binding protein implicated in inhibition of septum formation
AE008917	2115869	nsyn	G	A	A	V	BMEI2059	Nucleotide-binding protein implicated in inhibition of septum formation
AE008917	2116241	nsyn	G	T	L	I	BMEI2060	Uncharacterized protein conserved in bacteria
AE008918	657	nsyn	A	G	W	R	BMEII0002	Acetyltransferases, including N-acetylases of ribosomal proteins
AE008918	3598	intergenic	T	C	-	-	-	-
AE008918	5483	nsyn	T	A	F	L	BMEII0008	NA
AE008918	6028	syn	A	G	L	L	BMEII0009	2-keto-3-deoxy-6-phosphogluconate aldolase
AE008918	6519	nsyn	C	T	A	T	BMEII0010	Uncharacterized protein conserved in bacteria
AE008918	6626	nsyn	C	T	R	H	BMEII0010	Uncharacterized protein conserved in bacteria
AE008918	9405	nsyn	C	G	R	A	BMEII0011	Response regulator containing CheY-like receiver, AAA-type ATPase, and DNA-binding domains
AE008918	9406	nsyn	G	C	R	A	BMEII0011	Response regulator containing CheY-like receiver, AAA-type ATPase, and DNA-binding domains
AE008918	11402	nsyn	A	G	D	G	BMEII0012	Oligoendopeptidase F
AE008918	12644	nsyn	A	G	Y	C	BMEII0013	Anthranilate/para-aminobenzoate synthases component I
AE008918	13940	syn	G	A	T	T	BMEII0014	Branched-chain amino acid aminotransferase/ 4-amino-4-deoxychorismate lyase
AE008918	14058	nsyn	T	G	F	V	BMEII0014	Branched-chain amino acid aminotransferase/ 4-amino-4-deoxychorismate lyase
AE008918	14541	nsyn	G	T	E	*	BMEII0015	Homospermidine synthase
AE008918	14699	syn	C	T	C	C	BMEII0015	Homospermidine synthase
AE008918	14879	syn	T	C	A	A	BMEII0015	Homospermidine synthase
AE008918	16808	syn	C	T	T	T	BMEII0018	Protoheme ferro-lyase (ferrochelatase)
AE008918	18491	syn	T	C	T	T	BMEII0019	Membrane protease subunits, stomatin/prohibitin homologs
AE008918	20007	nsyn	G	A	T	I	BMEII0021	Predicted sugar phosphate isomerase involved in capsule formation
AE008918	23597	nsyn	C	T	L	F	BMEII0024	Membrane-bound lytic murein transglycosylase B
AE008918	26067	syn	G	A	L	L	BMEII0026	NA
AE008918	26283	syn	G	C	A	A	BMEII0026	NA
AE008918	27157	nsyn	A	C	K	Q	BMEII0028	Type IV secretory pathway, VirB4 components
AE008918	30716	nsyn	A	G	T	A	BMEII0030	Type IV secretory pathway, VirB6 components
AE008918	31101	nsyn	T	A	F	Y	BMEII0030	Type IV secretory pathway, VirB6 components
AE008918	32704	syn	A	G	S	S	BMEII0033	Type IV secretory pathway, VirB9 components
AE008918	33020	nsyn	C	G	R	G	BMEII0033	Type IV secretory pathway, VirB9 components

AE008918	33245	intergenic	C	A	-	-	-	-
AE008918	33253	intergenic	T	G	-	-	-	-
AE008918	33318	intergenic	A	C	-	-	-	-
AE008918	33321	intergenic	A	G	-	-	-	-
AE008918	33322	intergenic	G	C	-	-	-	-
AE008918	35819	syn	A	C	V	V	BMEII0036	Outer membrane protein and related peptidoglycan-associated (lipo)proteins
AE008918	41408	syn	G	A	A	A	BMEII0040	Glutamate synthase domain 2
AE008918	43205	syn	G	T	R	R	BMEII0040	Glutamate synthase domain 2
AE008918	43496	syn	G	A	R	R	BMEII0040	Glutamate synthase domain 2
AE008918	43604	syn	C	T	Q	Q	BMEII0040	Glutamate synthase domain 2
AE008918	47227	syn	G	A	I	I	BMEII0042	Molecular chaperone (small heat shock protein)
AE008918	49000	nsyn	T	G	S	A	BMEII0047	Lysophospholipase
AE008918	50546	nsyn	A	C	Y	D	BMEII0048	Archaeal fructose-1,6-bisphosphatase and related enzymes of inositol monophosphatase family
AE008918	51311	syn	T	C	A	A	BMEII0049	N-formylglutamate amidohydrolase
AE008918	54009	nsyn	A	G	V	A	BMEII0052	Signal transduction histidine kinase
AE008918	54074	syn	C	T	R	R	BMEII0052	Signal transduction histidine kinase
AE008918	54685	syn	A	G	S	S	BMEII0053	Uncharacterized membrane protein
AE008918	56085	intergenic	C	A	-	-	-	-
AE008918	57969	syn	G	A	D	D	BMEII0056	Cation transport ATPase
AE008918	59493	intergenic	T	C	-	-	-	-
AE008918	64394	nsyn	T	C	M	V	BMEII0062	Dehydrogenases with different specificities (related to short-chain alcohol dehydrogenases)
AE008918	64998	nsyn	A	C	I	S	BMEII0063	Dehydrogenases with different specificities (related to short-chain alcohol dehydrogenases)
AE008918	66030	nsyn	G	A	T	I	BMEII0064	Lactoylglutathione lyase and related lyases
AE008918	67057	syn	C	T	E	E	BMEII0066	ABC-type branched-chain amino acid transport systems, ATPase component
AE008918	67855	nsyn	A	G	F	S	BMEII0067	ABC-type branched-chain amino acid transport system, permease component
AE008918	68129	nsyn	C	G	V	L	BMEII0067	ABC-type branched-chain amino acid transport system, permease component
AE008918	68799	nsyn	G	C	H	Q	BMEII0067	ABC-type branched-chain amino acid transport system, permease component

AE008918	70308	nsyn	G	A	A	V	BMEII0069	ABC-type branched-chain amino acid transport systems, periplasmic component
AE008918	70702	nsyn	A	G	*	Q	BMEII0070	ABC-type branched-chain amino acid transport systems, periplasmic component
AE008918	71320	intergenic	C	A	-	-	-	-
AE008918	72951	nsyn	A	C	I	R	BMEII0072	Uncharacterized protein conserved in bacteria
AE008918	72987	nsyn	C	G	G	A	BMEII0072	Uncharacterized protein conserved in bacteria
AE008918	73003	nsyn	C	T	G	K	BMEII0072	Uncharacterized protein conserved in bacteria
AE008918	73004	nsyn	C	G	G	V	BMEII0072	Uncharacterized protein conserved in bacteria
AE008918	73005	nsyn	C	A	G	V	BMEII0072	Uncharacterized protein conserved in bacteria
AE008918	73007	syn	T	C	A	A	BMEII0072	Uncharacterized protein conserved in bacteria
AE008918	73026	nsyn	T	C	D	G	BMEII0072	Uncharacterized protein conserved in bacteria
AE008918	73040	nsyn	A	C	D	E	BMEII0072	Uncharacterized protein conserved in bacteria
AE008918	73072	nsyn	G	T	Q	K	BMEII0072	Uncharacterized protein conserved in bacteria
AE008918	73073	syn	C	G	R	R	BMEII0072	Uncharacterized protein conserved in bacteria
AE008918	73086	nsyn	C	G	G	A	BMEII0072	Uncharacterized protein conserved in bacteria
AE008918	73094	syn	T	C	E	E	BMEII0072	Uncharacterized protein conserved in bacteria
AE008918	73114	nsyn	C	T	V	M	BMEII0072	Uncharacterized protein conserved in bacteria
AE008918	73145	syn	C	T	A	A	BMEII0072	Uncharacterized protein conserved in bacteria
AE008918	73878	nsyn	G	A	A	T	BMEII0073	NA
AE008918	73952	intergenic	C	T	-	-	-	-
AE008918	73984	intergenic	T	C	-	-	-	-
AE008918	74279	nsyn	T	C	F	L	BMEII0074	Thiosulfate reductase cytochrome B subunit (membrane anchoring protein)
AE008918	76042	nsyn	T	C	D	G	BMEII0076	Non-ribosomal peptide synthetase modules and related proteins
AE008918	76888	nsyn	T	G	D	A	BMEII0076	Non-ribosomal peptide synthetase modules and related proteins
AE008918	76894	nsyn	T	G	H	P	BMEII0076	Non-ribosomal peptide synthetase modules and related proteins
AE008918	76897	nsyn	A	G	F	S	BMEII0076	Non-ribosomal peptide synthetase modules and related proteins
AE008918	76901	nsyn	A	G	S	P	BMEII0076	Non-ribosomal peptide synthetase modules and related proteins
AE008918	77228	nsyn	A	G	K	E	BMEII0077	Isochorismate synthase
AE008918	77608	nsyn	G	A	M	I	BMEII0077	Isochorismate synthase
AE008918	78880	nsyn	A	C	H	P	BMEII0078	Peptide arylation enzymes
AE008918	78932	syn	T	C	P	P	BMEII0078	Peptide arylation enzymes
AE008918	79751	syn	G	A	L	L	BMEII0078	Peptide arylation enzymes
AE008918	80299	nsyn	C	T	A	V	BMEII0079	Isochorismate hydrolase
AE008918	80325	nsyn	G	T	D	Y	BMEII0079	Isochorismate hydrolase

AE008918	81034	syn	A	G	A	A	BMEII0080	Dehydrogenases with different specificities (related to short-chain alcohol dehydrogenases)
AE008918	82301	syn	C	T	L	L	BMEII0081	Phosphopantetheinyl transferase component of siderophore synthetase
AE008918	87614	syn	G	C	A	A	BMEII0087	Uncharacterized ABC-type transport system, permease component
AE008918	88887	syn	C	A	G	G	BMEII0088	Inosine-uridine nucleoside N-ribohydrolase
AE008918	89654	nsyn	C	G	W	C	BMEII0089	Sugar kinases, ribokinase family
AE008918	90569	intergenic	G	T	-	-	-	-
AE008918	93365	intergenic	G	A	-	-	-	-
AE008918	94775	intergenic	A	G	-	-	-	-
AE008918	95171	nsyn	C	T	L	F	BMEII0094	NA
AE008918	95521	nsyn	T	C	L	P	BMEII0096	Coproporphyrinogen III oxidase and related Fe-S oxidoreductases
AE008918	97108	nsyn	A	G	K	R	BMEII0097	Cation transport ATPase
AE008918	97265	syn	C	T	G	G	BMEII0097	Cation transport ATPase
AE008918	98280	nsyn	C	G	P	A	BMEII0097	Cation transport ATPase
AE008918	99833	nsyn	T	C	K	E	BMEII0099	ABC-type branched-chain amino acid transport systems, ATPase component
AE008918	101738	syn	A	G	G	G	BMEII0102	Branched-chain amino acid ABC-type transport system, permease components
AE008918	103289	syn	G	A	N	N	BMEII0103	ABC-type branched-chain amino acid transport systems, periplasmic component
AE008918	103756	nsyn	C	A	G	*	BMEII0103	ABC-type branched-chain amino acid transport systems, periplasmic component
AE008918	104174	nsyn	A	G	V	A	BMEII0104	AraC-type DNA-binding domain-containing proteins
AE008918	105102	intergenic	C	T	-	-	-	-
AE008918	105147	intergenic	G	A	-	-	-	-
AE008918	105178	intergenic	G	A	-	-	-	-
AE008918	108540	syn	C	T	G	G	BMEII0107	ABC-type nitrate/sulfonate/bicarbonate transport system, permease component
AE008918	109463	syn	C	T	D	D	BMEII0108	ABC-type nitrate/sulfonate/bicarbonate transport system, ATPase component
AE008918	111466	nsyn	A	G	T	A	BMEII0110	Arylsulfatase A and related enzymes
AE008918	111925	nsyn	G	T	D	Y	BMEII0110	Arylsulfatase A and related enzymes
AE008918	113636	nsyn	G	C	D	H	BMEII0111	Predicted phosphohydrolases
AE008918	115556	nsyn	G	C	A	P	BMEII0113	ABC-type sugar transport systems, permease components
AE008918	116413	nsyn	T	C	V	A	BMEII0114	ABC-type sugar transport system, permease component

AE008918	116772	syn	T	C	G	G	BMEII0115	ABC-type sugar transport system, periplasmic component
AE008918	119179	nsyn	G	A	W	*	BMEII0117	Predicted permeases
AE008918	120225	syn	A	G	Y	Y	BMEII0119	ABC-type branched-chain amino acid transport systems, ATPase component
AE008918	122623	nsyn	A	G	I	T	BMEII0121	Branched-chain amino acid ABC-type transport system, permease components
AE008918	124279	nsyn	T	C	N	S	BMEII0122	NA
AE008918	124991	nsyn	C	T	G	R	BMEII0123	Predicted metal-dependent hydrolase with the TIM-barrel fold
AE008918	125073	syn	C	T	P	P	BMEII0123	Predicted metal-dependent hydrolase with the TIM-barrel fold
AE008918	126944	nsyn	C	G	A	G	BMEII0124	NAD-dependent aldehyde dehydrogenases
AE008918	126945	nsyn	G	C	A	G	BMEII0124	NAD-dependent aldehyde dehydrogenases
AE008918	126951	nsyn	C	G	R	A	BMEII0124	NAD-dependent aldehyde dehydrogenases
AE008918	126952	nsyn	G	C	R	A	BMEII0124	NAD-dependent aldehyde dehydrogenases
AE008918	127135	syn	A	C	L	L	BMEII0125	NAD-dependent aldehyde dehydrogenases
AE008918	127796	nsyn	A	G	V	A	BMEII0125	NAD-dependent aldehyde dehydrogenases
AE008918	127836	nsyn	C	G	D	H	BMEII0125	NAD-dependent aldehyde dehydrogenases
AE008918	127837	syn	G	C	R	R	BMEII0125	NAD-dependent aldehyde dehydrogenases
AE008918	128924	nsyn	C	T	V	I	BMEII0126	Amino acid transporters
AE008918	129438	nsyn	C	G	S	A	BMEII0126	Amino acid transporters
AE008918	129440	nsyn	A	C	S	A	BMEII0126	Amino acid transporters
AE008918	129441	syn	T	A	T	T	BMEII0126	Amino acid transporters
AE008918	129531	nsyn	G	T	D	E	BMEII0126	Amino acid transporters
AE008918	129769	intergenic	G	A	-	-	-	-
AE008918	130651	nsyn	A	C	M	L	BMEII0128	Uncharacterized conserved protein
AE008918	131209	nsyn	C	T	H	S	BMEII0128	Uncharacterized conserved protein
AE008918	131210	nsyn	A	C	H	S	BMEII0128	Uncharacterized conserved protein
AE008918	131211	nsyn	T	G	H	S	BMEII0128	Uncharacterized conserved protein
AE008918	132189	syn	C	T	L	L	BMEII0129	Predicted hydrolase (HAD superfamily)
AE008918	133217	syn	G	A	G	G	BMEII0130	Adenosylmethionine-8-amino-7-oxononanoate aminotransferase
AE008918	133909	nsyn	C	A	L	M	BMEII0131	4-aminobutyrate aminotransferase and related aminotransferases
AE008918	134240	nsyn	G	A	R	H	BMEII0131	4-aminobutyrate aminotransferase and related aminotransferases
AE008918	134361	syn	T	G	G	G	BMEII0131	4-aminobutyrate aminotransferase and related aminotransferases
AE008918	135483	nsyn	A	G	Y	C	BMEII0132	Putative homoserine kinase type II (protein kinase fold)
AE008918	135691	syn	A	G	P	P	BMEII0132	Putative homoserine kinase type II (protein kinase fold)
AE008918	136795	intergenic	A	C	-	-	-	-
AE008918	137258	intergenic	T	A	-	-	-	-

AE008918	137710	syn	A	C	G	G	BMEII0135	NAD-dependent aldehyde dehydrogenases
AE008918	139094	nsyn	T	C	F	L	BMEII0136	Lactoylglutathione lyase and related lyases
AE008918	141474	syn	A	G	S	S	BMEII0138	2-keto-4-pentenoate hydratase
AE008918	143605	nsyn	C	T	V	I	BMEII0141	NAD-dependent aldehyde dehydrogenases
AE008918	143828	nsyn	T	C	*	W	BMEII0142	NAD-dependent aldehyde dehydrogenases
AE008918	145094	syn	G	A	D	D	BMEII0143	AraC-type DNA-binding domain-containing proteins
AE008918	146986	intergenic	A	G	-	-	-	-
AE008918	147801	nsyn	T	C	D	G	BMEII0145	ABC-type sugar transport system, ATPase component
AE008918	148405	nsyn	T	A	S	C	BMEII0145	ABC-type sugar transport system, ATPase component
AE008918	149609	nsyn	T	A	N	I	BMEII0146	ABC-type xylose transport system, periplasmic component
AE008918	149731	syn	G	A	P	P	BMEII0146	ABC-type xylose transport system, periplasmic component
AE008918	153299	nsyn	T	C	T	A	BMEII0148	Type V secretory pathway, adhesin AidA
AE008918	153358	nsyn	G	A	T	I	BMEII0148	Type V secretory pathway, adhesin AidA
AE008918	154313	nsyn	A	G	S	P	BMEII0148	Type V secretory pathway, adhesin AidA
AE008918	155249	nsyn	T	G	T	P	BMEII0148	Type V secretory pathway, adhesin AidA
AE008918	157921	nsyn	T	C	D	G	BMEII0148	Type V secretory pathway, adhesin AidA
AE008918	158530	nsyn	T	C	D	G	BMEII0148	Type V secretory pathway, adhesin AidA
AE008918	160799	nsyn	C	G	G	A	BMEII0149	Type V secretory pathway, adhesin AidA
AE008918	162119	intergenic	T	G	-	-	-	-
AE008918	164775	nsyn	T	C	S	P	BMEII0152	Flagellar biosynthesis/type III secretory pathway lipoprotein
AE008918	166115	nsyn	C	T	A	V	BMEII0154	Flagellar motor protein
AE008918	166143	nsyn	A	G	I	M	BMEII0154	Flagellar motor protein
AE008918	166586	nsyn	C	T	T	I	BMEII0154	Flagellar motor protein
AE008918	167641	nsyn	G	T	R	L	BMEII0155	NA
AE008918	168216	nsyn	C	T	P	S	BMEII0155	NA
AE008918	169559	nsyn	C	T	H	Y	BMEII0156	NA
AE008918	170356	intergenic	T	G	-	-	-	-
AE008918	170741	syn	C	T	R	R	BMEII0158	Response regulators consisting of a CheY-like receiver domain and a winged-helix DNA-binding domain
AE008918	171962	nsyn	A	G	I	V	BMEII0159	Flagellar hook protein FlgE
AE008918	175570	nsyn	A	T	T	S	BMEII0162	Flagellar biosynthesis regulator FlaF
AE008918	178725	syn	C	T	C	C	BMEII0167	Flagellar biosynthesis pathway, component FlhA
AE008918	181223	intergenic	A	G	-	-	-	-
AE008918	181231	intergenic	T	C	-	-	-	-
AE008918	181312	intergenic	A	C	-	-	-	-

AE008918	181782	nsyn	T	C	V	A	BMEII0172	NA
AE008918	184662	nsyn	T	G	Q	P	BMEII0177	ABC-type Mn/Zn transport systems, ATPase component
AE008918	184968	intergenic	T	C	-	-	-	-
AE008918	184973	intergenic	T	G	-	-	-	-
AE008918	185304	nsyn	C	T	L	F	BMEII0178	ABC-type Zn2+ transport system, periplasmic component/surface adhesin
AE008918	188031	intergenic	T	C	-	-	-	-
AE008918	188629	syn	A	C	G	G	BMEII0181	Predicted N-acetylglucosamine kinase
AE008918	201621	syn	G	A	V	V	BMEII0190	NA
AE008918	201794	nsyn	C	T	G	S	BMEII0191	NA
AE008918	203025	nsyn	T	C	Q	R	BMEII0192	Glutamate-1-semialdehyde aminotransferase
AE008918	203080	nsyn	T	C	T	A	BMEII0192	Glutamate-1-semialdehyde aminotransferase
AE008918	205387	nsyn	A	G	E	G	BMEII0193	ABC-type spermidine/putrescine transport systems, ATPase components
AE008918	205552	intergenic	T	C	-	-	-	-
AE008918	206606	syn	A	G	L	L	BMEII0195	ABC-type spermidine/putrescine transport system, permease component II
AE008918	207281	nsyn	C	T	A	V	BMEII0196	Spermidine/putrescine-binding periplasmic protein
AE008918	210079	nsyn	A	T	V	D	BMEII0198	Gluconolactonase
AE008918	212706	nsyn	G	A	P	S	BMEII0200	ABC-type dipeptide/oligopeptide/nickel transport system, ATPase component
AE008918	215052	nsyn	G	T	R	S	BMEII0203	ABC-type dipeptide transport system, periplasmic component
AE008918	217116	nsyn	G	A	H	Y	BMEII0205	ABC-type oligopeptide transport system, ATPase component
AE008918	218512	nsyn	T	C	Q	R	BMEII0206	ABC-type dipeptide/oligopeptide/nickel transport system, ATPase component
AE008918	218910	nsyn	G	A	S	F	BMEII0207	ABC-type dipeptide/oligopeptide/nickel transport systems, permease components
AE008918	219416	nsyn	G	A	T	I	BMEII0208	ABC-type dipeptide/oligopeptide/nickel transport systems, permease components
AE008918	220864	nsyn	G	A	R	C	BMEII0210	ABC-type dipeptide transport system, periplasmic component
AE008918	221248	nsyn	A	G	Y	H	BMEII0210	ABC-type dipeptide transport system, periplasmic component
AE008918	221441	syn	G	A	N	N	BMEII0210	ABC-type dipeptide transport system, periplasmic component
AE008918	222456	syn	A	G	A	A	BMEII0211	Protein related to penicillin acylase
AE008918	224009	syn	T	C	L	L	BMEII0212	Protein related to penicillin acylase
AE008918	224137	syn	G	A	L	L	BMEII0212	Protein related to penicillin acylase
AE008918	225577	nsyn	A	G	M	T	BMEII0213	Acyl-CoA dehydrogenases

AE008918	225730	nsyn	G	C	A	G	BMEII0213	Acyl-CoA dehydrogenases
AE008918	226877	nsyn	C	T	P	S	BMEII0214	Enoyl-CoA hydratase/carnithine racemase
AE008918	228315	nsyn	A	G	M	V	BMEII0215	3-hydroxyacyl-CoA dehydrogenase
AE008918	228410	syn	C	G	P	P	BMEII0215	3-hydroxyacyl-CoA dehydrogenase
AE008918	228839	nsyn	A	G	T	A	BMEII0216	Pyruvate/2-oxoglutarate dehydrogenase complex, dehydrogenase (E1) component, eukaryotic type, alpha subunit
AE008918	230534	nsyn	A	G	M	V	BMEII0216	Pyruvate/2-oxoglutarate dehydrogenase complex, dehydrogenase (E1) component, eukaryotic type, alpha subunit
AE008918	240305	syn	T	A	P	P	BMEII0225	NAD-dependent aldehyde dehydrogenases
AE008918	241504	intergenic	T	C	-	-	-	-
AE008918	241558	syn	A	G	R	R	BMEII0226	Transcriptional regulators
AE008918	242221	syn	C	T	E	E	BMEII0226	Transcriptional regulators
AE008918	242366	intergenic	A	G	-	-	-	-
AE008918	243672	syn	T	C	S	S	BMEII0228	Transposase and inactivated derivatives
AE008918	245243	nsyn	T	G	D	E	BMEII0230	Peptide methionine sulfoxide reductase
AE008918	245503	nsyn	A	G	F	S	BMEII0231	Uncharacterized protein conserved in bacteria
AE008918	246324	intergenic	C	G	-	-	-	-
AE008918	250354	nsyn	T	C	T	A	BMEII0236	Peptide chain release factor RF-3
AE008918	251469	nsyn	C	T	G	D	BMEII0236	Peptide chain release factor RF-3
AE008918	251651	syn	G	A	F	F	BMEII0236	Peptide chain release factor RF-3
AE008918	253170	nsyn	G	A	A	T	BMEII0239	Phosphatidylserine/phosphatidylglycerophosphate/cardiolipin synthases and related enzymes
AE008918	254753	nsyn	T	C	S	G	BMEII0240	Metal-dependent hydrolase
AE008918	255867	nsyn	C	T	A	T	BMEII0241	NAD-dependent aldehyde dehydrogenases
AE008918	261179	intergenic	G	T	-	-	-	-
AE008918	262406	nsyn	T	C	T	A	BMEII0249	Dihydrodipicolinate reductase
AE008918	267348	nsyn	G	A	P	S	BMEII0253	Murein endopeptidase
AE008918	267545	nsyn	T	G	E	A	BMEII0253	Murein endopeptidase
AE008918	267585	nsyn	C	T	V	M	BMEII0253	Murein endopeptidase
AE008918	268675	nsyn	T	C	K	E	BMEII0255	Uncharacterized Fe-S protein
AE008918	269269	nsyn	T	C	K	E	BMEII0255	Uncharacterized Fe-S protein
AE008918	274999	nsyn	G	T	F	L	BMEII0261	Predicted endonuclease containing a URI domain
AE008918	277886	nsyn	G	A	E	K	BMEII0265	Methionyl-tRNA formyltransferase

AE008918	280691	nsyn	C	T	C	Y	BMEII0268	Acetylornithine deacetylase/Succinyl-diaminopimelate desuccinylase and related deacylases
AE008918	281603	intergenic	A	G	-	-	-	-
AE008918	281727	nsyn	A	T	N	Y	BMEII0269	Predicted periplasmic protein
AE008918	281851	nsyn	A	G	H	R	BMEII0269	Predicted periplasmic protein
AE008918	282071	syn	T	C	L	L	BMEII0269	Predicted periplasmic protein
AE008918	282855	nsyn	T	C	N	S	BMEII0270	Tetrahydrodipicolinate N-succinyltransferase
AE008918	282863	syn	C	T	A	A	BMEII0270	Tetrahydrodipicolinate N-succinyltransferase
AE008918	286485	nsyn	G	A	A	V	BMEII0274	Predicted GTPase
AE008918	286965	intergenic	A	C	-	-	-	-
AE008918	287246	nsyn	A	G	I	T	BMEII0275	Preprotein translocase subunit YidC
AE008918	287477	nsyn	T	C	K	R	BMEII0275	Preprotein translocase subunit YidC
AE008918	288571	syn	A	C	L	L	BMEII0275	Preprotein translocase subunit YidC
AE008918	289143	nsyn	G	T	A	E	BMEII0276	RNase P protein component
AE008918	290128	intergenic	T	G	-	-	-	-
AE008918	293112	intergenic	C	A	-	-	-	-
AE008918	293130	intergenic	A	C	-	-	-	-
AE008918	293166	intergenic	A	G	-	-	-	-
AE008918	296837	nsyn	C	G	N	K	BMEII0284	ABC-type dipeptide transport system, periplasmic component
AE008918	296838	nsyn	G	C	A	P	BMEII0284	ABC-type dipeptide transport system, periplasmic component
AE008918	296928	nsyn	T	G	S	A	BMEII0284	ABC-type dipeptide transport system, periplasmic component
AE008918	299213	syn	T	C	A	A	BMEII0286	ABC-type dipeptide/oligopeptide/nickel transport systems, permease components
AE008918	299848	syn	T	C	T	T	BMEII0287	ABC-type dipeptide/oligopeptide/nickel transport system, ATPase component
AE008918	301117	nsyn	A	G	N	D	BMEII0288	ABC-type oligopeptide transport system, ATPase component
AE008918	301373	nsyn	T	A	V	D	BMEII0288	ABC-type oligopeptide transport system, ATPase component
AE008918	302394	nsyn	A	G	E	G	BMEII0289	Asp-tRNAAsn/Glu-tRNAGln amidotransferase A subunit and related amidases
AE008918	302822	nsyn	T	G	S	A	BMEII0289	Asp-tRNAAsn/Glu-tRNAGln amidotransferase A subunit and related amidases
AE008918	303123	nsyn	C	G	R	P	BMEII0290	DNA polymerase III, epsilon subunit and related 3'-5' exonucleases
AE008918	305574	intergenic	A	G	-	-	-	-
AE008918	307437	intergenic	C	T	-	-	-	-
AE008918	307767	nsyn	T	C	L	P	BMEII0293	NA

AE008918	309250	syn	A	C	I	I	BMEII0295	Lactoylglutathione lyase and related lyases
AE008918	311436	syn	A	G	E	E	BMEII0297	Outer membrane receptor proteins, mostly Fe transport
AE008918	315703	syn	T	G	L	L	BMEII0300	ABC-type sugar transport system, ATPase component
AE008918	315792	nsyn	C	G	A	G	BMEII0300	ABC-type sugar transport system, ATPase component
AE008918	315794	nsyn	C	G	P	A	BMEII0300	ABC-type sugar transport system, ATPase component
AE008918	315839	nsyn	T	C	W	L	BMEII0300	ABC-type sugar transport system, ATPase component
AE008918	315840	nsyn	G	T	W	L	BMEII0300	ABC-type sugar transport system, ATPase component
AE008918	316223	nsyn	G	A	E	K	BMEII0300	ABC-type sugar transport system, ATPase component
AE008918	317242	nsyn	G	A	M	I	BMEII0300	ABC-type sugar transport system, ATPase component
AE008918	317288	nsyn	G	A	G	R	BMEII0300	ABC-type sugar transport system, ATPase component
AE008918	320703	syn	G	A	F	F	BMEII0305	Sulfite oxidase and related enzymes
AE008918	321230	nsyn	G	C	P	A	BMEII0305	Sulfite oxidase and related enzymes
AE008918	321371	nsyn	C	T	A	T	BMEII0305	Sulfite oxidase and related enzymes
AE008918	323110	syn	T	C	P	P	BMEII0307	FOG: WD40 repeat
AE008918	323584	syn	T	G	A	A	BMEII0307	FOG: WD40 repeat
AE008918	324102	nsyn	G	A	P	L	BMEII0308	Putative GTPases (G3E family)
AE008918	328299	syn	C	T	D	D	BMEII0313	Lactate dehydrogenase and related dehydrogenases
AE008918	328703	nsyn	T	C	M	T	BMEII0313	Lactate dehydrogenase and related dehydrogenases
AE008918	329716	nsyn	T	C	S	G	BMEII0314	Predicted glutamine amidotransferases
AE008918	330634	syn	C	T	C	C	BMEII0316	Dehydrogenases with different specificities (related to short-chain alcohol dehydrogenases)
AE008918	334540	nsyn	G	A	A	K	BMEII0318	Beta-lactamase class C and other penicillin binding proteins
AE008918	334541	nsyn	C	A	A	K	BMEII0318	Beta-lactamase class C and other penicillin binding proteins
AE008918	334583	nsyn	A	G	*	W	BMEII0318	Beta-lactamase class C and other penicillin binding proteins
AE008918	336339	nsyn	G	A	G	S	BMEII0322	NA
AE008918	337059	nsyn	A	G	I	M	BMEII0323	NAD/NADP transhydrogenase alpha subunit
AE008918	337880	nsyn	C	T	A	V	BMEII0323	NAD/NADP transhydrogenase alpha subunit
AE008918	338090	syn	T	G	A	A	BMEII0324	NAD/NADP transhydrogenase alpha subunit
AE008918	343241	intergenic	T	C	-	-	-	-
AE008918	343370	nsyn	T	C	*	W	BMEII0330	NA
AE008918	344937	nsyn	G	A	S	L	BMEII0333	NA
AE008918	345589	syn	C	G	G	G	BMEII0334	FOG: TPR repeat
AE008918	349854	nsyn	T	G	H	P	BMEII0339	Choline dehydrogenase and related flavoproteins
AE008918	351355	nsyn	C	T	A	T	BMEII0340	ABC-type branched-chain amino acid transport system, permease component

AE008918	351460	nsyn	T	G	K	Q	BMEII0340	ABC-type branched-chain amino acid transport system, permease component
AE008918	351523	syn	A	G	L	L	BMEII0340	ABC-type branched-chain amino acid transport system, permease component
AE008918	351551	syn	T	C	Q	Q	BMEII0340	ABC-type branched-chain amino acid transport system, permease component
AE008918	354313	nsyn	A	G	W	R	BMEII0343	ABC-type branched-chain amino acid transport systems, ATPase component
AE008918	356659	syn	C	T	S	S	BMEII0345	Transcriptional regulator
AE008918	357316	intergenic	T	C	-	-	-	-
AE008918	360143	nsyn	C	T	S	L	BMEII0348	4-aminobutyrate aminotransferase and related aminotransferases
AE008918	360884	nsyn	T	C	V	A	BMEII0348	4-aminobutyrate aminotransferase and related aminotransferases
AE008918	362572	nsyn	C	T	E	K	BMEII0350	Beta-lactamase class C and other penicillin binding proteins
AE008918	363945	intergenic	A	T	-	-	-	-
AE008918	364136	intergenic	C	T	-	-	-	-
AE008918	365340	nsyn	T	C	S	P	BMEII0351	Thiamine pyrophosphate-requiring enzymes [acetolactate synthase, pyruvate dehydrogenase (cytochrome), glyoxylate carboligase, phosphonopyruvate decarboxylase]
AE008918	365405	syn	T	C	I	I	BMEII0351	Thiamine pyrophosphate-requiring enzymes [acetolactate synthase, pyruvate dehydrogenase (cytochrome), glyoxylate carboligase, phosphonopyruvate decarboxylase]
AE008918	369886	nsyn	C	T	P	S	BMEII0356	Dihydroxyacid dehydratase/phosphogluconate dehydratase
AE008918	372391	syn	G	A	E	E	BMEII0358	2-keto-3-deoxy-6-phosphogluconate aldolase
AE008918	377318	syn	G	A	A	A	BMEII0363	Branched-chain amino acid aminotransferase/ 4-amino-4-deoxychorismate lyase
AE008918	378856	nsyn	C	T	E	K	BMEII0365	Urocanate hydratase
AE008918	379341	nsyn	A	G	V	A	BMEII0365	Urocanate hydratase
AE008918	381531	nsyn	C	A	E	*	BMEII0367	Histidine ammonia-lyase
AE008918	384786	syn	G	A	T	T	BMEII0369	Cytosine deaminase and related metal-dependent hydrolases
AE008918	385009	nsyn	A	G	K	G	BMEII0369	Cytosine deaminase and related metal-dependent hydrolases
AE008918	385010	nsyn	A	G	K	G	BMEII0369	Cytosine deaminase and related metal-dependent hydrolases
AE008918	389599	nsyn	A	T	L	M	BMEII0374	Alanine racemase
AE008918	391413	intergenic	A	G	-	-	-	-
AE008918	391630	nsyn	C	T	D	N	BMEII0376	Opacity protein and related surface antigens
AE008918	392386	intergenic	A	G	-	-	-	-
AE008918	392606	intergenic	G	A	-	-	-	-

AE008918	393697	syn	A	T	T	T	BMEII0377	L-lactate dehydrogenase (FMN-dependent) and related alpha-hydroxy acid dehydrogenases
AE008918	395060	nsyn	G	A	R	Q	BMEII0378	Anaerobic dehydrogenases, typically selenocysteine-containing
AE008918	395112	nsyn	G	A	M	I	BMEII0378	Anaerobic dehydrogenases, typically selenocysteine-containing
AE008918	395291	nsyn	C	T	A	V	BMEII0378	Anaerobic dehydrogenases, typically selenocysteine-containing
AE008918	395546	nsyn	G	A	R	H	BMEII0378	Anaerobic dehydrogenases, typically selenocysteine-containing
AE008918	396087	syn	T	C	A	A	BMEII0378	Anaerobic dehydrogenases, typically selenocysteine-containing
AE008918	397791	nsyn	G	A	A	T	BMEII0380	Membrane-fusion protein
AE008918	400392	syn	C	T	T	T	BMEII0382	Cation/multidrug efflux pump
AE008918	400467	syn	A	C	G	G	BMEII0382	Cation/multidrug efflux pump
AE008918	403881	nsyn	T	C	S	P	BMEII0384	Predicted phosphosugar isomerases
AE008918	404983	syn	T	C	G	G	BMEII0385	N-acetylglucosamine-6-phosphate deacetylase
AE008918	407221	intergenic	T	A	-	-	-	-
AE008918	409991	nsyn	C	T	S	F	BMEII0390	Transcriptional regulator
AE008918	410931	syn	G	C	G	G	BMEII0391	Glycine/D-amino acid oxidases (deaminating)
AE008918	412020	intergenic	G	A	-	-	-	-
AE008918	414040	nsyn	G	A	R	C	BMEII0392	Transcriptional accessory protein
AE008918	414760	nsyn	T	C	V	A	BMEII0393	Predicted transcriptional regulators
AE008918	415513	nsyn	T	G	S	A	BMEII0394	NADH:flavin oxidoreductases, Old Yellow Enzyme family
AE008918	417506	syn	A	T	V	V	BMEII0396	Arginase/agmatinase/formimionoglutamate hydrolase, arginase family
AE008918	418035	nsyn	C	G	A	G	BMEII0397	Predicted ornithine cyclodeaminase, mu-crystallin homolog
AE008918	418088	nsyn	A	G	S	G	BMEII0397	Predicted ornithine cyclodeaminase, mu-crystallin homolog
AE008918	419873	intergenic	C	A	-	-	-	-
AE008918	419966	intergenic	T	C	-	-	-	-
AE008918	422401	nsyn	T	A	F	L	BMEII0402	Uncharacterized protein, similar to the N-terminal domain of Lon protease
AE008918	423640	nsyn	G	C	G	A	BMEII0404	Isocitrate/isopropylmalate dehydrogenase
AE008918	424723	syn	T	G	G	G	BMEII0405	Adenosine deaminase
AE008918	426367	nsyn	C	T	A	T	BMEII0406	Permeases of the major facilitator superfamily
AE008918	427417	nsyn	C	G	V	L	BMEII0406	Permeases of the major facilitator superfamily
AE008918	427490	syn	A	G	G	G	BMEII0406	Permeases of the major facilitator superfamily
AE008918	429247	intergenic	G	T	-	-	-	-
AE008918	431739	nsyn	T	C	N	D	BMEII0412	NA
AE008918	432539	nsyn	G	A	G	D	BMEII0413	Citrate lyase beta subunit
AE008918	436187	nsyn	T	G	S	A	BMEII0418	Mg/Co/Ni transporter MgtE (contains CBS domain)

AE008918	436560	nsyn	C	A	A	E	BMEII0418	Mg/Co/Ni transporter MgtE (contains CBS domain)
AE008918	437303	intergenic	T	G	-	-	-	-
AE008918	441946	nsyn	C	G	A	G	BMEII0423	Fructose/tagatose bisphosphate aldolase
AE008918	443437	nsyn	A	G	C	R	BMEII0425	Triosephosphate isomerase
AE008918	443969	syn	C	T	L	L	BMEII0426	Transcriptional regulators of sugar metabolism
AE008918	446345	syn	C	T	V	V	BMEII0428	NA
AE008918	448551	syn	A	G	S	S	BMEII0430	Sugar (pentulose and hexulose) kinases
AE008918	452061	nsyn	A	C	R	S	BMEII0432	ABC-type sugar transport system, ATPase component
AE008918	452299	nsyn	A	G	T	A	BMEII0432	ABC-type sugar transport system, ATPase component
AE008918	452605	nsyn	A	G	T	A	BMEII0432	ABC-type sugar transport system, ATPase component
AE008918	453663	nsyn	G	A	A	T	BMEII0433	Ribose/xylose/arabinose/galactoside ABC-type transport systems, permease components
AE008918	453915	nsyn	A	G	Y	C	BMEII0434	NA
AE008918	453936	nsyn	T	C	L	P	BMEII0434	NA
AE008918	454251	nsyn	T	C	S	P	BMEII0435	ABC-type sugar transport system, periplasmic component
AE008918	454892	intergenic	T	C	-	-	-	-
AE008918	457051	syn	C	T	L	L	BMEII0437	4-hydroxybenzoate polyprenyltransferase and related prenyltransferases
AE008918	457081	nsyn	G	C	V	L	BMEII0437	4-hydroxybenzoate polyprenyltransferase and related prenyltransferases
AE008918	457227	nsyn	T	G	H	Q	BMEII0437	4-hydroxybenzoate polyprenyltransferase and related prenyltransferases
AE008918	457575	syn	T	C	S	S	BMEII0437	4-hydroxybenzoate polyprenyltransferase and related prenyltransferases
AE008918	458082	nsyn	T	C	S	P	BMEII0438	FAD/FMN-containing dehydrogenases
AE008918	462008	nsyn	C	T	L	F	BMEII0441	Ornithine/acetylornithine aminotransferase
AE008918	462324	intergenic	C	T	-	-	-	-
AE008918	462888	syn	T	C	A	A	BMEII0442	NA
AE008918	465038	intergenic	A	C	-	-	-	-
AE008918	465152	intergenic	A	G	-	-	-	-
AE008918	465211	intergenic	G	A	-	-	-	-
AE008918	465240	intergenic	C	T	-	-	-	-
AE008918	465280	intergenic	C	T	-	-	-	-
AE008918	465704	syn	A	G	G	G	BMEII0445	Transposase and inactivated derivatives
AE008918	465775	nsyn	A	G	F	L	BMEII0445	Transposase and inactivated derivatives
AE008918	466843	nsyn	G	A	R	C	BMEII0446	NA

AE008918	467608	nsyn	G	A	R	W	BMEII0446	NA
AE008918	467652	nsyn	C	T	G	E	BMEII0446	NA
AE008918	468750	syn	T	C	L	L	BMEII0447	Uncharacterized conserved protein
AE008918	469152	nsyn	C	T	D	N	BMEII0448	Predicted metal-dependent hydrolase
AE008918	470475	intergenic	G	C	-	-	-	-
AE008918	474202	syn	G	A	N	N	BMEII0451	Type I restriction-modification system methyltransferase subunit
AE008918	475246	syn	C	T	S	S	BMEII0451	Type I restriction-modification system methyltransferase subunit
AE008918	475366	syn	T	G	P	P	BMEII0451	Type I restriction-modification system methyltransferase subunit
AE008918	478180	intergenic	G	C	-	-	-	-
AE008918	482851	nsyn	A	G	N	D	BMEII0461	NA
AE008918	486115	syn	T	C	S	S	BMEII0463	Predicted ICC-like phosphoesterases
AE008918	487044	syn	T	C	L	L	BMEII0464	NA
AE008918	489951	intergenic	T	G	-	-	-	-
AE008918	489952	intergenic	G	T	-	-	-	-
AE008918	490291	intergenic	G	A	-	-	-	-
AE008918	491242	intergenic	T	C	-	-	-	-
AE008918	493134	syn	T	G	G	G	BMEII0469	Integral membrane protein possibly involved in chromosome condensation
AE008918	493357	nsyn	T	C	T	A	BMEII0470	Integral membrane protein possibly involved in chromosome condensation
AE008918	496669	syn	A	G	V	V	BMEII0473	Cation/multidrug efflux pump
AE008918	503072	nsyn	C	T	T	M	BMEII0478	Mannitol-1-phosphate/altronate dehydrogenases
AE008918	504605	nsyn	C	G	A	G	BMEII0479	ABC-type uncharacterized transport system, periplasmic component
AE008918	507378	nsyn	C	T	T	I	BMEII0484	ABC-type spermidine/putrescine transport system, permease component II
AE008918	508811	syn	C	T	G	G	BMEII0485	Altronate dehydratase
AE008918	510358	nsyn	T	C	F	S	BMEII0487	ABC-type dipeptide transport system, periplasmic component
AE008918	510647	syn	A	G	A	A	BMEII0487	ABC-type dipeptide transport system, periplasmic component
AE008918	512479	syn	T	C	V	V	BMEII0488	ABC-type dipeptide/oligopeptide/nickel transport systems, permease components
AE008918	512833	nsyn	C	T	R	W	BMEII0489	ABC-type dipeptide/oligopeptide/nickel transport systems, permease components
AE008918	512836	nsyn	G	C	V	L	BMEII0489	ABC-type dipeptide/oligopeptide/nickel transport systems, permease components

AE008918	512860	nsyn	A	G	N	D	BMEII0489	ABC-type dipeptide/oligopeptide/nickel transport systems, permease components
AE008918	516439	intergenic	C	T	-	-	-	-
AE008918	516931	nsyn	A	G	V	A	BMEII0493	Transcriptional regulator
AE008918	516989	nsyn	T	A	K	*	BMEII0493	Transcriptional regulator
AE008918	516990	syn	C	A	V	V	BMEII0493	Transcriptional regulator
AE008918	517050	syn	T	C	G	G	BMEII0493	Transcriptional regulator
AE008918	521356	nsyn	G	T	A	S	BMEII0496	Acetyl-CoA acetyltransferase
AE008918	521388	syn	G	C	R	R	BMEII0496	Acetyl-CoA acetyltransferase
AE008918	523298	nsyn	A	C	K	T	BMEII0497	3-hydroxyacyl-CoA dehydrogenase
AE008918	524164	nsyn	A	G	K	E	BMEII0498	Predicted transcriptional regulator
AE008918	524493	syn	C	T	I	I	BMEII0498	Predicted transcriptional regulator
AE008918	524637	intergenic	T	C	-	-	-	-
AE008918	526024	syn	G	A	N	N	BMEII0500	Lysyl-tRNA synthetase (class I)
AE008918	526245	nsyn	T	C	T	A	BMEII0500	Lysyl-tRNA synthetase (class I)
AE008918	526605	nsyn	C	T	G	R	BMEII0500	Lysyl-tRNA synthetase (class I)
AE008918	526789	syn	C	G	L	L	BMEII0500	Lysyl-tRNA synthetase (class I)
AE008918	526822	syn	A	G	A	A	BMEII0500	Lysyl-tRNA synthetase (class I)
AE008918	528622	intergenic	C	G	-	-	-	-
AE008918	528633	intergenic	G	A	-	-	-	-
AE008918	528636	intergenic	G	C	-	-	-	-
AE008918	528641	intergenic	G	C	-	-	-	-
AE008918	529156	intergenic	A	G	-	-	-	-
AE008918	531218	nsyn	A	G	H	R	BMEII0505	ABC-type dipeptide/oligopeptide/nickel transport systems, permease components
AE008918	533082	nsyn	C	G	S	*	BMEII0507	ABC-type dipeptide/oligopeptide/nickel transport system, ATPase component
AE008918	537032	nsyn	T	C	K	E	BMEII0511	Dihydroxyacid dehydratase/phosphogluconate dehydratase
AE008918	537555	syn	A	G	L	L	BMEII0511	Dihydroxyacid dehydratase/phosphogluconate dehydratase
AE008918	537875	nsyn	C	T	G	R	BMEII0511	Dihydroxyacid dehydratase/phosphogluconate dehydratase
AE008918	541168	nsyn	A	G	N	D	BMEII0514	Dehydrogenases with different specificities (related to short-chain alcohol dehydrogenases)
AE008918	543232	intergenic	C	A	-	-	-	-
AE008918	543503	syn	A	G	L	L	BMEII0516	Predicted membrane protein
AE008918	547583	nsyn	C	T	G	R	BMEII0521	Glycine/D-amino acid oxidases (deaminating)
AE008918	549238	syn	G	A	T	T	BMEII0523	Glutamine synthetase

AE008918	550396	nsyn	G	A	A	V	BMEII0524	NA
AE008918	552686	syn	C	T	T	T	BMEII0527	Exonuclease VII, large subunit
AE008918	553292	syn	A	C	G	G	BMEII0527	Exonuclease VII, large subunit
AE008918	553341	nsyn	G	A	V	I	BMEII0527	Exonuclease VII, large subunit
AE008918	557424	nsyn	A	G	N	S	BMEII0531	Predicted membrane protein
AE008918	558088	syn	T	C	R	R	BMEII0531	Predicted membrane protein
AE008918	560844	nsyn	A	G	I	M	BMEII0535	ABC-type Fe3+-hydroxamate transport system, periplasmic component
AE008918	563009	nsyn	G	T	A	S	BMEII0537	ABC-type cobalamin/Fe3+-siderophores transport systems, ATPase components
AE008918	563104	nsyn	T	G	Y	D	BMEII0538	NA
AE008918	563973	syn	G	A	N	N	BMEII0539	Uncharacterized conserved protein
AE008918	564565	nsyn	A	G	L	P	BMEII0539	Uncharacterized conserved protein
AE008918	566114	syn	G	A	V	V	BMEII0541	ABC-type sugar transport system, permease component
AE008918	568473	syn	T	C	F	F	BMEII0543	Dehydrogenases with different specificities (related to short-chain alcohol dehydrogenases)
AE008918	570791	nsyn	C	T	A	V	BMEII0546	Predicted amino acid aldolase or racemase
AE008918	570792	nsyn	C	T	A	V	BMEII0546	Predicted amino acid aldolase or racemase
AE008918	570974	nsyn	A	T	Q	L	BMEII0546	Predicted amino acid aldolase or racemase
AE008918	571820	nsyn	A	G	K	E	BMEII0547	NA
AE008918	572563	intergenic	C	T	-	-	-	-
AE008918	575880	intergenic	T	C	-	-	-	-
AE008918	576060	nsyn	G	T	P	Q	BMEII0551	Permeases of the drug/metabolite transporter (DMT) superfamily
AE008918	576384	nsyn	A	G	V	A	BMEII0551	Permeases of the drug/metabolite transporter (DMT) superfamily
AE008918	579516	nsyn	C	T	A	T	BMEII0554	Glutamine synthetase
AE008918	581490	syn	C	T	S	S	BMEII0556	Transcriptional regulators
AE008918	583324	nsyn	T	G	F	V	BMEII0557	Uncharacterized NAD(FAD)-dependent dehydrogenases
AE008918	583882	intergenic	G	T	-	-	-	-
AE008918	584352	intergenic	T	C	-	-	-	-
AE008918	587401	nsyn	T	C	L	P	BMEII0561	Glycine cleavage system protein P (pyridoxal-binding), C-terminal domain
AE008918	587681	syn	G	T	T	T	BMEII0561	Glycine cleavage system protein P (pyridoxal-binding), C-terminal domain
AE008918	588774	nsyn	G	A	E	K	BMEII0561	Glycine cleavage system protein P (pyridoxal-binding), C-terminal domain
AE008918	589315	intergenic	A	G	-	-	-	-

AE008918	592553	syn	G	A	A	A	BMEII0564	Delta 1-pyrroline-5-carboxylate dehydrogenase
AE008918	593435	nsyn	C	A	D	E	BMEII0564	Delta 1-pyrroline-5-carboxylate dehydrogenase
AE008918	594314	intergenic	A	G	-	-	-	-
AE008918	595067	nsyn	G	C	G	A	BMEII0565	ABC-type Fe3+ transport system, periplasmic component
AE008918	596026	intergenic	T	G	-	-	-	-
AE008918	596058	intergenic	C	G	-	-	-	-
AE008918	596059	intergenic	G	C	-	-	-	-
AE008918	596143	intergenic	C	T	-	-	-	-
AE008918	596339	syn	C	G	T	T	BMEII0566	ABC-type Fe3+ transport system, permease component
AE008918	596930	syn	G	A	S	S	BMEII0566	ABC-type Fe3+ transport system, permease component
AE008918	598686	nsyn	T	C	V	A	BMEII0567	ABC-type spermidine/putrescine transport systems, ATPase components
AE008918	599718	syn	G	C	A	A	BMEII0568	Archaeal fructose-1,6-bisphosphatase and related enzymes of inositol monophosphatase family
AE008918	602162	syn	A	G	V	V	BMEII0571	Acetolactate synthase
AE008918	602822	syn	T	G	P	P	BMEII0571	Acetolactate synthase
AE008918	603196	nsyn	G	C	R	G	BMEII0571	Acetolactate synthase
AE008918	604139	nsyn	G	A	A	V	BMEII0572	Sugar kinases, ribokinase family
AE008918	605128	syn	C	T	L	L	BMEII0572	Sugar kinases, ribokinase family
AE008918	606222	intergenic	T	C	-	-	-	-
AE008918	606493	nsyn	A	G	K	E	BMEII0574	Predicted dehydrogenases and related proteins
AE008918	607080	syn	T	C	D	D	BMEII0574	Predicted dehydrogenases and related proteins
AE008918	607720	syn	C	T	T	T	BMEII0575	NA
AE008918	609300	nsyn	A	T	L	M	BMEII0576	Transcriptional regulator
AE008918	609373	syn	T	C	L	L	BMEII0576	Transcriptional regulator
AE008918	609777	syn	T	C	F	F	BMEII0577	Peroxiredoxin
AE008918	609839	nsyn	C	T	S	L	BMEII0577	Peroxiredoxin
AE008918	610010	nsyn	G	T	G	V	BMEII0577	Peroxiredoxin
AE008918	610828	intergenic	C	G	-	-	-	-
AE008918	610830	intergenic	C	G	-	-	-	-
AE008918	610833	intergenic	A	C	-	-	-	-
AE008918	610836	intergenic	A	C	-	-	-	-
AE008918	610837	intergenic	A	C	-	-	-	-
AE008918	610838	intergenic	T	C	-	-	-	-
AE008918	610842	intergenic	C	G	-	-	-	-
AE008918	610844	intergenic	C	G	-	-	-	-

AE008918	611597	nsyn	T	A	Q	L	BMEII0579	Permeases of the major facilitator superfamily
AE008918	612579	intergenic	T	G	-	-	-	-
AE008918	615283	intergenic	C	G	-	-	-	-
AE008918	617000	intergenic	A	G	-	-	-	-
AE008918	619432	nsyn	A	C	T	P	BMEII0585	ABC-type Fe3+ transport system, permease component
AE008918	622543	nsyn	A	C	Q	P	BMEII0588	Uncharacterized protein required for formate dehydrogenase activity
AE008918	623386	nsyn	C	T	G	D	BMEII0589	Riboflavin synthase beta-chain
AE008918	624232	intergenic	G	A	-	-	-	-
AE008918	625474	nsyn	G	A	A	T	BMEII0590	ABC-type sugar transport system, periplasmic component
AE008918	627239	nsyn	C	T	R	C	BMEII0592	ABC-type sugar transport system, permease component
AE008918	627377	nsyn	A	C	K	Q	BMEII0592	ABC-type sugar transport system, permease component
AE008918	628514	nsyn	C	T	P	S	BMEII0593	ABC-type sugar transport systems, ATPase components
AE008918	628835	intergenic	C	T	-	-	-	-
AE008918	628953	syn	C	T	A	A	BMEII0594	NA
AE008918	629152	nsyn	C	T	R	C	BMEII0595	NA
AE008918	629336	nsyn	G	A	G	D	BMEII0595	NA
AE008918	630867	intergenic	C	A	-	-	-	-
AE008918	632125	nsyn	C	G	R	A	BMEII0598	Exopolyphosphatase
AE008918	632126	nsyn	G	C	R	A	BMEII0598	Exopolyphosphatase
AE008918	632138	nsyn	C	G	V	L	BMEII0598	Exopolyphosphatase
AE008918	632139	nsyn	G	C	F	L	BMEII0598	Exopolyphosphatase
AE008918	635969	intergenic	A	G	-	-	-	-
AE008918	636065	intergenic	A	C	-	-	-	-
AE008918	637381	syn	G	A	L	L	BMEII0602	N-methylhydantoinase A/acetone carboxylase, beta subunit
AE008918	638774	syn	A	G	V	V	BMEII0602	N-methylhydantoinase A/acetone carboxylase, beta subunit
AE008918	639790	intergenic	C	T	-	-	-	-
AE008918	640958	syn	G	A	R	R	BMEII0604	ABC-type enterochelin transport system, ATPase component
AE008918	644368	intergenic	T	C	-	-	-	-
AE008918	646004	intergenic	G	A	-	-	-	-
AE008918	646755	nsyn	A	C	F	V	BMEII0609	NA
AE008918	646841	nsyn	T	C	S	G	BMEII0610	Predicted oxidoreductase
AE008918	648118	nsyn	A	T	L	H	BMEII0610	Predicted oxidoreductase
AE008918	648901	nsyn	G	A	R	C	BMEII0611	Predicted permeases
AE008918	651209	syn	A	G	V	V	BMEII0614	Permeases of the drug/metabolite transporter (DMT) superfamily

AE008918	651907	intergenic	T	C	-	-	-	-
AE008918	653292	nsyn	C	G	P	R	BMEII0617	Permeases
AE008918	653293	nsyn	G	C	P	R	BMEII0617	Permeases
AE008918	653798	nsyn	C	G	P	A	BMEII0618	Permeases
AE008918	653905	syn	G	C	L	L	BMEII0618	Permeases
AE008918	655176	nsyn	T	G	D	A	BMEII0620	Di- and tricarboxylate transporters
AE008918	656118	nsyn	C	T	S	N	BMEII0620	Di- and tricarboxylate transporters
AE008918	656405	syn	A	G	N	N	BMEII0620	Di- and tricarboxylate transporters
AE008918	656768	intergenic	C	T	-	-	-	-
AE008918	657013	nsyn	G	A	R	C	BMEII0621	ABC-type sugar transport systems, ATPase components
AE008918	657366	nsyn	A	G	I	T	BMEII0621	ABC-type sugar transport systems, ATPase components
AE008918	658132	syn	T	G	I	I	BMEII0622	ABC-type sugar transport system, permease component
AE008918	659674	intergenic	A	G	-	-	-	-
AE008918	660156	nsyn	C	G	S	T	BMEII0625	ABC-type sugar transport system, periplasmic component
AE008918	661171	nsyn	T	C	I	V	BMEII0625	ABC-type sugar transport system, periplasmic component
AE008918	663848	syn	C	T	T	T	BMEII0627	Adenine deaminase
AE008918	665120	nsyn	A	G	F	L	BMEII0628	ABC-type branched-chain amino acid transport systems, ATPase component
AE008918	665436	nsyn	C	T	R	H	BMEII0629	ABC-type branched-chain amino acid transport systems, ATPase component
AE008918	666318	intergenic	G	A	-	-	-	-
AE008918	666666	nsyn	T	A	H	L	BMEII0631	ABC-type branched-chain amino acid transport system, permease component
AE008918	668513	nsyn	C	T	R	H	BMEII0633	ABC-type branched-chain amino acid transport systems, periplasmic component
AE008918	669486	nsyn	C	T	G	S	BMEII0634	Adenylosuccinate lyase
AE008918	669766	syn	T	A	P	P	BMEII0634	Adenylosuccinate lyase
AE008918	672217	syn	T	A	T	T	BMEII0638	Predicted hydrolases or acyltransferases (alpha/beta hydrolase superfamily)
AE008918	672324	nsyn	C	T	A	T	BMEII0638	Predicted hydrolases or acyltransferases (alpha/beta hydrolase superfamily)
AE008918	673034	nsyn	T	G	V	G	BMEII0639	Transcriptional regulator
AE008918	673988	intergenic	T	A	-	-	-	-
AE008918	673990	intergenic	A	G	-	-	-	-
AE008918	673991	intergenic	A	C	-	-	-	-
AE008918	673997	intergenic	A	G	-	-	-	-

AE008918	674000	intergenic	T	G	-	-	-	-
AE008918	674002	intergenic	T	C	-	-	-	-
AE008918	674004	intergenic	C	T	-	-	-	-
AE008918	674005	intergenic	C	G	-	-	-	-
AE008918	674012	intergenic	T	G	-	-	-	-
AE008918	674015	intergenic	A	G	-	-	-	-
AE008918	674018	intergenic	T	G	-	-	-	-
AE008918	674785	nsyn	C	T	C	Y	BMEII0640	2-polyprenyl-6-methoxyphenol hydroxylase and related FAD-dependent oxidoreductases
AE008918	678470	nsyn	T	G	V	G	BMEII0645	Acyl CoA:acetate/3-ketoacid CoA transferase, beta subunit
AE008918	678902	syn	T	C	G	G	BMEII0646	Acetyl-CoA acetyltransferase
AE008918	678950	syn	A	G	L	L	BMEII0646	Acetyl-CoA acetyltransferase
AE008918	680693	intergenic	T	G	-	-	-	-
AE008918	682658	intergenic	T	A	-	-	-	-
AE008918	683856	intergenic	T	C	-	-	-	-
AE008918	683857	intergenic	G	C	-	-	-	-
AE008918	683859	intergenic	G	A	-	-	-	-
AE008918	683863	intergenic	T	C	-	-	-	-
AE008918	683866	intergenic	C	G	-	-	-	-
AE008918	683868	intergenic	C	G	-	-	-	-
AE008918	683869	intergenic	A	G	-	-	-	-
AE008918	683881	intergenic	T	C	-	-	-	-
AE008918	684088	intergenic	G	A	-	-	-	-
AE008918	684714	nsyn	C	T	P	L	BMEII0654	FOG: GGDEF domain
AE008918	684995	nsyn	A	T	S	C	BMEII0654	FOG: GGDEF domain
AE008918	688091	intergenic	C	T	-	-	-	-
AE008918	690813	nsyn	T	C	T	A	BMEII0658	NA
AE008918	693831	nsyn	T	C	M	V	BMEII0662	Permeases of the major facilitator superfamily
AE008918	694371	nsyn	C	T	A	T	BMEII0662	Permeases of the major facilitator superfamily
AE008918	707163	nsyn	A	G	V	A	BMEII0672	Predicted endonuclease involved in recombination (possible Holliday junction resolvase in Mycoplasmas and B. subtilis)
AE008918	708485	nsyn	C	A	D	E	BMEII0674	Asp-tRNAAsn/Glu-tRNAGln amidotransferase C subunit
AE008918	709839	syn	G	A	E	E	BMEII0675	Asp-tRNAAsn/Glu-tRNAGln amidotransferase A subunit and related amidases

AE008918	710075	nsyn	C	T	T	I	BMEII0675	Asp-tRNAAsn/Glu-tRNAGln amidotransferase A subunit and related amidases
AE008918	712952	intergenic	G	A	-	-	-	-
AE008918	713391	nsyn	T	A	L	M	BMEII0677	Permeases of the drug/metabolite transporter (DMT) superfamily
AE008918	714650	nsyn	C	T	A	T	BMEII0678	Lipoate-protein ligase B
AE008918	714690	syn	C	A	L	L	BMEII0678	Lipoate-protein ligase B
AE008918	716693	intergenic	T	C	-	-	-	-
AE008918	719746	syn	T	C	L	L	BMEII0682	Uncharacterized conserved protein
AE008918	721416	nsyn	C	G	A	P	BMEII0682	Uncharacterized conserved protein
AE008918	721705	intergenic	T	G	-	-	-	-
AE008918	722437	syn	C	T	G	G	BMEII0683	Cytochrome c biogenesis protein
AE008918	724781	syn	C	T	D	D	BMEII0685	Glucosamine 6-phosphate synthetase, contains amidotransferase and phosphosugar isomerase domains
AE008918	727633	syn	C	T	P	P	BMEII0686	RecG-like helicase
AE008918	730527	nsyn	G	A	G	D	BMEII0688	Transcription-repair coupling factor (superfamily II helicase)
AE008918	730988	nsyn	T	C	W	R	BMEII0688	Transcription-repair coupling factor (superfamily II helicase)
AE008918	733982	syn	A	G	R	R	BMEII0691	ABC-type oligopeptide transport system, periplasmic component
AE008918	734214	nsyn	C	G	G	A	BMEII0691	ABC-type oligopeptide transport system, periplasmic component
AE008918	735964	intergenic	T	C	-	-	-	-
AE008918	736059	syn	C	T	N	N	BMEII0692	Invasion protein B, involved in pathogenesis
AE008918	739840	syn	C	T	R	R	BMEII0696	NADPH:quinone reductase and related Zn-dependent oxidoreductases
AE008918	740239	nsyn	C	A	F	L	BMEII0697	NADPH:quinone reductase and related Zn-dependent oxidoreductases
AE008918	741002	syn	T	C	P	P	BMEII0698	ABC-type uncharacterized transport systems, ATPase components
AE008918	742788	nsyn	A	G	E	G	BMEII0699	ABC-type uncharacterized transport system, permease component
AE008918	744618	nsyn	A	T	I	F	BMEII0702	Uncharacterized ABC-type transport system, periplasmic component/surface lipoprotein
AE008918	744645	nsyn	G	A	V	M	BMEII0702	Uncharacterized ABC-type transport system, periplasmic component/surface lipoprotein
AE008918	747148	nsyn	A	G	F	L	BMEII0704	Bacterioferritin (cytochrome b1)
AE008918	747302	nsyn	A	C	D	E	BMEII0704	Bacterioferritin (cytochrome b1)
AE008918	747768	intergenic	A	C	-	-	-	-
AE008918	751343	nsyn	A	C	D	A	BMEII0710	Transposase and inactivated derivatives

AE008918	751386	syn	G	A	P	P	BMEII0710	Transposase and inactivated derivatives
AE008918	753281	syn	T	C	R	R	BMEII0713	Transposase and inactivated derivatives
AE008918	753282	nsyn	G	T	A	S	BMEII0713	Transposase and inactivated derivatives
AE008918	757304	nsyn	A	G	E	G	BMEII0717	NA
AE008918	757305	nsyn	A	G	E	G	BMEII0717	NA
AE008918	757576	syn	C	A	R	R	BMEII0718	Transposase and inactivated derivatives
AE008918	757654	syn	C	T	D	D	BMEII0718	Transposase and inactivated derivatives
AE008918	757696	nsyn	A	G	K	R	BMEII0718	Transposase and inactivated derivatives
AE008918	757734	nsyn	G	T	G	V	BMEII0718	Transposase and inactivated derivatives
AE008918	757881	syn	G	A	A	A	BMEII0719	Transposase and inactivated derivatives
AE008918	757921	nsyn	G	A	D	N	BMEII0719	Transposase and inactivated derivatives
AE008918	757950	syn	C	T	G	G	BMEII0719	Transposase and inactivated derivatives
AE008918	757971	syn	C	T	C	C	BMEII0719	Transposase and inactivated derivatives
AE008918	758050	intergenic	T	C	-	-	-	-
AE008918	758507	intergenic	T	G	-	-	-	-
AE008918	758510	intergenic	A	G	-	-	-	-
AE008918	759388	nsyn	C	A	H	N	BMEII0721	AraC-type DNA-binding domain-containing proteins
AE008918	759596	nsyn	A	G	H	R	BMEII0721	AraC-type DNA-binding domain-containing proteins
AE008918	760184	intergenic	G	A	-	-	-	-
AE008918	761466	nsyn	G	A	S	L	BMEII0724	Beta-mannanase
AE008918	762336	nsyn	G	A	P	L	BMEII0726	NA
AE008918	762424	nsyn	G	A	A	V	BMEII0727	Predicted UDP-glucose 6-dehydrogenase
AE008918	763678	nsyn	C	A	G	V	BMEII0727	Predicted UDP-glucose 6-dehydrogenase
AE008918	764244	nsyn	G	A	A	V	BMEII0728	Glycosyltransferases, probably involved in cell wall biogenesis
AE008918	764954	syn	A	G	S	S	BMEII0728	Glycosyltransferases, probably involved in cell wall biogenesis
AE008918	765912	nsyn	A	G	I	T	BMEII0730	UDP-glucose 4-epimerase
AE008918	767356	nsyn	T	C	V	A	BMEII0732	NA
AE008918	769242	nsyn	T	C	F	S	BMEII0734	ABC-type oligopeptide transport system, periplasmic component
AE008918	769615	syn	C	G	L	L	BMEII0734	ABC-type oligopeptide transport system, periplasmic component
AE008918	769755	nsyn	A	G	H	R	BMEII0734	ABC-type oligopeptide transport system, periplasmic component
AE008918	770053	syn	C	T	D	D	BMEII0735	ABC-type oligopeptide transport system, periplasmic component
AE008918	770484	nsyn	T	C	I	T	BMEII0735	ABC-type oligopeptide transport system, periplasmic component
AE008918	771619	intergenic	G	C	-	-	-	-
AE008918	771903	nsyn	C	T	H	Y	BMEII0736	ABC-type dipeptide/oligopeptide/nickel transport systems, permease components

AE008918	773328	syn	C	T	I	I	BMEII0737	ABC-type dipeptide/oligopeptide/nickel transport systems, permease components
AE008918	774253	syn	C	T	A	A	BMEII0738	ABC-type uncharacterized transport system, duplicated ATPase component
AE008918	779587	intergenic	G	C	-	-	-	-
AE008918	781191	syn	T	C	G	G	BMEII0742	DNA segregation ATPase FtsK/SpoIIIE and related proteins
AE008918	783388	nsyn	T	C	*	W	BMEII0745	Pyruvate/2-oxoglutarate dehydrogenase complex, dihydrolipoamide dehydrogenase (E3) component, and related enzymes
AE008918	784221	nsyn	G	A	R	C	BMEII0745	Pyruvate/2-oxoglutarate dehydrogenase complex, dihydrolipoamide dehydrogenase (E3) component, and related enzymes
AE008918	785528	syn	A	G	I	I	BMEII0746	Pyruvate/2-oxoglutarate dehydrogenase complex, dihydrolipoamide acyltransferase (E2) component, and related enzymes
AE008918	785922	nsyn	G	A	T	I	BMEII0747	Pyruvate/2-oxoglutarate dehydrogenase complex, dehydrogenase (E1) component, eukaryotic type, beta subunit
AE008918	786928	nsyn	G	T	T	K	BMEII0748	Pyruvate/2-oxoglutarate dehydrogenase complex, dehydrogenase (E1) component, eukaryotic type, alpha subunit
AE008918	788611	intergenic	G	C	-	-	-	-
AE008918	788613	intergenic	G	C	-	-	-	-
AE008918	788618	intergenic	T	G	-	-	-	-
AE008918	788619	intergenic	C	G	-	-	-	-
AE008918	788620	intergenic	A	G	-	-	-	-
AE008918	788623	intergenic	C	G	-	-	-	-
AE008918	788625	intergenic	C	G	-	-	-	-
AE008918	788775	syn	A	C	G	G	BMEII0750	ABC-type sugar transport systems, ATPase components
AE008918	789245	nsyn	G	A	R	C	BMEII0750	ABC-type sugar transport systems, ATPase components
AE008918	789745	nsyn	C	G	G	R	BMEII0751	Predicted dehydrogenases and related proteins
AE008918	790156	nsyn	T	G	M	L	BMEII0751	Predicted dehydrogenases and related proteins
AE008918	790252	nsyn	T	C	N	D	BMEII0751	Predicted dehydrogenases and related proteins
AE008918	793567	syn	C	T	E	E	BMEII0754	ABC-type sugar transport system, periplasmic component
AE008918	793973	nsyn	A	G	V	A	BMEII0755	ABC-type sugar transport system, periplasmic component
AE008918	794616	syn	C	T	G	G	BMEII0756	Predicted N-acetylglucosamine kinase
AE008918	795512	syn	A	G	S	S	BMEII0757	Transcriptional regulators
AE008918	797501	intergenic	G	A	-	-	-	-

AE008918	799250	intergenic	T	C	-	-	-	-
AE008918	799561	syn	G	C	A	A	BMEII0761	ABC-type transport system involved in cytochrome bd biosynthesis, fused ATPase and permease components
AE008918	803568	intergenic	C	T	-	-	-	-
AE008918	804508	syn	T	G	T	T	BMEII0767	Multisubunit Na+/H+ antiporter, MnhE subunit
AE008918	805112	nsyn	T	C	T	A	BMEII0768	Formate hydrogenlyase subunit 3/Multisubunit Na+/H+ antiporter, MnhD subunit
AE008918	805448	nsyn	G	T	P	T	BMEII0768	Formate hydrogenlyase subunit 3/Multisubunit Na+/H+ antiporter, MnhD subunit
AE008918	805493	nsyn	T	A	T	S	BMEII0768	Formate hydrogenlyase subunit 3/Multisubunit Na+/H+ antiporter, MnhD subunit
AE008918	805724	nsyn	T	G	T	P	BMEII0768	Formate hydrogenlyase subunit 3/Multisubunit Na+/H+ antiporter, MnhD subunit
AE008918	807504	nsyn	T	G	S	R	BMEII0770	NADH:ubiquinone oxidoreductase subunit 5 (chain L)/Multisubunit Na+/H+ antiporter, MnhA subunit
AE008918	807522	nsyn	T	C	T	A	BMEII0770	NADH:ubiquinone oxidoreductase subunit 5 (chain L)/Multisubunit Na+/H+ antiporter, MnhA subunit
AE008918	807878	nsyn	G	A	P	L	BMEII0770	NADH:ubiquinone oxidoreductase subunit 5 (chain L)/Multisubunit Na+/H+ antiporter, MnhA subunit
AE008918	809042	nsyn	A	C	L	R	BMEII0770	NADH:ubiquinone oxidoreductase subunit 5 (chain L)/Multisubunit Na+/H+ antiporter, MnhA subunit
AE008918	810595	nsyn	C	T	W	*	BMEII0771	Uncharacterized protein conserved in bacteria
AE008918	813354	nsyn	A	G	I	V	BMEII0774	NA
AE008918	815553	nsyn	C	A	H	N	BMEII0776	7-keto-8-aminopelargonate synthetase and related enzymes
AE008918	815915	syn	A	G	P	P	BMEII0776	7-keto-8-aminopelargonate synthetase and related enzymes
AE008918	818114	syn	C	A	A	A	BMEII0779	3-oxoacyl-[acyl-carrier-protein] synthase III
AE008918	820285	nsyn	T	C	L	P	BMEII0781	Homoserine trans-succinylase
AE008918	821052	nsyn	G	A	Q	*	BMEII0782	Lyzozyme M1 (1,4-beta-N-acetylmuramidase)
AE008918	821652	intergenic	T	C	-	-	-	-
AE008918	824983	nsyn	C	G	A	G	BMEII0786	NADH dehydrogenase, FAD-containing subunit
AE008918	828828	syn	G	A	P	P	BMEII0792	Signal transduction histidine kinase
AE008918	830398	nsyn	G	T	T	K	BMEII0793	Multidrug resistance efflux pump
AE008918	834303	nsyn	G	T	A	S	BMEII0797	ABC-type nitrate/sulfonate/bicarbonate transport systems, periplasmic components
AE008918	839070	nsyn	A	G	L	P	BMEII0803	Membrane-fusion protein
AE008918	839518	nsyn	T	C	T	A	BMEII0803	Membrane-fusion protein
AE008918	840072	syn	G	A	I	I	BMEII0804	Transcriptional regulator

AE008918	841382	nsyn	T	C	I	V	BMEII0806	Predicted permease
AE008918	842276	intergenic	C	T	-	-	-	-
AE008918	843641	nsyn	C	A	A	S	BMEII0808	Predicted transporter component
AE008918	844292	intergenic	T	G	-	-	-	-
AE008918	844321	intergenic	C	T	-	-	-	-
AE008918	844328	intergenic	A	C	-	-	-	-
AE008918	844491	intergenic	A	C	-	-	-	-
AE008918	845559	intergenic	C	G	-	-	-	-
AE008918	847538	nsyn	G	A	R	C	BMEII0813	Phosphoglycerate dehydrogenase and related dehydrogenases
AE008918	848504	nsyn	G	A	S	F	BMEII0814	AraC-type DNA-binding domain-containing proteins
AE008918	848918	nsyn	T	C	H	R	BMEII0814	AraC-type DNA-binding domain-containing proteins
AE008918	849479	nsyn	A	G	H	R	BMEII0815	Acyl-coenzyme A synthetases/AMP-(fatty) acid ligases
AE008918	850416	syn	C	G	S	S	BMEII0815	Acyl-coenzyme A synthetases/AMP-(fatty) acid ligases
AE008918	852366	nsyn	T	G	Y	D	BMEII0817	Acetyl-CoA acetyltransferase
AE008918	852816	nsyn	A	G	M	V	BMEII0817	Acetyl-CoA acetyltransferase
AE008918	854076	nsyn	T	G	M	R	BMEII0818	Acyl-CoA dehydrogenases
AE008918	855274	syn	T	C	L	L	BMEII0820	Transcriptional regulator
AE008918	857638	syn	G	A	T	T	BMEII0824	Glycerol kinase
AE008918	860175	intergenic	A	G	-	-	-	-
AE008918	860827	syn	A	G	D	D	BMEII0826	Uncharacterized proteins, LmbE homologs
AE008918	861596	nsyn	C	T	E	K	BMEII0827	Nucleoside-diphosphate-sugar pyrophosphorylase involved in lipopolysaccharide biosynthesis/translation initiation factor 2B, gamma/epsilon subunits (eIF-2Bgamma/eIF-2Bepsilon)
AE008918	862700	nsyn	C	A	C	F	BMEII0829	SAM-dependent methyltransferases
AE008918	863287	nsyn	T	G	H	Q	BMEII0830	dTDP-4-dehydrorhamnose 3,5-epimerase and related enzymes
AE008918	863837	nsyn	T	A	I	F	BMEII0831	Uncharacterized protein conserved in bacteria with an aminopeptidase-like domain
AE008918	864489	syn	A	G	G	G	BMEII0831	Uncharacterized protein conserved in bacteria with an aminopeptidase-like domain
AE008918	865127	syn	C	A	S	S	BMEII0832	Nucleoside-diphosphate-sugar epimerases
AE008918	868288	nsyn	T	A	Q	H	BMEII0834	Glutamate-1-semialdehyde aminotransferase
AE008918	868798	syn	A	G	D	D	BMEII0834	Glutamate-1-semialdehyde aminotransferase
AE008918	869550	nsyn	A	G	V	A	BMEII0835	Glycosyltransferase
AE008918	869896	nsyn	C	G	V	L	BMEII0835	Glycosyltransferase
AE008918	871240	syn	C	A	R	R	BMEII0836	Uncharacterized conserved protein

AE008918	874925	nsyn	A	C	M	L	BMEII0839	UDP-N-acetylmuramyl pentapeptide phosphotransferase/ UDP-N-acetylglucosamine-1-phosphate transferase
AE008918	877879	syn	A	G	L	L	BMEII0842	NA
AE008918	878128	syn	C	T	F	F	BMEII0842	NA
AE008918	879206	intergenic	G	A	-	-	-	-
AE008918	879283	intergenic	T	C	-	-	-	-
AE008918	880151	intergenic	T	C	-	-	-	-
AE008918	880161	intergenic	A	G	-	-	-	-
AE008918	880262	nsyn	A	C	H	Q	BMEII0845	Glycosyltransferase
AE008918	882122	nsyn	G	A	T	M	BMEII0846	Glycosyltransferase
AE008918	883733	nsyn	G	A	R	C	BMEII0847	Glycosyltransferase
AE008918	884029	intergenic	G	T	-	-	-	-
AE008918	885049	syn	A	G	L	L	BMEII0848	GDP-D-mannose dehydratase
AE008918	885420	nsyn	A	G	N	D	BMEII0849	Nucleoside-diphosphate-sugar epimerases
AE008918	885566	syn	A	G	E	E	BMEII0849	Nucleoside-diphosphate-sugar epimerases
AE008918	886412	nsyn	A	C	M	R	BMEII0851	Periplasmic protein involved in polysaccharide export
AE008918	887454	intergenic	C	T	-	-	-	-
AE008918	888409	syn	T	C	Q	Q	BMEII0852	Uncharacterized protein involved in exopolysaccharide biosynthesis
AE008918	890149	nsyn	A	T	D	E	BMEII0853	Response regulator containing a CheY-like receiver domain and an HTH DNA-binding domain
AE008918	890238	nsyn	G	A	R	C	BMEII0853	Response regulator containing a CheY-like receiver domain and an HTH DNA-binding domain
AE008918	890504	intergenic	C	T	-	-	-	-
AE008918	890590	intergenic	T	C	-	-	-	-
AE008918	890786	intergenic	A	G	-	-	-	-
AE008918	892522	syn	A	G	Y	Y	BMEII0856	Uncharacterized protein conserved in bacteria
AE008918	893039	nsyn	A	G	L	P	BMEII0856	Uncharacterized protein conserved in bacteria
AE008918	896032	syn	T	C	I	I	BMEII0859	ABC-type dipeptide transport system, periplasmic component
AE008918	898679	syn	G	A	L	L	BMEII0861	ABC-type dipeptide/oligopeptide/nickel transport systems, permease components
AE008918	899064	syn	T	C	L	L	BMEII0861	ABC-type dipeptide/oligopeptide/nickel transport systems, permease components
AE008918	899678	nsyn	G	A	C	Y	BMEII0862	Dihydrodipicolinate synthase/N-acetylneuraminate lyase
AE008918	900809	nsyn	G	C	V	L	BMEII0863	ABC-type dipeptide/oligopeptide/nickel transport system, ATPase component

AE008918	901055	nsyn	A	G	K	E	BMEII0863	ABC-type dipeptide/oligopeptide/nickel transport system, ATPase component
AE008918	902962	nsyn	G	A	L	F	BMEII0865	Predicted dehydrogenases and related proteins
AE008918	904827	syn	G	A	G	G	BMEII0867	Zn-dependent alcohol dehydrogenases, class III
AE008918	906349	nsyn	G	C	L	V	BMEII0868	ABC-type branched-chain amino acid transport systems, periplasmic component
AE008918	907892	nsyn	A	C	S	A	BMEII0869	NAD-dependent aldehyde dehydrogenases
AE008918	910876	nsyn	A	G	W	R	BMEII0872	Dehydrogenases with different specificities (related to short-chain alcohol dehydrogenases)
AE008918	911719	nsyn	C	T	M	I	BMEII0873	ABC-type branched-chain amino acid transport systems, ATPase component
AE008918	914379	nsyn	T	C	H	R	BMEII0874	Branched-chain amino acid ABC-type transport system, permease components
AE008918	915070	nsyn	C	T	A	T	BMEII0875	ABC-type branched-chain amino acid transport systems, periplasmic component
AE008918	916396	syn	C	T	A	A	BMEII0876	NADPH:quinone reductase and related Zn-dependent oxidoreductases
AE008918	917110	syn	A	G	A	A	BMEII0876	NADPH:quinone reductase and related Zn-dependent oxidoreductases
AE008918	917456	intergenic	T	C	-	-	-	-
AE008918	918195	intergenic	A	G	-	-	-	-
AE008918	918376	nsyn	C	T	G	E	BMEII0879	Cytochrome P450
AE008918	924215	nsyn	A	C	L	V	BMEII0882	Polyferredoxin
AE008918	924378	syn	A	C	G	G	BMEII0882	Polyferredoxin
AE008918	924814	nsyn	C	G	R	A	BMEII0882	Polyferredoxin
AE008918	924815	nsyn	G	C	R	A	BMEII0882	Polyferredoxin
AE008918	924858	nsyn	C	G	P	R	BMEII0882	Polyferredoxin
AE008918	924859	nsyn	G	C	P	R	BMEII0882	Polyferredoxin
AE008918	926837	syn	T	C	Q	Q	BMEII0885	Uncharacterized protein probably involved in high-affinity Fe2+ transport
AE008918	929247	nsyn	C	T	A	T	BMEII0888	Nucleoside phosphorylase
AE008918	930065	nsyn	G	A	H	Y	BMEII0889	Uncharacterized protein, possibly involved in aromatic compounds catabolism
AE008918	930082	nsyn	C	T	R	H	BMEII0889	Uncharacterized protein, possibly involved in aromatic compounds catabolism
AE008918	930104	nsyn	T	C	K	E	BMEII0889	Uncharacterized protein, possibly involved in aromatic compounds catabolism

AE008918	930106	nsyn	G	T	P	Q	BMEII0889	Uncharacterized protein, possibly involved in aromatic compounds catabolism
AE008918	930114	syn	G	A	S	S	BMEII0889	Uncharacterized protein, possibly involved in aromatic compounds catabolism
AE008918	930134	nsyn	A	G	F	L	BMEII0889	Uncharacterized protein, possibly involved in aromatic compounds catabolism
AE008918	930138	syn	T	C	E	E	BMEII0889	Uncharacterized protein, possibly involved in aromatic compounds catabolism
AE008918	930142	nsyn	C	T	G	E	BMEII0889	Uncharacterized protein, possibly involved in aromatic compounds catabolism
AE008918	930144	nsyn	T	C	K	M	BMEII0889	Uncharacterized protein, possibly involved in aromatic compounds catabolism
AE008918	930145	nsyn	T	A	K	M	BMEII0889	Uncharacterized protein, possibly involved in aromatic compounds catabolism
AE008918	930149	nsyn	C	G	V	L	BMEII0889	Uncharacterized protein, possibly involved in aromatic compounds catabolism
AE008918	930163	nsyn	G	A	T	M	BMEII0889	Uncharacterized protein, possibly involved in aromatic compounds catabolism
AE008918	930165	nsyn	G	T	C	*	BMEII0889	Uncharacterized protein, possibly involved in aromatic compounds catabolism
AE008918	930170	nsyn	A	G	C	R	BMEII0889	Uncharacterized protein, possibly involved in aromatic compounds catabolism
AE008918	930172	nsyn	A	G	F	S	BMEII0889	Uncharacterized protein, possibly involved in aromatic compounds catabolism
AE008918	930191	nsyn	G	C	Q	E	BMEII0889	Uncharacterized protein, possibly involved in aromatic compounds catabolism
AE008918	930209	nsyn	G	A	H	Y	BMEII0889	Uncharacterized protein, possibly involved in aromatic compounds catabolism
AE008918	930226	intergenic	C	G	-	-	-	-
AE008918	930234	intergenic	T	C	-	-	-	-
AE008918	930240	intergenic	T	G	-	-	-	-
AE008918	930242	intergenic	G	A	-	-	-	-
AE008918	930254	intergenic	T	C	-	-	-	-
AE008918	930265	intergenic	A	G	-	-	-	-
AE008918	930281	intergenic	C	T	-	-	-	-
AE008918	930294	intergenic	G	A	-	-	-	-
AE008918	932407	nsyn	A	G	V	A	BMEII0891	Disulfide bond formation protein DsbB

AE008918	934798	nsyn	A	G	N	S	BMEII0894	Transcriptional regulator
AE008918	936592	intergenic	G	C	-	-	-	-
AE008918	937802	syn	G	A	T	T	BMEII0896	IMP dehydrogenase/GMP reductase
AE008918	940967	intergenic	C	T	-	-	-	-
AE008918	941278	syn	C	T	G	G	BMEII0899	Phosphomannomutase
AE008918	944160	nsyn	C	T	V	M	BMEII0901	NA
AE008918	945642	intergenic	A	T	-	-	-	-
AE008918	945717	nsyn	A	G	I	V	BMEII0904	Predicted signal transduction protein containing sensor and EAL domains
AE008918	946772	syn	G	T	T	T	BMEII0904	Predicted signal transduction protein containing sensor and EAL domains
AE008918	947592	intergenic	A	T	-	-	-	-
AE008918	949981	nsyn	A	G	V	A	BMEII0909	Amino acid transporters
AE008918	953913	nsyn	G	T	E	*	BMEII0914	Membrane-fusion protein
AE008918	954757	nsyn	C	A	T	K	BMEII0914	Membrane-fusion protein
AE008918	954909	nsyn	A	G	T	A	BMEII0914	Membrane-fusion protein
AE008918	955416	nsyn	A	G	N	D	BMEII0915	Cation/multidrug efflux pump
AE008918	957194	nsyn	T	C	I	T	BMEII0916	Cation/multidrug efflux pump
AE008918	958713	nsyn	C	G	Q	E	BMEII0917	NA
AE008918	959127	syn	C	T	V	V	BMEII0918	NA
AE008918	964064	nsyn	G	C	P	A	BMEII0923	Spermidine/putrescine-binding periplasmic protein
AE008918	965566	intergenic	G	A	-	-	-	-
AE008918	967141	syn	C	T	Q	Q	BMEII0927	Septum formation inhibitor
AE008918	968217	intergenic	T	A	-	-	-	-
AE008918	970342	syn	G	A	N	N	BMEII0930	Ribonucleotide reductase, alpha subunit
AE008918	970813	syn	A	C	V	V	BMEII0930	Ribonucleotide reductase, alpha subunit
AE008918	975700	intergenic	G	A	-	-	-	-
AE008918	976922	nsyn	C	T	V	I	BMEII0939	Trehalose utilization protein
AE008918	977040	nsyn	G	T	H	Q	BMEII0939	Trehalose utilization protein
AE008918	978727	syn	A	C	G	G	BMEII0942	ABC-type sugar transport system, permease component
AE008918	979156	syn	G	A	F	F	BMEII0942	ABC-type sugar transport system, permease component
AE008918	981114	nsyn	G	A	S	L	BMEII0945	ABC-type sugar transport system, periplasmic component
AE008918	982418	nsyn	A	C	E	A	BMEII0946	Transcriptional regulators
AE008918	982841	nsyn	C	A	A	D	BMEII0946	Transcriptional regulators
AE008918	983117	nsyn	G	C	C	S	BMEII0946	Transcriptional regulators
AE008918	983214	syn	A	T	R	R	BMEII0946	Transcriptional regulators

AE008918	983481	nsyn	A	T	S	T	BMEII0947	cAMP-binding proteins - catabolite gene activator and regulatory subunit of cAMP-dependent protein kinases
AE008918	984394	nsyn	A	G	S	G	BMEII0948	Nitrate/nitrite transporter
AE008918	984446	nsyn	T	C	V	A	BMEII0948	Nitrate/nitrite transporter
AE008918	986597	nsyn	C	T	A	V	BMEII0948	Nitrate/nitrite transporter
AE008918	988902	syn	T	C	T	T	BMEII0950	Nitrate reductase alpha subunit
AE008918	989490	syn	G	C	G	G	BMEII0950	Nitrate reductase alpha subunit
AE008918	990822	nsyn	A	G	T	A	BMEII0951	Nitrate reductase beta subunit
AE008918	991722	nsyn	C	A	L	M	BMEII0951	Nitrate reductase beta subunit
AE008918	991833	nsyn	T	C	F	L	BMEII0951	Nitrate reductase beta subunit
AE008918	992173	nsyn	T	A	V	D	BMEII0951	Nitrate reductase beta subunit
AE008918	993014	intergenic	A	G	-	-	-	-
AE008918	993850	intergenic	A	T	-	-	-	-
AE008918	996260	nsyn	G	A	P	S	BMEII0957	3-polyprenyl-4-hydroxybenzoate decarboxylase and related decarboxylases
AE008918	998441	nsyn	T	C	V	A	BMEII0959	Collagenase and related proteases
AE008918	999288	nsyn	A	G	S	G	BMEII0960	Collagenase and related proteases
AE008918	1000558	nsyn	G	A	G	S	BMEII0961	ABC-type nitrate/sulfonate/bicarbonate transport system, ATPase component
AE008918	1000681	nsyn	C	T	Q	*	BMEII0961	ABC-type nitrate/sulfonate/bicarbonate transport system, ATPase component
AE008918	1000853	nsyn	T	C	V	A	BMEII0961	ABC-type nitrate/sulfonate/bicarbonate transport system, ATPase component
AE008918	1002069	nsyn	T	C	L	P	BMEII0963	ABC-type nitrate/sulfonate/bicarbonate transport systems, periplasmic components
AE008918	1002558	nsyn	A	C	Q	P	BMEII0963	ABC-type nitrate/sulfonate/bicarbonate transport systems, periplasmic components
AE008918	1003833	nsyn	G	T	H	Q	BMEII0964	Uncharacterized protein involved in response to NO
AE008918	1004482	nsyn	T	C	S	G	BMEII0965	Plastocyanin
AE008918	1004747	syn	G	A	A	A	BMEII0965	Plastocyanin
AE008918	1006558	nsyn	T	C	Q	R	BMEII0967	Membrane-associated lipoprotein involved in thiamine biosynthesis
AE008918	1006952	nsyn	C	T	E	K	BMEII0969	Predicted lipoprotein involved in nitrous oxide reduction
AE008918	1009829	syn	G	A	N	N	BMEII0972	Nitrous oxidase accessory protein
AE008918	1011037	syn	G	A	G	G	BMEII0973	Nitrous oxide reductase
AE008918	1011431	nsyn	A	G	L	P	BMEII0973	Nitrous oxide reductase
AE008918	1012636	nsyn	T	C	Y	C	BMEII0975	Regulator of nitric oxide reductase transcription

AE008918	1013267	nsyn	G	T	H	N	BMEII0975	Regulator of nitric oxide reductase transcription
AE008918	1013596	nsyn	C	T	S	N	BMEII0975	Regulator of nitric oxide reductase transcription
AE008918	1014819	intergenic	G	A	-	-	-	-
AE008918	1015022	intergenic	C	G	-	-	-	-
AE008918	1015174	intergenic	A	G	-	-	-	-
AE008918	1016854	nsyn	A	G	K	E	BMEII0976	ABC-type uncharacterized transport system, permease and ATPase components
AE008918	1019947	syn	A	G	G	G	BMEII0979	Ribulose kinase
AE008918	1020745	syn	T	C	Q	Q	BMEII0980	Short-chain alcohol dehydrogenase of unknown specificity
AE008918	1024369	syn	A	G	R	R	BMEII0983	ABC-type xylose transport system, periplasmic component
AE008918	1024882	syn	C	T	L	L	BMEII0984	NA
AE008918	1028074	intergenic	C	A	-	-	-	-
AE008918	1032007	intergenic	C	T	-	-	-	-
AE008918	1032008	intergenic	C	T	-	-	-	-
AE008918	1034855	intergenic	C	T	-	-	-	-
AE008918	1034923	syn	G	A	L	L	BMEII0996	Nitric oxide reductase activation protein
AE008918	1035039	nsyn	T	A	S	C	BMEII0996	Nitric oxide reductase activation protein
AE008918	1035687	nsyn	T	C	N	D	BMEII0996	Nitric oxide reductase activation protein
AE008918	1035761	nsyn	G	A	A	V	BMEII0996	Nitric oxide reductase activation protein
AE008918	1039495	syn	T	C	E	E	BMEII0999	Cytochrome c, mono- and diheme variants
AE008918	1041543	syn	A	G	L	L	BMEII1003	Membrane protein involved in the export of O-antigen and teichoic acid
AE008918	1041697	nsyn	G	C	T	S	BMEII1003	Membrane protein involved in the export of O-antigen and teichoic acid
AE008918	1041791	nsyn	C	T	D	N	BMEII1003	Membrane protein involved in the export of O-antigen and teichoic acid
AE008918	1042202	nsyn	A	C	L	V	BMEII1003	Membrane protein involved in the export of O-antigen and teichoic acid
AE008918	1042411	nsyn	C	G	G	A	BMEII1003	Membrane protein involved in the export of O-antigen and teichoic acid
AE008918	1043630	nsyn	A	C	I	L	BMEII1005	Malate/L-lactate dehydrogenases
AE008918	1044232	syn	C	T	P	P	BMEII1006	Dihydrodipicolinate synthase/N-acetylneuraminate lyase
AE008918	1044428	nsyn	G	T	A	D	BMEII1006	Dihydrodipicolinate synthase/N-acetylneuraminate lyase
AE008918	1044561	syn	A	G	L	L	BMEII1006	Dihydrodipicolinate synthase/N-acetylneuraminate lyase
AE008918	1047050	intergenic	A	G	-	-	-	-
AE008918	1047303	nsyn	A	G	I	M	BMEII1009	FOG: GAF domain

AE008918	1049477	syn	G	A	T	T	BMEII1011	Sulfite reductase, alpha subunit (flavoprotein)
AE008918	1051952	intergenic	T	C	-	-	-	-
AE008918	1053858	nsyn	G	A	A	T	BMEII1015	Signal transduction histidine kinase
AE008918	1054457	syn	T	G	V	V	BMEII1015	Signal transduction histidine kinase
AE008918	1054965	nsyn	C	T	S	L	BMEII1016	Putative intracellular protease/amidase
AE008918	1056247	nsyn	C	G	A	G	BMEII1018	NADH:flavin oxidoreductases, Old Yellow Enzyme family
AE008918	1056269	nsyn	C	G	N	K	BMEII1018	NADH:flavin oxidoreductases, Old Yellow Enzyme family
AE008918	1057072	nsyn	T	C	L	P	BMEII1018	NADH:flavin oxidoreductases, Old Yellow Enzyme family
AE008918	1058009	nsyn	A	G	V	A	BMEII1019	Predicted acyl-CoA transferases/carnitine dehydratase
AE008918	1063633	nsyn	G	A	A	T	BMEII1024	Permeases of the major facilitator superfamily
AE008918	1063651	nsyn	C	G	P	A	BMEII1024	Permeases of the major facilitator superfamily
AE008918	1063696	nsyn	C	T	L	F	BMEII1024	Permeases of the major facilitator superfamily
AE008918	1066220	syn	A	G	Q	Q	BMEII1026	DNA mismatch repair enzyme (predicted ATPase)
AE008918	1066229	nsyn	T	G	H	Q	BMEII1026	DNA mismatch repair enzyme (predicted ATPase)
AE008918	1066230	nsyn	C	A	P	T	BMEII1026	DNA mismatch repair enzyme (predicted ATPase)
AE008918	1066317	nsyn	G	T	A	S	BMEII1026	DNA mismatch repair enzyme (predicted ATPase)
AE008918	1067272	nsyn	A	G	T	A	BMEII1027	Uncharacterized protein conserved in bacteria
AE008918	1067343	nsyn	T	C	D	G	BMEII1028	Tetraacyldisaccharide-1-P 4'-kinase
AE008918	1068623	nsyn	G	C	A	G	BMEII1029	3-deoxy-D-manno-octulosonic-acid transferase
AE008918	1071882	nsyn	C	G	P	R	BMEII1033	Predicted Zn-dependent proteases and their inactivated homologs
AE008918	1071883	nsyn	G	C	P	R	BMEII1033	Predicted Zn-dependent proteases and their inactivated homologs
AE008918	1072105	nsyn	A	G	M	T	BMEII1033	Predicted Zn-dependent proteases and their inactivated homologs
AE008918	1072456	nsyn	G	A	T	I	BMEII1033	Predicted Zn-dependent proteases and their inactivated homologs
AE008918	1078448	nsyn	G	A	P	S	BMEII1037	Predicted Zn-dependent peptidases
AE008918	1080542	syn	A	G	L	L	BMEII1039	16S rRNA uridine-516 pseudouridylate synthase and related pseudouridylate synthases
AE008918	1080739	nsyn	T	G	K	T	BMEII1039	16S rRNA uridine-516 pseudouridylate synthase and related pseudouridylate synthases
AE008918	1081650	nsyn	C	T	P	L	BMEII1040	Cytosine/adenosine deaminases
AE008918	1083533	intergenic	C	T	-	-	-	-
AE008918	1083644	syn	C	T	V	V	BMEII1043	Isoleucyl-tRNA synthetase
AE008918	1085043	nsyn	A	G	V	A	BMEII1043	Isoleucyl-tRNA synthetase
AE008918	1086926	nsyn	G	A	P	S	BMEII1044	FAD synthase
AE008918	1088383	nsyn	A	G	V	A	BMEII1045	Predicted sugar phosphatases of the HAD superfamily
AE008918	1090427	syn	G	C	G	G	BMEII1048	Chaperonin GroEL (HSP60 family)
AE008918	1090467	nsyn	T	C	C	R	BMEII1048	Chaperonin GroEL (HSP60 family)

AE008918	1090873	nsyn	T	C	L	P	BMEII1048	Chaperonin GroEL (HSP60 family)
AE008918	1094455	intergenic	C	T	-	-	-	-
AE008918	1094573	nsyn	C	T	S	F	BMEII1053	Fucose permease
AE008918	1094627	nsyn	C	G	S	W	BMEII1053	Fucose permease
AE008918	1096483	nsyn	G	C	N	K	BMEII1054	ATP phosphoribosyltransferase
AE008918	1096510	nsyn	A	C	F	L	BMEII1054	ATP phosphoribosyltransferase
AE008918	1099331	nsyn	C	T	S	N	BMEII1057	Putative threonine efflux protein
AE008918	1100725	intergenic	G	A	-	-	-	-
AE008918	1107061	syn	A	G	L	L	BMEII1064	FAD/FMN-containing dehydrogenases
AE008918	1107871	nsyn	G	A	A	T	BMEII1065	Transcriptional regulator
AE008918	1109901	syn	C	T	F	F	BMEII1067	Uncharacterized protein conserved in bacteria
AE008918	1113862	syn	G	A	I	I	BMEII1071	NA
AE008918	1115095	syn	G	T	A	A	BMEII1073	Cytochrome B561
AE008918	1115409	nsyn	T	C	T	A	BMEII1073	Cytochrome B561
AE008918	1120807	nsyn	C	T	E	K	BMEII1079	NA
AE008918	1121136	intergenic	A	G	-	-	-	-
AE008918	1121554	nsyn	A	G	M	T	BMEII1080	Flagellar biosynthesis pathway, component FliP
AE008918	1124973	intergenic	A	C	-	-	-	-
AE008918	1124982	intergenic	T	G	-	-	-	-
AE008918	1125010	intergenic	T	G	-	-	-	-
AE008918	1125419	nsyn	G	T	P	Q	BMEII1085	Flagellar basal body P-ring biosynthesis protein
AE008918	1125737	nsyn	G	C	P	A	BMEII1086	Flagellar basal body rod protein
AE008918	1127740	intergenic	T	C	-	-	-	-
AE008918	1128412	syn	T	C	G	G	BMEII1090	3-hydroxyisobutyrate dehydrogenase and related beta-hydroxyacid dehydrogenases
AE008918	1132408	nsyn	C	T	A	V	BMEII1094	Nucleoside-diphosphate-sugar epimerases
AE008918	1132577	syn	T	G	L	L	BMEII1094	Nucleoside-diphosphate-sugar epimerases
AE008918	1133117	nsyn	A	C	D	A	BMEII1095	Ribulose-5-phosphate 4-epimerase and related epimerases and aldolases
AE008918	1134001	syn	C	A	A	A	BMEII1096	Permeases of the major facilitator superfamily
AE008918	1134128	nsyn	C	T	H	Y	BMEII1096	Permeases of the major facilitator superfamily
AE008918	1134157	syn	C	T	L	L	BMEII1096	Permeases of the major facilitator superfamily
AE008918	1134194	nsyn	G	A	G	S	BMEII1096	Permeases of the major facilitator superfamily
AE008918	1134717	intergenic	G	A	-	-	-	-
AE008918	1134729	intergenic	C	T	-	-	-	-
AE008918	1134746	intergenic	A	G	-	-	-	-

AE008918	1136571	nsyn	G	A	P	S	BMEII1098	AraC-type DNA-binding domain-containing proteins
AE008918	1138945	nsyn	C	A	R	L	BMEII1100	Uncharacterized protein conserved in bacteria
AE008918	1139873	nsyn	T	C	N	S	BMEII1101	Glycosyltransferases involved in cell wall biogenesis
AE008918	1140064	nsyn	T	C	D	G	BMEII1102	NA
AE008918	1140626	intergenic	C	T	-	-	-	-
AE008918	1143305	nsyn	C	T	M	I	BMEII1105	Flagellar biosynthesis/type III secretory pathway ATPase
AE008918	1143354	nsyn	G	A	T	I	BMEII1105	Flagellar biosynthesis/type III secretory pathway ATPase
AE008918	1145476	nsyn	C	T	Q	*	BMEII1109	Flagellar motor component
AE008918	1146460	syn	G	A	L	L	BMEII1110	Flagellar motor switch protein
AE008918	1146606	nsyn	C	T	T	M	BMEII1110	Flagellar motor switch protein
AE008918	1147644	nsyn	T	C	C	R	BMEII1111	NA
AE008918	1147695	nsyn	G	A	A	T	BMEII1111	NA
AE008918	1151688	intergenic	T	G	-	-	-	-
AE008918	1151879	syn	T	C	K	K	BMEII1117	Transcriptional regulator
AE008918	1153134	nsyn	T	C	F	L	BMEII1118	Multidrug resistance efflux pump
AE008918	1155793	intergenic	A	G	-	-	-	-
AE008918	1155960	intergenic	T	C	-	-	-	-
AE008918	1157192	intergenic	T	C	-	-	-	-
AE008918	1157307	intergenic	T	C	-	-	-	-
AE008918	1157972	syn	T	C	R	R	BMEII1121	ABC-type uncharacterized transport system, permease component
AE008918	1160725	nsyn	T	G	D	A	BMEII1124	6-phosphogluconate dehydrogenase
AE008918	1160796	syn	A	C	P	P	BMEII1124	6-phosphogluconate dehydrogenase
AE008918	1161363	syn	A	G	L	L	BMEII1124	6-phosphogluconate dehydrogenase
AE008918	1162155	syn	T	C	D	D	BMEII1125	Predicted dehydrogenases and related proteins
AE008918	1169892	nsyn	A	G	S	G	BMEII1132	Uncharacterized protein involved in exopolysaccharide biosynthesis
AE008918	1170192	nsyn	A	G	R	G	BMEII1132	Uncharacterized protein involved in exopolysaccharide biosynthesis
AE008918	1170648	nsyn	G	A	A	T	BMEII1132	Uncharacterized protein involved in exopolysaccharide biosynthesis
AE008918	1172463	intergenic	G	T	-	-	-	-
AE008918	1172885	intergenic	G	A	-	-	-	-
AE008918	1173764	nsyn	A	C	S	R	BMEII1134	Asp-tRNAAsn/Glu-tRNAGln amidotransferase A subunit and related amidases
AE008918	1175996	nsyn	T	A	L	Q	BMEII1137	ABC-type uncharacterized transport system, permease component
AE008918	1176285	syn	C	A	T	T	BMEII1137	ABC-type uncharacterized transport system, permease component
AE008918	1176731	nsyn	T	C	L	P	BMEII1137	ABC-type uncharacterized transport system, permease component
