# Supplementary material for: Comparative Genomic Analysis of Brucella melitensis Vaccine Strain M5 Provides Insights into Virulence Attenuation
Source: PLoS One. 2013 Aug 14;8(8):e70852. doi: 10.1371/journal.pone.0070852 (PMC3743847; doi:10.1371/journal.pone.0070852)
Supplement: Table S4 — Distribution of SNPs on virulence-related genes. (PDF) [file pone.0070852.s005.pdf]

Table S4: mutaions between M5 and 16M in virulence associated genes

Functional group	Gene	ORF of 16M	ORF of M5	SNP	Insersion	Deletion
**"Classical" virulence factors**						
Evelope molecules						
Lipopolysaccharide	*lpsB*	BMEI0509	M5GL002903	A1044G		
	lpsA	BMEI1326	M5GL001479	C278T(S93L)		
	wbdA	BMEI0997	M5GL000999	T347C(L116S)<br>G1103C(R368P)		
	wbpZ	BMEI1393	M5GL001558	G942A		
	pmm	BMEI1396	M5GL001561	T397C(F133L)<br>A903G		
	wbkA	BMEI1404	M5GL001566			
	rfbD	BMEI1413	M5GL001572			
	perA	BMEI1414	M5GL001573			
	wbpL	BMEI1426	M5GL001578	T1004G(L335R)		
	pgm	BMEI1886	M5GL002265	A752G(H251R)		
	manB	BMEII0899	M5GL000375	C279T		
	wbpW	BMEII0900	M5GL000393			
Outer membrance protein	*omp19*	BMEI0135	M5GL001873			
	omp25	BMEI1249	M5GL001304	T276C		
	omp10	BMEII0017	M5GL002026			
Peptidoglycan	*mgtA*	BMEI0271	M5GL003111	C142T(P48S)<br>T252C<br>G380A(R127K)		
	uppS	BMEI0827	M5GL002568			
	amiC	BMEI1056	M5GL001096	A96C<br>A545T(N182I)		
	dacF	BMEII0350	M5GL001341	G1261A(E421K)		
Secretion or transport system						
T4SS	*virB1*	BMEII0025	M5GL000739			
	virB2	BMEII0026	M5GL000746	G54A<br>G270C		
	virB3	BMEII0027	M5GL000747			
	virB4	BMEII0028	M5GL000748	A463C(K155Q)		

		virB5	BMEII0029	M5GL000749		
		virB6	BMEII0030	M5GL000750	A622G(T208A)<br>T1007A(F336Y)	
		virB7	BMEII0031	M5GL000751		
		virB8	BMEII0032	M5GL000752		
		virB9	BMEII0033	M5GL000753	A384G<br>C700G(R234G)	
		virB10	BMEII0034	M5GL000754		
		virB11	BMEII0035	M5GL000755		
		virB12	BMEII0036	M5GL000759	A402C	
		macA	BMEI0359	M5GL003245		
		bicA	BMEI0605	M5GL002807		840-15**
		tig	BMEI1069	M5GL001112		
		BMEI1258	BMEI1258	M5GL001314	C372A(H124Q)<br>T570C	
		BMEII0336	BMEII0336	M5GL001037		
		rbsC	BMEII0701	M5GL000164		
		ssuB	BMEII0799	M5GL000251		
		BMEII0923	BMEII0923	M5GL000394	C613G(P205A)	
	Flagella	*fliC*	BMEII0150	M5GL000876		
		fliF	BMEII0151	M5GL000880		
		motB	BMEII0154	M5GL000885	C188T(A63V)<br>A216G(I72M)<br>C659T(T220I)	
		flgE	BMEII0159	M5GL000892	A427G(I143V)	
		flhA	BMEII0166	M5GL000902		
		flgI	BMEII1084	M5GL000667		
**Regulation**		*pstP*	BMEI0190	M5GL001813	G1035A	
		asnC	BMEI0357	M5GL003208		
		lysR	BMEI0513	M5GL002899		
		LTTR	BMEI0116		T176C(L59P)	391-3
		ntrY	BMEI0867	M5GL002528	T252C<br>C1714A(Q572K)	
		spoT	BMEI1296	M5GL001447	C133G(R45G)<br>A198C	

		feuQ	BMEI1336	M5GL001489		
		feuP	BMEI1337	M5GL001490	G328A(A110T)	
		vsrB	BMEI1606	M5GL001756*		3209-1
		glnL	BMEI1786	M5GL002371		
		bvrS	BMEI2035	end of contig56		
		bvrR	BMEI2036	M5GL002033	G300T	
		nodV	BMEII0052	M5GL000775	G465A<br>T530C(V177A)	
		RpiR	BMEII0573	M5GL000054		
		divK	BMEII0659	M5GL000112		
		gntR	BMEII1066	M5GL000649		
		deoR	BMEII1093	M5GL000676		
		vjbR	BMEII1116	M5GL000698		
**Metal acquisition**		*mgtB*	BMEII0056	M5GL000797	C921T	
		dhbC	BMEII0077	M5GL000809	A196G(K66E)<br>G576A(M192I)	
		znuC	BMEII0177	M5GL000903	A149C(Q50P)	
		znuA	BMEII0178	M5GL000502	C250T(L84F)	
		nikA	BMEII0487	M5GL002228	T242C(F81S)<br>A531G	
		fbpA	BMEII0584	M5GL000066		
**Amino acid metabolism**						
	Sythesis	*lysA*	BMEI0084	M5GL001924	C739G(P247D)<br>C740A(P247D)<br>G1181T(G394V)	
		leuC	BMEI0157	M5GL001848		
		leuA	BMEI0451	M5GL002967	G878T (G293V)	
		carAB	BMEI0526	M5GL002888	G484A(A162T)	
		serB	BMEI0615	M5GL002796	G712C(V238L)	
		ilvI	BMEI0617	M5GL002793	T684C	
		ilvC	BMEI0624	M5GL002785	T162C	
		thrA	BMEI0725	M5GL002681	C1269G(D423E)	
		cysK	BMEI0933	M5GL000934	T308C(V103A)	

		thrC	BMEI1450	M5GL001603	G1323A	
		aroC	BMEI1506	M5GL001656	C684T<br>G946A(D316N)	
		hisD	BMEI1668	M5GL002448		
		metH	BMEI1759	M5GL002400	G523C(V175L)<br>C2041T(R681W)<br>C2924T(P975L)<br>C3375T	
		ilvD	BMEI1848	M5GL002306	A1235G(H412R)	
		hisF	BMEI2041	M5GL002031		
		glt1	BMEII0039	M5GL000760		
		gltD	BMEII0040	M5GL000771	G1659A<br>C1767T<br>C2058A<br>C3855T	
	Degradation	*gtyA*	BMEI1192	M5GL001243		560-1<br>753-562
		xfp	BMEII0881	M5GL000314		
	Transport	*livH*	BMEI0258	M5GL003098	G449T(G150V)<br>C909A(D303E)	
		dppA	BMEI0433	M5GL002985	A35G	
		artI	BMEI1104	M5GL001151		
		bacA	BMEI1553	end of contig46		
	Unknown	*aspC*	BMEI0516	M5GL002897	A450G	
		aspB	BMEI0626	M5GL002784		
**Sugar metabolism**						
	Catabolism	*pyc*	BMEI0266	M5GL003106	T135C<br>C156T<br>C186T<br>C885T<br>T1358A(L453Q)<br>A2846G(D949G)<br>G3220A(G1074S)	
		gloA	BMEI0730	M5GL002674		
		cbbE	BMEI1116	M5GL001163		
		galE	BMEI1237	M5GL001290		

	pgi	BMEI1636	M5GL001786			
	rbsK	BMEII0089	M5GL000852	G369C		
	eryC	BMEII0428	M5GL001391	G270A		
	eryB	BMEII0429	M5GL002174			
	galcD	BMEII0485	M5GL002175	C672T		
	zwf	BMEII0513	M5GL002230			
	mocC	BMEII0570	M5GL003242			
	glpK	BMEII0823	M5GL000289			
	gnd	BMEII1124	M5GL000706	T267C<br>T834G<br>A905C(D302A)		
	mosA	BMEI1301	M5GL001452	T120A<br>A445G(I149V)		
	ndvB	BMEI1837	M5GL002321	C2556T<br>A2784G<br>A3587C(D1196A)<br>T3588A(D1196A)<br>C3589T(H1197S)<br>A3590C(H1197S)<br>T3594C<br>C3774G(G1258E)<br>G3775C(G1259R)<br>T3780G<br>C3782G(P1261R)<br>C4638T<br>G4996A(A1666T)<br>G6600T(E2200D)<br>C7557G	3638-1	3631-1
	gtrB	BMEII1101	M5GL000684	A95G(N32S)		
Transport	*mosC*	BMEI0267	M5GL003107			
	malK	BMEI1713	M5GL002417	C1026T		
	exsA	BMEI1742	M5GL002418			
	dbsA	BMEII0300	M5GL001018	G1668A<br>G1714A(G572R)		1-1053
	araG(gguA)	BMEII0361	M5GL001357			
	ugpA	BMEII0591	M5GL000071			

	ugpA	BMEII0624	M5GL000080	
	gluP	BMEII1053	M5GL000635	C50T(S17F)
				C104G(S35W)

DNA/RNA metabolism

Synthesis	*hpt*	BMEI0082	M5GL001926	
	purE	BMEI0296	M5GL003138	
	dut	BMEI0358	M5GL003209	
	pth	BMEI0480	M5GL002936	G222A
	miaA	BMEI0616	M5GL002795	T629A(V210E)
	purL	BMEI1127	M5GL001174	T918C
				A1483C(T495P)
				A1489C(T497P)
	purM	BMEI1240	M5GL001294	G189A
				T339C
	purN	BMEI1241	M5GL001295	
	pyrC	BMEI1281	M5GL001432	
	purF	BMEI1488	M5GL001638	
	purD	BMEI1519	M5GL001668	T95C(L32P)
				G919A(V307I)
	pyrD	BMEI1611	M5GL001761	A138G
	pyrC	BMEII0669	M5GL000151	
	pyrB	BMEII0670	M5GL000162	
	ndrI	BMEII0931	M5GL000414	
	nrdH	BMEII0932	M5GL000422	
Catabolism	*xseA*	BMEII0527	M5GL003227	C810T
				A1416C
				G1465A9V489I)
Repair	*xerD*	BMEI0040	M5GL001969	T90C
	alkA	BMEI0382	M5GL003274	
	recA	BMEI0787	M5GL002614	
	rpsA	BMEI1915	M5GL002152	G240A
	mutM	BMEI1946	M5GL002123	
	aidB	BMEII0671	M5GL000163	

	Regulation	*mgps*	BMEI0275	M5GL003116	C177T<br>T366G<br>T1476C<br>A1647T<br>C1681T(L561F)<br>C1818T<br>A1820C(D607A)<br>T2388C	
		rpoA	BMEI0781	M5GL002622	C183T<br>G855T	
		tldD	BMEI1468	M5GL001622	G65A(S22N)	
**Vitamines /cofacteurs**		*cobB*	BMEI0705	M5GL002705	C442T(L148F)	
		dxps	BMEI1498	M5GL001648	G567A	
		hemH	BMEII0018	M5GL000738	C483T	
		cobW	BMEII0308	M5GL001017	C1028T(P343L)	735-15
		pncA	BMEI0545	M5GL002871	C237T<br>A1006C(M336L)	
**Stress proteins /Chaperones**		*ppiD*	BMEI0845	M5GL002549		
		hfq	BMEI0872	M5GL002524		
		lon	BMEI0876	M5GL002520	T2109C	
		htrA	BMEI1330	M5GL001483		
		dnaJ	BMEI1513	M5GL001664	A303C<br>G398A(G133D)<br>A555G	
		dnaK	BMEI2002	M5GL002066	A37C(M13L)	
**Oxidoreduction**		*dsbB domain*	BMEI0384	M5GL003276	G120A(M40I)	
		trkH	BMEI0512	M5GL002900		
		caiB domain	BMEI0898	M5GL002498	G201T(W67C)<br>G552A	
		dsbA domain	BMEI1040	M5GL001080	T726C	

	dsbA domain	BMEI1060	M5GL001101	T483C		
	dsbA domain	BMEI1440	M5GL001592			
	cysI	BMEI1766	M5GL002393*			1504-4<br>1672-88
	cycY	BMEI1849	M5GL002305			
	fdhA	BMEII0378	M5GL001371	G1112A(R371Q)<br>G1164A(M388I)<br>C1343T(A448V)<br>G1598A(R533H)<br>T2139C		
	sodC	BMEII0581	M5GL000057			
	cydB	BMEII0759	M5GL000197			
	cydC	BMEII0761	M5GL000248	C1404G		809-12
	cydD	BMEII0762	M5GL000250			
	narG	BMEII0950	M5GL000529*		0-851	72-1,176-1
	norE	BMEII1001	M5GL000584			
	caiB domain	BMEII1019	M5GL000603	T686C(V229A)		
**nitrogen metabolism**	*glnA*	BMEI0979	M5GL000982	T1075G(F359V)		
	nifS	BMEI1043	M5GL001083	C274T(P92S)		
	glnD	BMEI1804	M5GL002353	T427C(Y143H)<br>G627C<br>T2544C		
**Other genes**	*Tetratricopeptide repeat family protein*	BMEI1531	M5GL001680			
	pheB	BMEII0136	M5GL000874	T271C(F91L)		
	Zinc protease	BMEII1037	M5GL000620	C154T(P52S)		
**Unknown function**		BMEI0085	M5GL001923			
		BMEI0455	M5GL002963	G500T(R167L)		
		BMEI0671	M5GL002741	C226T(R76C)<br>T960C<br>T1065C		
		BMEI1229	M5GL001281			

BMEI1339	M5GL001492		
BMEI1361	M5GL001524		
BMEI1443	M5GL001596		
BMEI1448	M5GL001601		
BMEI1658	M5GL002486		
BMEI1809	M5GL002349	A568G(I190V)	
BMEI1844	M5GL002313	A180G<br>A321G	
BMEI1859	M5GL002295		
BMEI1879	M5GL002272		
BMEI1902	M5GL002247		
BMEII0128	M5GL000860	A31C(M11L)<br>C589T(H197S)<br>A590C(H197S)<br>T591G(H197S)	
BMEII0274	M5GL001016	C389T(A130V)	
BMEII0318	M5GL001026	G727A(A243K)<br>C728A(A243K)<br>A770G(*257W)	771-446
BMEII0626	M5GL000110		
BMEII0935	end of Contig20		
BMEII1045	M5GL000628	T380C(V127A)	

*the mutations caused frameshift downstream

**location of indel - length of fragment
