# Supplementary material for: Comparative Genomic Analysis of Brucella melitensis Vaccine Strain M5 Provides Insights into Virulence Attenuation
Source: PLoS One. 2013 Aug 14;8(8):e70852. doi: 10.1371/journal.pone.0070852 (PMC3743847; doi:10.1371/journal.pone.0070852)
Supplement: Table S5 — RDs aligned to reference sequences. (PDF) [file pone.0070852.s006.pdf]

Table S5: RDs aligned to references

Chromosome	RD #	RD Length	Reference									
			AE008917	NC_007618	NC_006932	NC_004310	NC_013119	NC_012441	NC_010742	NC_010169	NC_010103	NC_009505
I	RD1	337	337,1*	335,1	335,1	336,1	336,1	337,1	335,1	336,1	336,1	334,1
	RD2	309	309,1	297,1	297,1	309,1	309,1	309,1	297,1	309,1	309,1	308,1
	RD3	906	906,1	903,1	903,1	903,1	852,1	905,1	903,1	903,1	903,1	902,1
	RD1a**	461	461,1	459,1	459,1	460,1	460,1	461,1	459,1	460,1	460,1	458,1
	RD4	342	342,1	179,1	179,1	334,1	335,2	180,1	179,1	334,3	236,2	58,1
	RD5	883	883,1; 733-883 (139),1	733-883 (139),2	733-883 (139),2	733-883 (139),2	883,1; 733-883(138),1	733-883 (139),2	733-883 (139),2	882,1; 733-883(147),1	733-883 (139),2	879,1
II			AE008918	NC_007624	NC_006933	NC_004311	NC_013118	NC_012442	NC_010740	NC_010167	NC_010104	NC_009504
	RD6	306	306,1	305,1	305,1	305,1	305,1	303,1	305,1	304,1	305,1	305,1
	RD7	330	330,1	328,1	328,1	329,1	329,1	330,1	328,1	330,1	328,1	329,1
	RD8	719	719,1	718,1	718,1	719,1	719,1	718,1	718,1	718,1	718,1	719,1
	RD9	335	335,1	330,1	330,1	330,1	330,1	329,1	330,1	329,1	330,1	330,1
	RD10	327	327,1	326,1	326,1	326,1	325,1	326,1	326,1	326,1	325,1	324,1
	RD11	321	321,1	315,1	315,1	312,1	315,1	315,1	315,1	312,1	312,1	314,1

*match,hit

**RD1a: included in RD1

Reference List

AE008917, AE008918: *Brucella melitensis* 16M
NC_007618, NC_007624: *Brucella abortus* 2308
NC_006932, NC_006933: *Brucella abortus* 9-941
NC_004310, NC_004311: *Brucella suis* 1330
NC_013119, NC_013118: *Brucella microti* CCM 4915
NC_012441, NC_012442: *Brucella melitensis* ATCC 23457
NC_010742, NC_010740: *Brucella abortus* S19
NC_010169, NC_010167: *Brucella suis* ATCC 23445
NC_010103, NC_010104: *Brucella canis* ATCC 23365
NC_009505, NC_009504: *Brucella ovis* ATCC 25840
